# *Canis* STR‐Seq: A Universal Approach for Non‐Invasive Genetic Monitoring of Wolves and Coyotes

**DOI:** 10.1002/ece3.73300

**Published:** 2026-03-16

**Authors:** Emily Walker, Brent R. Patterson, Glen A. Rutledge, Linda Y. Rutledge

**Affiliations:** ^1^ Ministry of Natural Resources and Forestry, Wildlife Research and Monitoring Section Peterborough Ontario Canada; ^2^ Environmental and Life Sciences Trent University Peterborough Ontario Canada; ^3^ Independent Researcher Victoria British Columbia Canada; ^4^ Forest and Conservation Sciences University of British Columbia Vancouver British Columbia Canada

**Keywords:** *Canis*, conservation, genotype‐by‐sequencing, microsatellites, non‐invasive monitoring, wolves

## Abstract

Population genetic studies have traditionally relied on data from short tandem repeat (STR) markers, known as microsatellites, to produce individual genotypes used in population genetics research. However, size fragment analysis from traditional capillary electrophoresis presents scoring challenges and limits data comparisons among labs. Here, we present a new, cost‐effective universal microsatellite genotype‐by‐sequencing assay for *Canis* species that allows for unambiguous allele calls, flags homoplasy for more accurate assignment tests and estimates of diversity, and improves genotyping output from low‐template DNA. We note size homoplasy in 18 of 26 loci, with the number of alleles being 32% higher in the dataset that included sequence mutations (Na_mut_ = 334) compared to the dataset on the basis of size alone (Na_len_ = 253). Assignment tests with Bayesian cluster analysis were similar for both datasets, although 64 of 84 samples had higher assignment values to their primary cluster when mutations were considered. We document and code a list of sequence mutations associated with each locus and propose a framework for building an accessible, universal STR dataset for wolves, coyotes, and dogs that improves cluster assignments and admixture estimates in a system with complex demography and hybridization patterns. Overall, the assay provides an improved microsatellite method of genetic monitoring to aid conservation of wolf populations.

## Graphical abstract

We present a new, universal microsatellite genotype‐by‐sequencing assay for *Canis* species that allows for unambiguous allele calls, flags homoplasy for more accurate assignment tests, and improves genotyping output from low‐template DNA. We suggest, as others have done previously, that microsatellites are still effective, affordable, and sometimes preferable markers for use in population genetics studies and that GBS technology has enabled a microsatellite renaissance. The focus on a universal approach to provide open access reference datasets provides a long‐awaited consolidation of genotyping methodology that promotes a cooperative framework for global wolf conservation.

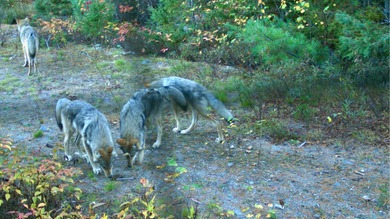

**Abbreviations**
allele_len: allele call based on length only
allele_mut: allele call based on length and point mutations in MRA, FFR, RFR
CE: capillary electrophoresis
FFR: forward flanking region
GBS: genotype‐by‐sequencing
MRA: microsatellite repeat array
PCR: polymerase chain reaction
RFR: reverse flanking region
Sc: scat
SNP: single‐nucleotide polymorphism
STR: short tandem repeat
Ti: tissue

## 1 Introduction

Unprecedented biodiversity loss from global climate change (Bongaarts 2019) and direct anthropogenic drivers (Jaureguiberry et al. 2022) presents an urgent challenge to wildlife professionals and policymakers tasked with mitigating these impacts and conserving species at risk. For over 50 years, population genetics has played a key part in wildlife conservation (Charlesworth and Charlesworth 2017) with genetic monitoring being critical for understanding a wide range of ecological patterns, including (but not limited to) species distribution, hybridization, effective population size, and other indicators of extinction risk. With the arrival of cost‐effective high‐throughput sequencing (HTS) technology, assessment of genome‐wide genetic variation has become increasingly important for wildlife conservation and management (Hohenlohe et al. 2021; Kardos et al. 2021). Furthermore, non‐invasive genetic monitoring has become a common, and often preferable or necessary, approach to understanding population dynamics (Ferreira et al. 2018; Zemanova 2021). The application of HTS to non‐invasive monitoring provides an opportunity to expand efforts to inform conservation policy with minimal impact on vulnerable and elusive species (Carroll et al. 2018; Ibouroi et al. 2021) like wolves (Valière et al. 2003; Caniglia et al. 2014; Rutledge et al. 2017; Dufresnes et al. 2019; Murphy et al. 2019).

**FIGURE 1:**
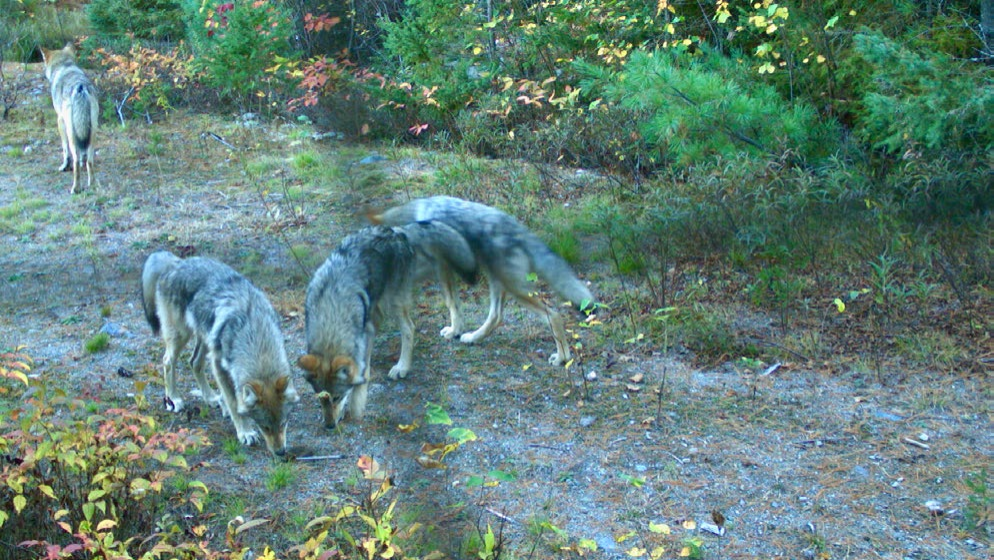
Eastern Wolves. Trail camera image of eastern wolves (*Canis* sp. *cf. lycaon*) in Killarney Provincial Park in central Ontario, Canada.

Wolves (Figure 1) have been extirpated from most of their original range but remain one of the most widely studied large carnivores (Ripple et al. 2014) because, in large part, of their iconic status and widespread vulnerability to human influences (Marco et al. 2014; Murray et al. 2024). Genetic monitoring of global wolf populations has been an important conservation tool for over 20 years (Wilson et al. 2000; De Barba et al. 2017; Dufresnes et al. 2019) with non‐invasive approaches becoming increasingly utilized (Rutledge et al. 2009; Caniglia et al. 2014; Stansbury et al. 2016). Although size fragment analysis of short tandem repeat (STR) microsatellite markers by capillary electrophoresis (CE) has long been the approach to generate individual genotypes for population genetics, the methodology has several limitations. The main criticisms relate to scoring challenges and low template allelic dropout that cause errors in the data, undetected size homoplasy of alleles, and a lack of cross‐compatibility of data generated in different labs (Estoup et al. 2002; De Barba et al. 2017). These challenges pushed many researchers to embrace genotype‐by‐sequencing of single nucleotide polymorphisms (SNPs) (Campbell et al. 2015; Fitak et al. 2015; von Thaden et al. 2017; Eriksson et al. 2020; Zimmerman et al. 2020; Hayward et al. 2022). Although SNPs are often the favored marker for fine‐scale genetic resolution (Timm 2020), microsatellites are still relevant for population genetics (Hauser et al. 2021) and sometimes preferable to SNPs. For example, different SNP markers on the basis of high minor allele frequencies (MAFs) are recommended for different tasks (individual identification vs. parentage vs. population delineation) or different populations (Giangregoria et al. 2023; Harmoinen et al. 2025; Hervey et al. 2025). Microsatellite markers, in comparison, are more broadly applicable to various tasks and populations because of their inherent polymorphism, which also allows far fewer loci to be used than SNPs to achieve the same resolution (Morin et al. 2009; Haasl and Payseur 2011). Also, microsatellites mutate more rapidly (Haasl and Payseur 2011) to reveal patterns of recent divergence; they outperform SNPs in parentage analyses (Giangregoria et al. 2023), especially for species with low genetic diversity (Hauser et al. 2021), and there are extensive primer resources available from past microsatellite development. Furthermore, the issues previously described with using microsatellites and CE analysis of fragment size are overcome by adopting a novel approach that uses HTS of microsatellites amplified in a single multiplexed polymerase chain reaction (PCR) (Vartia et al. 2015; De Barba et al. 2017; Bradbury et al. 2018; Šarhanová et al. 2018; Curto et al. 2019; Salado et al. 2021).

The approach of genotype‐by‐sequencing (GBS) of microsatellites with HTS was first used in forensic research (Fordyce et al. 2011; Scheible et al. 2011; Neste et al. 2012) but has become increasingly attractive to population genetics research. The method has been applied to a wide range of species and sample types (Vartia et al. 2015; De Barba et al. 2017; Bradbury et al. 2018; Šarhanová et al. 2018; Curto et al. 2019; Tibihika et al. 2019; Eriksson et al. 2020; Gallagher et al. 2022; Lepais et al. 2022; Liu et al. 2024), creating a microsatellite revival in the field. Its allure is warranted because GBS is faster, cheaper, more accurate, and more transferable than traditional CE methods. The pitfalls of using microsatellites with CE fragment size analysis are largely overcome with analysis of microsatellite sequences, even for low template DNA (De Barba et al. 2017), and allow continued use of previously developed microsatellite markers.

Although some GBS SNP assays have been developed for wolves (Stronen et al. 2022; Hervey et al. 2025) and coyotes (Eriksson et al. 2020), we know of only one instance where sequencing microsatellites has been used for genotyping *Canis* species (Salado et al. 2021). That research, however, focused on evaluating software packages for genotyping on the basis only of length and did not incorporate the added allelic diversity obtained by including sequence mutations. Here, we present a rigorously tested assay for genotype‐by‐sequencing of STRs for *Canis* spp. (*Canis* STR‐seq) that provides a universal genotyping approach that minimizes scoring error, mitigates allelic dropout for low template samples, identifies homoplasy to provide accurate measures of diversity, and provides a baseline open access database for reference populations that can be used in combination with new datasets from any lab using the assay as described. This assay is intended to complement existing GBS SNP assays to broaden the population genetics toolkit for wolf conservation. We also provide a reference of mutation codes that reflect size homoplasy of alleles, and we propose a standard coding system for each mutation to enable inclusion of this diversity in various downstream applications such as parentage tests, estimates of heterozygosity, inference of population structure, and ancestry. The assay can be used with any *Canis* species to understand distribution, gene flow, hybridization, and relatedness and infer evolutionary history at different timescales (Šarhanová et al. 2018). Overall, the assay embraces the microsatellite revival and answers the call for harmonization of genetic markers and standardization of GBS assays (de Groot et al. 2016) for improved conservation of wolf populations.

## 2 Materials and Methods

### 2.1 Sample Selection and Screening

Samples for this study included newly extracted and archived DNA samples stored frozen at −20°C in the Ontario Ministry of Natural Resources genomics laboratory at Trent University, Peterborough Ontario (Data S7). For tissue, blood, and hair samples (*n* = 88) (quantified, high template tissue [Ti] samples), we included representative samples from various *Canis* species, including domestic dogs ( *C. lupus familiaris* ; *n* = 6), western gray wolves from Alberta ( *C. lupus* ; *n* = 6), western coyotes from Alberta ( *C. latrans* ; *n* = 6), eastern wolves from Central Ontario (*C*. sp. *cf. lycaon*; *n* = 6), Great Lakes‐boreal wolves from northern Ontario ( *C. lupus* × *lycaon*; *n* = 6), and eastern coyotes from Southern Ontario ( *C. lycaon* × *latrans*; *n* = 6) as well as unknown or admixed samples from Southern Ontario (*Canis* sp.; *n* = 52). For scat samples (*n* = 22) (low template scat (Sc) samples), we used previously collected samples that had been stored at −20°C until processing.

**FIGURE 2:**
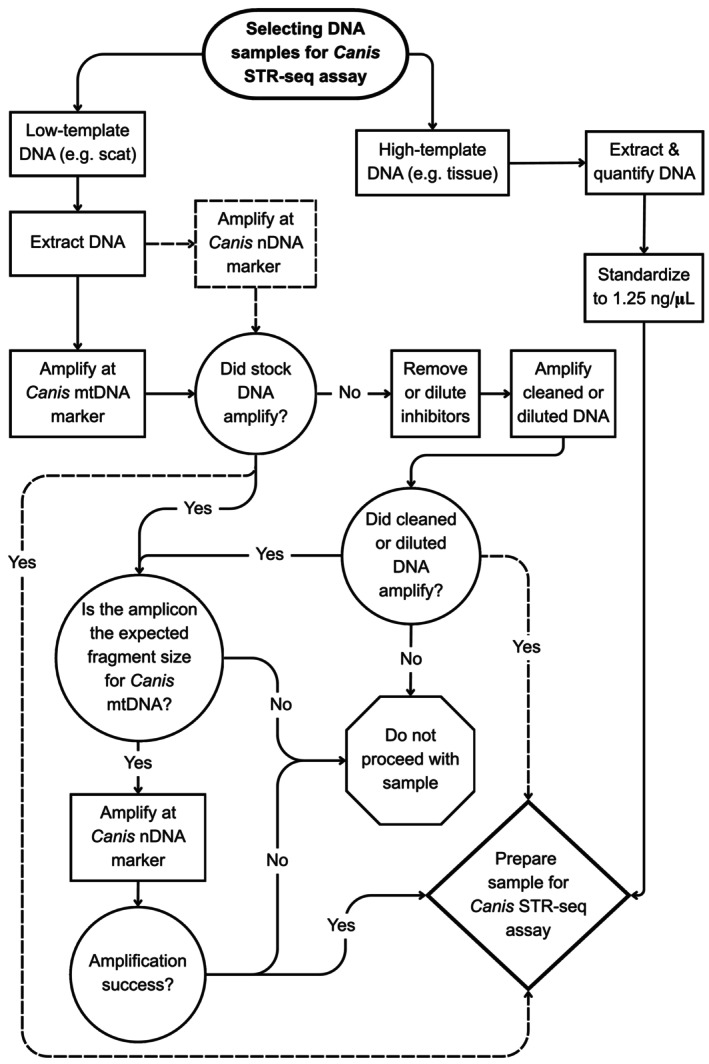
Sample Triage. Flow chart showing sample triage workflow for inclusion in the *Canis* STR‐seq assay. The dotted line represents an alternate workflow that avoids amplification at mtDNA and goes directly to amplification with a *Canis*‐specific nuclear DNA (nDNA) marker (See Data S2).

We extracted DNA with either the EZNA Tissue DNA Kit (OMEGA) or DNeasy Blood and Tissue extraction kit (Qiagen) according to the manufacturer's protocols with some modifications (see Data S2). For sequencing, we only included scat samples that passed triage stages (Figure 2), which included: (1) successful amplification at a *Canis* mitochondrial DNA control region marker to ensure samples were from *Canis* species and not red fox ( *Vulpes vulpes* ), followed by (2) successful amplification at a nuclear microsatellite marker to ensure sufficient amplification of nuclear DNA (Data S2).

### 2.2 STR Marker Selection, Primer Redesign and Preliminary Testing

A total of 33 microsatellite markers were initially selected from previously published work, targeting common di‐nucleotide and tetranucleotide markers that amplify fragments ≤ 220 bp (Data S5). We also included a *Canis* Amelogenin sex marker (Yan et al. 2013). Forward and reverse primers were re‐designed against the ROS_Cfam_1.0 dog reference genome (GCF_014441545.1) in Geneious Prime 2024.0.7 (https://www.geneious.com) to target a universal product length of 100–150 bp, with primers between 17 to 24 bp, melting temperature 58°C–62°C with maximum 5°C difference between paired primers, 40%–60% GC content, while avoiding hairpins and dimers. Targeting common specificity and shorter target length across markers was done to: (a) improve equal amplification across loci in a single amplification, (b) allow for complete coverage in both directions with paired‐end 300 cycle sequencing, and (c) create shorter fragments that improve outcomes on fragmented DNA from noninvasive or historical samples. We wanted to use this assay on DNA from fecal samples that include a potential mixture of prey items, so we also tested the new primer combinations in Primer‐BLAST (Ye et al. 2012) (with mismatch set to ≤ 3 bp and maximum fragment size of 1000 bp) against red fox ( *Vulpes vulpes* ), white‐tailed deer ( *Odocoileus virginianus* ), elk ( *Cervus canadensis* ), and beaver ( *Castor canadensis* ), as well as other regions of the dog genome (Data S5). As a first screen, we excluded two primer pairs (Cfam_STR006 and Cfam_STR028) from further assessment because the fragment was either too large or had poor amplification, leaving 31 STR primer pairs and the Amelogenin sex marker at this stage.

### 2.3 DNA Library Preparation

We prepared two different sequencing libraries: a high template library with DNA extracted from tissue samples (Ti) (*n* = 88; Data S7), and a low template library with DNA extracted from scat samples (Sc) that passed triage (*n* = 22; Data S7). Both libraries included additional no‐template negative controls throughout the process from extraction to sequencing. Sequencing libraries were created for each sample on the basis of a two‐step protocol. An initial single multiplex PCR amplified 31 microsatellite loci and the sex marker (PCR1), followed by a second reaction (PCR2) to ligate Nextera XT unique dual indexes (N7XX and S5XX), allowing for unambiguous identification of each individual sample after sequencing. For PCR1, we used Platinum Multiplex PCR Mastermix (Cat No. 4464269; Applied Biosystems) in a single 25 μL reaction (2 μL of 1.6 μM primer mix, 4 μL of DNA, 12.5 μL of mastermix, and 6.5 μL water). PCR1 was run with the following conditions: initial activation step of 95°C for 15 min followed by 30 cycles of 94°C for 30 s, 63°C for 90 s and 72°C for 90 s, with a 10‐min final extension at 72°C. A post‐PCR cleanup was performed with magnetic AMPure XP Beads (Beckman‐Coulter) at a 1.0× ratio of beads to DNA with a final elution volume of 50 μL. Indexes were then added to the cleaned product during PCR2, which included 5 μL of cleaned PCR1 amplicon product, 25 μL of NEBNext Ultra II Q5 PCR Mastermix (Cat. No. M0544L, New England Biolabs), 5 μL of Nextera XT Index 1 (N7XX), 5 μL of Nextera XT Index 2 (S5XX) (Cat. No. FC‐131‐2001; Illumina) and 10 μL of molecular grade water for a final reaction volume of 50 μL. PCR2 was run with the following conditions: an initial activation of 98°C for 30 s followed by 8 cycles of 98°C for 10 s, 67°C for 75 s, and a final extension of 67°C for 5 min. The PCR2 product was cleaned with magnetic AMPure XP Beads (Beckman‐Coulter) at a 1.0× ratio of beads to DNA and eluted in a final volume of 25 μL elution buffer. Individual amplicon libraries were then quantified with the Quant‐iT PicoGreen dsDNA kit (Cat. No. P7589; Thermofisher) and the Infinite 200 Pro plate‐reader (Tecan) to measure fluorescence. Libraries were standardized to 4 nM and pooled equimolar into a final combined 4 nM library—one for tissue and one for scat. The concentration of the pooled library was confirmed with Quantifluor ONE dsDNA system (Cat. No. E4871; Promega) and the Quantus handheld fluorometer (Promega) according to the manufacturer's protocol. The expected size distribution of the pooled library was confirmed with an E‐Gel power snap electrophoresis system (Cat. No. G8300; Thermofisher) alongside a 50 bp DNA ladder (Cat. No. 10488090; Thermofisher).

### 2.4 Sequencing, Genotyping and Errors

Pooled libraries were prepared for paired end 2 × 150 bp high throughput sequencing on the Illumina MiSeq platform. The libraries were diluted to 6 pM with hybridization buffer, denatured using sodium hydroxide and spiked with 30% PhiX control v3 (Cat. No. FC‐110‐3001; Illumina) to account for low complexity libraries. Demultiplexing on the basis of individual indexes was done automatically with Illumina MiSeq software. Tissue (Ti) samples were sequenced with a MiSeq Standard Kit v2 (300 cycles) (Cat. No. MS‐102‐2002; Illumina) and the scat (Sc) samples were sequenced with a MiSeq Reagent Micro Kit v2 (300 cycles) (Cat. No. MS‐103‐1002; Illumina). We used Seq2Sat in the SatAnalyzer toolkit (Liu et al. 2024) to analyze sequence reads and call genotypes on the basis of the read quality, depth‐of‐read, read ratio, sequence composition and length, followed by visual inspection and manual correction of calls. SatAnalyzer parameters were set as follows: number of mismatches for primer pair: 2; minimum reads for an allele: 10; max % mismatches for flanking region: 0.5; reads ratio of top 2 largest alleles when size difference = 1ssr unit: 0.7; reads ratio of top 2 largest alleles when size difference = 2ssr units: 0.2; min % against largest allele reads for an allele: 10; max ratio of two allele variants: 1.5; minimum reads quality score: 20; minimum length of a read: 80 bp; max mismatches for sex primer pair: 2, max mismatches for sex ratio: 2, minimum number of reads for sex alleles: 20, reads ratio of Y/X alleles: 0.02, minimum number of reads for each sex variant: 10, and number of threads: 2. On the basis of preliminary assessments, we excluded five additional loci because of evidence of 1 bp allele shifts (Cfam_STR003, Cfam_STR025) and challenges associated with visualizing and scoring tetranucleotide markers (Cfam_STR005, Cfam_STR023, Cfam_STR024) in SatAnalyzer, which was developed for dinucleotide markers (and does not display tetranucleotide stutter patterns as effectively), leaving 26 microsatellites and the sex marker (Data S5) for inclusion in assessment of genotyping errors.

To validate the new STR primers (*n* = 26) and compare CE genotypes with genotypes from the new *Canis* STR‐seq assay, we amplified a subsample of the *Canis* tissue DNA (*n* = 15) in simplex reactions with the newly designed primers with the forward primer fluorescently tagged with 6FAM for size fragment analysis by CE on an ABI3730 Genetic Analyzer (Applied Biosystems) (Data S2). CE genotypes were determined by automatic scoring in Genemarker v7.1 (SoftGenetics) followed by visual inspection and manual corrections where required. On the basis of comparative peak morphology and genotype calls from CE simplexes and the STR‐seq for 15 samples (Data S3), we consolidated the genotype calls from both methods and established scoring criteria for the *Canis* STR‐seq assay (Figure S5). Note that the Peak Morphology Comparisons document provides a visual reference for those wanting to use the STR‐seq approach but are more familiar with CE peak morphology.

### 2.5 Genotyping Error

Both the tissue and scat STR‐seq runs were independently analyzed and scored with SatAnalyzer (Liu et al. 2024) by two different individuals familiar with the data and scoring criteria. We used the R package allelematch v2.5.4 (Galpern et al. 2012) to identify duplicate samples and identify scoring errors in both datasets. Sample CP‐2023‐007 and sample CP‐2023‐071 were identified as the same individual, so sample CP‐2023‐071 was excluded from further analysis. Also, samples CP‐2023‐009 and CP‐2023‐064 were identified as the same individual, so sample CP‐2023‐064 was also excluded. Finally, samples CP‐2023‐058 and CP‐2023‐059 were identified as the same individual as sample CP‐2023‐008, so CP‐2023‐058 and CP‐2023‐059 were excluded from further analysis. These duplicates were originally included without prior knowledge, but comparison of data from the new assay with previous CE genotyping confirmed them as duplicates. For the tissue samples, this left 84 individuals with two independent scores at 26 loci and the sex marker.

For the scat dataset (*n* = 22), two samples (CP‐2023‐146 and CP‐2023‐147) had mixed profiles on the basis of independent assessments and were excluded from further analysis. Initial assessment showed that CP2023‐2023‐087 and CP2023‐089 were the same individual so CP‐2023‐089 was excluded from further analysis. Also, three paired scores mismatched only because of missing data (CP‐2023‐099, CP‐2023‐111, and CP‐2023‐129), suggesting poor quality, so these three low quality samples were also excluded, leaving 16 scat samples for further analysis. Note that samples CAN004247 and CAN004248 were positive tissue controls included in the scat library, so they were removed from the error rate calculations for the scat samples.

We assessed scoring error for the multilocus dataset (with an approach used for previous microsatellite data, Rutledge et al. 2017) of the 26 microsatellites and one sex marker for 84 tissue and 16 scat samples. To assess scoring errors in the tissue data, we ran the dataset of 168 genotypes (84 × 2 independent scores) through allelematch (Galpern et al. 2012), allowing for 4 mismatches as recommended by the amUniqueProfile plot. Similarly, for the scat samples, we ran 32 genotypes (16 × 2 independent scores) through allelematch allowing for 6 mismatches as recommended by the amUniqueProfile plot. For each dataset, we calculated the error rate for each locus, the error rate across all loci, the error rate when loci with an error rate > 0.08 in the tissue data were removed, and the error rate when loci with an error rate > 0.05 in the tissue data were removed. We also assessed differences in sex identification between the independent SatAnalyzer calls and compared that with known sex from previous sex identification on the basis of CE and/or field sex ID where available. Consensus genotypes for the tissue and scat data were determined by comparisons and examination of the output from SatAnalyzer and discussions about why the errors occurred.

### 2.6 Microsatellite Size Homoplasy

We assessed size homoplasy for each allele at each locus for the tissue samples on the basis of combined sample output files from SatAnalyzer (i.e., sampleID_genotypes_mra_final.txt output files). These files show where mutations occur in the microsatellite repeat array (MRA), the forward flanking (FF) and reverse flanking (RF) regions. Although it is possible for sequencing error to imply an allele with homoplasy, it is rare for this to be carried through to an allele call and the evidence of the same mutations across multiple samples suggests that the mutations are representative of homoplasy. We created a custom Python script (allele_muts.py; Rutledge and Rutledge 2025) to assess and code variation in the MRA, FF and RF regions. The output incorporates sequence mutations into the allele call (allele_mut) by assigning unique 2‐digit codes to mutations in each region, resulting in an 8 or 9 digit allele call (depending on whether or not the allele length is greater than or less than 100 bp) with the following format: AAAMMFFRR, where AAA is the allele code on the basis of length (allele_len), MM is the MRA mutation code, FF is the snpsFF mutation code and RR is the snpsRF mutation code (Data S1). For example, if a sample at locus Cfam_STR001 had an allele call of 96 with no mutations in MRA, snpsFF or snpsRF, then the *allele_mut* call would be 96000000. Similarly, if a sample at locus Cfam_STR007 had an allele call of 109 but had the second documented MRA mutation (code = “02”), no mutations at snpsFF (code = “00”), and the third documented snpsRF mutation (code = “03”), then the allele_mut call would be 109020003. These data, when compiled, account for diversity because of homoplasy at each allele of each locus, are easily decipherable and can be completely decoded by referencing the mutation codes (Data S4).

### 2.7 Genetic Diversity and Population Structure

We used consolidated genotypes of 84 individuals at 26 loci from the tissue (Ti) dataset to calculate estimates of diversity and heterozygosity with GenAlEx 6.503 (Peakall and Smouse 2012) and infer population structure on the basis of the standard allele dataset that represents length only (allele_len) and the mutations dataset that incorporated sequence mutations into the allele calls (allele_mut); we also ran the analysis without the dog reference group (*n* = 6) to more fully understand how those data might impact assignment values, and on the allele_len scat (Sc) dataset to assess ancestry on the basis of noninvasive samples. We inferred population structure and admixture with the F model for correlated allele frequencies in Structure v2.3.4 (Pritchard et al. 2000; Falush et al. 2003; Hubisz et al. 2009) with 5 runs at each of *K* = 1 − *K* = 10 with 150,000 burnin and 1,500,000 iterations. We used StructureSelector (Li and Liu 2018) to estimate optimal clusters with multiple methods and implemented CLUMPP v1.1.2 (Jakobsson and Rosenberg 2007) with the GREEDY option and 1000 random input order repeats to combine Structure output files. Final assignment values (for both the allele_len and allele_mut Ti datasets and the allele_len Sc dataset) were recorded (Data S6) and plots were visualized as output from StructureSelector on the basis of optimal clusters (Figure S8). We report assignments for the allele_len and allele_mut Ti samples, with and without the dog reference group, and for the allele_len Sc samples, on the basis of *Q* ≥ 0.8 as assigned to a known cluster and *Q* < 0.8 considered admixed.

## 3 Results

### 3.1 Sequencing, Genotyping and Errors

Sequencing output for the tissue samples included 34,181,810 total reads with 16,741,956 reads assigned to the *Canis* STR‐seq amplicons (average 53.5% assigned reads per sample). Output for the scat samples included 7,282,378 reads, with 107,238 assigned to the assay amplicons (average 50.1% assigned reads per sample). Unassigned reads were attributed to low‐quality reads, excess adapter sequences, and 30% PhiX control spike (to account for low complexity libraries). Reads were well above the 150 reads per locus recommended by Liu et al. (2024), and assigned reads were generally consistent across samples within each sequencing run (Ti and Sc) (see Table S1 and Figures S6, S7). SatAnalyzer produced accurate genotypes and was effective on triaged scat samples. Comparisons of simplex CE data and *Canis* STR‐seq data provided informative comparative morphology (Data S3) to establish scoring criteria (Data S2). Most scores were consistent between the methods, but some issues were flagged in both methods. Errors with CE scores were associated with incorrect binning, over‐saturation, or high stutter not scored as an allele; all of these were checked and CE scores were corrected. The main issue with STR‐seq scores was the missed large‐sized alleles because of preferential amplification of small alleles. We noted that in three samples (CAN006169; CP2023‐036; and CP2023‐040), Cfam_STR032 has a 1 bp insertion at the end of the MRA region, resulting in allele_len of 82 and is flanked by allele_len 81 and 83 (Data S4).

For the tissue samples scored at 26 loci, allelematch correctly paired the duplicate genotypes to identify 84 unique individuals. Of those, 28 individuals had a score of 1 (0 mismatches), 26 had a score of 0.98 (1 mismatch), 26 had a score of 0.96 (2 mismatches), and 4 had a score of 0.94 (3 mismatches). The multi‐locus error rate across all tissue samples and all 26 loci was 2.1%, but was reduced to 0.8% when the loci with error rates ≥ 0.05 were excluded (Table 1). Most errors were due to a missed large allele call (38.2%) and missed allele calls where front stutter was 50% of the reads of the main allele (32.6%). The remaining errors were due to back stutter alleles not being scored when reads were at least 80% of the main allele (12.4%), missed small allele calls (14.6%), and calling alleles that were mixed or undecipherable and should have been scored as missing data (2.2%).

**TABLE 1:** Multilocus scoring errors for tissue (Ti) samples (*n* = 84) and scat (Sc) samples (*n* = 18).

Locus	Number of errors (Ti)	Error rate (Ti)	Number of errors (Sc)	Error rate (Sc)
Cfam_STR001	5	0.030	1	0.028
Cfam_STR002	0	0.000	0	0.000
Cfam_STR004	3	0.018	0	0.000
Cfam_STR007	1	0.006	0	0.000
Cfam_STR008	2	0.012	0	0.000
Cfam_STR009*	15	0.089	7	0.194
Cfam_STR010	0	0.000	1	0.028
Cfam_STR011	0	0.000	0	0.000
Cfam_STR012**	9	0.054	4	0.111
Cfam_STR013	1	0.006	0	0.000
Cfam_STR014	4	0.024	1	0.028
Cfam_STR015	0	0.000	1	0.028
Cfam_STR016	1	0.006	0	0.000
Cfam_STR017	1	0.006	0	0.000
Cfam_STR018	0	0.000	0	0.000
Cfam_STR019*	16	0.095	1	0.028
Cfam_STR020	2	0.012	0	0.000
Cfam_STR021	1	0.006	0	0.000
Cfam_STR022	1	0.006	0	0.000
Cfam_STR026	0	0.000	0	0.000
Cfam_STR027	0	0.000	1	0.028
Cfam_STR029	0	0.000	1	0.028
Cfam_STR030**	10	0.060	2	0.056
Cfam_STR031	4	0.024	0	0.000
Cfam_STR032	1	0.006	0	0.000
Cfam_STR033*	14	0.083	1	0.028
Total All Loci	91	0.021	21	0.022
Total (loci with error rate > 0.08 removed)	46	0.012	12	0.014
Total (loci with error rate > 0.05 removed)	27	0.008	6	0.008

*Note:* Error rates for assay calculated for each locus, for all loci across all samples, for loci with error rate < 0.08 (3 loci removed—marked with *), for loci with error rate < 0.05 (2 additional loci removed—marked with **).

For the scat samples, allelematch correctly matched paired scores for 16 individuals across 26 loci, with six having no mismatches (1.0), seven having one mismatch (0.98), two having 2 mismatches (0.96), and one having six mismatches (0.90—with two of these due to one locus with missing data for one of the paired genotypes at locus Cfam_STR012). Removing the 5 high error rate loci identified in the tissue data decreased the error rate for the scat samples from 2.2% to 0.8% (Table 1).

For the tissue comparisons, there was only one inconsistency between independent scores for sex assignment. This was due to one score being just over the threshold of 0.02 for Y/X ratio setting. When comparing with previous sex assignments from CE, the female assignment is correct (and was consistent with the field ID for this individual). All other sex assignments were consistent for CE and STR‐seq identification. For the scat sex comparisons from independent runs in SatAnalyzer, there were 2 mismatched assignments on the basis of scores from independent assessment, with one score showing as female and the other assigned as inconclusive. These inconsistencies were due to differences in rounding of the Y/X ratio with the two different scores (e.g., one had a ratio of 0.02 and called it a female, the other had a ratio of 0.0178 and called it inconclusive) and the other call had one run calling a female with a ratio of 0.01 and the other calling it inconclusive with a ratio of 0.008. For the scat samples, there were two samples called inconclusive that were females on the basis of CE and two that were called as males (Y/X ratios 0.0993 and 0.05) that were previously identified as females with CE, suggesting the Y/X parameter for sex identification should be increased to at least 0.1 in future runs. We also note that in a subsequent independent run of the assay, where library preparation was done by independent technicians at a separate lab, and sequencing was done on a different Illumina MiSeq sequencer, the positive control samples CAN004247 and CAN004248 had identical genotypes to those reported here, thereby further corroborating the assay across independent labs.

### 3.2 Size Homoplasy, Genetic Diversity and Population Structure

The analysis of the sequence data showed that sequence mutations and size homoplasy were common in the dataset. We identified repeated occurrences of mutations in the MRA, FF and RF regions, resulting in homoplasy for 18 of the 26 loci analyzed (Table 2). Overall genetic diversity was higher when allele calls included sequence mutations (allele_mut) compared to allele calls on the basis of length alone (allele_len). The total number of alleles increased 32% from 253 in the allele_len dataset to 334 in the allele_mut dataset and observed heterozygosity increased from 0.69 (SE ±0.03) to 0.71 (SE ±0.02).

**TABLE 2:** Homoplasy. Proportion of alleles with homoplasy detected in this study. Number of individuals = 84, number of loci = 26.

Locus name	Number of fragment size classes	Number of size classes with homoplasy	Proportion of size classes with homoplasy	Number of size classes that occur only as a mutated version
Cfam_STR001	12	0	0.00	0
Cfam_STR002	9	4	0.44	1
Cfam_STR004	12	1	0.08	0
Cfam_STR007	12	6	0.50	3
Cfam_STR008	11	0	0.00	0
Cfam_STR009	10	6	0.60	0
Cfam_STR010	10	2	0.20	0
Cfam_STR011	9	4	0.44	1
Cfam_STR012	7	5	0.71	1
Cfam_STR013	6	3	0.50	4
Cfam_STR014	5	3	0.60	3
Cfam_STR015	6	0	0.00	0
Cfam_STR016	7	1	0.14	0
Cfam_STR017	9	1	0.11	0
Cfam_STR018	9	1	0.11	0
Cfam_STR019	16	1	0.06	0
Cfam_STR020	11	0	0.00	0
Cfam_STR021	6	0	0.00	0
Cfam_STR022	12	7	0.58	7
Cfam_STR026	12	0	0.00	0
Cfam_STR027	8	0	0.00	0
Cfam_STR029	9	0	0.00	0
Cfam_STR030	10	5	0.50	0
Cfam_STR031	11	4	0.36	1
Cfam_STR032	12	1	0.08	0
Cfam_STR033	12	5	0.42	0

On the basis of various methods implemented in StructureSelector (Li and Liu 2018), the optimal number of clusters varied somewhat across methods (LnProbData, Δ*K*, (Evanno et al. 2005) Means & Medians (Puechmaille 2016)) and datasets (allele_len vs. allele_mut). On the basis of Δ*K*, all datasets resulted in the first broad division of Eurasian vs. North American ancestry at *K* = 2 (Figure S8); beyond that, for the datasets with dogs Δ*K* suggests optimal clusters at *K* = 4 for the allele_len dataset and *K* = 5 for the allele_mut dataset (Figure S8a,b). For LnProbData, the values plateau at *K* = 5 or *K* = 6 for both datasets. The Means and Medians approach suggests *K* = 6 for both datasets. In both datasets, an Eastern wolf cluster appears at *K* = 4 with continued resolution to separate Eastern coyotes from Western coyotes at *K* = 5. Great Lakes grey wolves continued to cluster with Western grey wolves at *K* = 6 and an unknown cluster appears (Figures S9, S10). We note, however, that at *K* = 6, the signature from this unknown cluster was only present in Eastern *Canis* populations and did not appear in dogs, Western gray wolves or Western coyotes (Data S6). For datasets excluding dogs, methods generally converge on *K* = 5 for both the allele_len and allele_mut datasets (Figure S8c,d) and *K* = 5 was identified for the allele_len scat dataset (Figure S8e).

**FIGURE 3:**
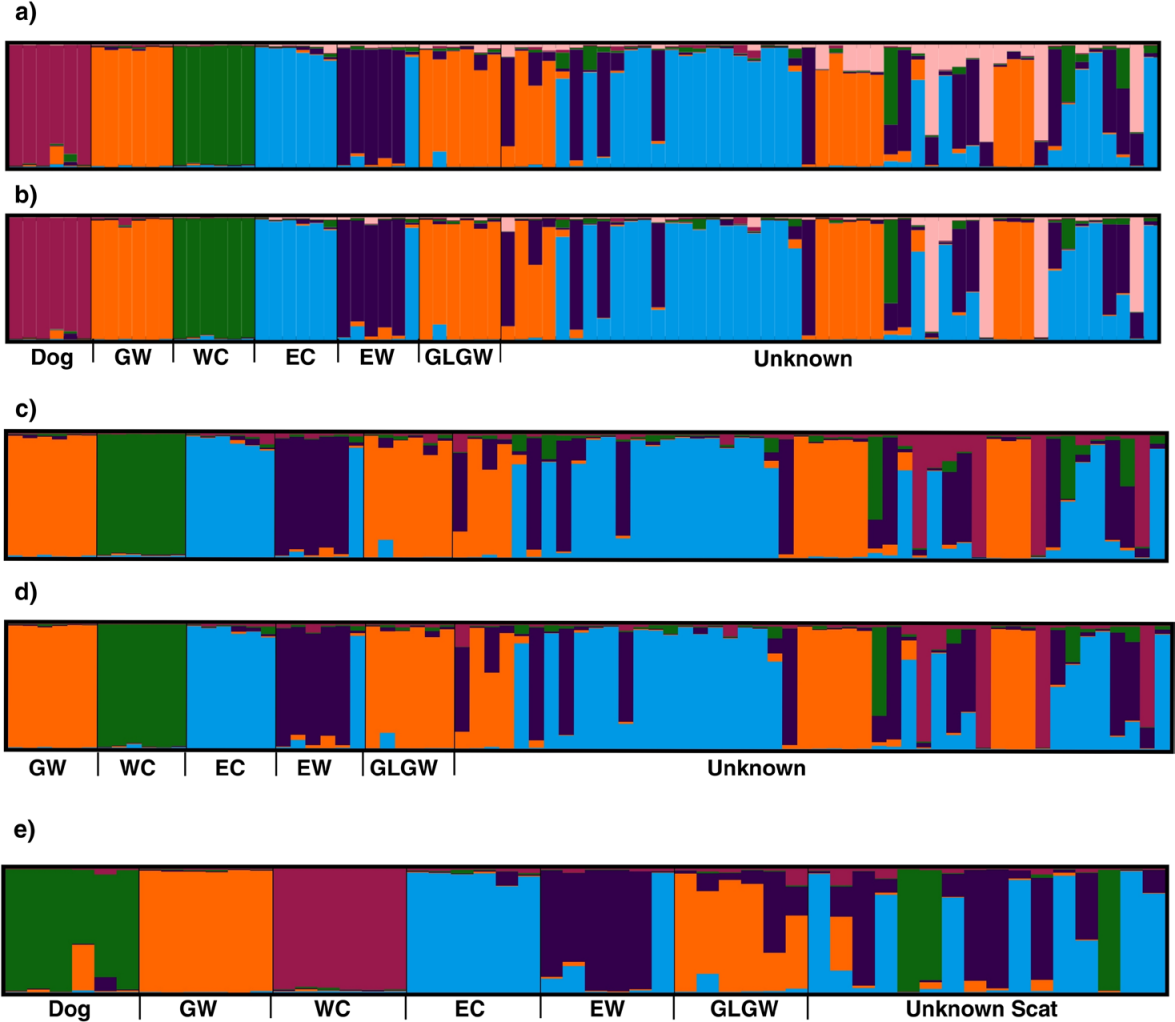
Structure Output. Output for *K* = 6 clusters from tissue sample analysis with structure, including dogs (a, b) and for *K* = 5 excluding dogs (c, d) on the basis of microsatellite length only (allele_len) (a, c) and microsatellite length plus mutation data (allele_mut) (b, d). Output at *K* = 5 for the allele_len dataset with the unknown scat samples is also shown (e). Assignments (*Q* values) are similar for tissue datasets, but the allele_mut data showed higher assignment to the predominant cluster for 64 of 84 individuals. Dog = domestic dog, GW = Western gray wolf, WC = Western coyote, EC = Eastern coyote, EW = Eastern wolf, GLGW = Great Lakes gray wolf, Unknown = samples of unknown ancestry for Ti (a–d) and Sc (e) samples.

Assignments were generally consistent between the two allele_len and allele_mut datasets (Figures 3a,b, S9, S10 and S6), but the allele_mut dataset resulted in higher assignment values to the main cluster in 64 samples, compared to 19 samples where the allele_len had a higher *Q* value to the main cluster and one sample had no change (Data S6). Overall, for the allele_len dataset the assignments for unknown samples (on the basis of *Q* values from Structure) were: gray wolf (*n* = 6), Eastern wolf (*n* = 4), Eastern coyote (*n* = 13), Admixed (*n* = 25), and the allele_mut dataset were: gray wolf (*n* = 10), Eastern wolf (*n* = 3), Eastern coyote (*n* = 17), Admixed (*n* = 15), Unknown Source (*n* = 3). Analysis of datasets that excluded dogs had similar outcomes to the analyses with dogs; both Western gray wolves and Great Lakes gray wolves form a single cluster and an unknown cluster is identified (Figures 2c,d and Data S6). We note that excluding dogs resulted in some previous Admixed assignments shifting to gray wolf (*n* = 5), Eastern wolf (*n* = 4), or Unknown Source (*n* = 5); results from the scat dataset identified unknown scat samples as Eastern coyotes (*n* = 4), Eastern wolves (*n* = 4), dogs (*n* = 3), and Admixed (*n* = 5) (Figure 3e and Data S6).

## 4 Discussion

We present a robust *Canis* STR‐seq assay that embraces the renewed interest in microsatellites by sequencing amplicons with Illumina high‐throughput sequencing technology. The assay avoids the pitfalls of traditional genotyping of microsatellites on the basis of size fragment analysis with CE and provides a cost‐effective way to sequence hundreds of samples together on a single sequencing run. Overall, the new approach produced highly repeatable genotypes with a universal workflow that alleviates the major pitfalls of microsatellite genotyping with capillary electrophoresis.

### 4.1 Sequencing, Genotyping and Errors

The average number of sequencing reads was well above 150 reads per locus, as recommended by Liu et al. (2024) (Table S1), and assigned reads per sample were generally consistent for both Ti and Sc samples (Figures S6, S7). Performance of the sequence‐based genotyping method produced highly accurate profiles at 27 loci for various *Canis* species from a variety of sample types (blood, tissue, hair, and scat) and provides a baseline dataset that includes sequence mutations to reflect true diversity. Although scoring error was high for three loci and moderate for two additional loci, the error rate can be alleviated by increasing familiarity with the data analysis workflow, consolidated independent scoring by at least two people, and following specific scoring criteria (Liu et al. 2024). Although some time is required to become familiar with SatAnalyzer and its functionality, we found that scoring with SatAnalyzer was highly accurate and provided an easy platform to visually inspect and manually correct allele calls from high‐throughput sequencing data. It has several benefits over traditional fragment analysis that requires proprietary software. First, it is open access, so it does not require purchase or subscriptions to software platforms, and it works effectively in both Windows and Linux environments, as noted in (Liu et al. 2024). Although we were unable to run SatAnalyzer on a new MacOS with the M1 chip technology, this does not preclude the use of Seq2Sat in the command line without the SatAnalyzer web‐based interface. Second, scoring is easier because: (a) the visual representation of the mutations makes it easier to distinguish alleles from stutter, (b) there are no fluorescent dyes so the primers are less expensive and there are no issues with pull‐up because of dye interaction, (c) it can detect alleles with very few reads (e.g., 10, although 150 minimum is recommended), (d) there is no binning of alleles and no need to shift or expand bins on the basis of different genotyping runs, and (e) it flags homoplasy to allow an accurate reflection of allelic diversity.

Error rates for STR‐seq data from tissue samples are consistent with previous multilocus estimates for CE panels with fewer loci used with bear tissue (Bonin et al. 2004) (18 markers: 0.8%) and wolf scat (Rutledge et al. 2017) (12 markers: 0.5%). Identification of confirmed duplicate samples in the tissue assay and consistent genotypes for two positive controls (CAN004247 and CAN004248) included in both tissue and scat sequencing runs validated the consistency of the assay to provide repeatable results, demonstrating the reliability of the process to track individuals. Inconsistencies in sex identification were very low and easily remedied by referencing field data and previous genetic sex identification to inform parameterization of the Y/X ratio; we note that one run was done with an older Linux version of SatAnalyzer and the other (with the inconclusives) was done with a recent Windows version, thereby flagging the importance of using the most up to date version of the software available, regardless of platform.

### 4.2 Size Homoplasy, Genetic Diversity and Population Structure

We report high homoplasy in STR markers traditionally used for wolf genotyping. This is consistent with other studies that report 44.7% – 63.5% of loci having mutations that result in multiple alleles with the same fragment length (Šarhanová et al. 2018), suggesting previous microsatellite studies have underestimated observed heterozygosity. Although the presence of homoplasy does not necessarily impact individual identification, our results demonstrated a marginal increase in heterozygosity when homoplasy was considered (increase of 0.02, overall).

Bayesian cluster analysis that included the mutations improved the power of cluster discrimination and resulted in more confident assignment to main clusters, probably because certain mutations are highly associated with certain *Canis* ancestral types. We suspect that for alleles where there is a significant mutational signature (e.g., in the flanking region), these alleles are linked to specific ancestry, although the appearance of an “unknown” cluster at *K* = 6 (including dogs) and at *K* = 5 (excluding dogs) could be (a) a ghost cluster that represents an unknown ancestral signature (Guillot et al. 2005), (b) a spurious cluster that may not represent a real biological grouping (Puechmaille 2016), or (c) representative of a regional family group since they seem to be from the same region in Ontario. Although we note some shifts in assignments among different datasets (Data S6), we recognize that the small sample size of the reference populations may be impacting our ability to resolve these variations. Specific *Q* values, therefore, should be interpreted with caution. Building a larger reference database will be an important next step for future broad‐scale monitoring. However, the discovery of novel allele sequences at some STR markers across multiple individuals has the potential to reveal species‐ or population‐specific private alleles, which could be useful for assessment of hybridization and admixture in future studies. We note that certain allele_mut scores appear to be localized in samples from eastern regions and absent from western regions, suggesting a possible eastern wolf ancestry; a more thorough analysis with a larger dataset that includes a larger sample size for the known reference groups and from a broader geographical range will help clarify the ancestry of specific alleles and resolve the cluster anomaly. The assignment of scat samples to Eastern coyote, Eastern wolf, and dog clusters supports the use of the assay for noninvasive monitoring of at‐risk Eastern wolves across their range.

## 5 Conclusions

There has been a trend toward SNP genotyping in population genetics, with some suggesting SNPs have many advantages over microsatellites in population genetics (Fitak et al. 2015; Eriksson et al. 2020; Hayward et al. 2022; Hervey et al. 2025). The advantages, however, are often related to challenges with traditional capillary electrophoresis approaches to microsatellite genotyping and don't necessarily apply to sequenced microsatellite data. In one study that compared three sets of sequence data (SNPs, microsatellites, nonrepetitive nuclear loci), there was no difference in outcomes at broad spatial scales; and although the SNP data were more effective at detecting genetic structure at fine spatial scales, the number of loci and alleles impacted the resolution for all marker sets (D'Aloia et al. 2020). These results suggest that incorporation of mutations to the microsatellite sequence data could provide additional fine‐scale resolution for detecting genetic structure. Furthermore, microsatellites are still informative for individual identification, noninvasive monitoring, inference of population structure (Haasl and Payseur 2011; Timm 2020) and other population processes (Hauser et al. 2021). In fact, many of the advantages of SNPs dissipate when comparing GBS SNP assays to GBS STR (microsatellites) assays, making microsatellites the preferred marker in some cases (Morin et al. 2009; Haasl and Payseur 2011; Hauser et al. 2021). Further, sequencing of microsatellites provides SNP data alongside size fragment length data, providing an opportunity to more fully explore the complexity of mutation models responsible for microsatellite evolution. The *Canis* STR‐seq assay represents the first step toward implementing a universal approach to genetic monitoring of wolf populations (including non‐invasive approaches) and provides a foundation for future research by providing a baseline reference dataset that can be expanded by researchers adopting this assay (de Groot et al. 2016). We suggest, as others have done previously, that microsatellites are still effective, affordable, and sometimes preferable markers for use in population genetics studies (Hodel et al. 2016; Timm 2020; Hauser et al. 2021) and that GBS technology has enabled a microsatellite renaissance. The focus on a universal approach to provide open access reference datasets provides a long‐awaited consolidation of genotyping methodology that promotes a cooperative framework for global wolf conservation.

## Author Contributions

**Emily Walker:** formal analysis (supporting), investigation (equal), methodology (equal), project administration (supporting), validation (equal), writing – original draft (supporting). **Brent R. Patterson:** funding acquisition (supporting), project administration (supporting), resources (supporting), writing – review and editing (supporting). **Glen A. Rutledge:** methodology (supporting), software (lead), writing – review and editing (supporting). **Linda Y. Rutledge:** conceptualization (lead), data curation (lead), formal analysis (lead), funding acquisition (lead), investigation (lead), methodology (equal), project administration (lead), software (supporting), supervision (lead), validation (equal), visualization (lead), writing – original draft (lead), writing – review and editing (lead).

## Funding

Funding for this project was provided by Canadian Nuclear Labs acting as an administrative agent for CANDU Owners Group Inc.

## Ethics Statement

Tissue and blood samples used during this study were either collected from licensed hunters or trappers or from animals live‐trapped, radio‐collared, and released as part of other research projects. All capture and handling methods were approved by the Ontario Ministry of Natural Resources Animal Care Committee (Protocol nos. 75‐05 to 75‐20) and the Trent University Animal Care Committee (Protocol nos. 08039, 24318).

## Conflicts of Interest

The authors declare no conflicts of interest.

## Supporting information


**Data S1:** ece373300‐sup‐0001‐DataS1.xlsx.

**Data S2:** ece373300‐sup‐0002‐DataS2.docx.

**Data S3:** ece373300‐sup‐0003‐DataS3.pdf.

**Data S4:** ece373300‐sup‐0004‐DataS4.xlsx.

**Data S5:** ece373300‐sup‐0005‐DataS5.xlsx.

**Data S6:** ece373300‐sup‐0006‐DataS6.xlsx.

**Data S7:** ece373300‐sup‐0007‐DataS7.xlsx.

## Data Availability

All data and Supporting Information are available through the Dryad repository (https://doi.org/10.5061/dryad.02v6wwqgb). The allele_muts.py code is available on the GitHub repository (DOI: https://doi.org/10.5281/zenodo.15794713).
