# Supplementary material for: Canis STR‐Seq: A Universal Approach for Non‐Invasive Genetic Monitoring of Wolves and Coyotes
Source: Ecol Evol. 2026 Mar 16;16(3):e73300. doi: 10.1002/ece3.73300 (PMC13093683; doi:10.1002/ece3.73300)
Supplement: Supplementary file 2 — Data S2: ece373300‐sup‐0002‐DataS2.docx. [file ECE3-16-e73300-s005.docx]

**Supplemental Methods and Results**

***DNA Extraction***

New tissue, blood, and hair samples were extracted using the EZNA DNA Blood and Tissue Kit (OMEGA) according to manufacturer’s protocols. Samples were eluted and stored in 100 𝛍L of elution buffer (OMEGA). Blood in snow samples were thawed in 50 mL tubes and spun down in a centrifuge at 3100 RMP for 25 minutes to collect a pellet. The supernatant was poured off and the pellet was resuspended in 250 𝛍L of 1X lysis buffer and extracted using a DNAeasy Tissue and Blood extraction kit (Qiagen) according to manufacturer’s protocols. DNA was eluted in 100 𝛍L of AE Buffer (Qiagen).

DNA was extracted from scat samples according to a previously established protocol.^1^ Briefly, we thawed frozen scat and swabbed the outside of the scat with a cotton swab and placed the swab immediately into 500 𝛍L 1x lysis buffer followed by extraction with a DNeasy Blood and Tissue Kit (Qiagen) according to manufacturer’s protocols. DNA was eluted in 100 𝛍L of AE buffer (Qiagen) and then concentrated using an Eppendorf vacufuge for 40 minutes to 1 hour until samples were condensed to approximately 50 – 60 µL in volume. For all extractions, an extraction negative was included to track any possible contamination of reagents and to ensure integrity of results.

***Triaging Scat Samples (mtDNA and nDNA amplification)***

To ensure the mtDNA amplification could distinguish *Canis* species from red fox and prey items, we tested mtDNA amplification with *Canis* mtDNA control region primers ABI13279^2^  (5’ – GAAGCTCTTGCTCCACCATC – 3’) and AB13280^3^  (5’ – GGGCCCGGAGCGAGAAGAGGGAC – 3’) on the following species: red fox (*Vuples vuples*)*,* moose (*Alces alces*)*, caribou (Rangifer tarandus*), elk (*Cervus canadensis*) white tail deer (*Odocoileus virginianus*) and beaver (*Castor canadensis*) with the following conditions. Each mtDNA PCR used 1x PCR Buffer (Promega), 1.5 mM MgCl_2_ (Promega), 0.2 mM dNTPs (Invitrogen), 0.2 𝛍g/𝛍L bovine serum albumin (BSA) (Thermofisher), 0.2 𝛍M of each primer, 0.05U/𝛍L of *Taq* DNA polymerase (Promega) and 4 𝛍L of DNA template in a final reaction volume of 15 𝛍L. The PCR cycling conditions were as follows; 94℃ for 5 minutes, 30 cycles of 94 ℃ for 30 seconds, 60 ℃ for 30 seconds and 72 ℃ for 20 seconds, with a final extension of 72℃ for 2 minutes. We noted that the primers did not effectively amplify red fox, beaver, elk, or caribou. The primers did, however, amplify moose and white-tailed deer but at a larger fragment size so the amplification can still be used to distinguish *Canis* from these two main prey items based on fragment size (Figure S1).


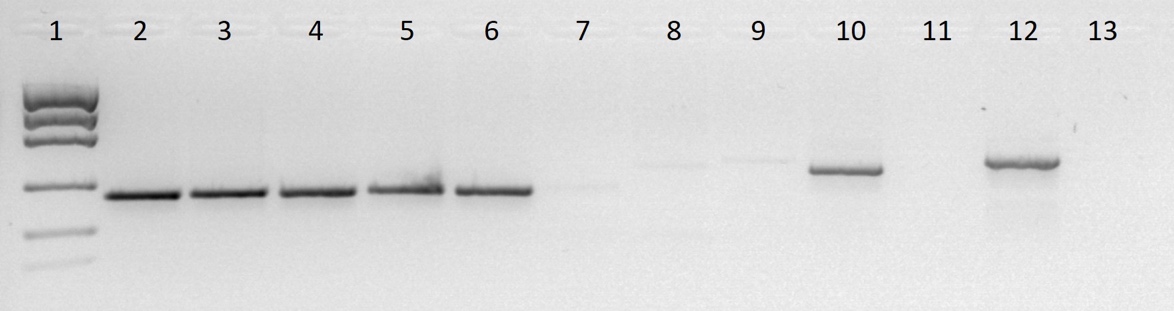


**Figure S1. Testing mtDNA Amplification.** *Canis* mtDNA primers were used to test amplification on non-target species. Lane 1: 6 𝛍L of Low Mass DNA Ladder (Invitrogen, Cat No, 10068013); Lane 2 – 5: *Canis* positive control DNA 2.5ng/𝛍L, 1ng/𝛍L, 500 pg/𝛍L, and 250 pg/𝛍L respectively; Lane 6: coyote; Lane 7: red fox; Lane 8: beaver; Lane 9: elk; Lane 10: moose; Lane 11: caribou; Lane 12: white-tailed deer; and Lane 13: No Template PCR negative control.

To ensure scat samples were from *Canis spp.,* we amplified scat DNA with *Canis* primers targeting the control region of mitochondrial DNA (mtDNA). Both stock DNA and a 1 in 20 dilution were amplified with primers ABI13279 and AB13280 as described above. Amplicons were visualized with gel electrophoresis on a 2% agarose gel containing Gel Red (Millipore Sigma, Cat. No. SCT 123) staining agent to assess amplification success and determine presence or absence of inhibitors present in the DNA. If there was no band present after amplification using stock DNA but there was a band present using diluted DNA, this indicated inhibitors were present in the scat DNA and the diluted sample was used moving forward (Figure S2). Samples that did not amplify were excluded from further analysis. (We note that presence of inhibitors could also be addressed by cleaning DNA extracts through a commercial clean-up kit to potentially avoid testing dilutions, although we did not test that here).


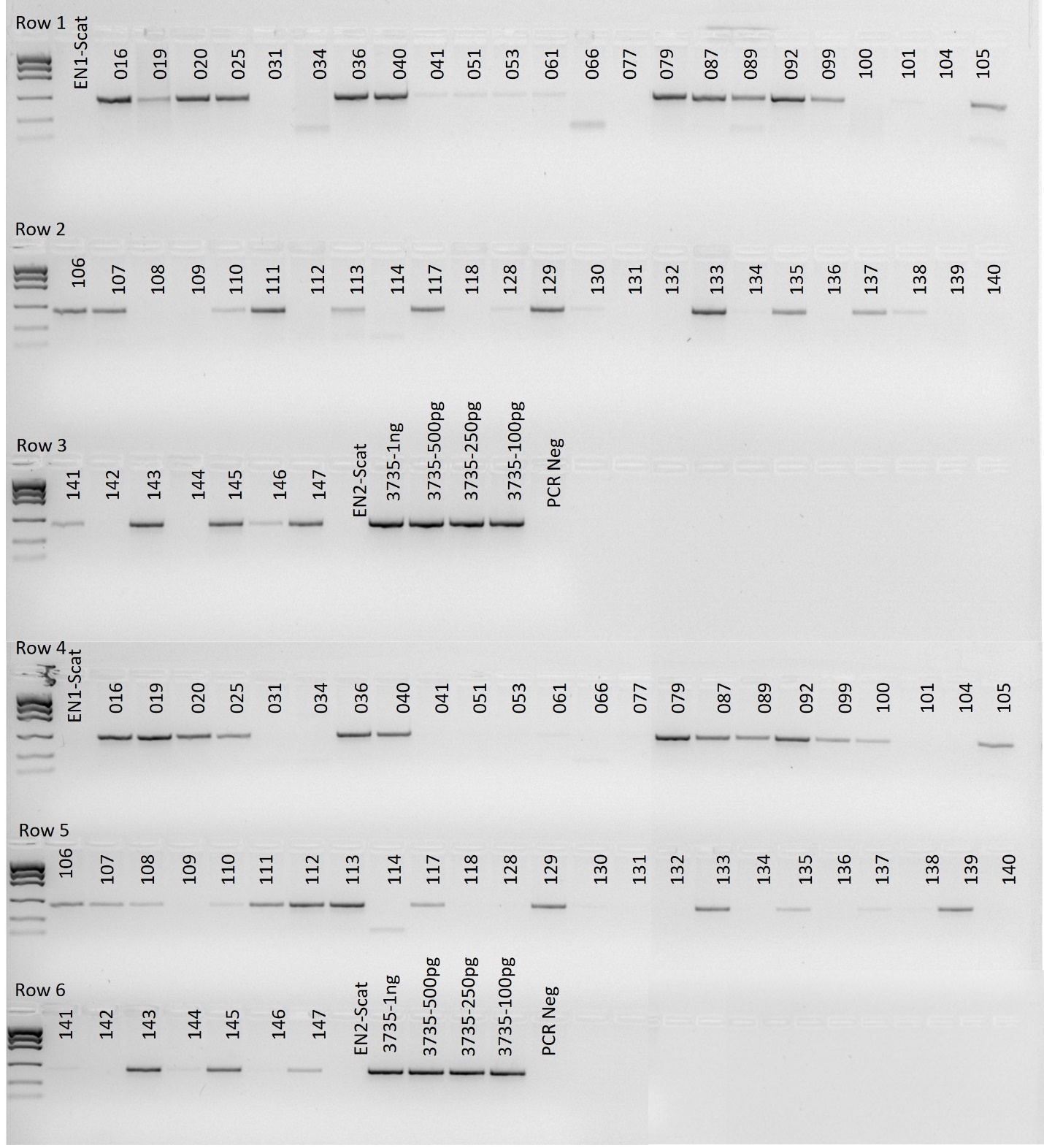


**Figure S2. mtDNA Triage.** DNA extracted from scat samples were amplified using both stock DNA (Rows 1-3) and diluted DNA (Rows 4-6) with *Canis* mtDNA primers ABI12379 and ABI13280. If a sample did not amplify using stock DNA (example Row 2, sample 112) but did amplify using diluted DNA (example Row 5, sample 112) it was determined that inhibitors were present and the diluted DNA sample was used for the *Canis* STR-seq assay.

Samples that passed the mtDNA test were tested for presence of *Canis* nuclear DNA. We amplified scat samples at the Cfam_STR015 (i.e. cxx204) nuclear microsatellite marker^4^ with primers: cxx204-F: 5’-CGAGAGCAACATAGGCATGA-3’ and cxx204-R: 5’-CAAAGTGCTGTGGCAGGTC-3’. Each PCR included 1x PCR Buffer (200mM Tris-HCL, pH 8.4, 500mM KCl), 1.5 mM MgCl_2_, 0.2 mM dNTPs, 0.2 𝛍g/𝛍L BSA, 0.3 𝛍M of each primer, 0.05U/𝛍L *Taq* DNA polymerase and 4𝛍L of DNA template in a final volume of 12𝛍L. The PCR conditions were as follows; 94℃ for 5 minutes, 30 cycles of 94℃ for 30 seconds, 56℃ for 1 minute, 72℃ for 1 minute, and a final extension at 60℃ for 45 minutes. (Note that this extension time is left over from legacy workflows to ensure the addition of an adenosine (A) base (+A) to the end of the fragment for more consistent scoring and is not necessarily required for this assay to perform properly). Amplicons were visualized with gel electrophoresis on a 2% agarose gel containing Gel Red (MilliporeSigma, Cat. No. SCT 123) staining agent to assess amplification success and determine presence or absence of *Canis* nuclear DNA. Target DNA was quantified by comparing the amplification success and intensity of the unknown samples to the amplification success and intensity of known quantity positive tissue control DNA in a serial dilution of 1 ng/𝛍l, 500 pg/𝛍L, and 250 pg/𝛍L (Figure S3).


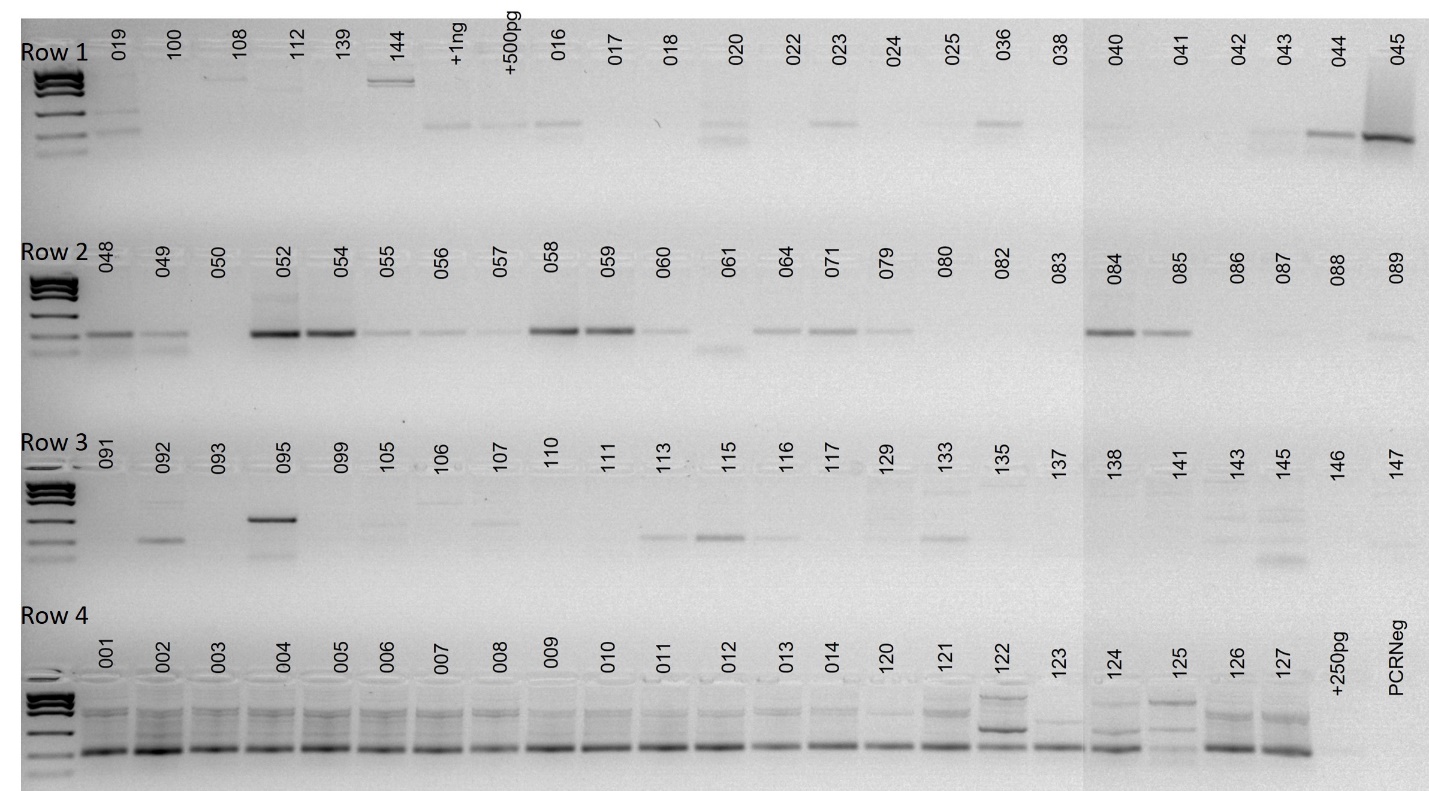


**Figure S3. Estimating target nuclear DNA.** DNA extracted from scat was amplified with the *Canis* DNA marker Cfam_STR015 to test for the presence of *Canis* nuclear DNA. To quantify the target nuclear DNA, amplification success was compared to *Canis* positive control samples at 1 ng/𝛍l, 500 pg/𝛍L (Row 1) and 250 pg/𝛍L (Row 4).

*In silico* tests suggested that Cfam_STR015 (cxx204) also amplifies red fox (See Supplemental Table Primers). To identify a *Canis* specific nuclear primer that could be used as a single test of species, inhibitors, and target we amplified scat samples at the Cfam_STR008, Cfam_STR012 and Cfam_STR029 nuclear microsatellite markers to test amplification on non-target species. These three markers were selected based on *in silico* primer blast tests (Supplemental Table Primers). Each PCR included 6 𝛍L of Qiagen Multiplex PCR Mastermix (Qiagen, Cat No. 206145), 1.2 𝛍L of 2x primer mix 2.8 𝛍L of RNAase free water and 2𝛍L of DNA template standardized to 1.25 ng/𝛍L in a final volume of 12 𝛍L. The PCR conditions were as follows; 94℃ for 5 minutes, 30 cycles of 94℃ for 30 seconds, 56℃ for 1 minute, 72℃ for 1 minute, and a final extension at 60℃ for 45 minutes. Amplification was visualized on a 1.5% agarose gel containing Gel Red (MilliporeSigma, Cat. No. SCT 123) (Figure S4). From this test it was determined that Cfam_STR008 and Cfam_STR012 did not amplify non-target nuclear DNA and could be used as a single amplification test for triaging scat samples (species ID, inhibitor test, quantify target DNA). However, marker Cfam_STR029 amplifies red fox (Lane C) at the same fragment size as *Canis* species and should not be used for *Canis* specific amplification tests in the absence of the initial *Canis* mtDNA triage step (described above).


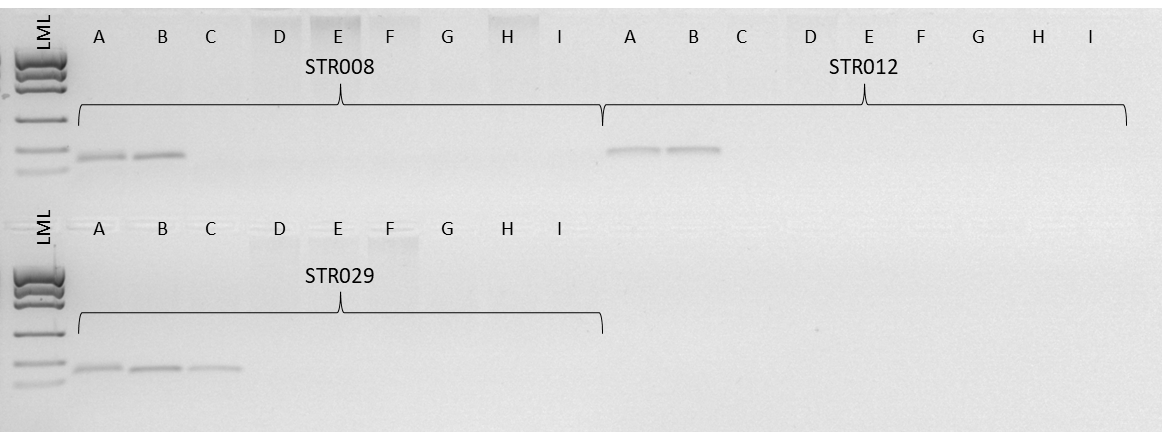
**Figure S4. Testing *Canis* STR primers for specificity.** Cfam_STR008, Cfam_STR012 and Cfam_STR029 were screened against non-target species test specificity to *Canis* species. Lane 1 in both Row 1 and Row 2: 6 𝛍L of Low Mass DNA Ladder (Invitrogen, Cat No, 10068013); Lanes A-I for all three markers are as follows: A- 1.25ng/𝛍L of *Canis* positive control, B- 1.25 ng/𝛍L of coyote, C- 1.25 ng/𝛍L red fox, D- 1.25 ng/𝛍L beaver*,* E- 1.25 ng/𝛍L white-tailed deer*,* F- 1.25 ng/𝛍L moose, G- 1.25 ng/𝛍L caribou, H- 1.25 ng/𝛍L elk and I- No Template PCR negative control.

***Capillary Electrophoresis vs SatAnalyzer***

To help develop scoring criteria for the new genotype-by-sequencing (GBS) *Canis* STR-seq assay, we ran simplex amplification reactions with 6FAM labelled forward primers at each locus on 15 tissue samples and scored them with a traditional capillary electrophoresis (CE) approach to compare genotypes and peak morphology of the CE simplexes with the GBS output in SatAnalyzer. Each simplex included 1x primer mix of 6FAM labelled forward primer and reverse primer for each STR, 1x Qiagen Multiplex PCR Mastermix (Qiagen, Toronto ON. Cat No. 206143), 4 𝛍L of DNA (standardized to 1.25 ng/𝛍L) in a final volume of 12 𝛍L. The PCR cycling conditions were as follows; 95℃ for 5 minutes, 30 cycles of 94℃ for 30 seconds, 60℃ for 90 seconds and 72℃ for 60 seconds with a final extension of 60℃ for 45 minutes. This PCR can also be run with a shorter final extension of 72℃ for 2 mins with the same result. Amplicons were visualized on an ABI 3730 (Applied Biosystems) by combining 1 𝛍L of PCR product with 9 𝛍L of HiDi-Formamide and GenScan 500 ROX size standard (Applied Biosystems) mixture. Samples were genotyped with standardized bins in Genemarker v 7.1 (SoftGenetics).

***Scoring Criteria in SatAnalyzer***

In SatAnalyzer alleles were defined based on number of reads, length of the sequences (allele_len), and the occurrence of SNPs within each length class, forward and/or reverse flanking regions (allele_mut). Loci comprising of one length fragment with a frequency of equal to or >80% of all reads were scored homozygous for the allele characterized by its respective length. Stutter peaks were identified as less than 50% of the true allele in number of reads. Genotypes were scored heterozygous if i) the difference in length of the potential alleles is greater than one repeat motif length (Figure S5a-b), ii) the two alleles differ by only one repeat and the allele of lower frequency (less reads) is larger (bp) than the allele with higher frequency (Figure S5c), iii) the larger allele is not lower in frequency than the shorter allele and the shorter allele must have a frequency of 80% or greater in reads than the larger allele (Figure S5d) iv) the larger allele has stutter peaks of equal height, score the largest band as the true allele (Figure S5e) v) the alleles are of the same length but one has a mutation present (Figure S5f) and vi) the larger allele has less than 50% frequency but is still scored as true second allele (Figure S5g).


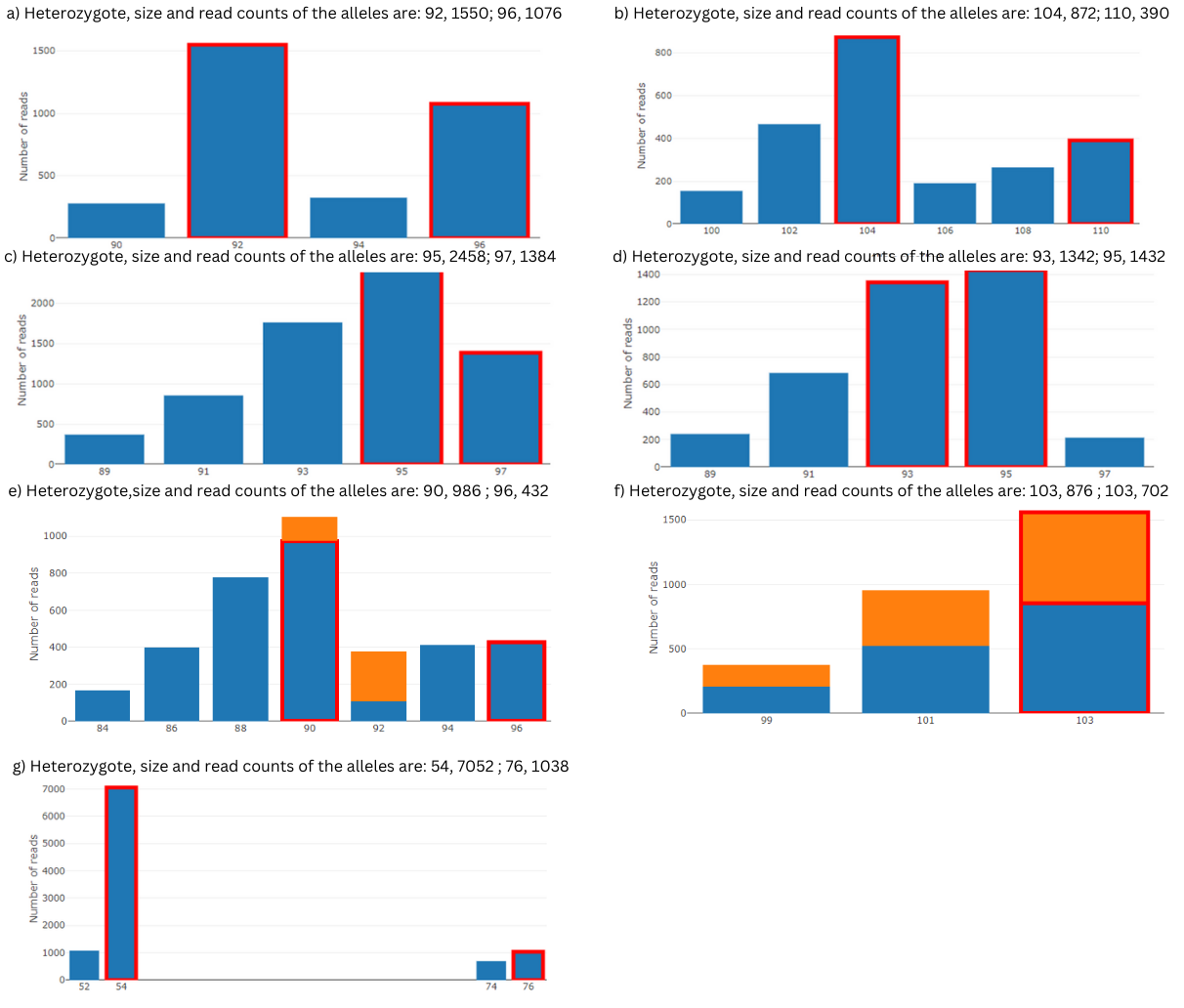


**Figure S5.** Scoring Heterozygotes with SatAnalyzer^5^ . Number of reads per amplicon length. Panel a) shows unambiguous heterozygote genotype. Panels b- g, shows examples matching the three cases of stutter control and interpretation. b) two alleles with a length difference above the repetition motif length; c) two alleles with length difference equal to the motif length, whose frequency of the shorter is higher than the longer one; d) two alleles with length difference equal to the motif length, whose frequency of the shortest allele is more than 80% of the longer one; e) larger allele has stutter peaks of equal height; f) alleles are the same length but one has a mutation present and g) larger allele is significantly lower in frequency than the smaller allele. Bars bordered with red correspond to amplicon lengths chosen as alleles by the genotyping method. The x axis corresponds to allele length and y axis corresponds to the number of reads supporting it.

***Sequencing Reads, Hardy-Weinberg Equilibrium (HWE), Linkage Disequilibrium (LD), Structure & Principal Components Analysis (PCA)***

We calculated the reads per allele per locus for both tissue (Ti) and scat (Sc) sequencing runs based on the allele call (Table S1). In all cases the average and median number of sequencing reads was well above the recommended 150 reads per locus.^5^ Output from SatAnalyzer showed two samples skewed the upper range of the Ti samples (Figure S6), suggesting those samples had excess of DNA template in PCR1. Assigned reads across the Sc samples was generally consistent (Figure S7), suggesting the triage approach to sample selection helped to standardize template DNA input in PCR1.

Previous analysis with a larger dataset indicates that 12 (Cfam_STR006, Cfam_STR011, Cfam_STR012, Cfam_STR013, Cfam_STR014, Cfam_STR015, Cfam_STR016, Cfam_STR017, Cfam_STR018, Cfam_STR019, Cfam_STR020, Cfam_STR021) of the original 33 loci considered are in Hardy-Weinberg Equilibrium.^6^  Despite the very low sample sizes (n=6 for each population of Dogs, Western Grey Wolves, Western Coyotes, Eastern Coyotes, Eastern Wolves, Great Lakes Wolves), Chi-squared tests of HWE conducted in GenAlEx 6.503^7^ on allele_len genotypes at loci used in the STRUCTURE analysis showed no significant deviation from HWE in all but six cases: Dogs (Cfam_STR013, p<0.01; Cfam_STR014, Monomorphic), Western Coyotes (Cfam_STR002, p<0.05), Eastern Coyotes (Cfam_STR012, p<0.05; Cfam_STR015, Monomorphic), Great Lakes Wolves (Cfam_STR009, p<0.05). The deviations observed were variable across populations and loci and should be interpreted with caution given the small sample sizes. However, the limited deviations and variability, combined with previous analysis on several of the loci used, suggest that there is no specific loci of concern. Assessment with larger sample sizes will improve confidence in HWE across populations. We also conducted tests of linkage disequilibrium in Genepop v.4^8^ with dememorization = 10000, batches = 100, iterations = 5000 and found no evidence of LD between any of the loci used in Structure analysis. Although, again, additional tests with a larger dataset will provide further insight into LD.

Overall, six clusters were identified as the optimal number of clusters for allele_len and allele_mut datasets (Figures S8a,b) and five clusters were identified when Dogs were excluded (Figures S8c,d). Output from Structure^9^  was generally consistent between the allele_len and the allele_mut datasets, with similar clustering patterns at K=2 to K=8 (Figures S9 and S10).

**
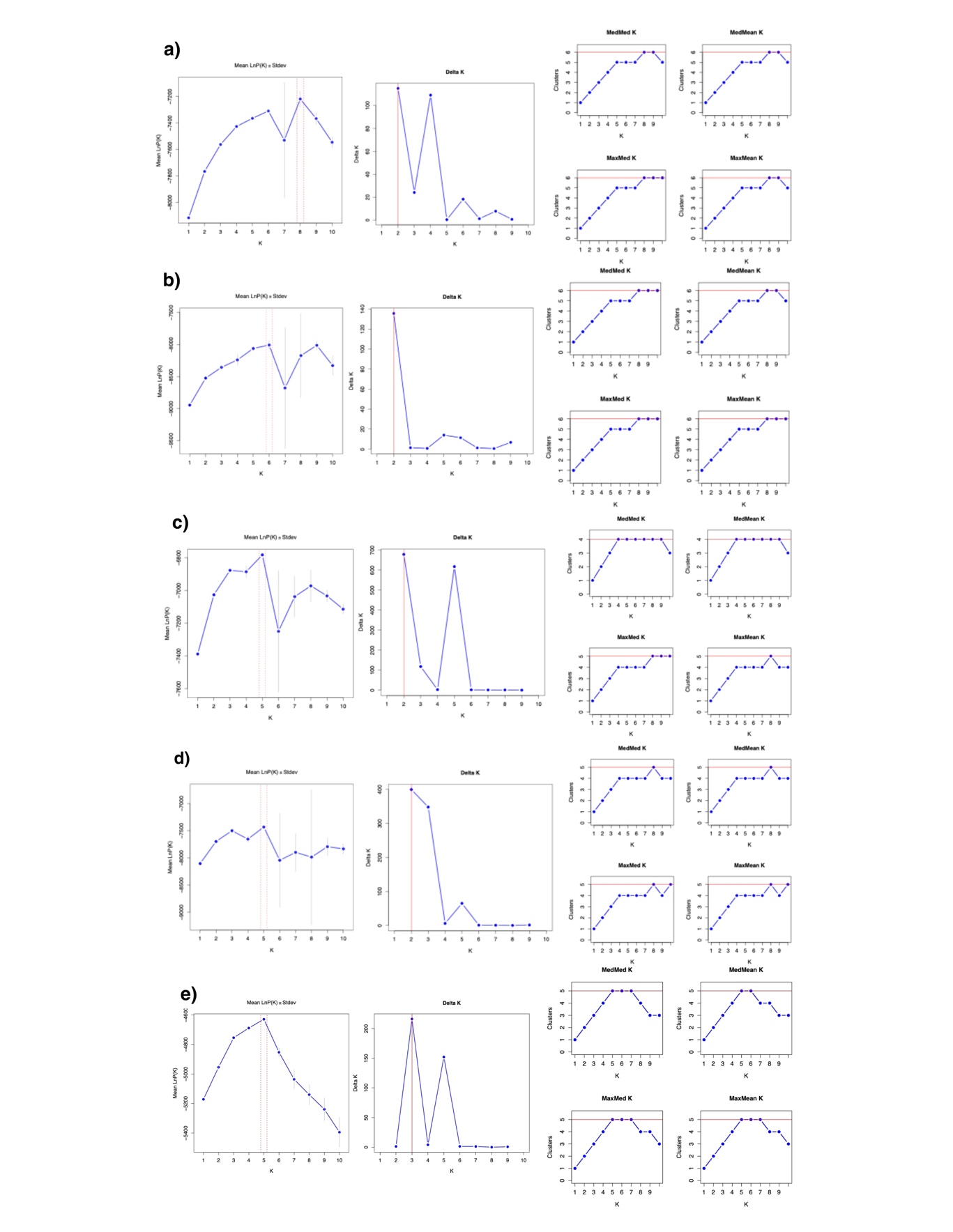
**

**Figure S8.** Optimal Clusters. Optimal clusters (including Ln Prob data^10^ , delta K,^11^ and median K^12^) from datasets including Dogs a) allele_lenWithDogs, b) allele_mutWithDogs, and excluding Dogs c) allele_lenNoDogs, d) allele_mutNoDogs. Panel e) shows output for the allele_len dataset that included the scat samples.


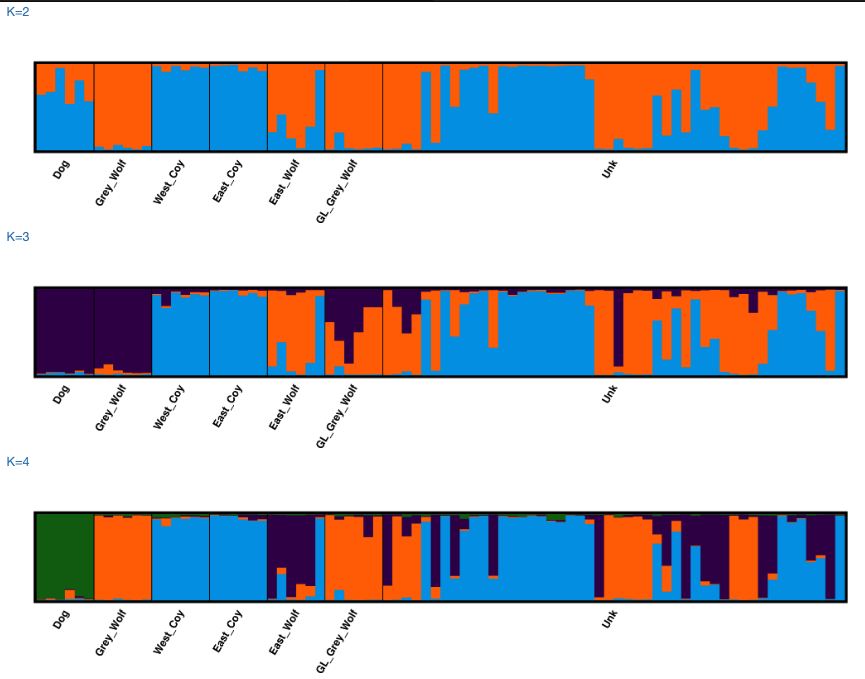


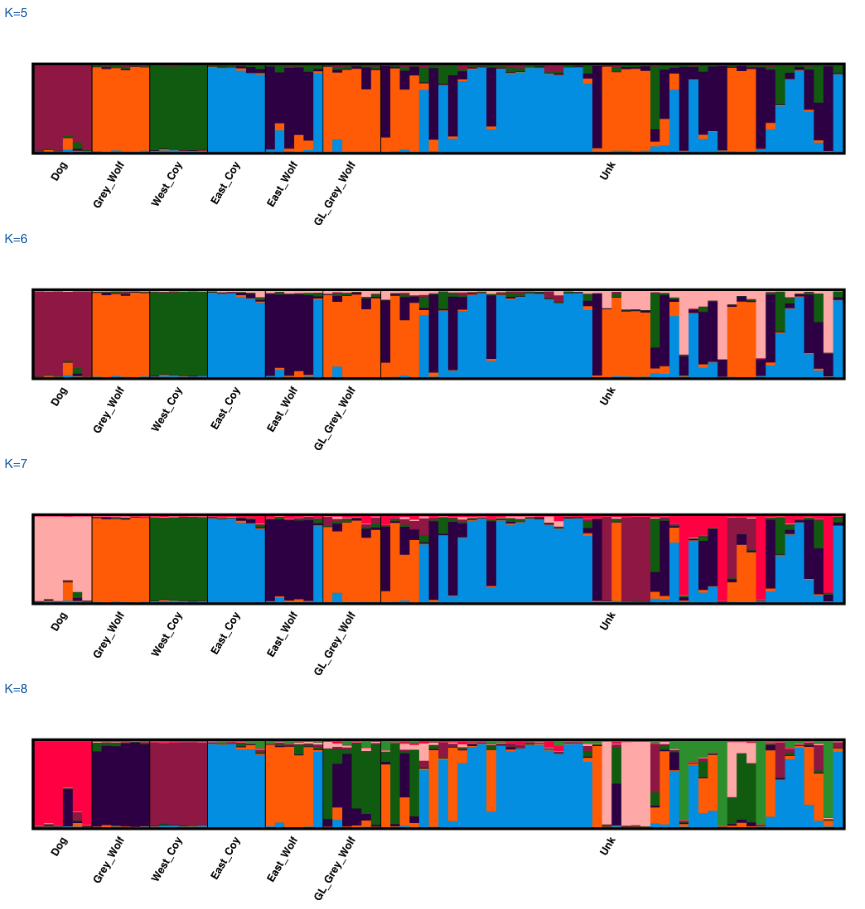


Figure S9. Output from Structure (K=2 – K=8) for the allele_lenWithDogs dataset.


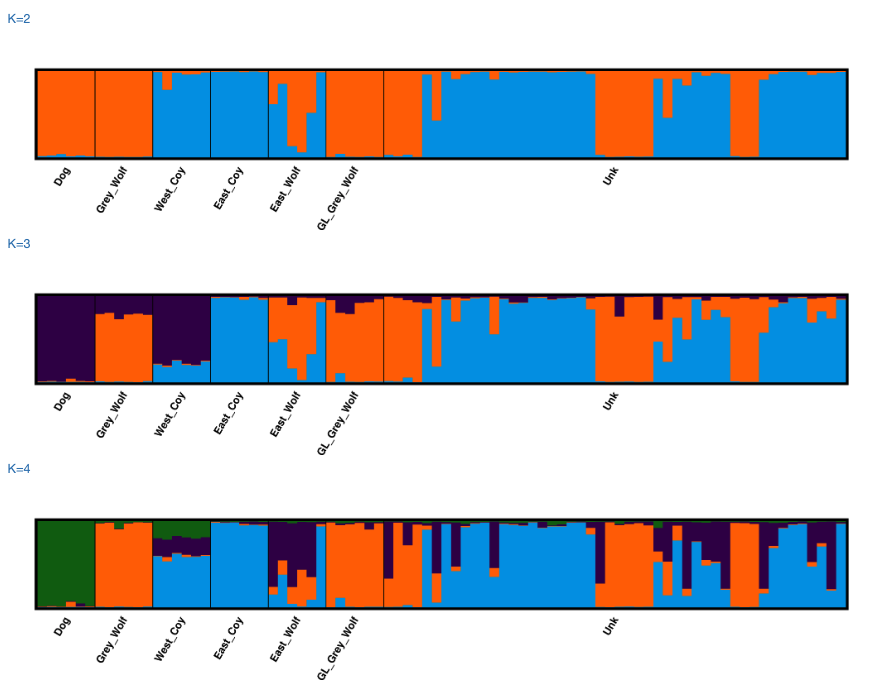

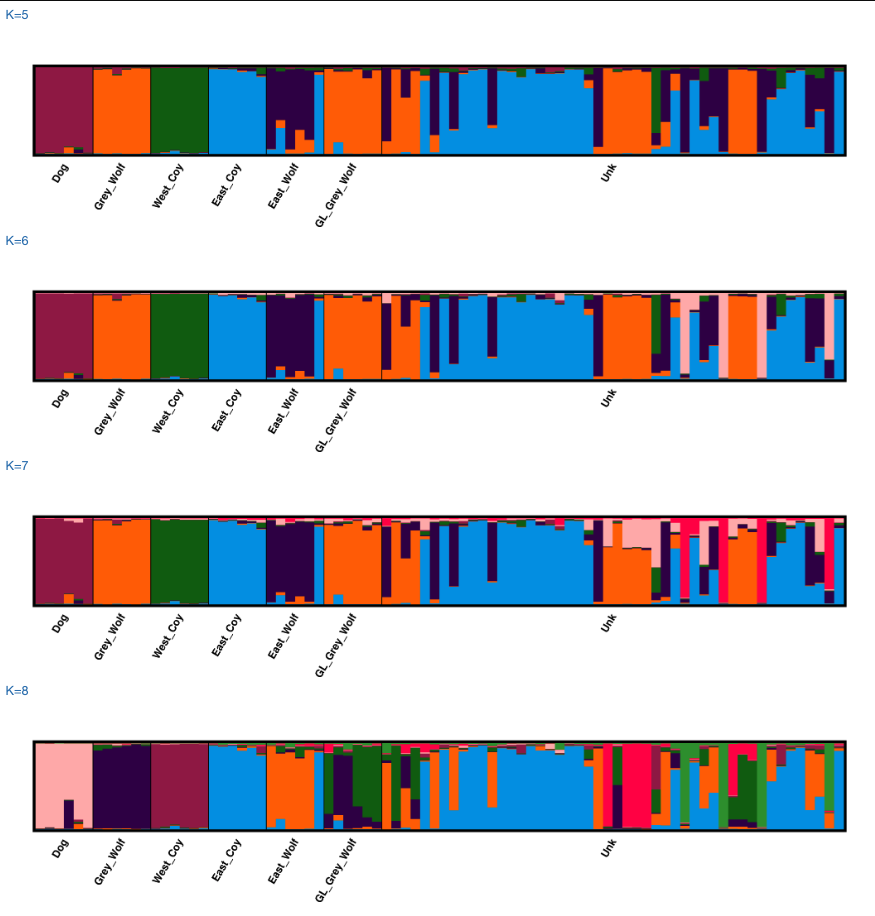


**Figure S10.** Output from Structure (K=2 – K=8) for the allele_mutWithDogs dataset.

We used the adegenet package^13^ in R to conduct principal components analysis (PCA) on the allele_len and allele_mut tissue (Ti) datasets with, and without, the Dog reference group, and also for the scat (Sc) dataset with Dogs (Figure S11). As expected, we note a close relationship between Western Grey Wolves (GW) and Great Lakes Grey Wolves (GLGW) and between Western Coyotes (WC) and Eastern Coyotes (EC) in all versions of the PCA. Although there are few notable differences among the PCAs, there is some tighter clustering of Eastern Coyotes in the allele_mut PCA without the Dog data compared to the allele_len data (Figures S11c; S11d). We also note that dogs (n=3) and Eastern wolves (n=4) are clearly identified in the scat PCA (Figure S11e), which is consistent with the Structure results (Figure 2e in the main text) and supports the use of the assay for noninvasive monitoring of *Canis* species. Again, given the small sample sizes of the reference populations these results should be interpreted with caution.

**
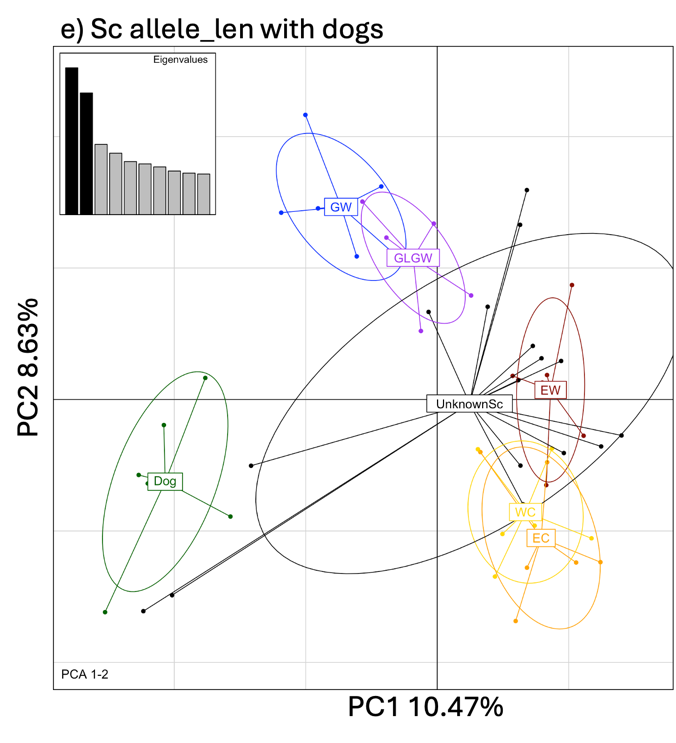
**
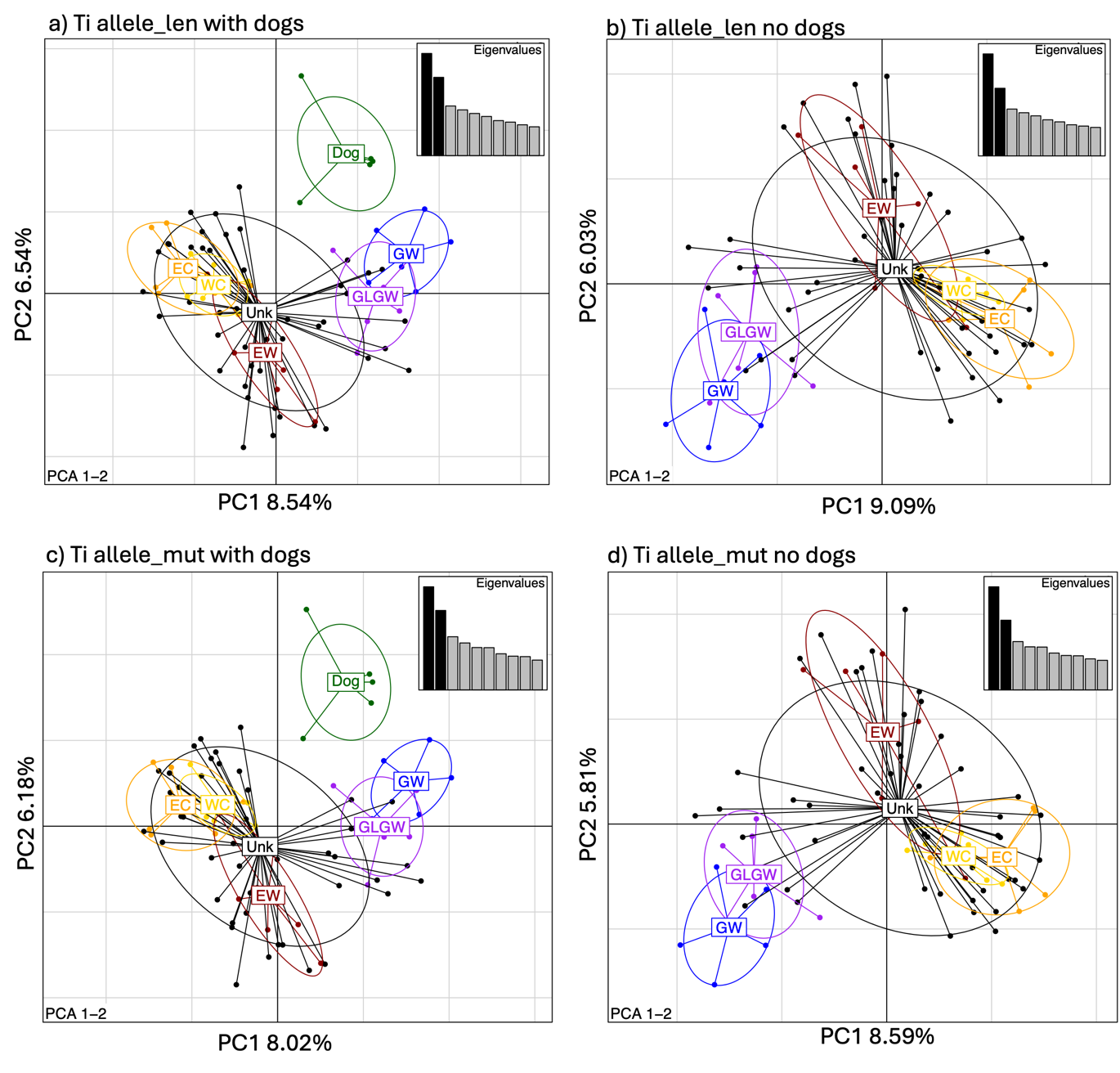


**Figure S11.** Principal components analysis based on allele_len (a, b), allele_mut (c, d) tissue (Ti) datasets, and on the allele_len scat (Sc) dataset. Dog=dogs; GW=Western Grey Wolves, GLGW=Great Lakes Grey Wolves; EW=Eastern Wolves; WC=Western Coyotes; EC=Eastern Coyotes; Unk=Unknown Individuals (Tissue); UnknownSc=Unknown Individuals (Scat)

**Supplemental References**

1. Rutledge, L. Y., Holloway, J. J., Patterson, B. R. & White, B. N. An improved field method to obtain DNA for individual identification from wolf scat. *J. Wildl. Manag.* **73**, 1430–1435 (2009).

2. Pilgrim, K. L., Boyd, D. K. & Forbes, S. H. Testing for Wolf-Coyote Hybridization in the Rocky Mountains Using Mitochondrial DNA. *J. Wildl. Manag.* **62**, 683 (1998).

3. Wilson, P. J. *et al.* DNA profiles of the eastern Canadian wolf and the red wolf provide evidence for a common evolutionary history independent of the gray wolf. *Can J Zool* **78**, 2156–2166 (2000).

4. Ostrander, E. A., Sprague, G. F. & Rine, J. Identification and Characterization of Dinucleotide Repeat (CA)n Markers for Genetic Mapping in Dog. *Genomics* **16**, 207–213 (1993).

5. Liu, P., Wilson, P., Redquest, B., Keobouasone, S. & Manseau, M. Seq2Sat and SatAnalyzer toolkit: Towards comprehensive microsatellite genotyping from sequencing data. *Mol. Ecol. Resour.* **24**, e13929 (2024).

6. Rutledge, L. Y. *et al.* Protection from harvesting restores the natural social structure of eastern wolf packs. *Biol. Conserv.* **143**, 332–339 (2010).

7. Peakall, R. & Smouse, P. E. GenAlEx 6.5: genetic analysis in Excel. Population genetic software for teaching and research—an update. *Bioinformatics* **28**, 2537–2539 (2012).

8. Rousset, F. genepop’007: a complete re‐implementation of the genepop software for Windows and Linux. *Mol. Ecol. Resour.* **8**, 103–106 (2008).

9. Falush, D., Stephens, M. & Pritchard, J. K. Inference of population structure using multilocus genotype data: dominant markers and null alleles. *Mol Ecol Notes* **7**, 574–578 (2007).

10. Pritchard, J. K., Stephens, M. & Donnelly, P. Inference of population structure using multilocus genotype data. *Genetics* **155**, 945–959 (2000).

11. Evanno, G., Regnaut, S. & Goudet, J. Detecting the number of clusters of individuals using the software structure: a simulation study. *Mol. Ecol.* **14**, 2611–2620 (2005).

12. Puechmaille, S. J. The program structure does not reliably recover the correct population structure when sampling is uneven: subsampling and new estimators alleviate the problem. *Mol. Ecol. Resour.* **16**, 608–627 (2016).

13. Jombart, T. adegenet: a R package for the multivariate analysis of genetic markers. *Bioinformatics* **24**, 1403–1405 (2008).
