# Supplementary material for: Canis STR‐Seq: A Universal Approach for Non‐Invasive Genetic Monitoring of Wolves and Coyotes
Source: Ecol Evol. 2026 Mar 16;16(3):e73300. doi: 10.1002/ece3.73300 (PMC13093683; doi:10.1002/ece3.73300)
Supplement: Supplementary file 3 — Data S3: ece373300‐sup‐0003‐DataS3.pdf. [file ECE3-16-e73300-s002.pdf]

Supplementary File (Peak Morphology Comparisons). Comparison of microsatellite peak morphologies and scores from capillary electrophoresis (scored in GeneMarker) vs genotyping-by-sequencing (scored in SatAnalyzer) for 15 samples used in this study. Red outlines around SatAnalyzer peaks indicate allele call; orange sections indicate sequence reads with sequence variation, which in some cases* represent a new allele with sequence mutations (see SatAnalyzer manual for details). Allele calles in red respresent a call that is inconsistent or differs from the expected bp shift.

Capillary Electrophoresis - GeneMarker

Genotyping By Sequencing - SatAnalyzer

CfamSTR001 - Allele size difference - 43bp

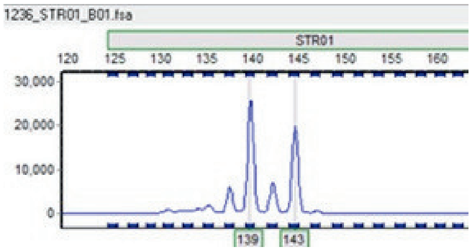

139/143

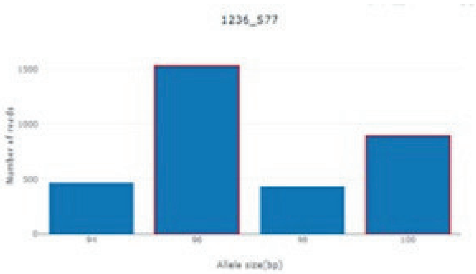

96/100

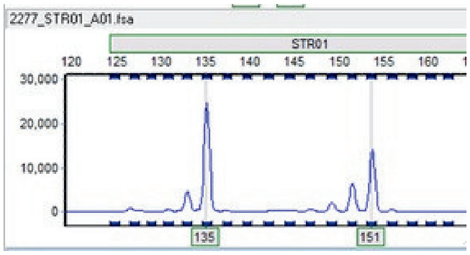

135/151

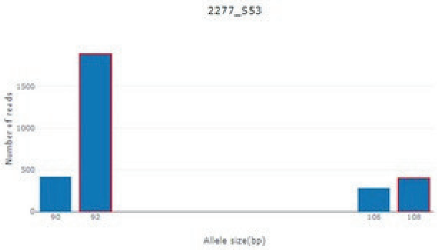

92/108

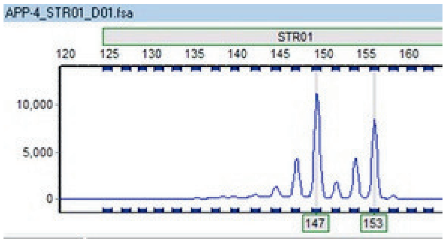

147/153

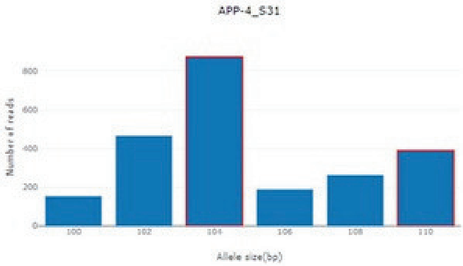

104/110

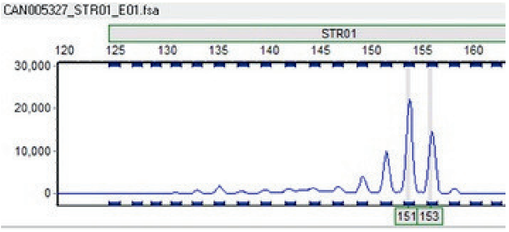

151/153

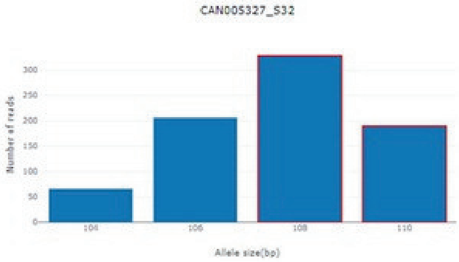

108/110

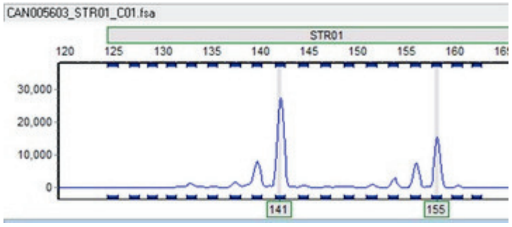

141/155

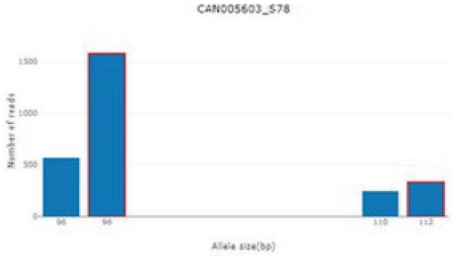

98/112

Capillary Electrophoresis - GeneMarker

Genotyping By Sequencing - SatAnalyzer

CfamSTR001 - Allele size difference - 43bp

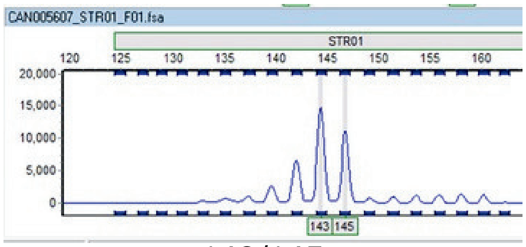

143/145

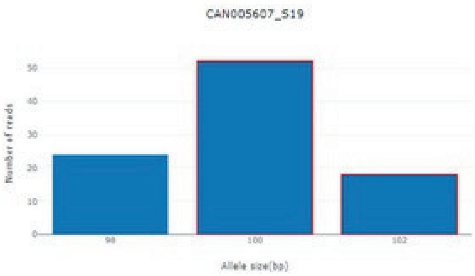

100/102

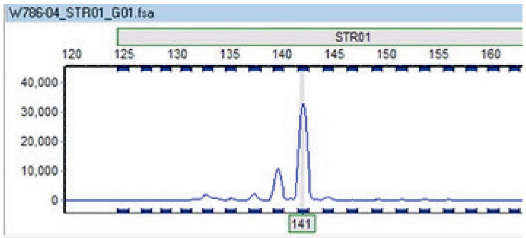

141/141

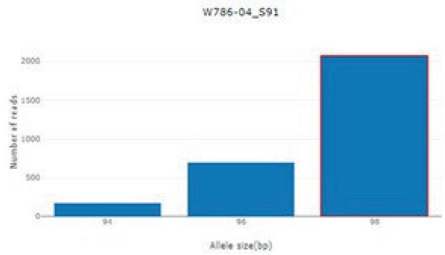

98/98

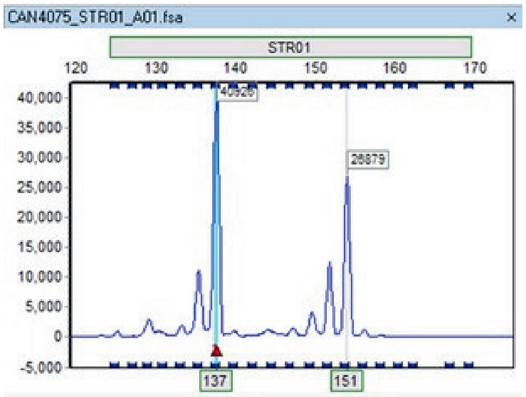

137/151

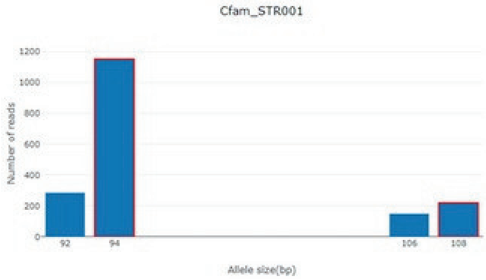

94/108

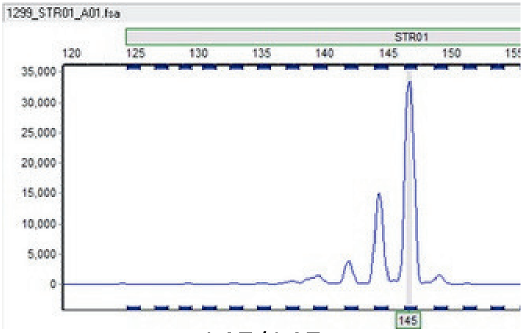

145/145

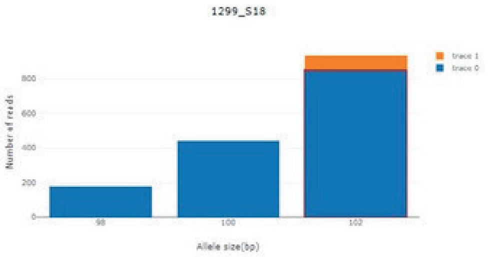

102/102

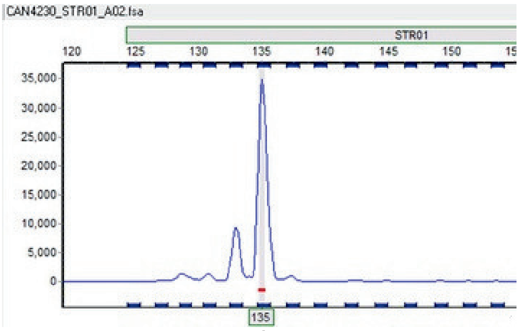

135/135

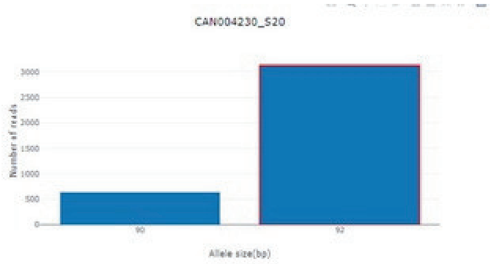

92/92

Capillary Electrophoresis - GeneMarker

Genotyping By Sequencing - SatAnalyzer

CfamSTR001 - Allele size difference - 43bp

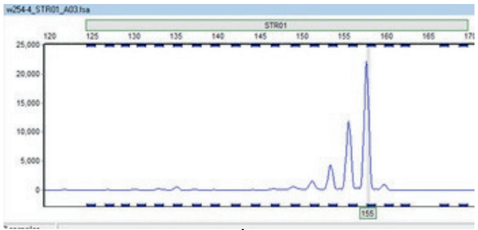

155/155

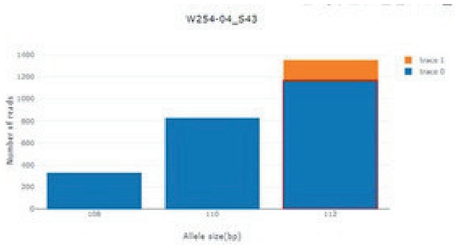

112/112

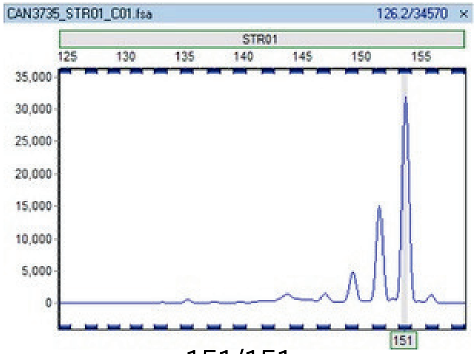

151/151

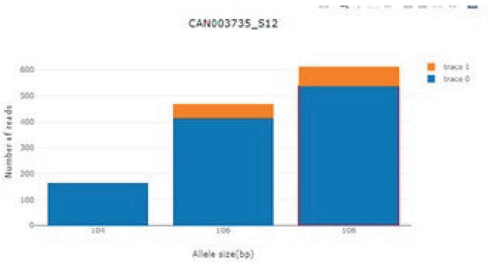

108/108

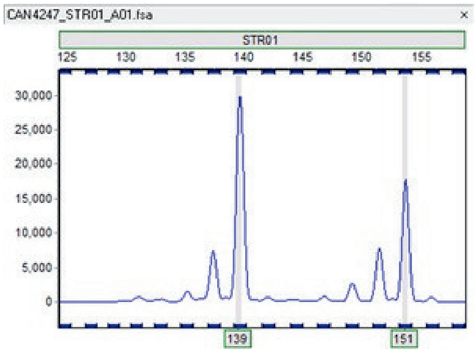

139/151

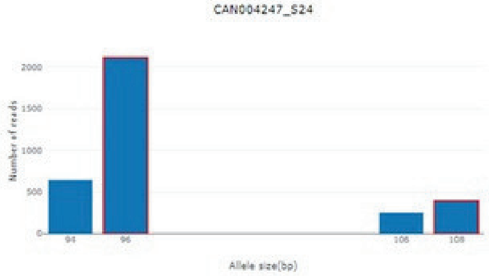

96/108

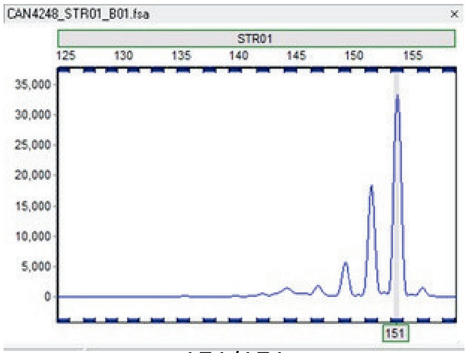

151/151

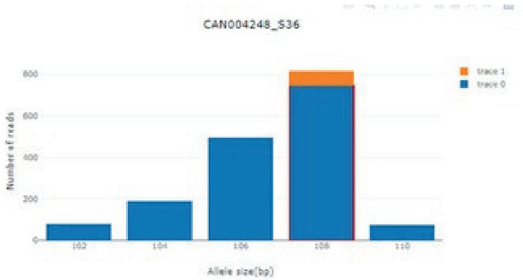

108/108

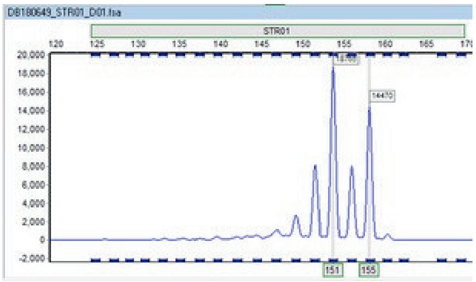

151/155

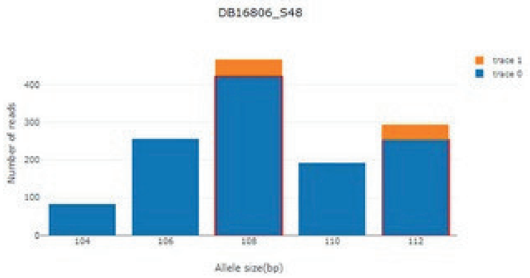

108/112

Capillary Electrophoresis - GeneMarker

Genotyping By Sequencing - SatAnalyzer

CfamSTR002 - Allele size difference - 38bp

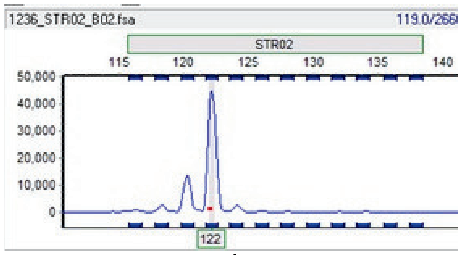

122/122

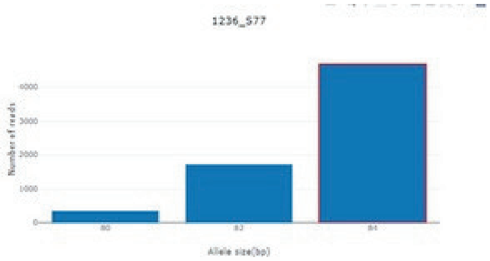

84/84

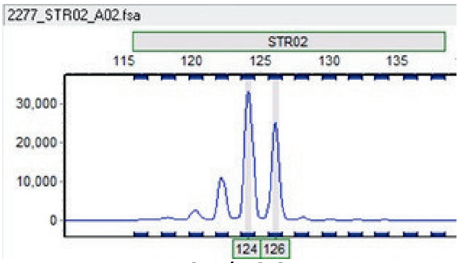

124/126

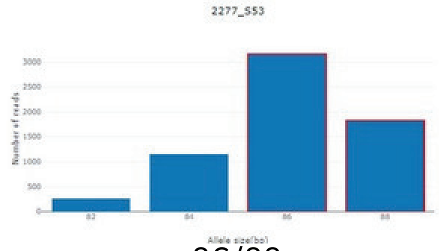

86/88

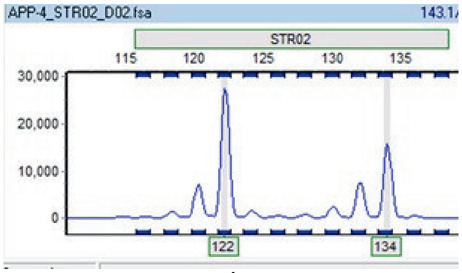

122/134

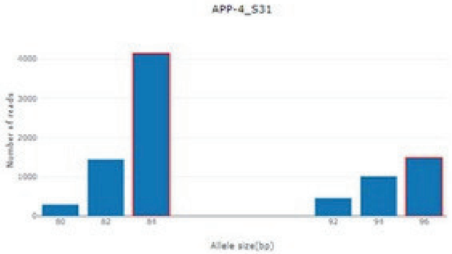

84/96

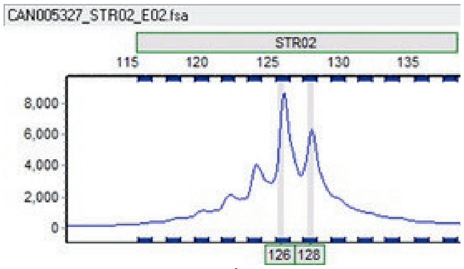

126/128

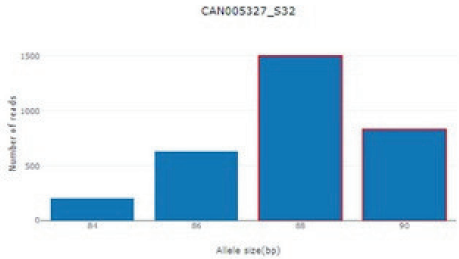

88/90

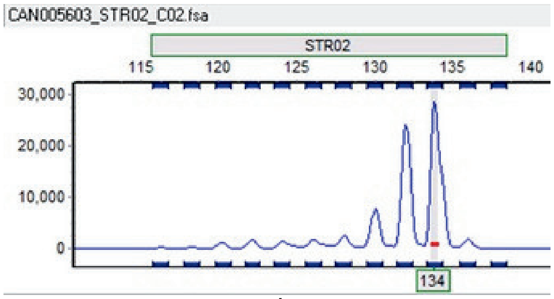

134/134

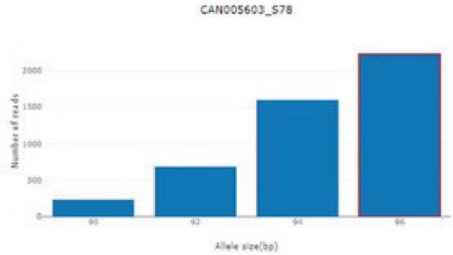

96/96

Capillary Electrophoresis - GeneMarker

Genotyping By Sequencing - SatAnalyzer

CfamSTR002 - Allele size difference - 38bp

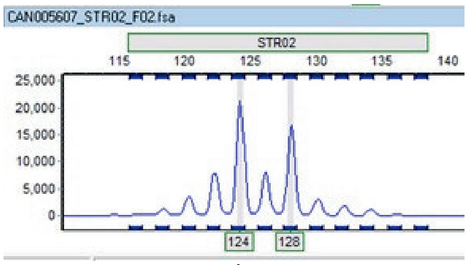

124/128

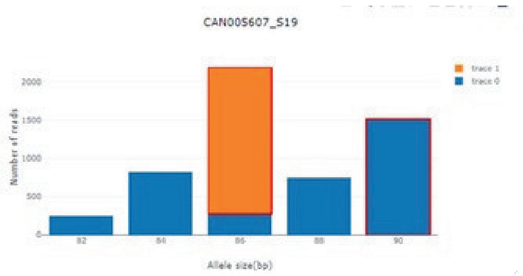

86/90

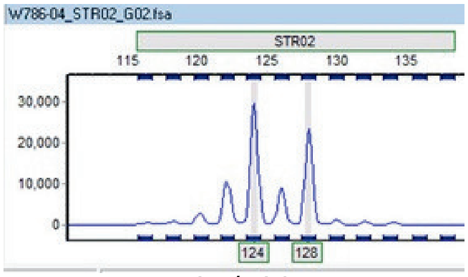

124/128

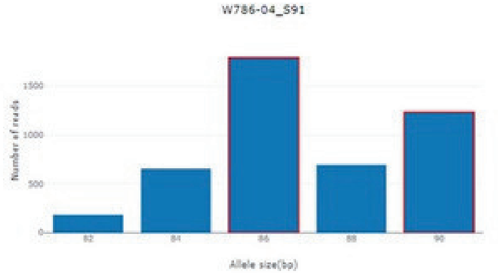

86/90

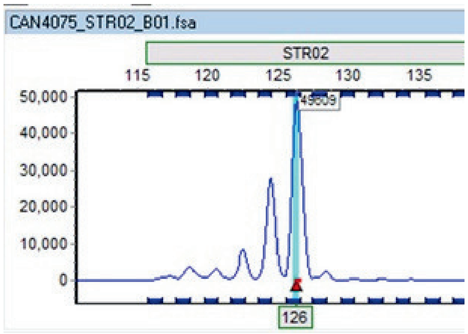

126/126

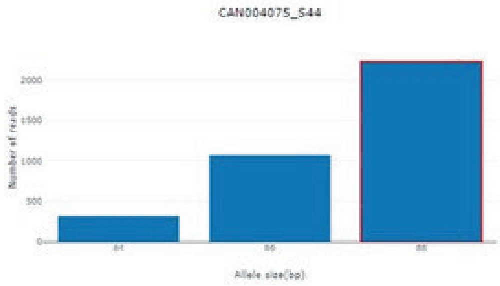

88/88

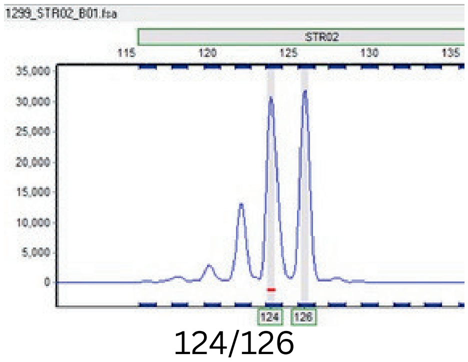

124/126

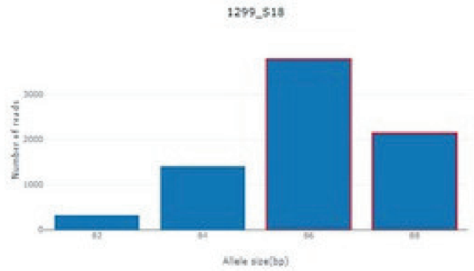

86/88

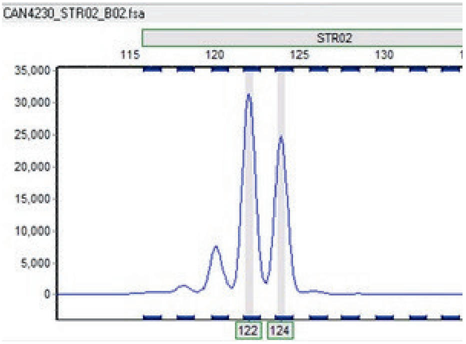

122/124

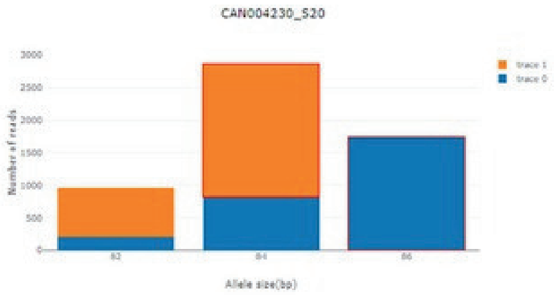

84/86

Capillary Electrophoresis - GeneMarker

Genotyping By Sequencing - SatAnalyzer

CfamSTR002 - Allele size difference - 38bp

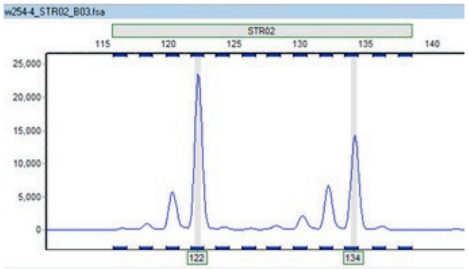

122/134

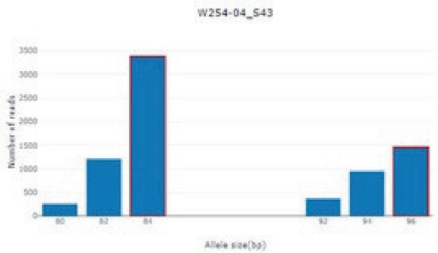

84/96

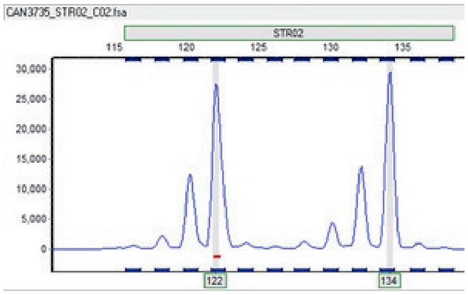

122/134

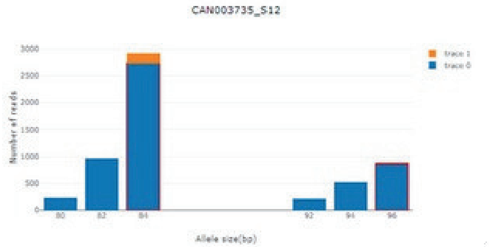

84/96

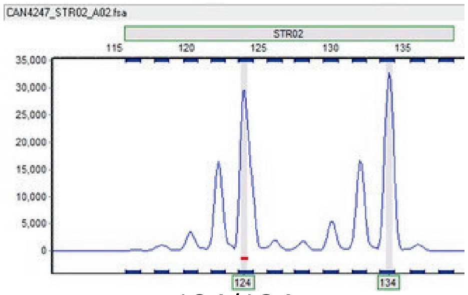

124/134

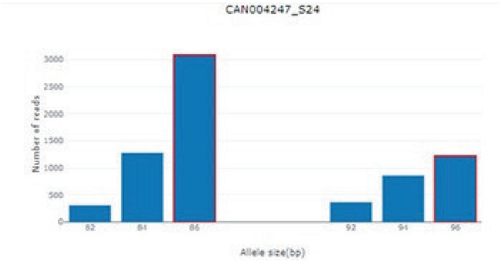

86/96

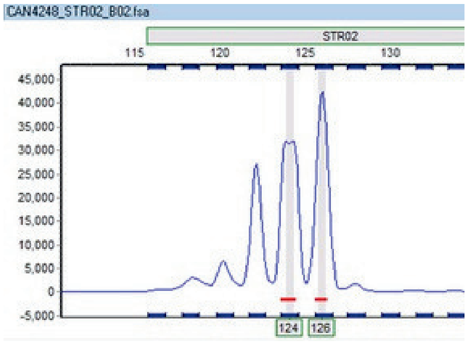

124/126

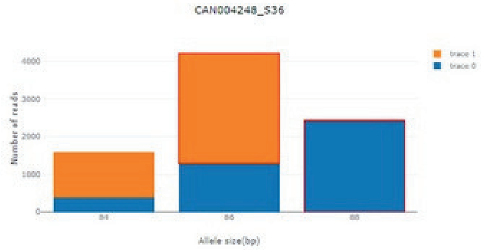

86/88

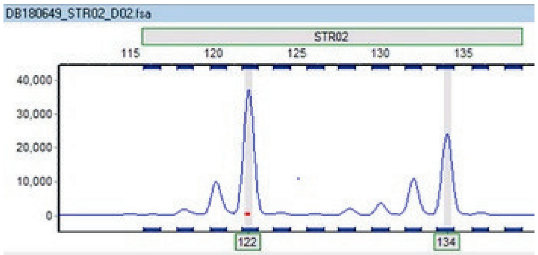

122/134

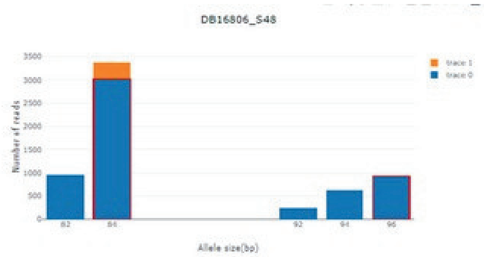

84/96

Capillary Electrophoresis - GeneMarker

Genotyping By Sequencing - SatAnalyzer

CfamSTR003 - Allele size difference - 41 and 42 bp

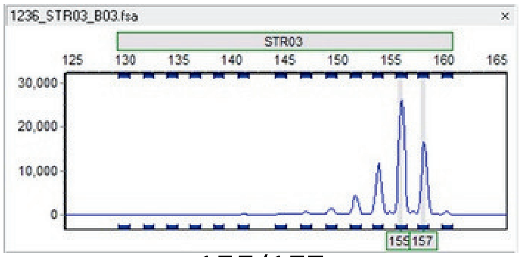

155/157

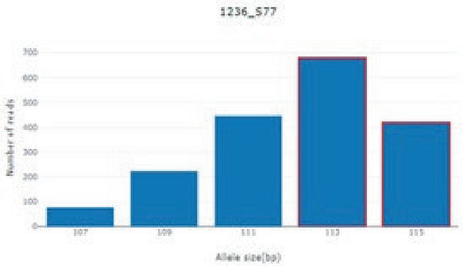

113/115

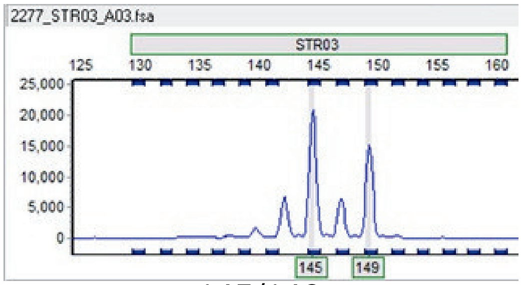

145/149

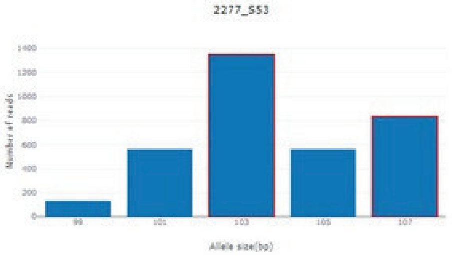

103/107

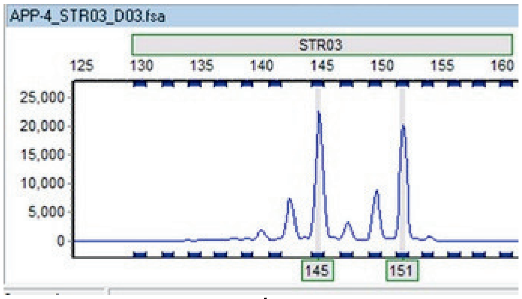

145/151

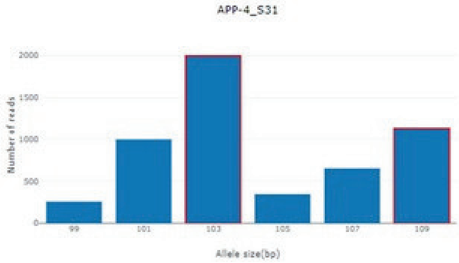

103/109

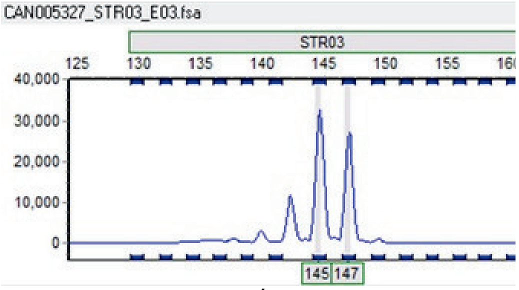

145/147

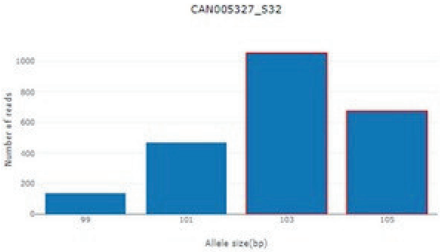

103/105

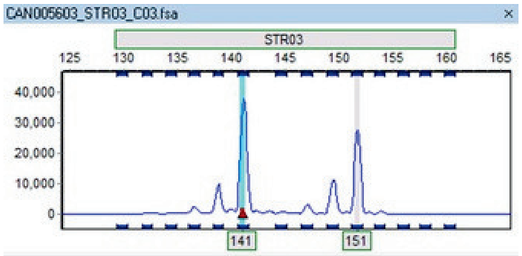

141/151

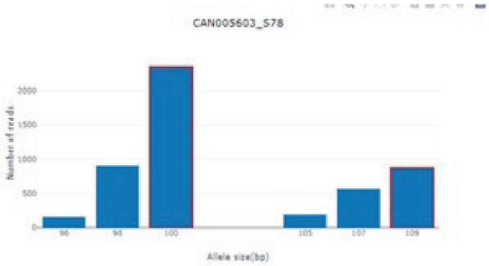

100/109

Capillary Electrophoresis - GeneMarker

Genotyping By Sequencing - SatAnalyzer

CfamSTR003 - Allele size difference - 41 and 42 bp

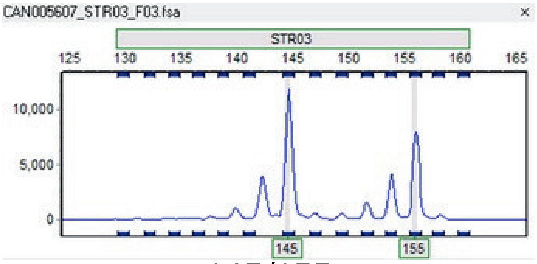

145/155

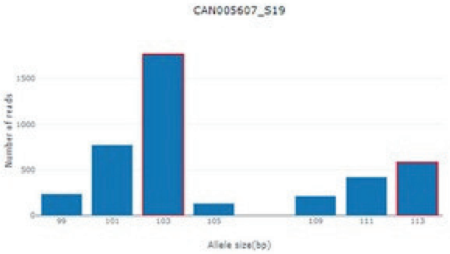

103/113

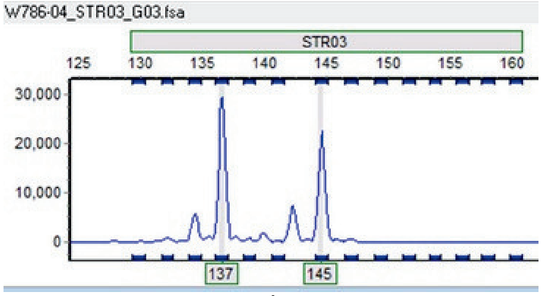

137/145

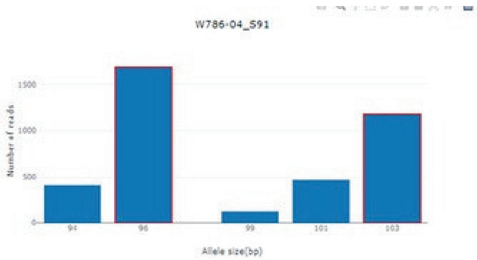

96/103

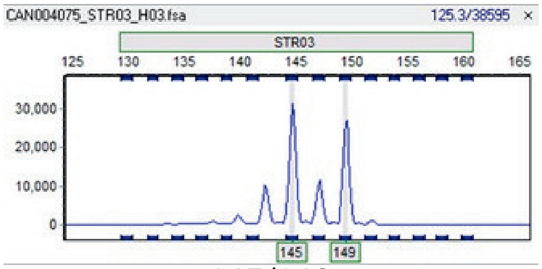

145/149

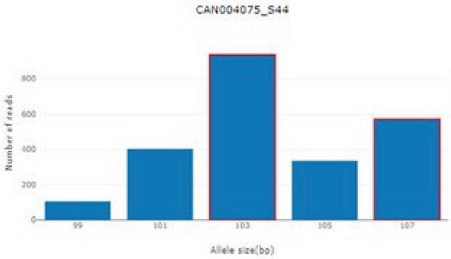

103/107

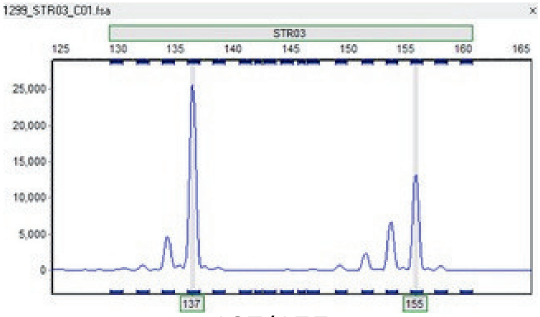

137/155

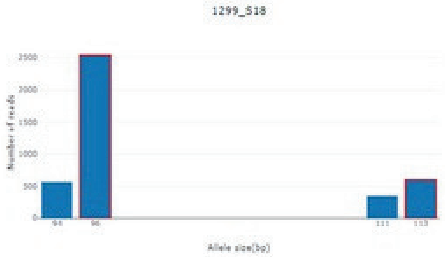

96/113

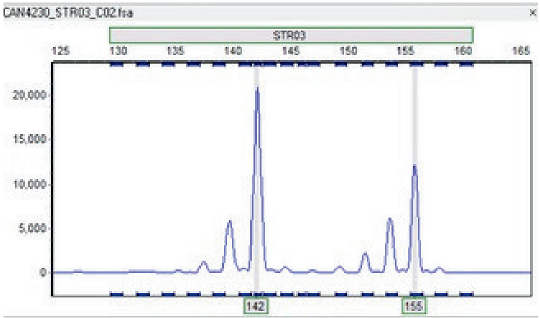

142/155

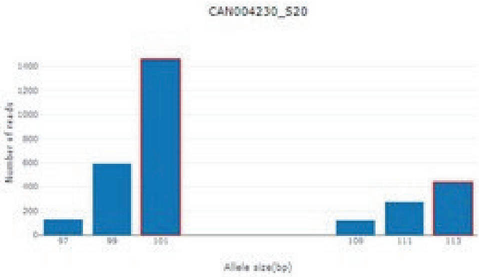

101/113

Capillary Electrophoresis - GeneMarker

Genotyping By Sequencing - SatAnalyzer

CfamSTR003 - Allele size difference - 41 and 42 bp

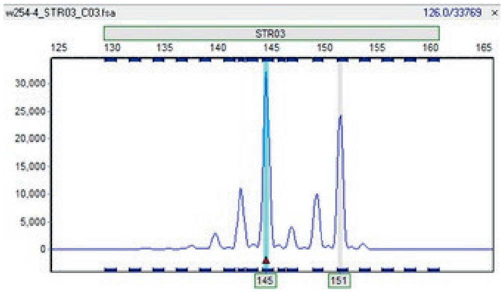

145/151

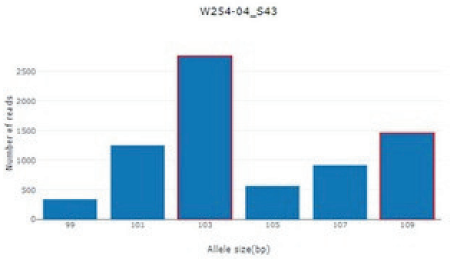

103/109

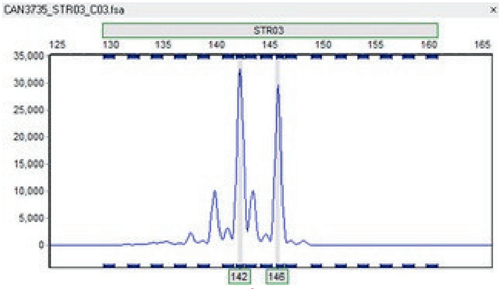

142/146

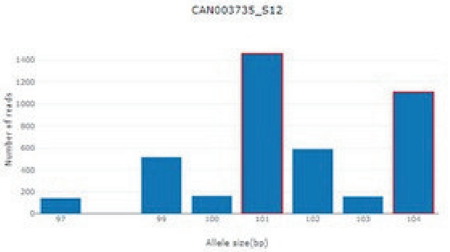

101/104

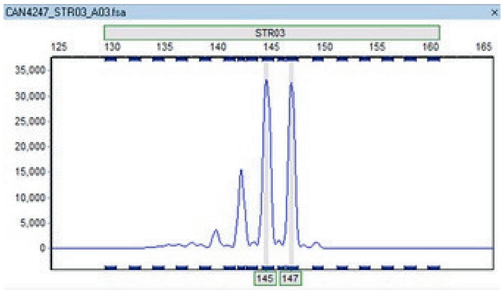

145/147

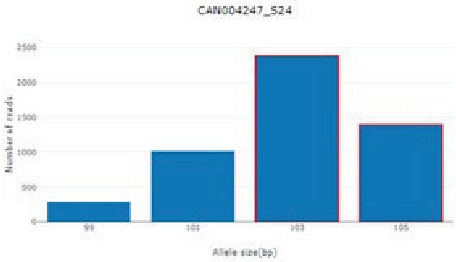

103/105

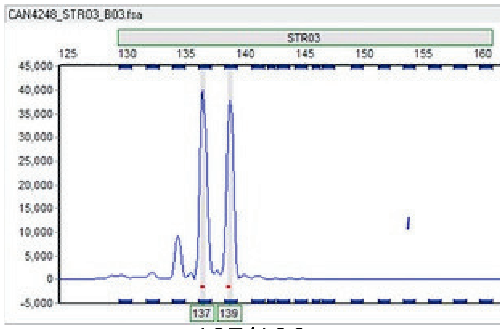

137/139

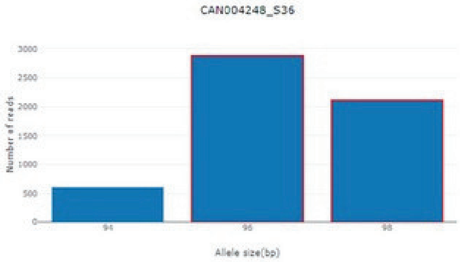

96/98

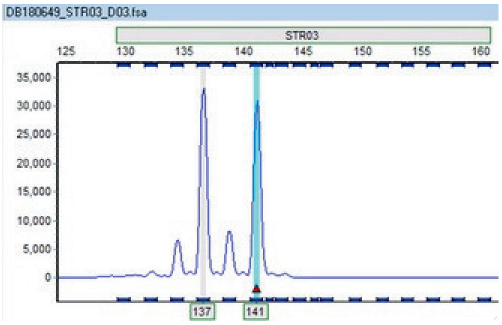

137/141

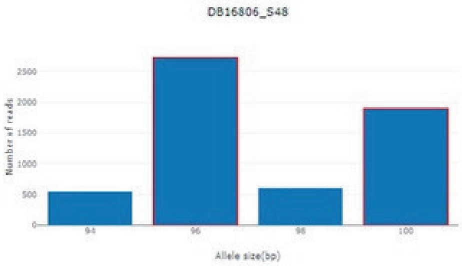

96/100

Capillary Electrophoresis - GeneMarker

Genotyping By Sequencing - SatAnalyzer

CfamSTR004 - Allele size difference - 34 bp

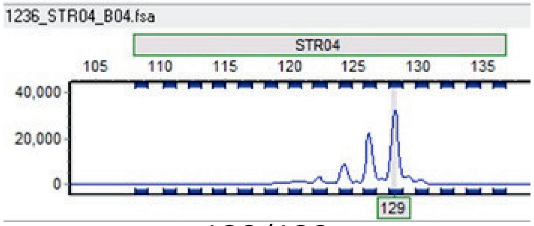

129/129

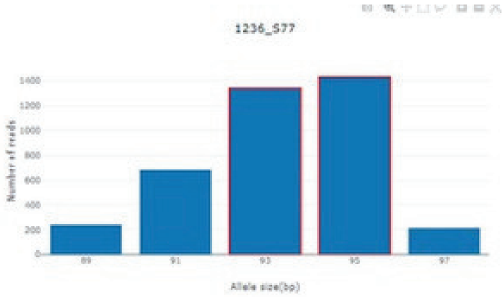

93/95

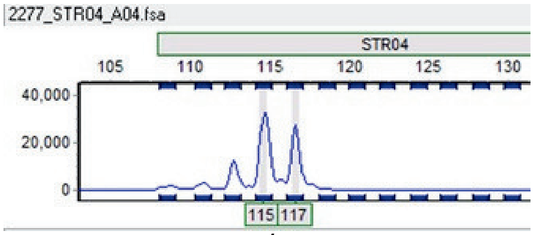

115/117

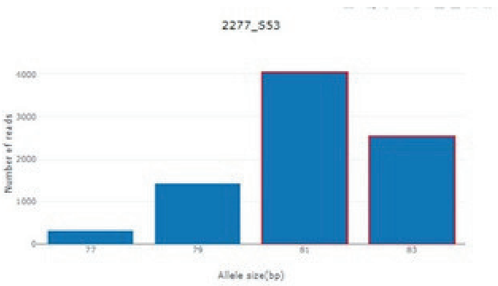

81/83

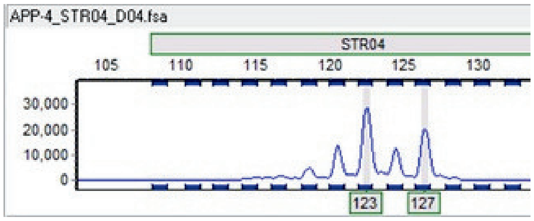

123/127

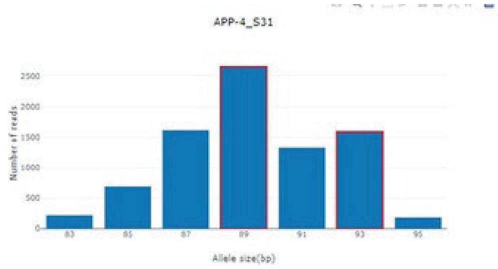

89/93

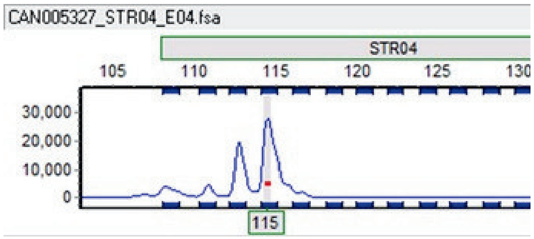

115/115

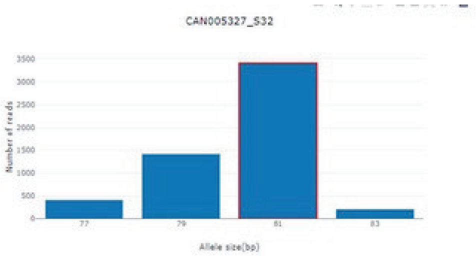

81/81

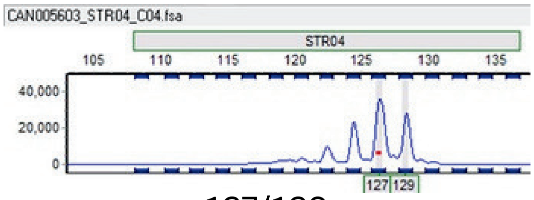

127/129

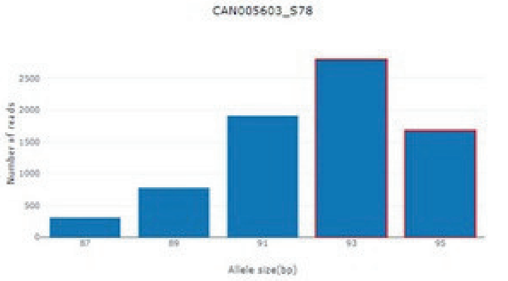

93/95

Capillary Electrophoresis - GeneMarker

Genotyping By Sequencing - SatAnalyzer

CfamSTR004 - Allele size difference - 34 bp

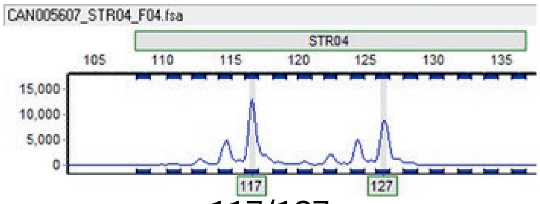

117/127

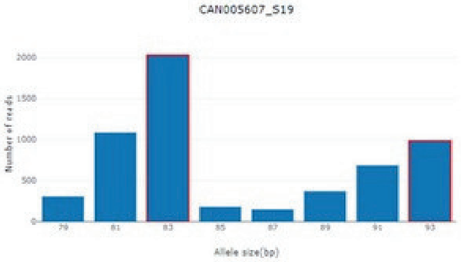

83/93

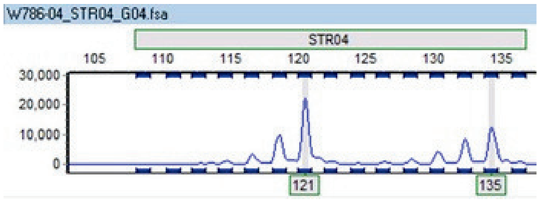

121/135

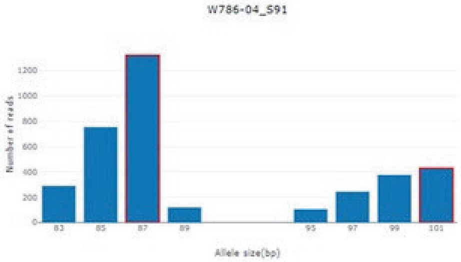

87/101

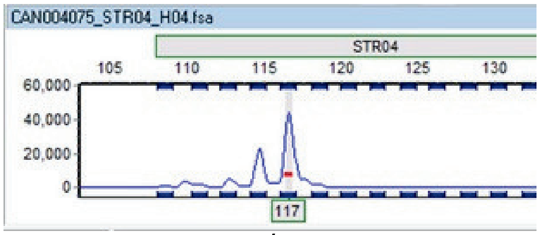

117/117

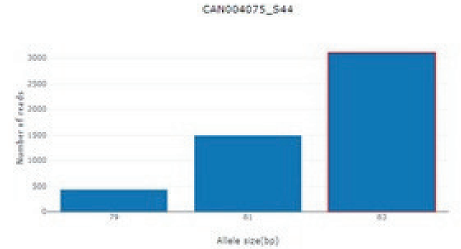

83/83

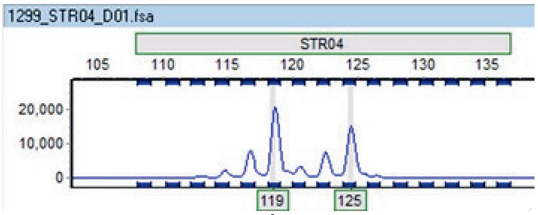

119/125

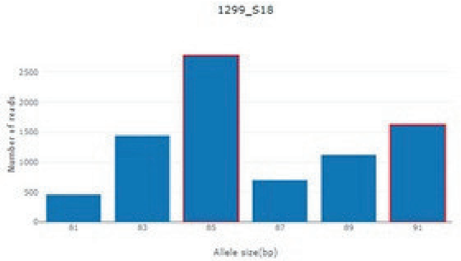

85/91

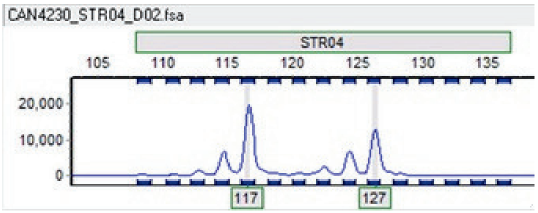

117/127

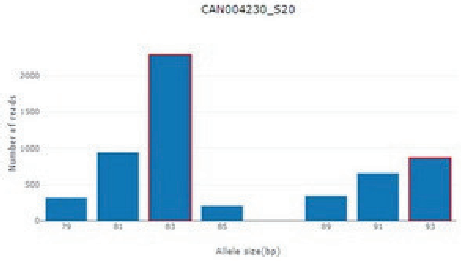

83/93

Capillary Electrophoresis - GeneMarker

Genotyping By Sequencing - SatAnalyzer

CfamSTR004 - Allele size difference - 34 bp

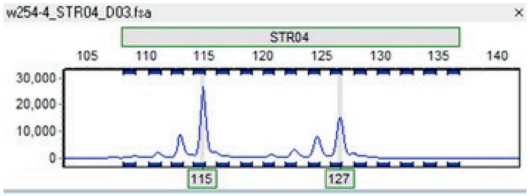

115/127

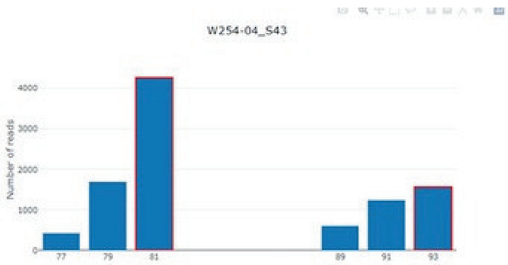

81/93

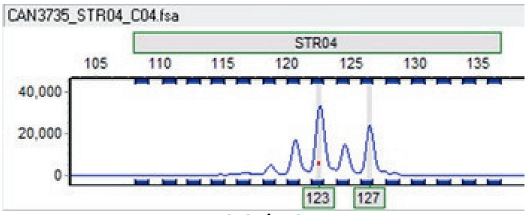

123/127

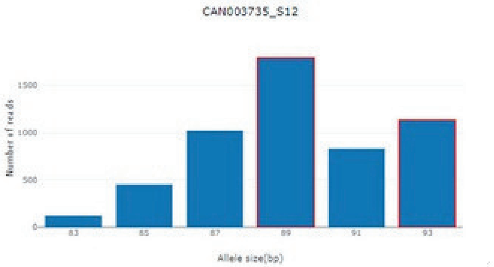

89/93

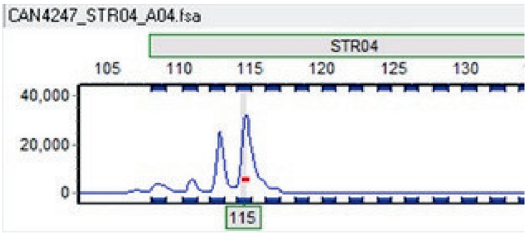

115/115

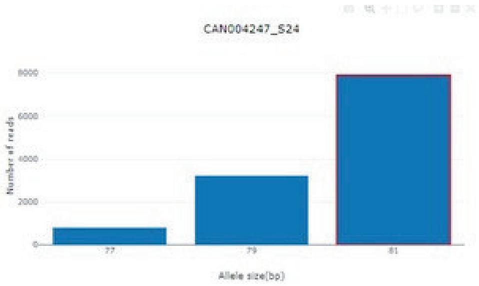

81/81

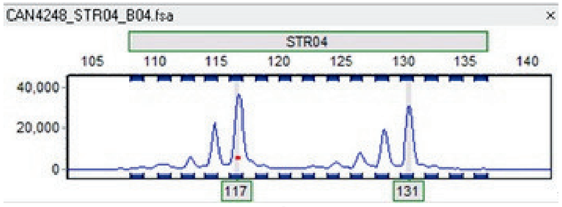

117/131

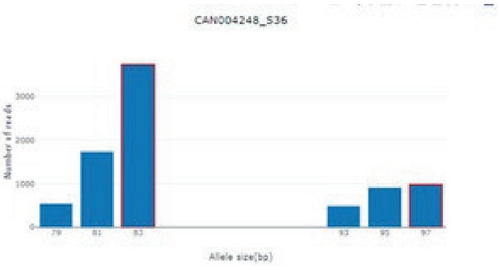

83/97

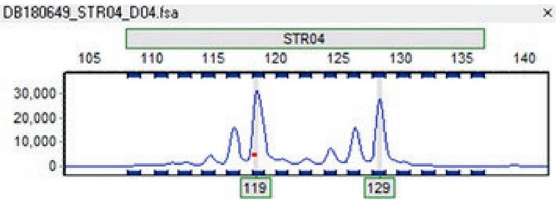

119/129

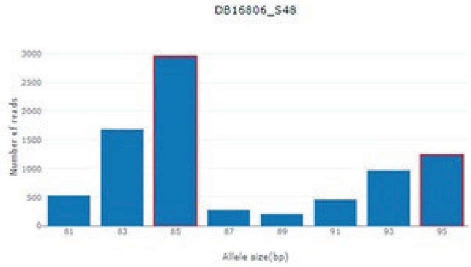

85/95

Capillary Electrophoresis - GeneMarker

Genotyping By Sequencing - SatAnalyzer

CfamSTR005 - Allele size difference - 39 bp

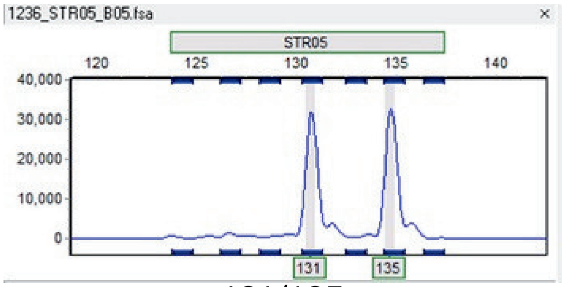

131/135

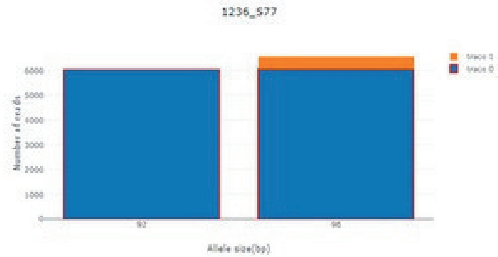

92/96

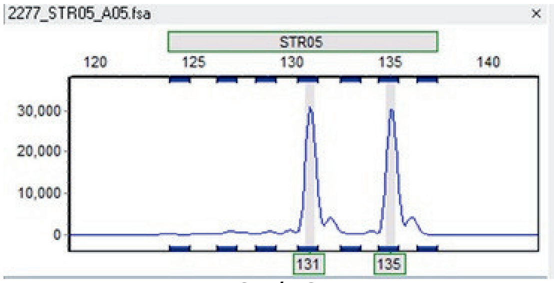

131/135

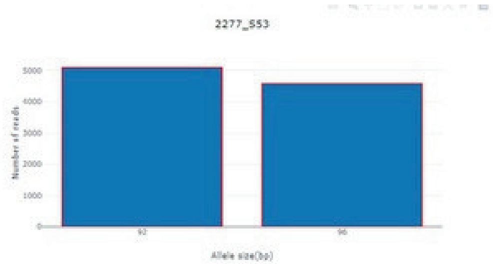

92/96

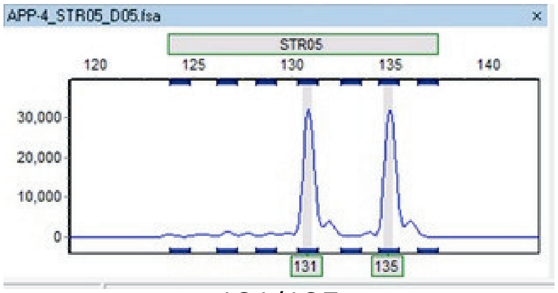

131/135

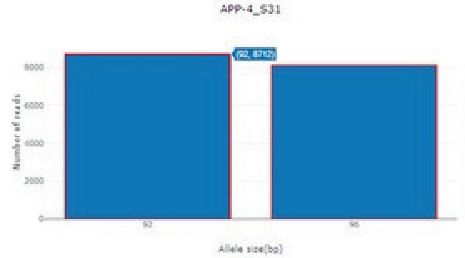

92/96

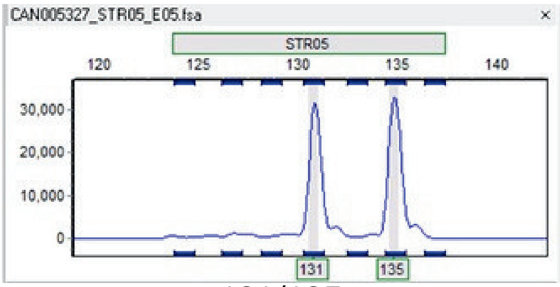

131/135

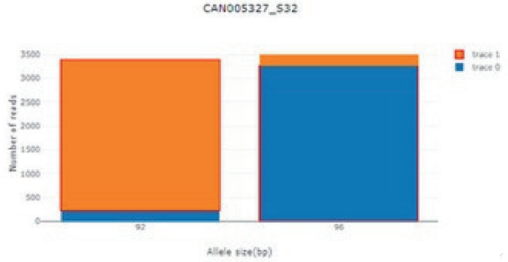

92/96

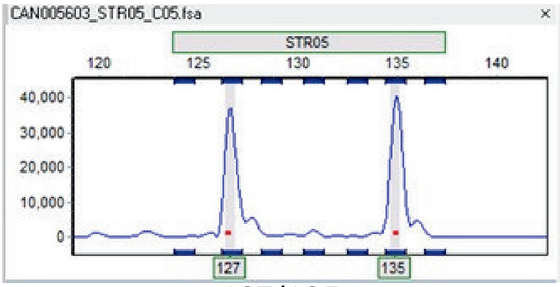

127/135

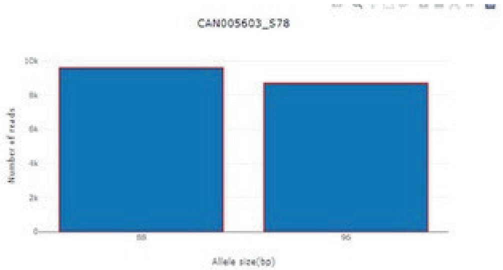

88/96

Capillary Electrophoresis - GeneMarker

Genotyping By Sequencing - SatAnalyzer

CfamSTR005 - Allele size difference - 39 bp

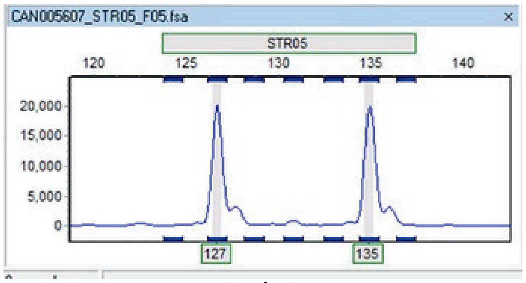

127/135

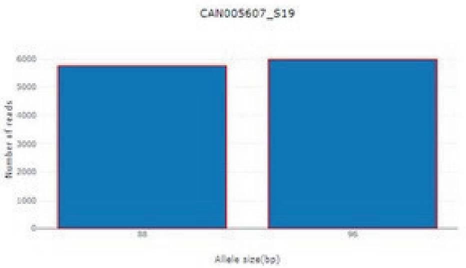

88/96

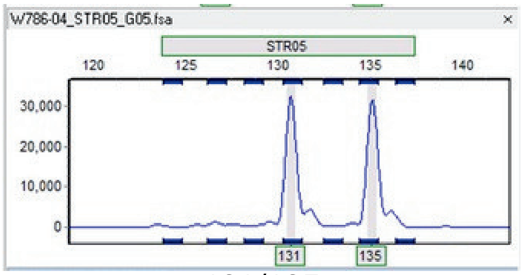

131/135

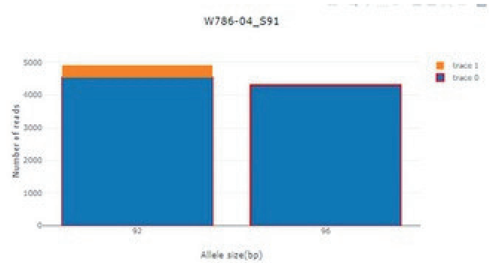

92/96

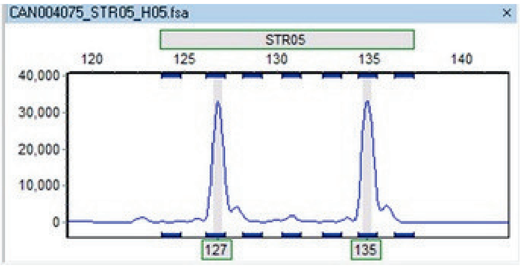

127/135

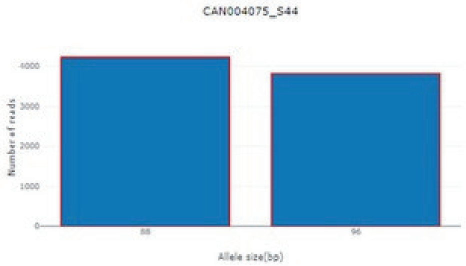

88/96

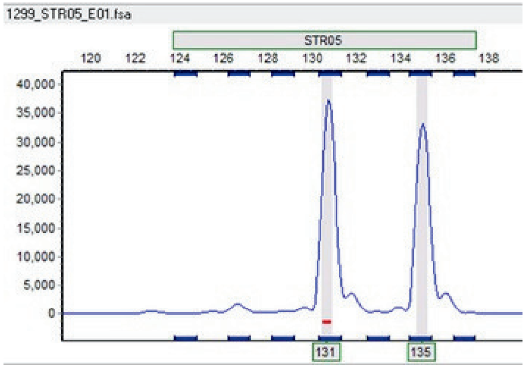

131/135

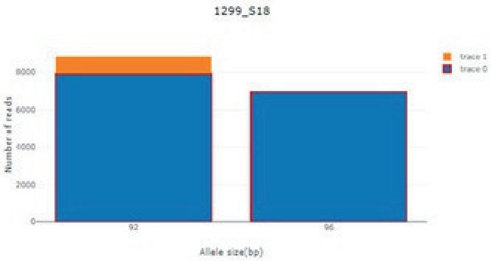

92/96

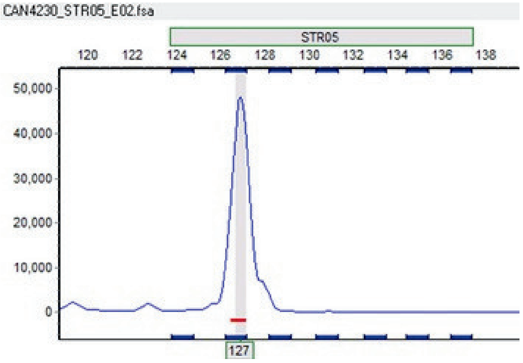

127/127

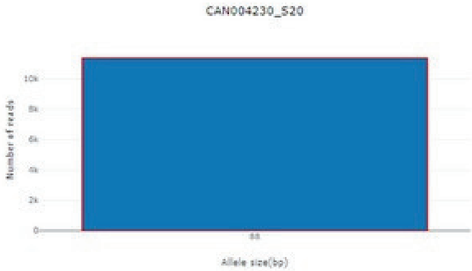

88/88

Capillary Electrophoresis - GeneMarker

Genotyping By Sequencing - SatAnalyzer

CfamSTR005 - Allele size difference - 39 bp

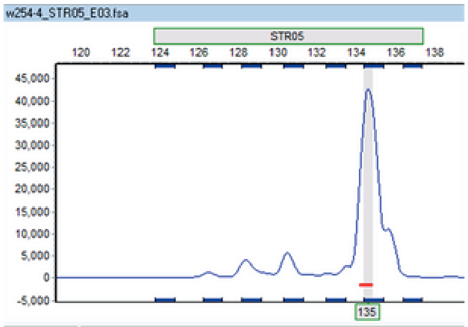

135/135

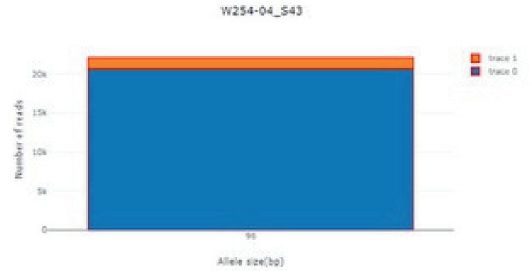

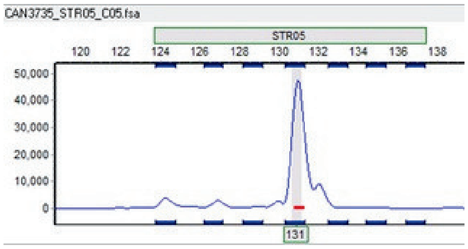

131/131

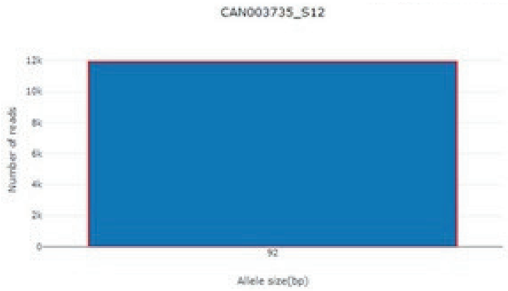

92/92

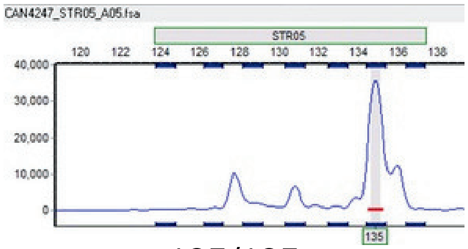

135/135

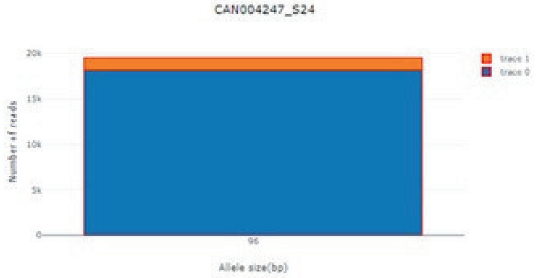

96/96

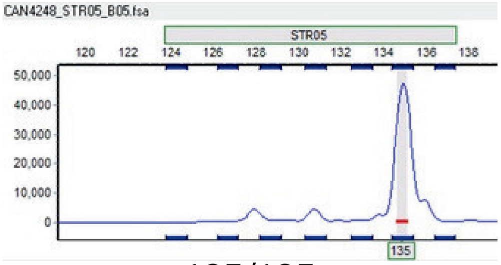

135/135

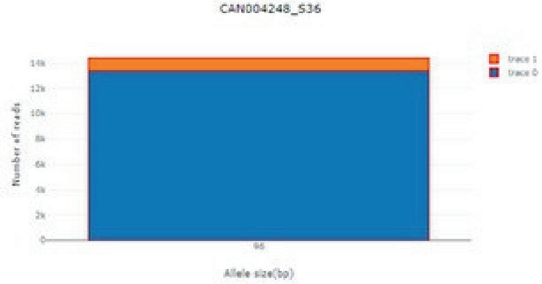

96/96

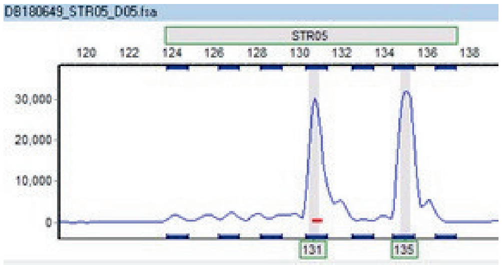

131/135

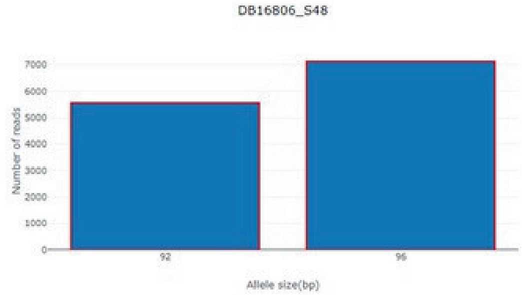

92/96

Capillary Electrophoresis - GeneMarker

Genotyping By Sequencing - SatAnalyzer

CfamSTR007 - Allele size difference - 38 bp

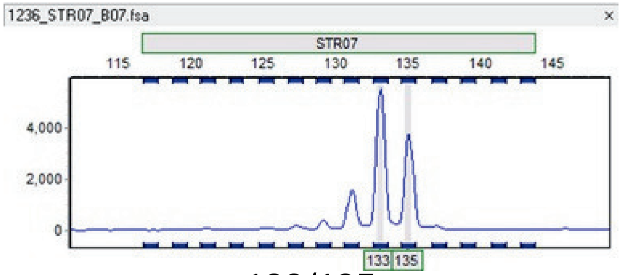

133/135

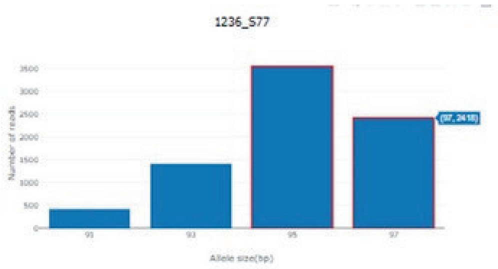

95/97

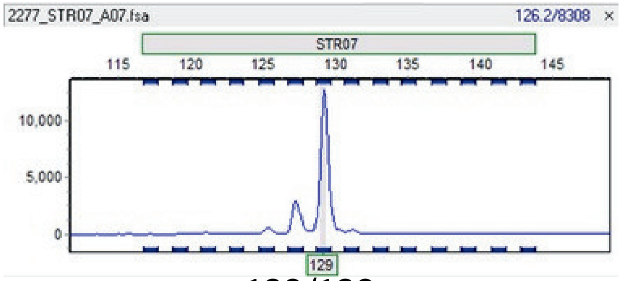

129/129

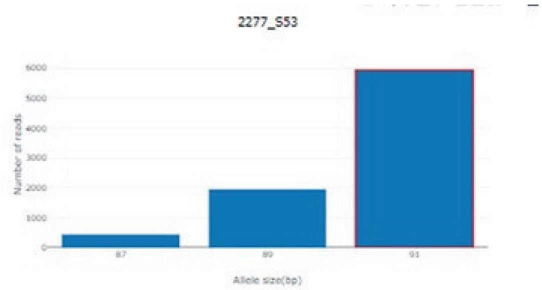

91/91

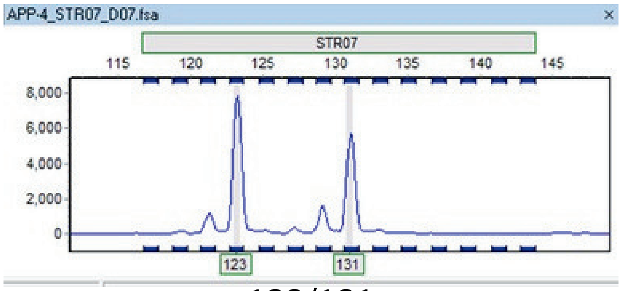

123/131

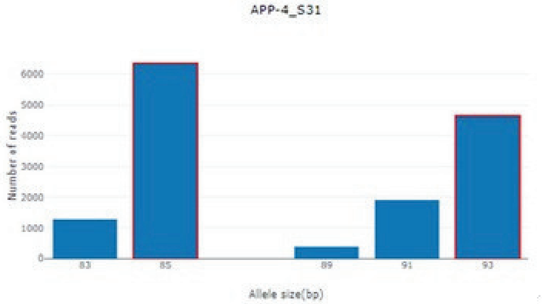

85/93

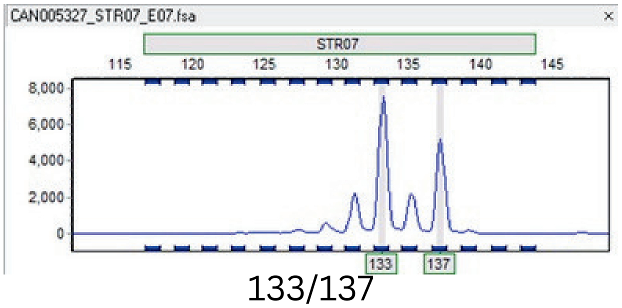

133/137

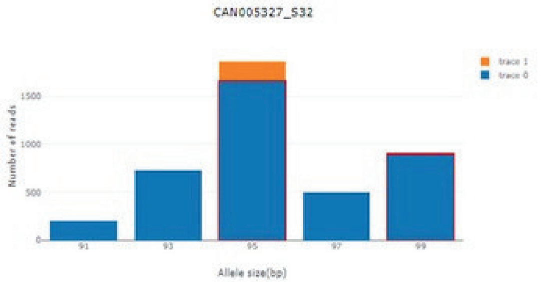

95/99

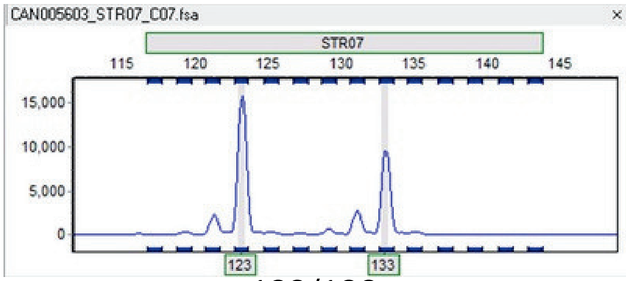

123/133

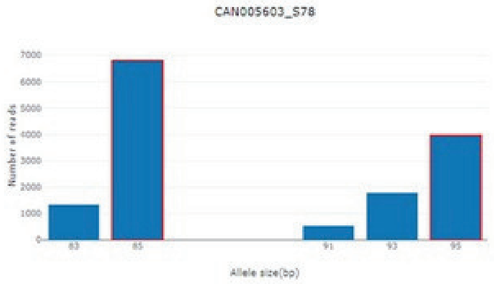

85/95

Capillary Electrophoresis - GeneMarker

Genotyping By Sequencing - SatAnalyzer

CfamSTR007 - Allele size difference - 38 bp

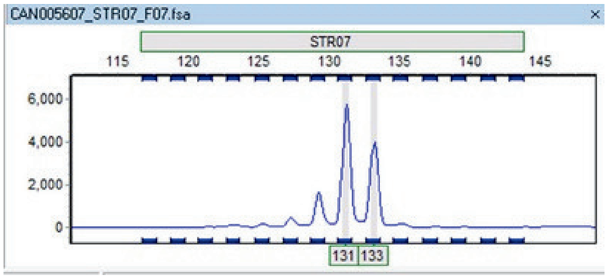

131/133

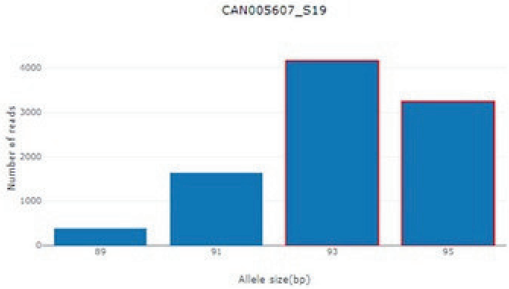

93/95

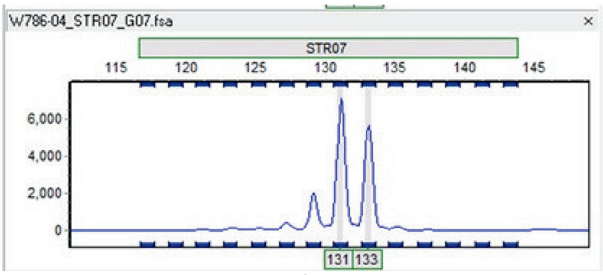

131/133

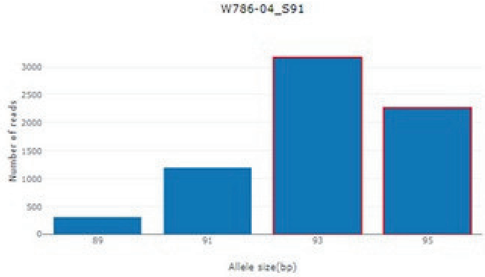

93/95

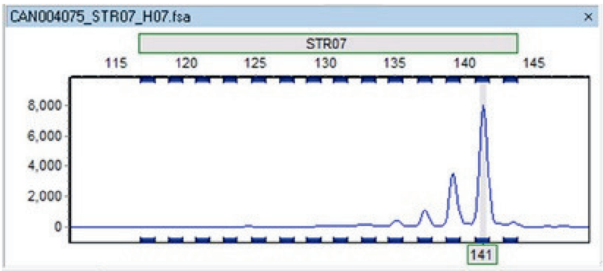

141/141

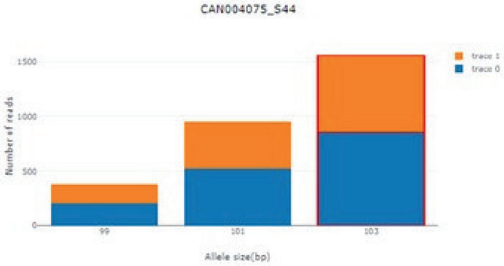

103/103*

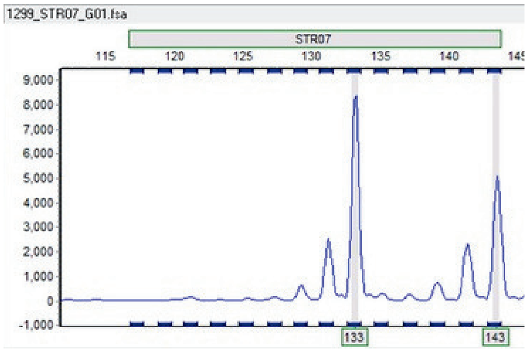

133/141

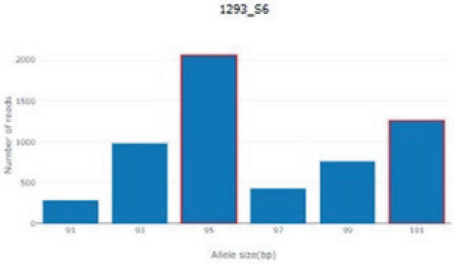

95/103

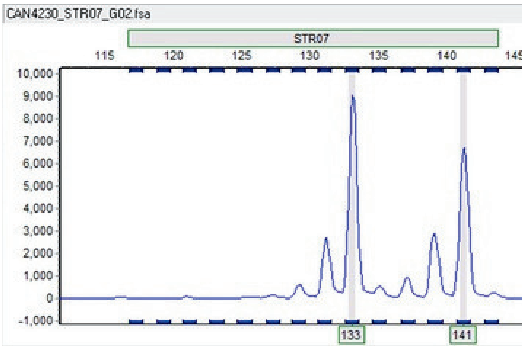

133/141

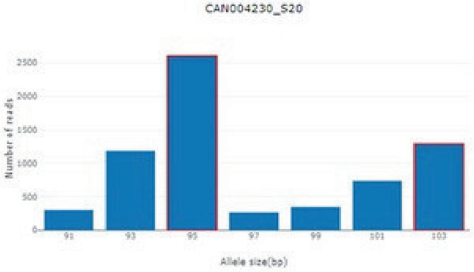

95/103

Capillary Electrophoresis - GeneMarker

Genotyping By Sequencing - SatAnalyzer

CfamSTR007 - Allele size difference - 38 bp

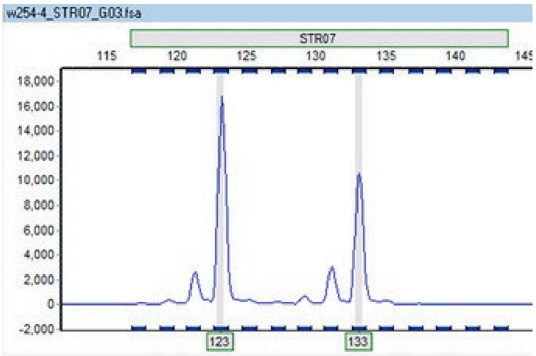

123/133

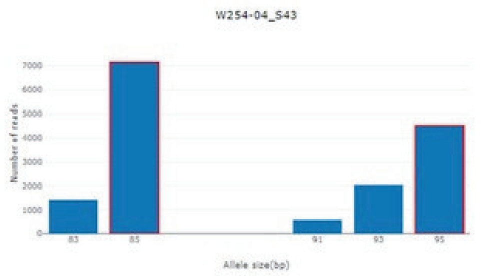

85/95

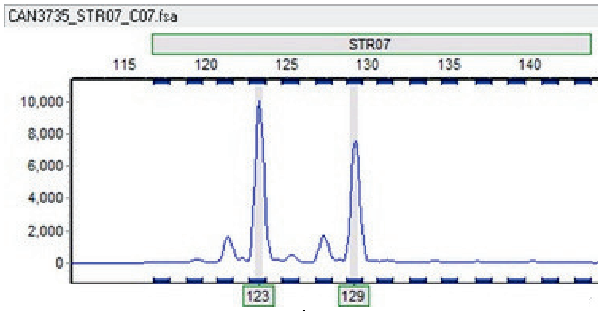

123/129

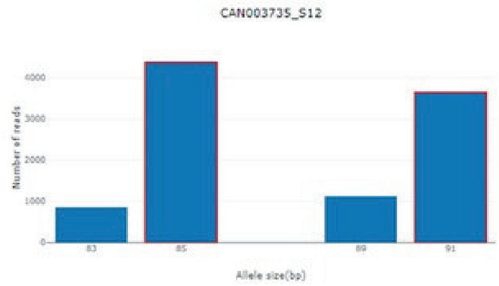

85/91

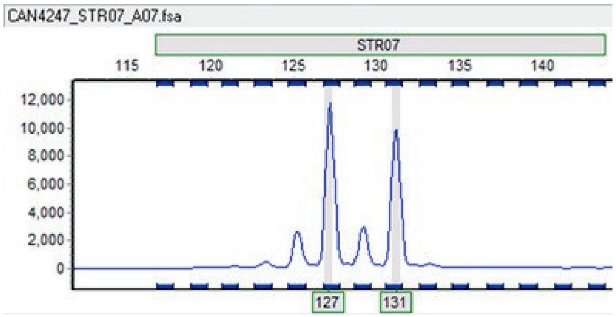

127/131

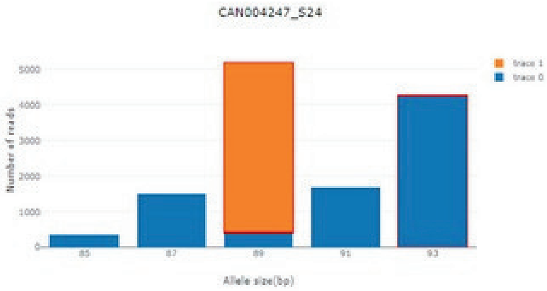

89/93

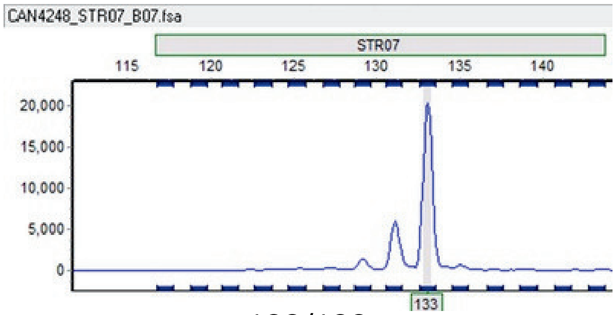

133/133

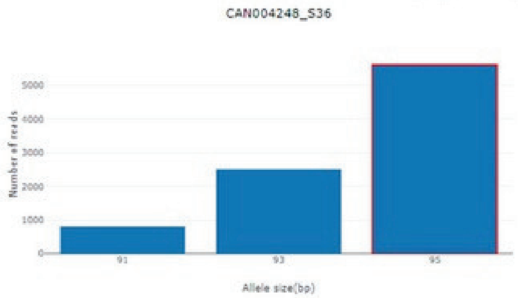

95/95

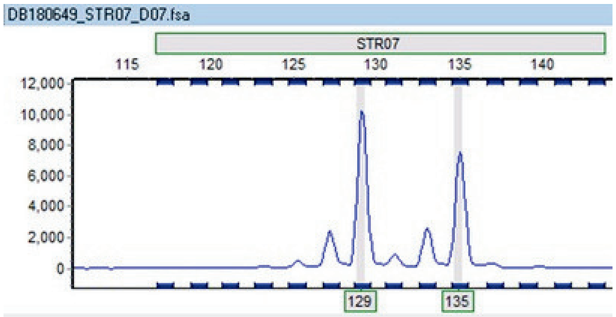

129/135

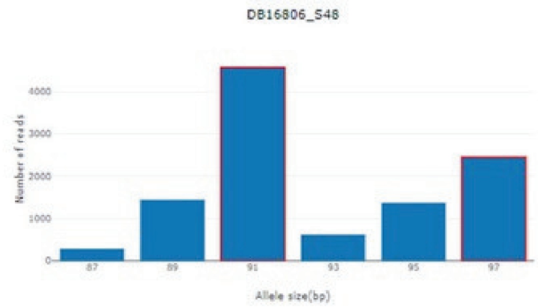

91/97

Capillary Electrophoresis - GeneMarker

Genotyping By Sequencing - SatAnalyzer

CfamSTR008 - Allele size difference - 36 bp

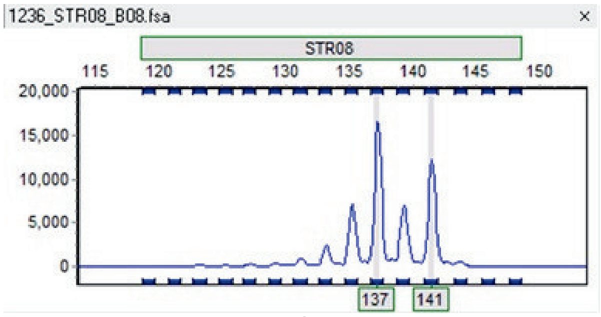

137/141

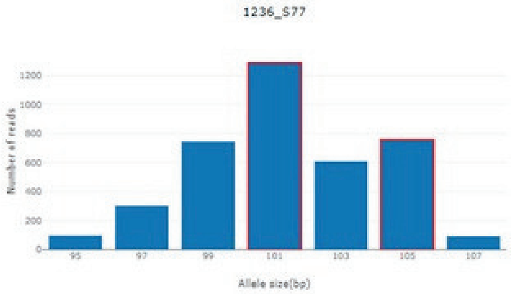

101/105

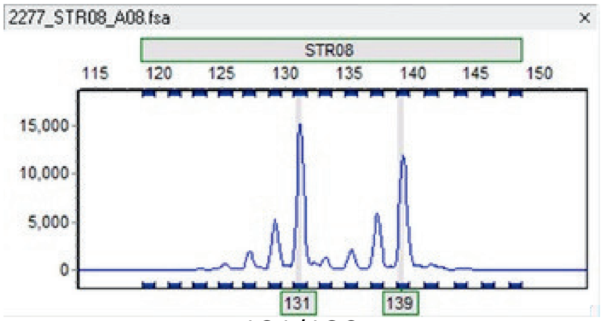

131/139

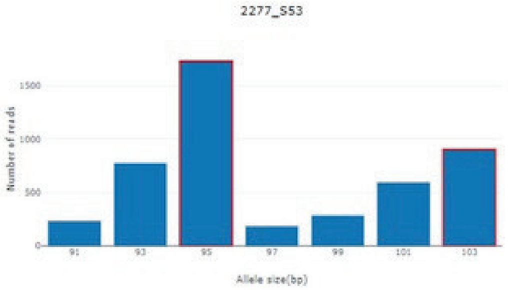

95/103

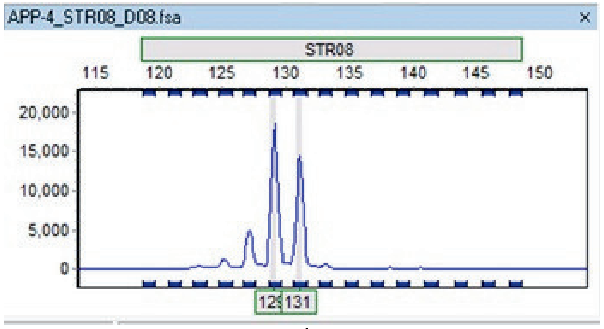

129/131

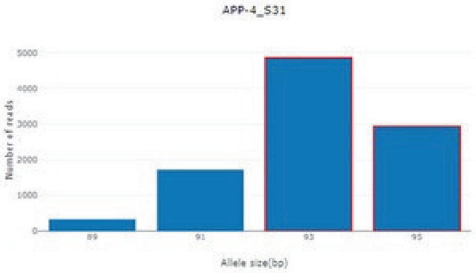

93/95

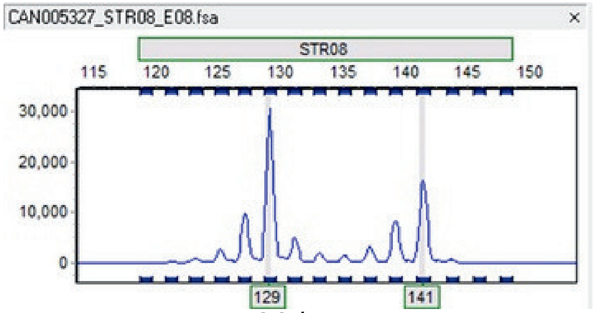

129/141

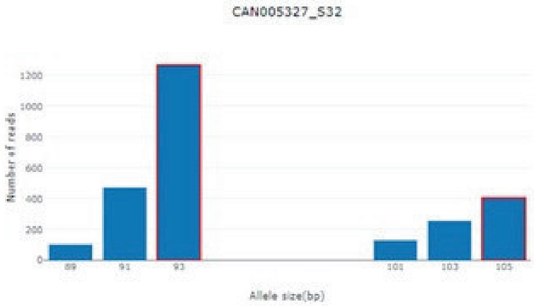

93/105

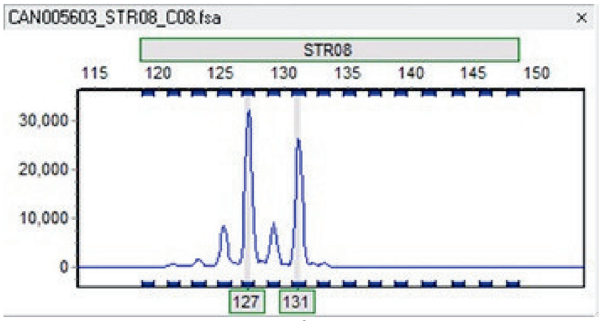

127/131

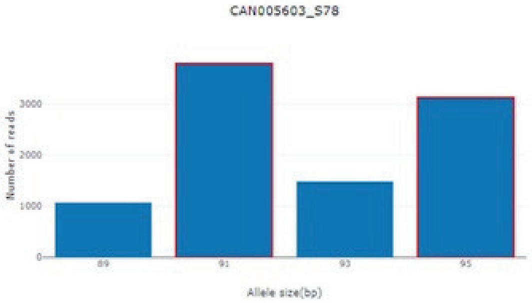

91/95

Capillary Electrophoresis - GeneMarker

Genotyping By Sequencing - SatAnalyzer

CfamSTR008 - Allele size difference - 36 bp

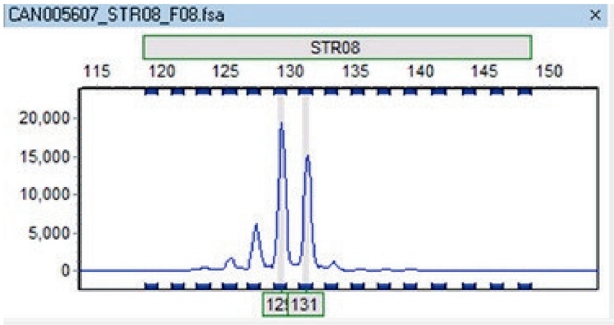

129/131

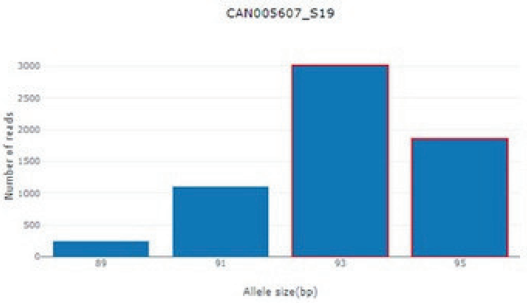

93/95

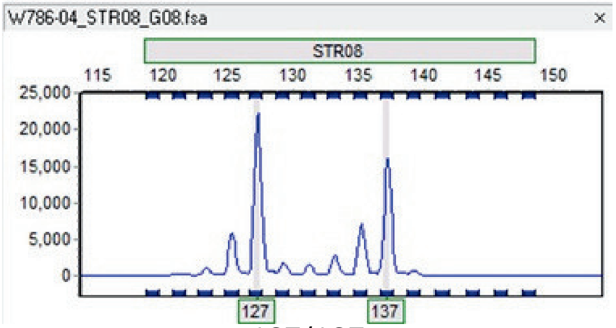

127/137

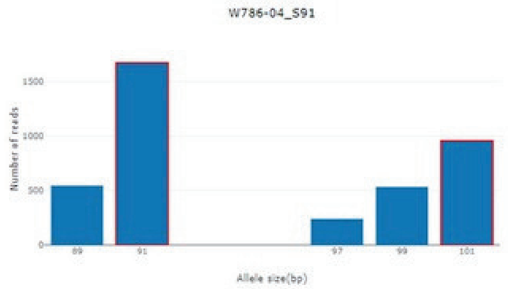

91/101

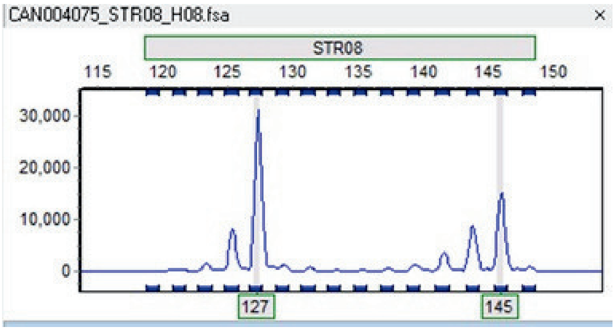

127/145

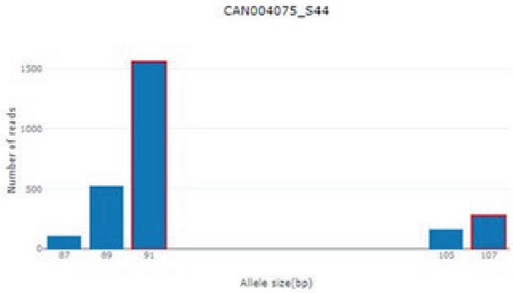

91/109

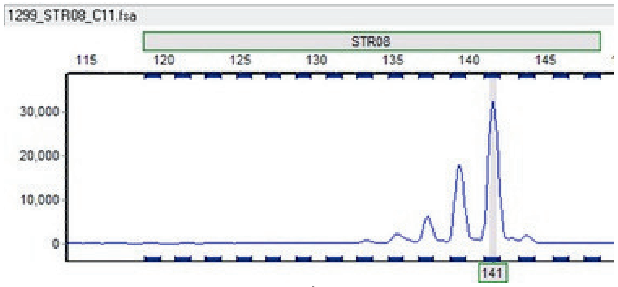

141/141

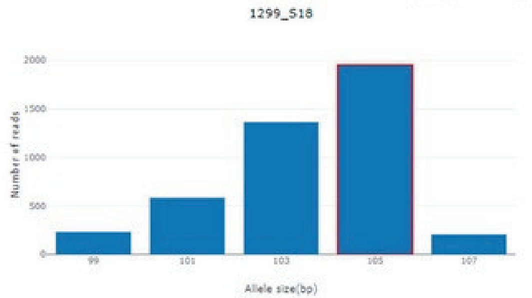

105/105

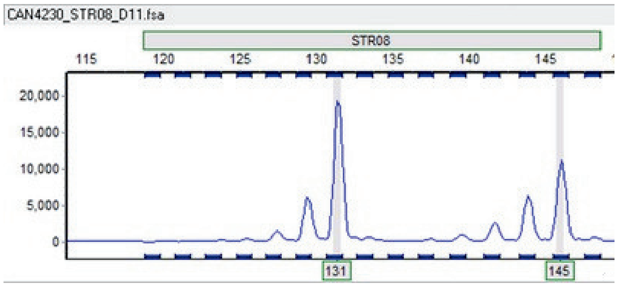

131/145

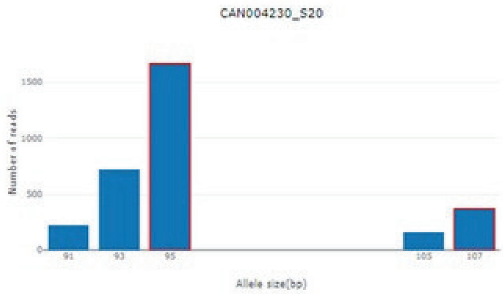

95/107

Capillary Electrophoresis - GeneMarker

Genotyping By Sequencing - SatAnalyzer

CfamSTR008 - Allele size difference - 36 bp

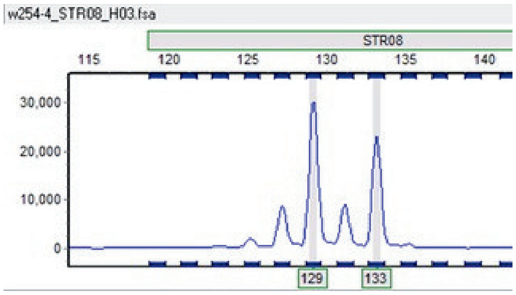

129/133

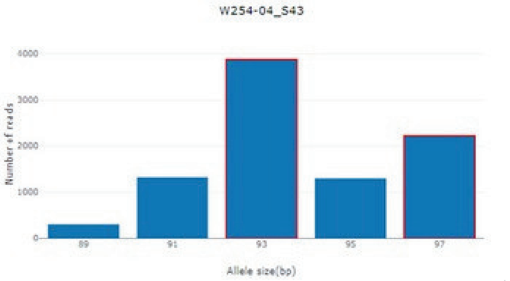

93/97

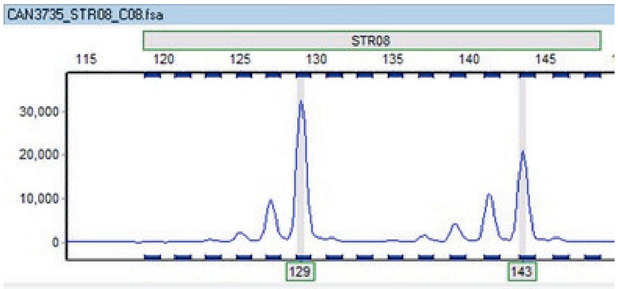

129/143

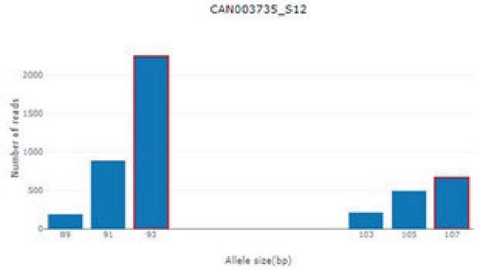

93/107

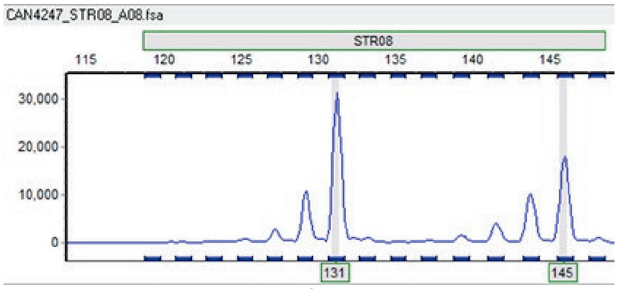

131/145

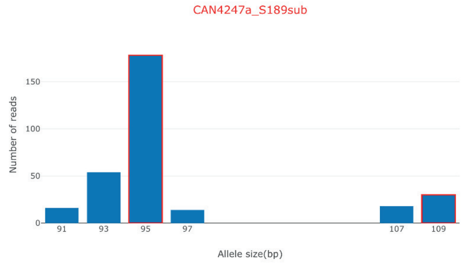

95/109

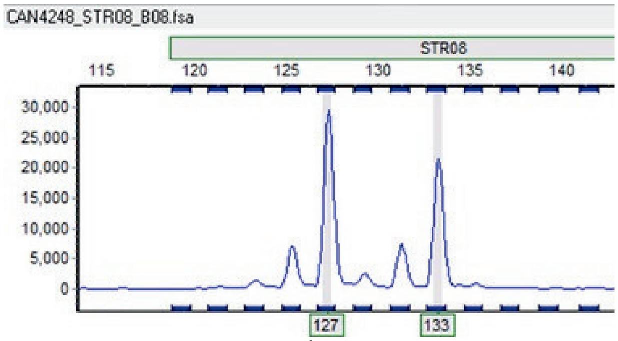

127/133

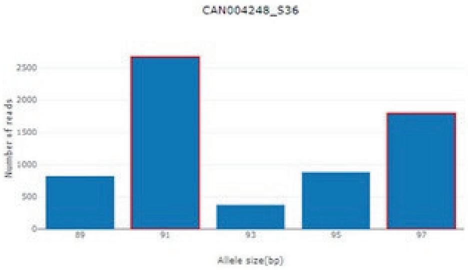

91/97

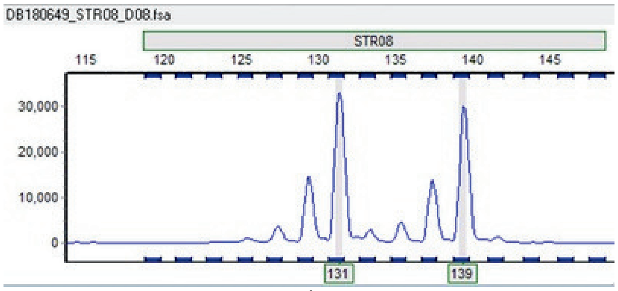

131/139

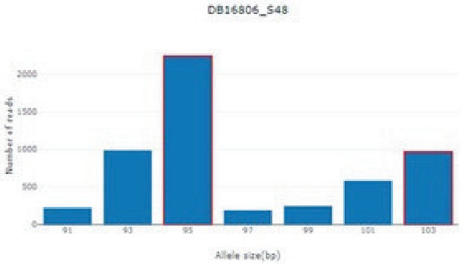

95/103

Capillary Electrophoresis - GeneMarker

Genotyping By Sequencing - SatAnalyzer

CfamSTR009 - Allele size difference - 40 bp

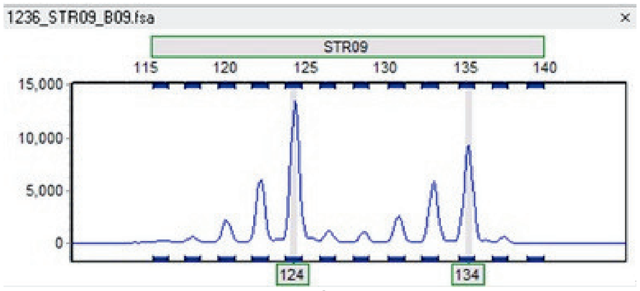

124/134

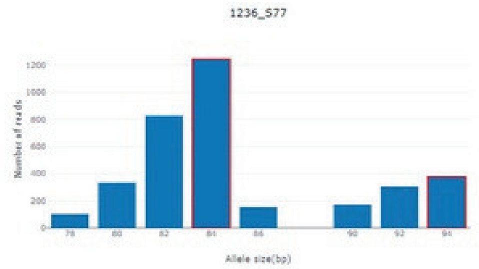

84/94

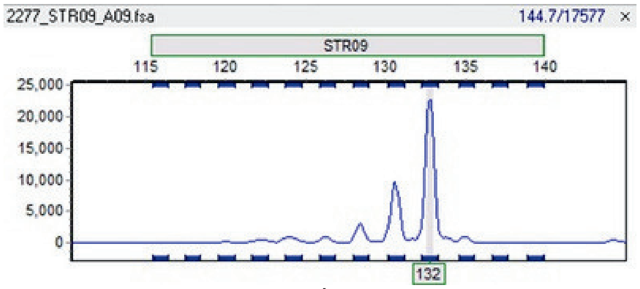

132/132

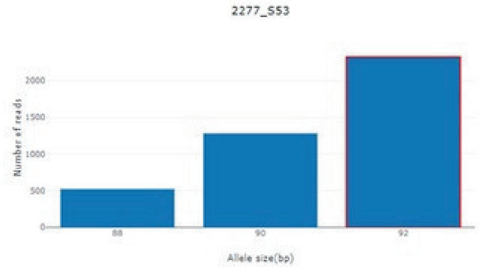

92/92

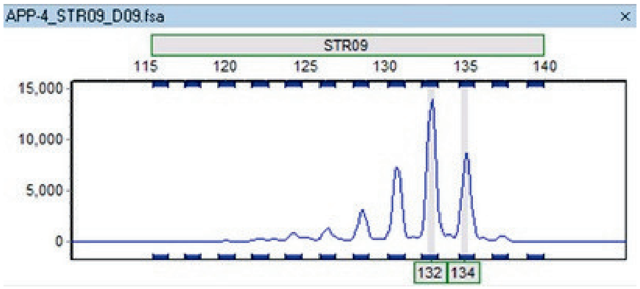

132/134

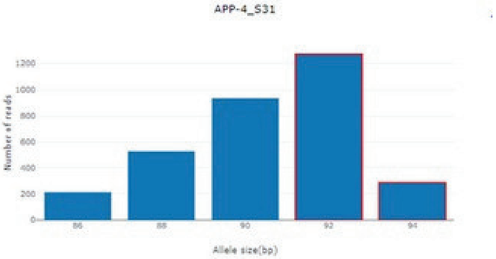

92/94

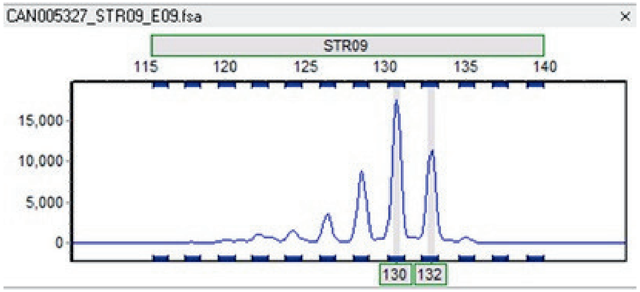

130/132

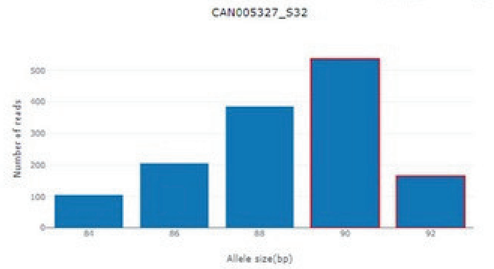

90/92

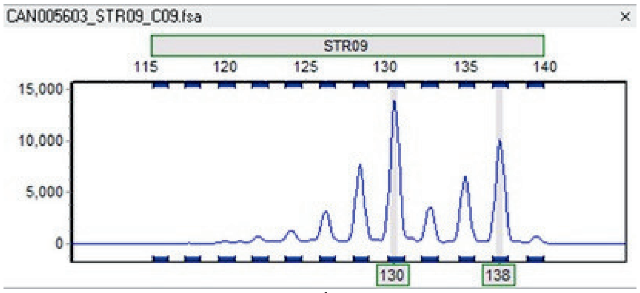

130/138

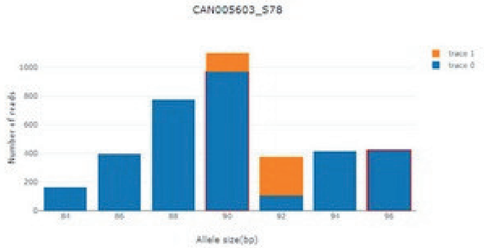

90/96

Capillary Electrophoresis - GeneMarker

Genotyping By Sequencing - SatAnalyzer

CfamSTR009 - Allele size difference - 40 bp

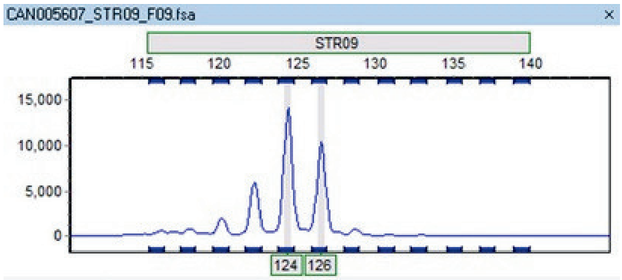

124/126

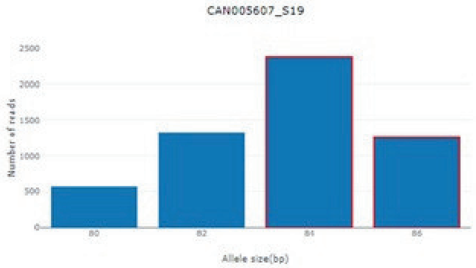

84/86

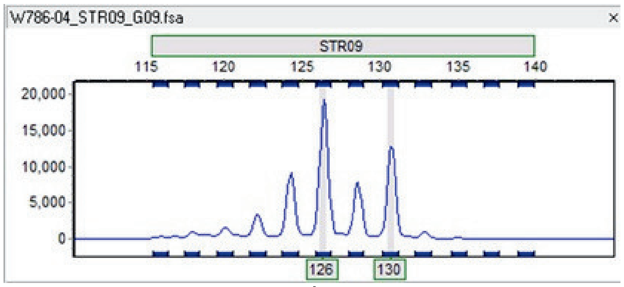

126/130

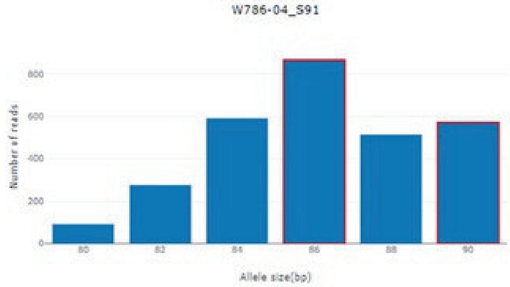

86/90

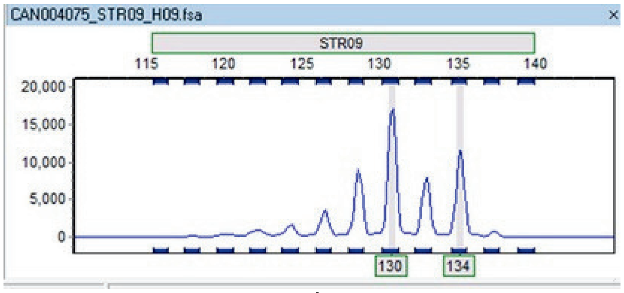

130/134

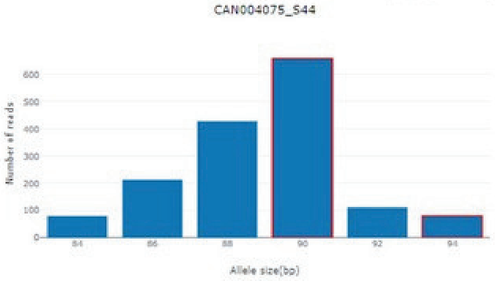

90/94

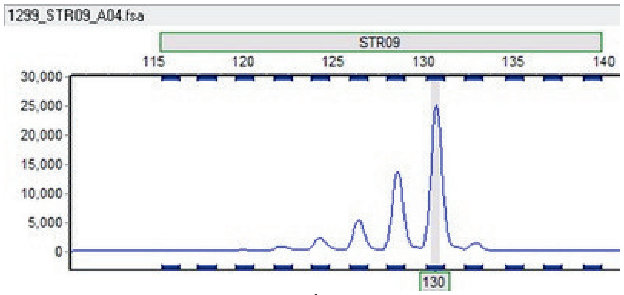

130/130

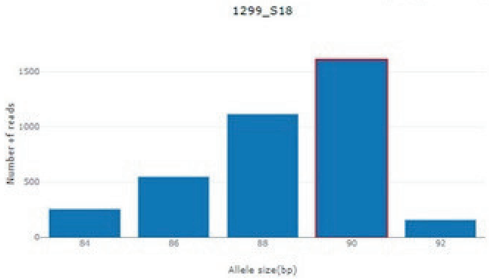

90/90

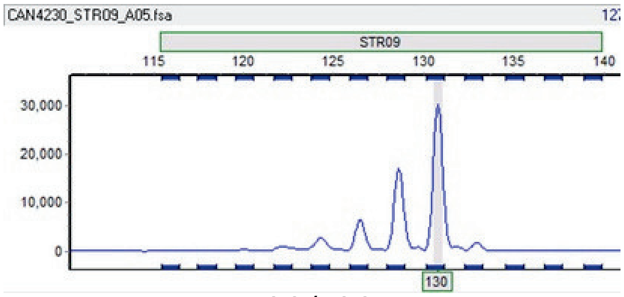

130/130

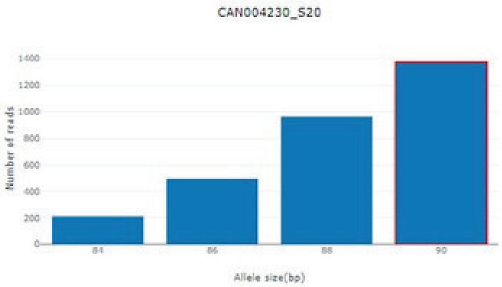

90/90

Capillary Electrophoresis - GeneMarker

Genotyping By Sequencing - SatAnalyzer

CfamSTR009 - Allele size difference - 40 bp

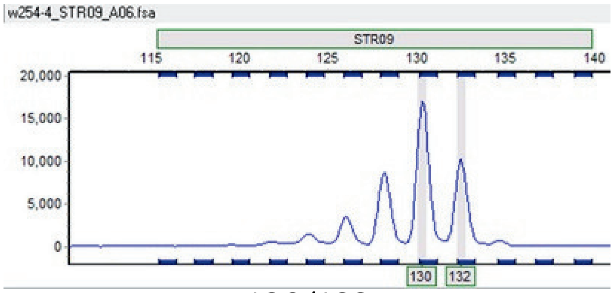

130/132

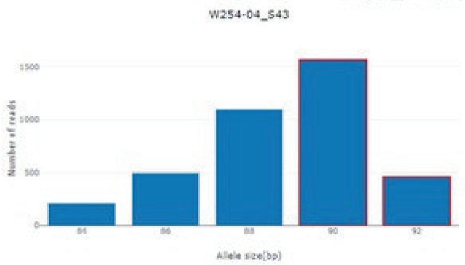

90/92

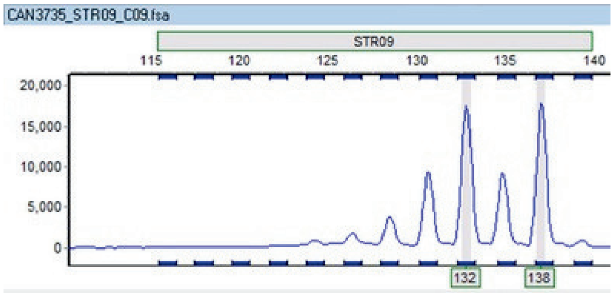

132/138

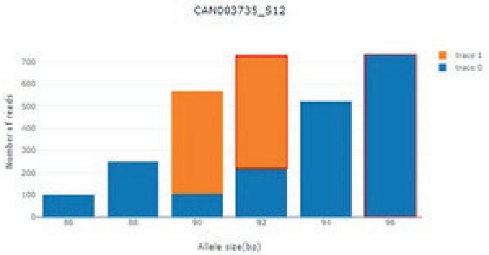

92/98

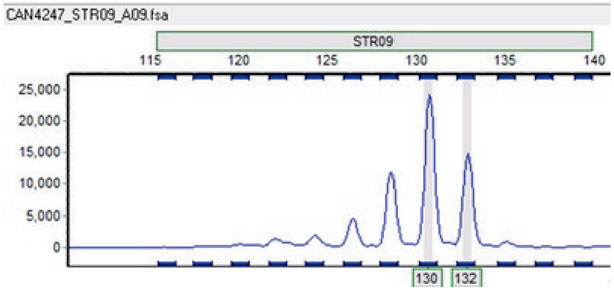

130/132

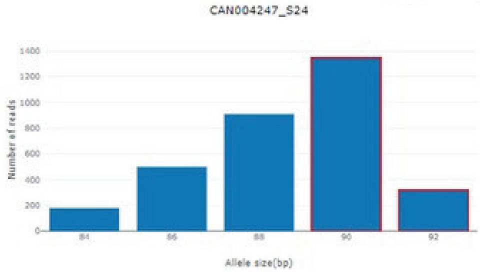

90/92

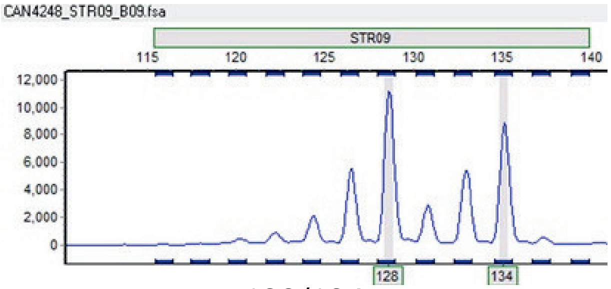

128/134

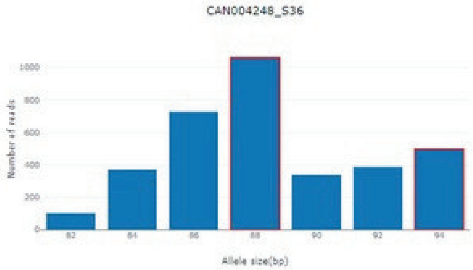

88/94

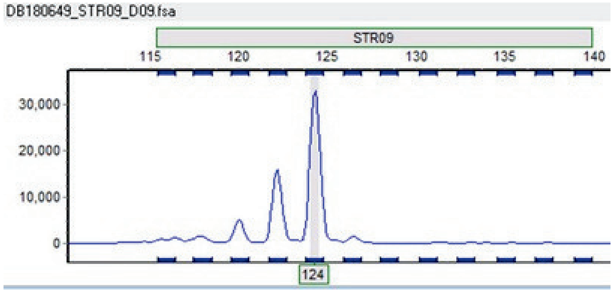

124/124

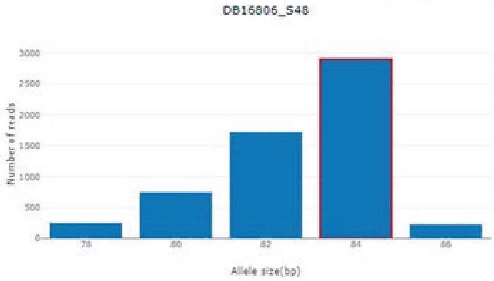

84/84

Capillary Electrophoresis - GeneMarker

Genotyping By Sequencing - SatAnalyzer

CfamSTR010 - Allele size difference - 44 bp

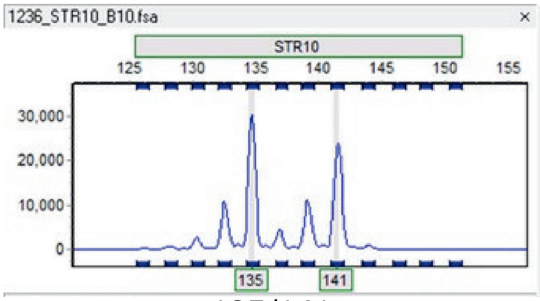

135/141

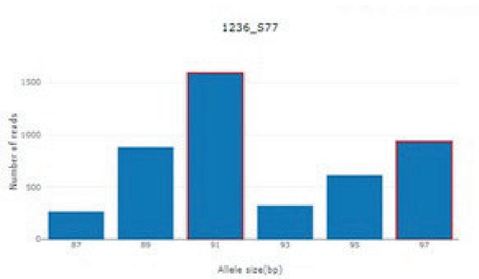

91/97

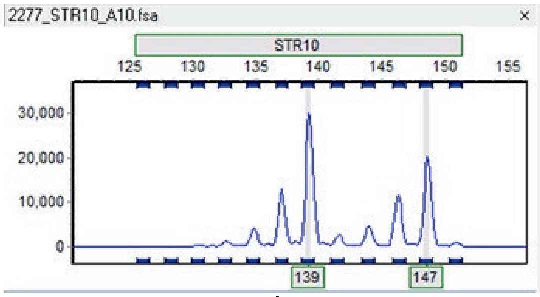

139/147

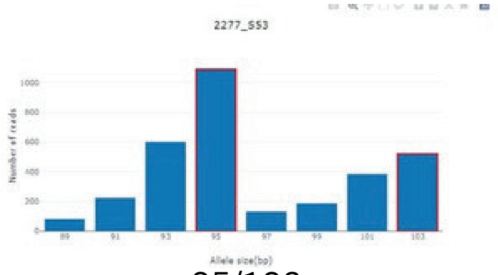

95/103

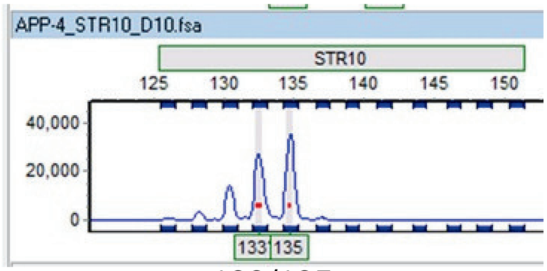

133/135

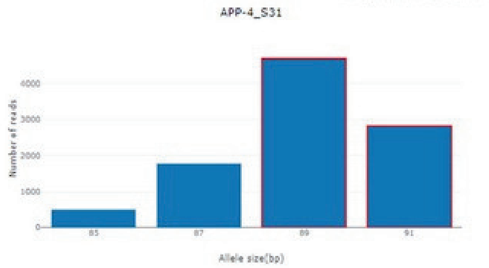

89/91

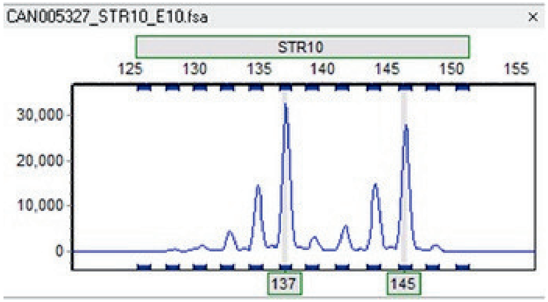

137/145

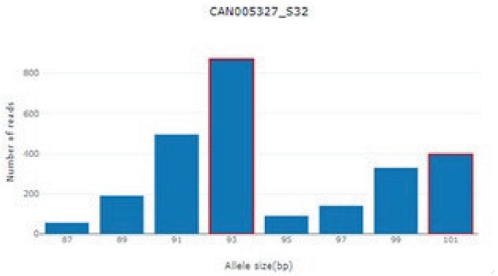

93/101

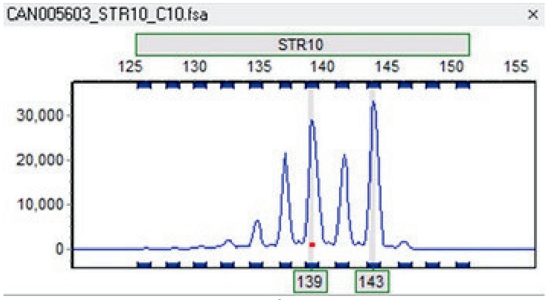

139/143

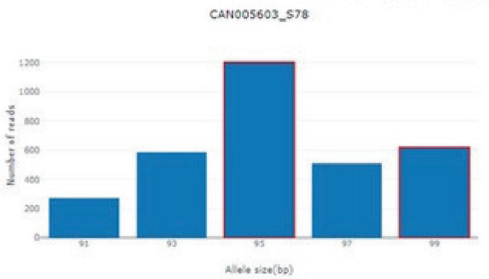

95/99

Capillary Electrophoresis - GeneMarker

Genotyping By Sequencing - SatAnalyzer

CfamSTR010 - Allele size difference - 44 bp

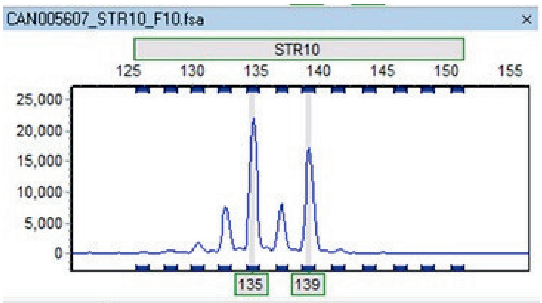

135/139

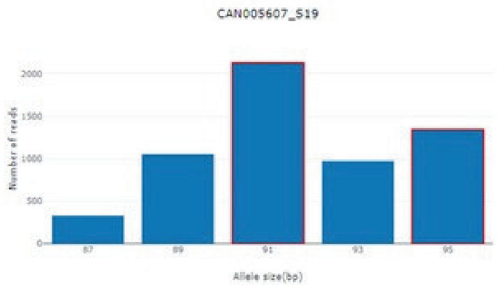

91/95

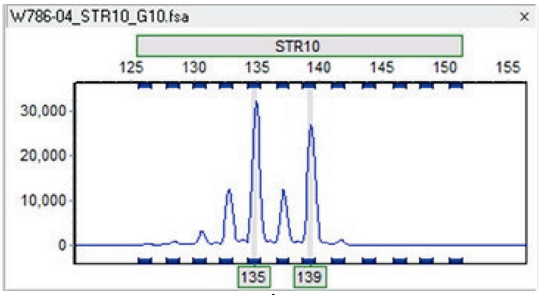

135/139

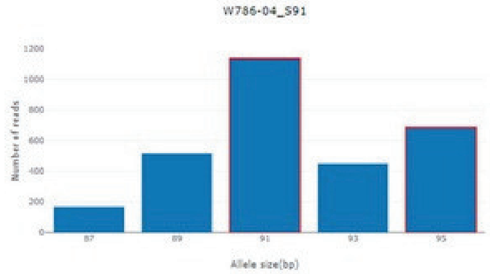

91/95

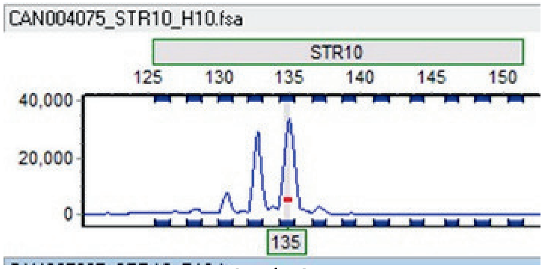

135/135

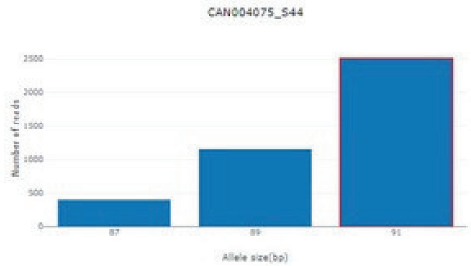

91/91

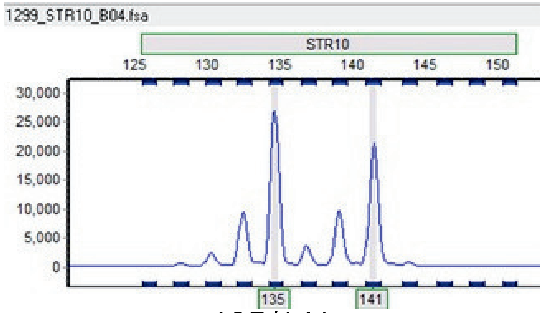

135/141

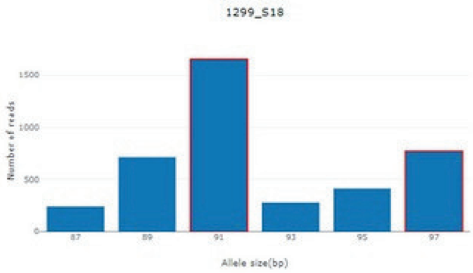

91/97

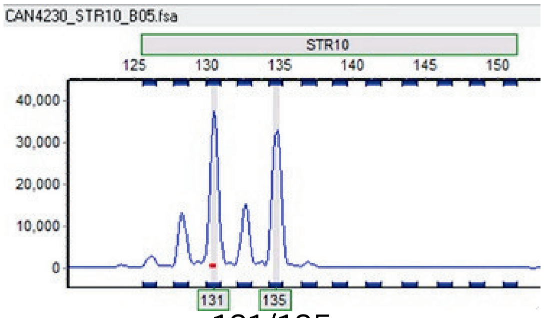

131/135

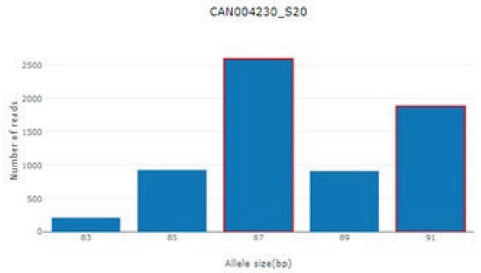

87/91

Capillary Electrophoresis - GeneMarker

Genotyping By Sequencing - SatAnalyzer

CfamSTR010 - Allele size difference - 44 bp

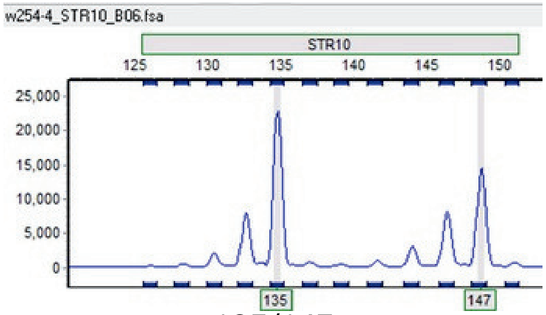

135/147

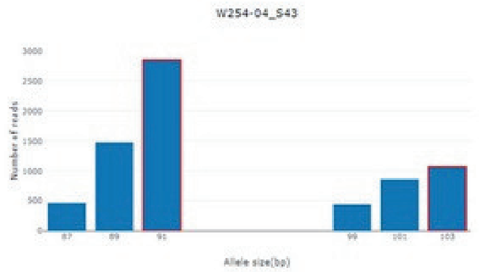

91/103

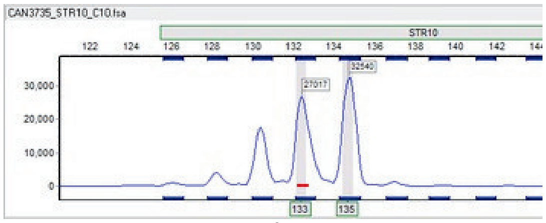

133/135

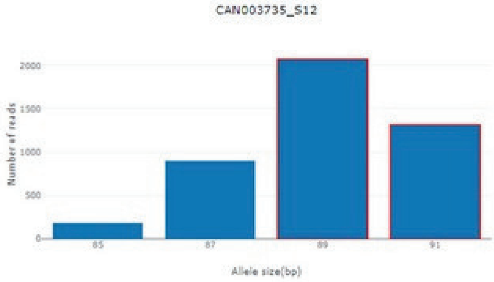

89/91

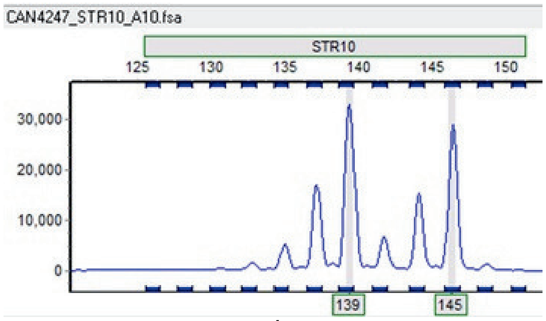

139/145

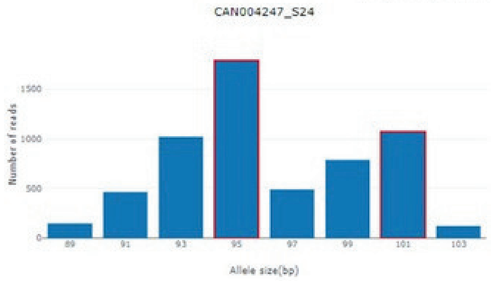

95/101

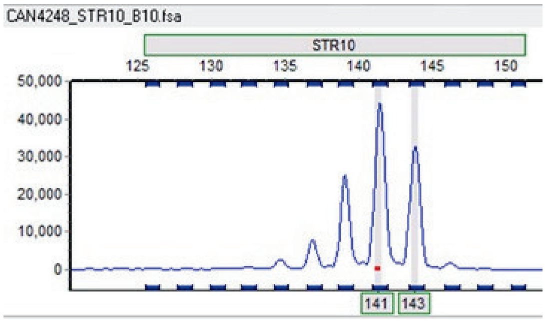

141/143

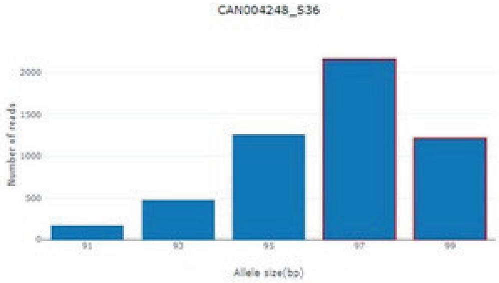

97/99

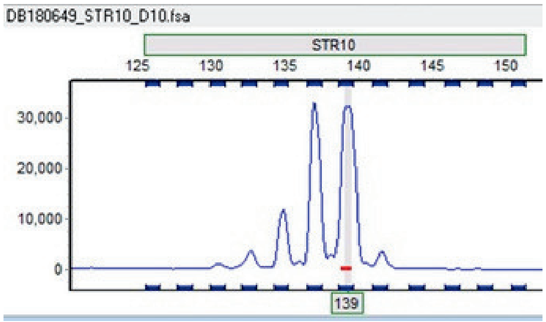

139/139

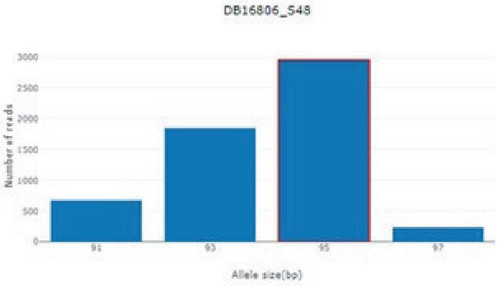

95/95

Capillary Electrophoresis - GeneMarker

Genotyping By Sequencing - SatAnalyzer

CfamSTR011 - Allele size difference - 39 bp

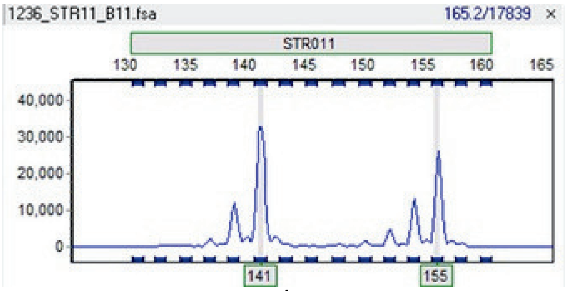

141/155

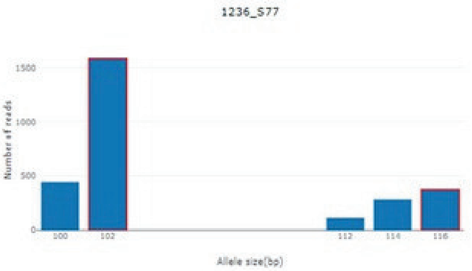

102/116

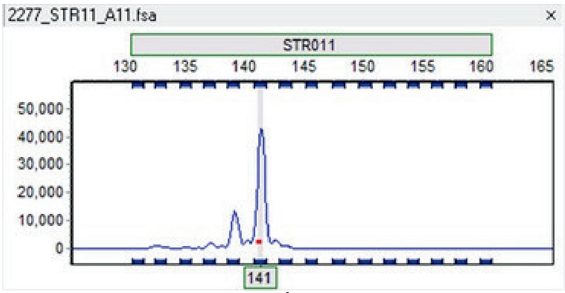

141/141

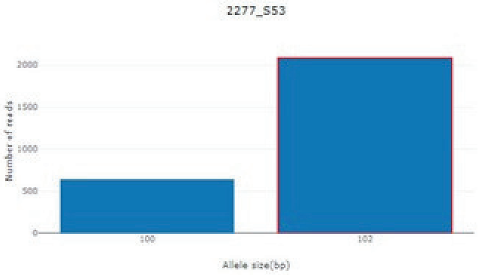

102/102

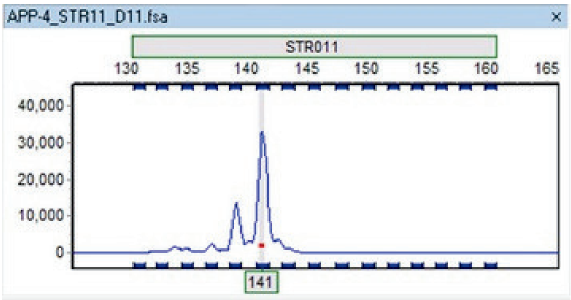

141/141

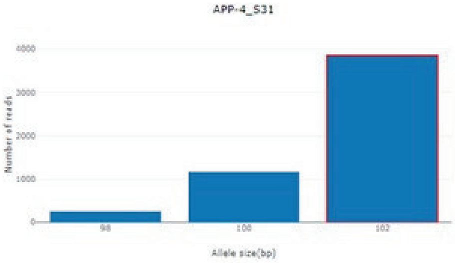

102/102

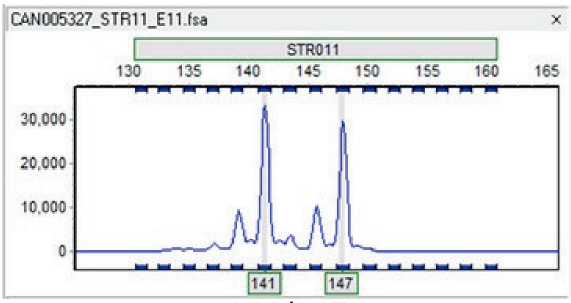

141/147

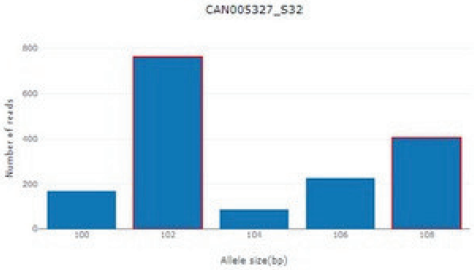

102/108

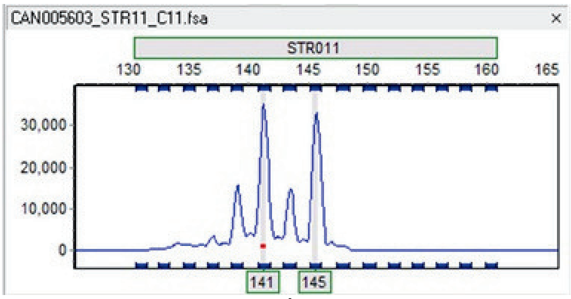

141/145

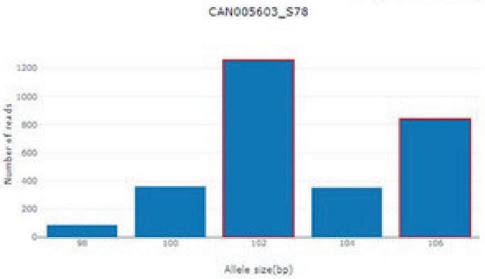

102/106

Capillary Electrophoresis - GeneMarker

Genotyping By Sequencing - SatAnalyzer

CfamSTR011 - Allele size difference - 39 bp

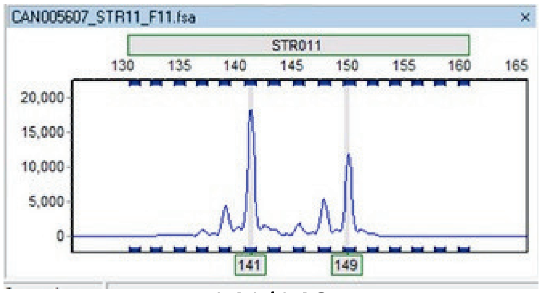

141/149

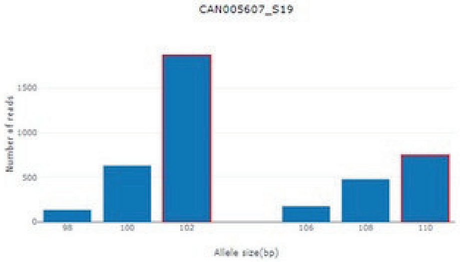

102/110

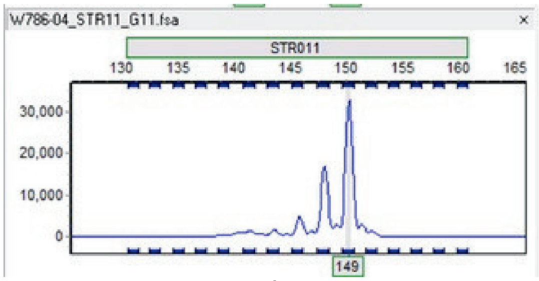

149/149

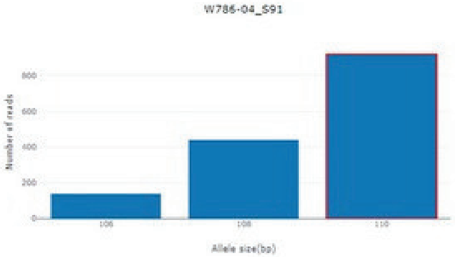

110/110

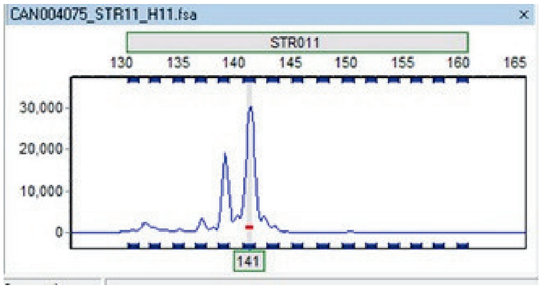

141/141

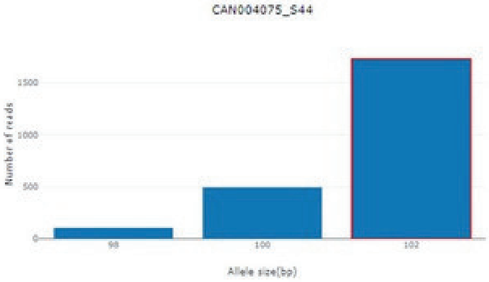

102/102

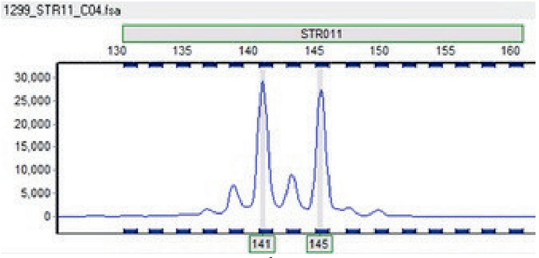

141/145

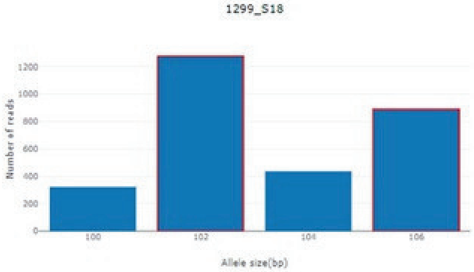

102/106

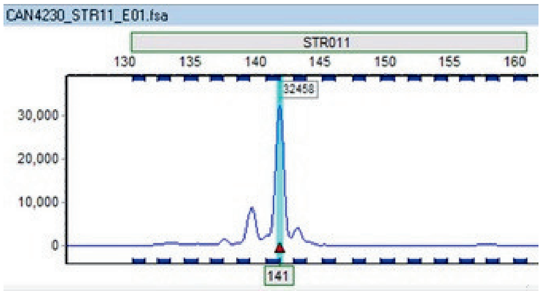

141/141

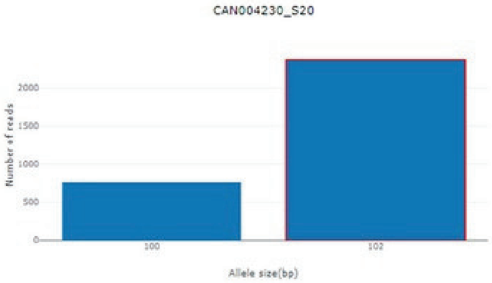

102/102

Capillary Electrophoresis - GeneMarker

Genotyping By Sequencing - SatAnalyzer

CfamSTR011 - Allele size difference - 39 bp

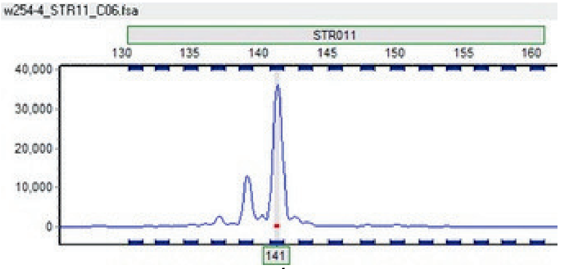

141/141

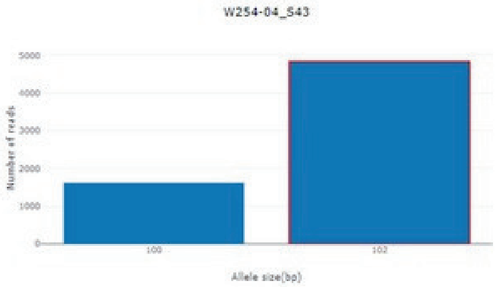

102/102

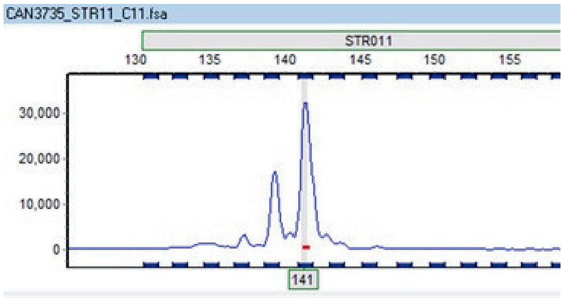

141/141

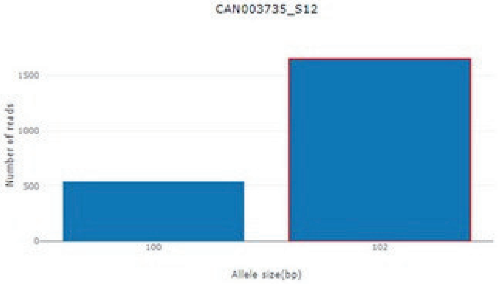

102/102

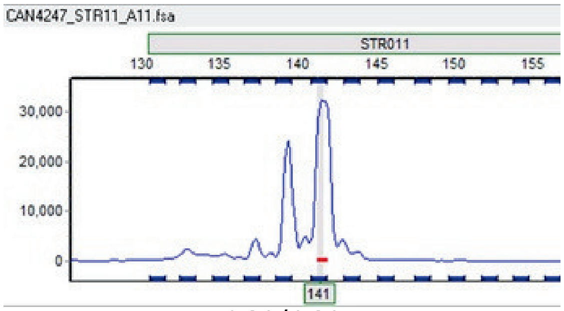

141/141

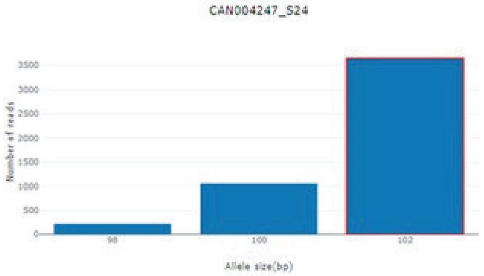

102/102

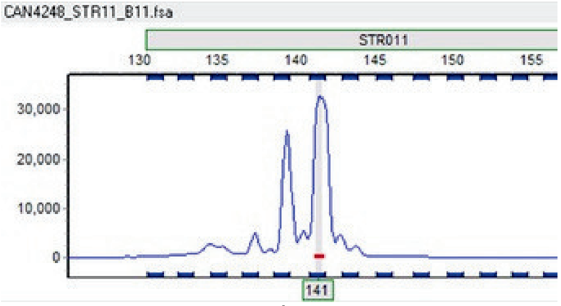

141/141

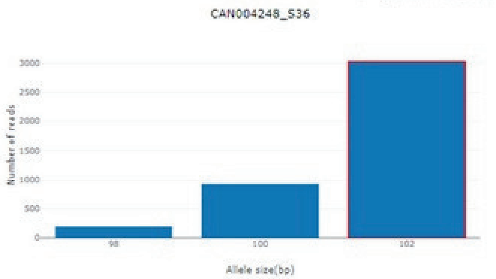

102/102

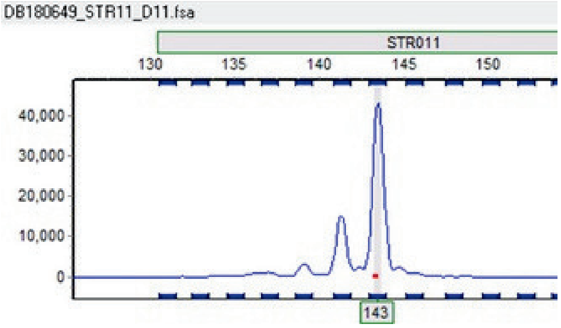

143/143

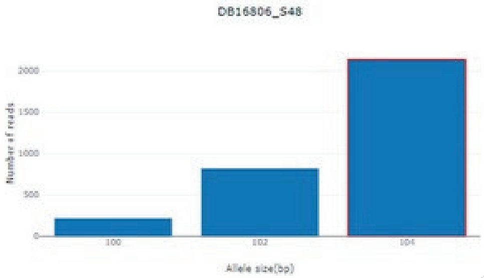

104/104

Capillary Electrophoresis - GeneMarker

Genotyping By Sequencing - SatAnalyzer

CfamSTR012 - Allele size difference - 44 bp

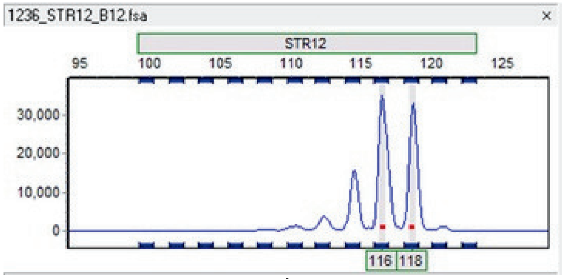

116/118

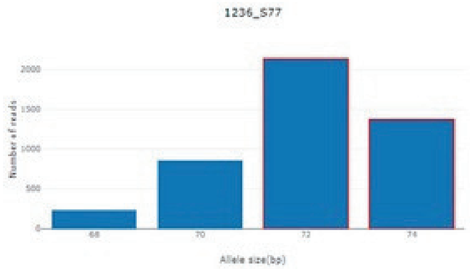

72/74

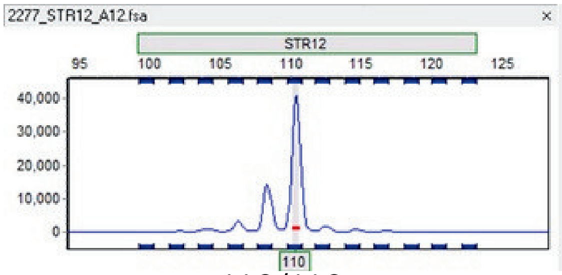

110/110

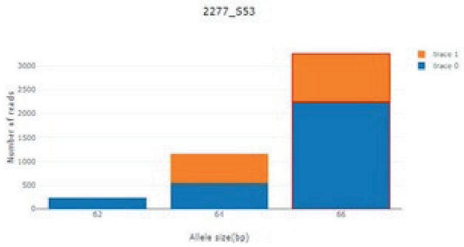

66/66*

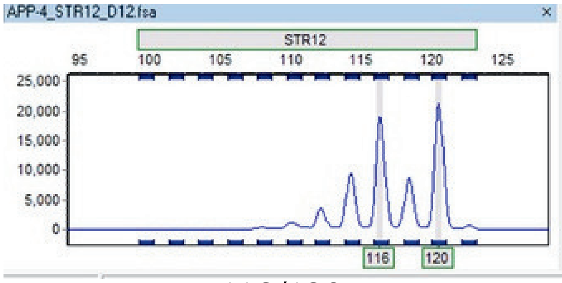

116/120

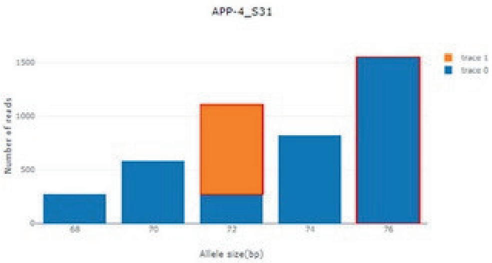

72/76

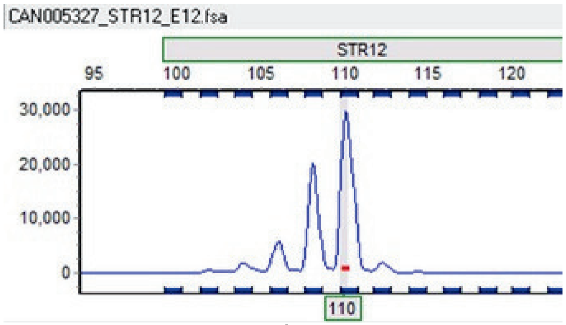

110/110

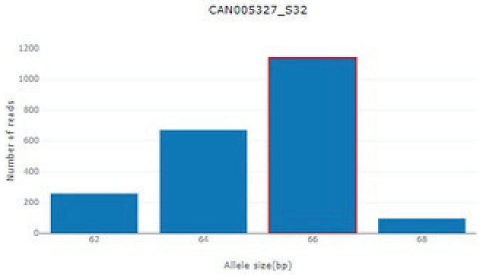

66/66

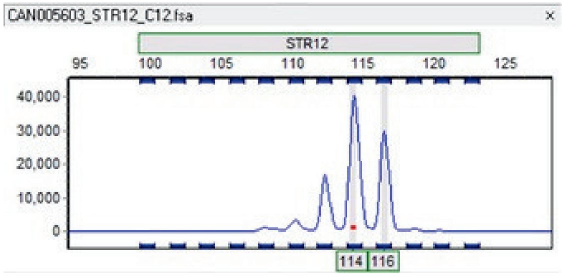

114/116

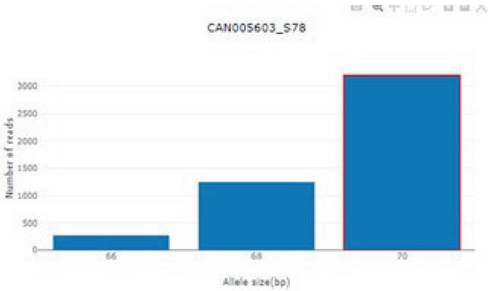

70/70

Capillary Electrophoresis - GeneMarker

Genotyping By Sequencing - SatAnalyzer

CfamSTR012 - Allele size difference - 44 bp

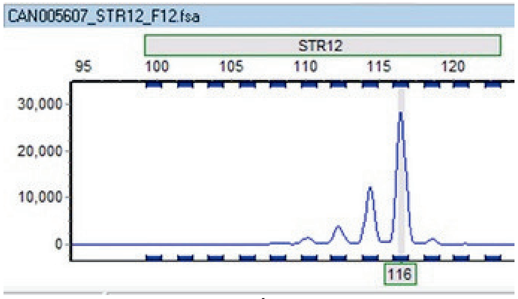

116/116

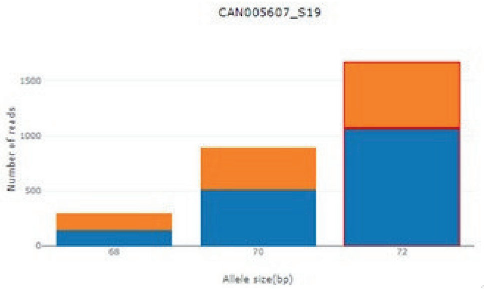

72/72*

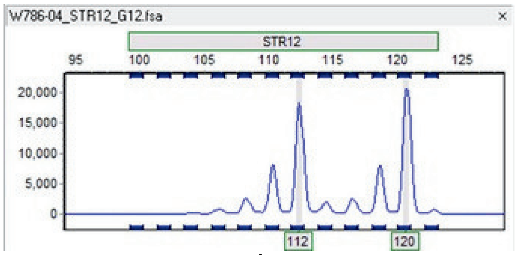

112/120

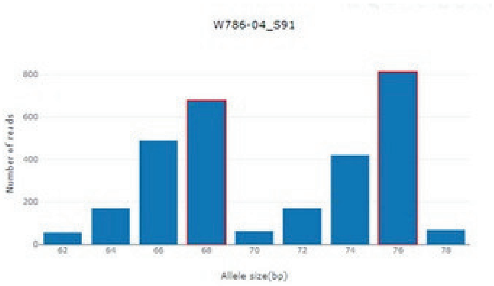

68/76

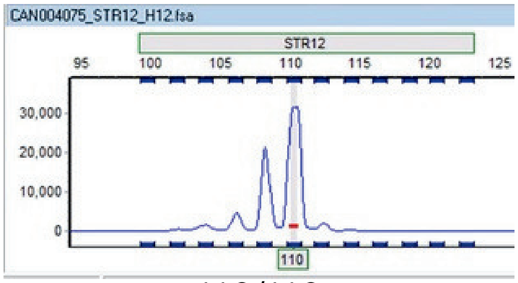

110/110

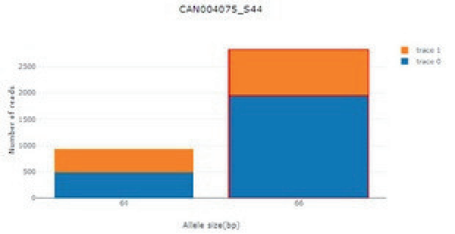

66/66*

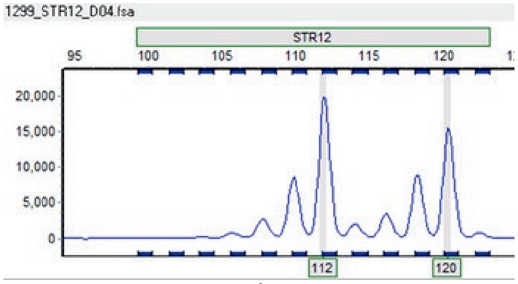

112/120

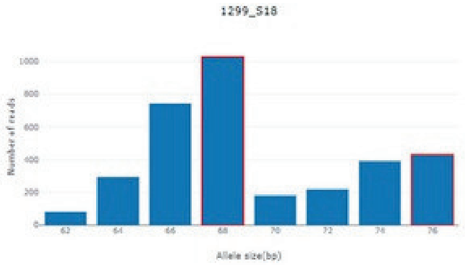

68/76

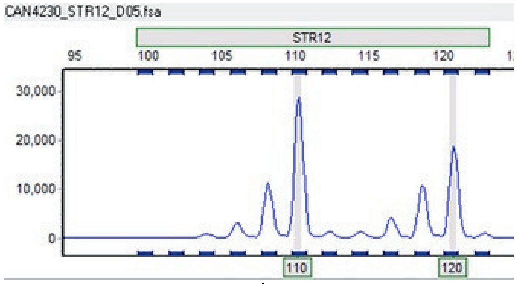

110/120

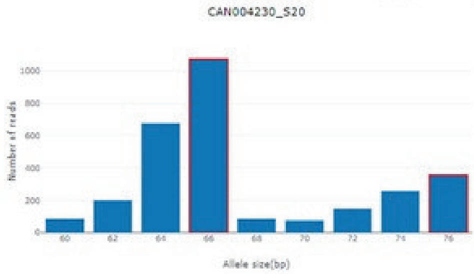

66/76

Capillary Electrophoresis - GeneMarker

Genotyping By Sequencing - SatAnalyzer

CfamSTR012 - Allele size difference - 44 bp

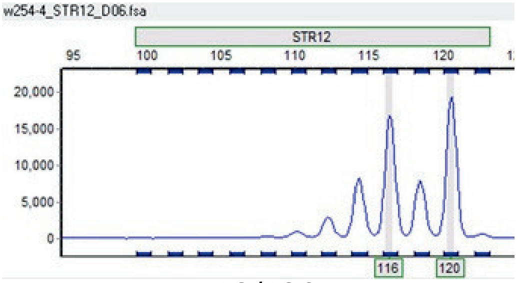

116/120

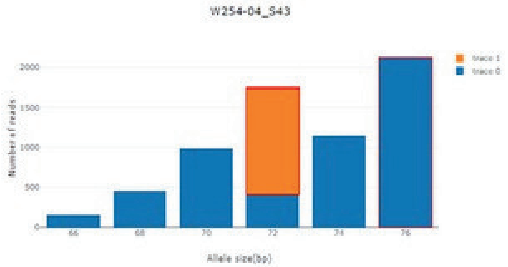

72/76

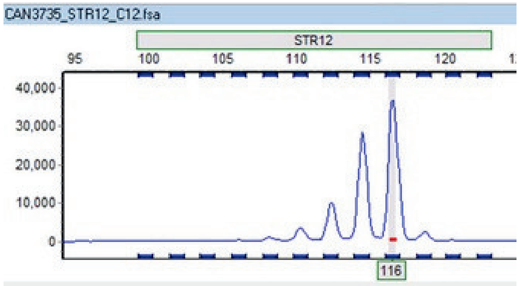

116/116

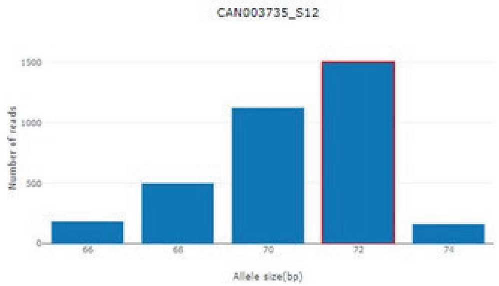

72/72

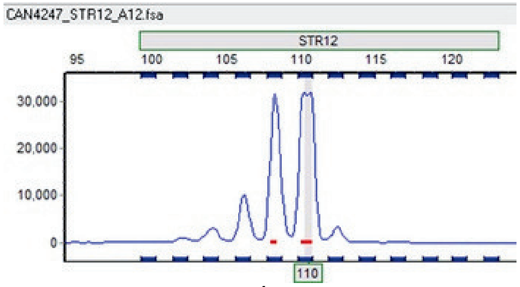

110/110

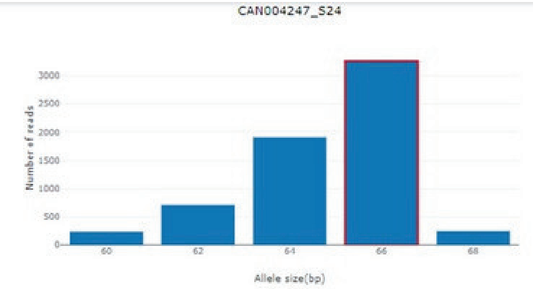

66/66

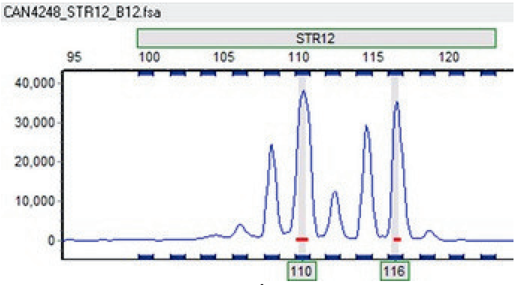

110/116

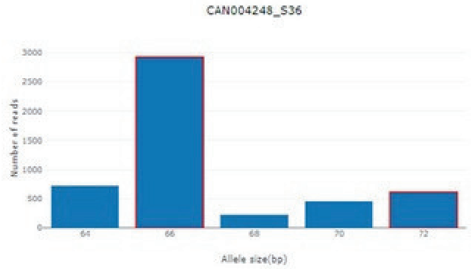

66/72

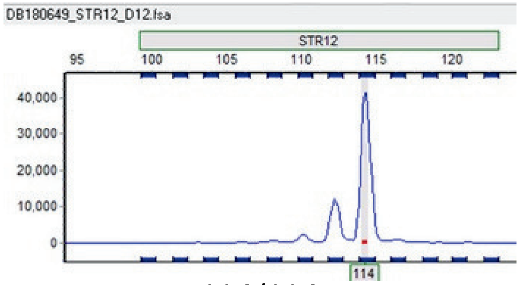

114/114

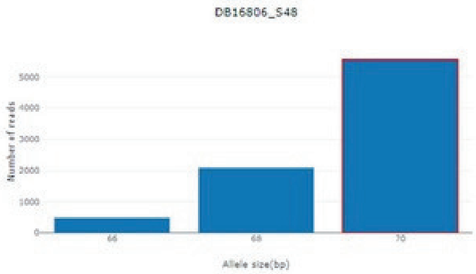

70/70

Capillary Electrophoresis - GeneMarker

Genotyping By Sequencing - SatAnalyzer

CfamSTR013 - Allele size difference - 34 bp

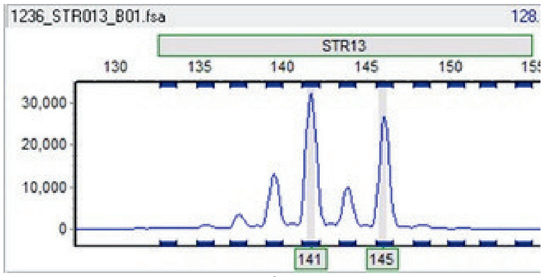

141/145

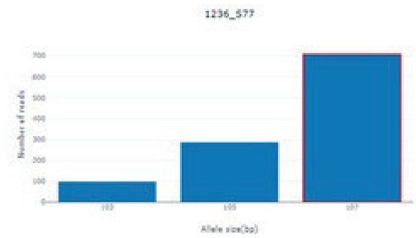

107/107

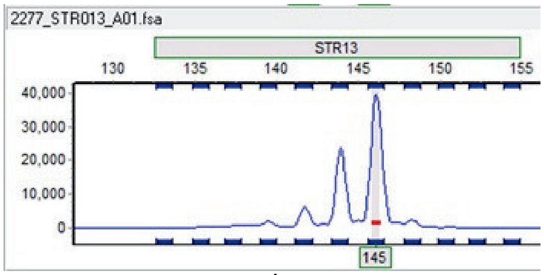

145/145

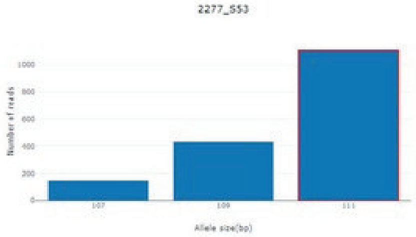

111/111

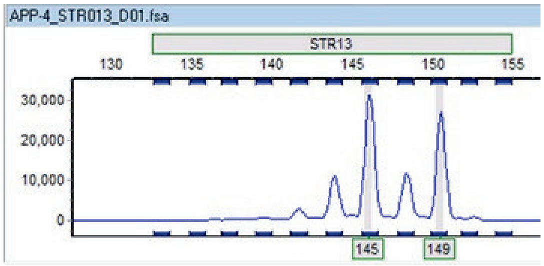

145/149

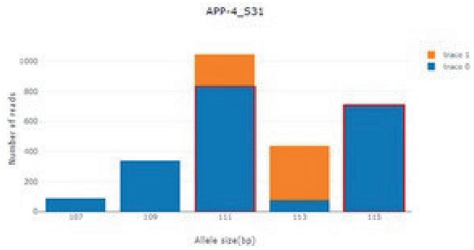

111/115

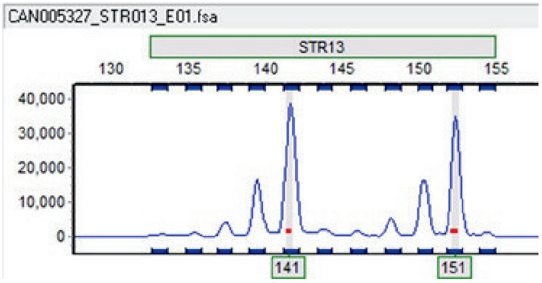

141/151

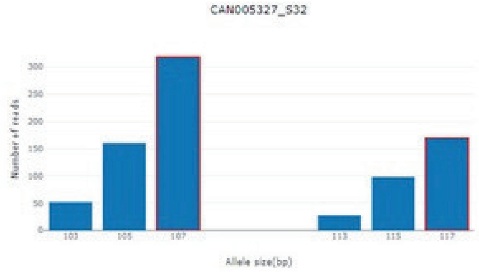

107/117

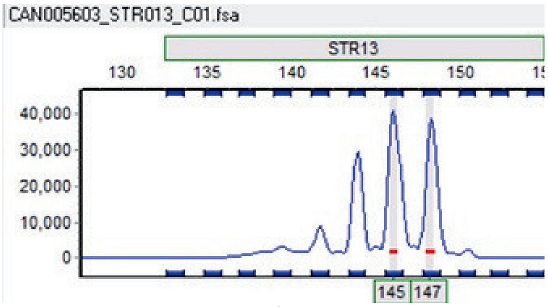

145/147

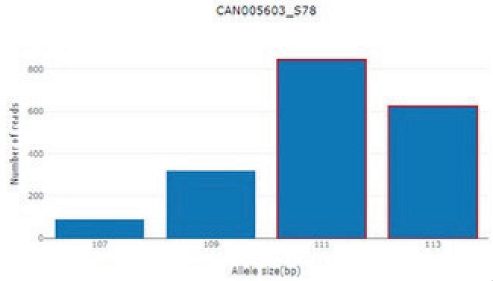

111/113

Capillary Electrophoresis - GeneMarker

Genotyping By Sequencing - SatAnalyzer

CfamSTR013 - Allele size difference - 34 bp

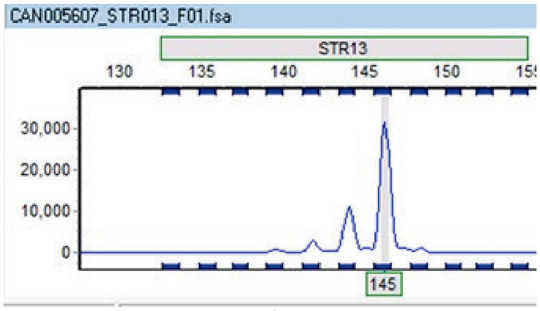

145/145

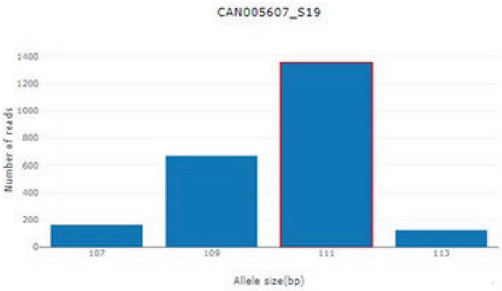

111/111

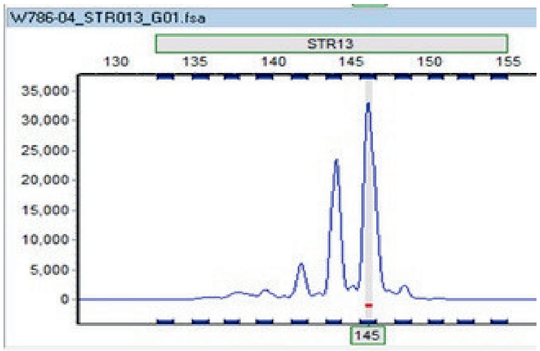

145/145

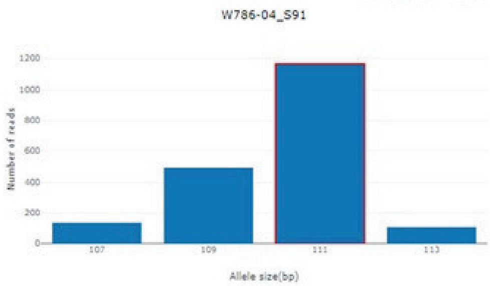

111/111

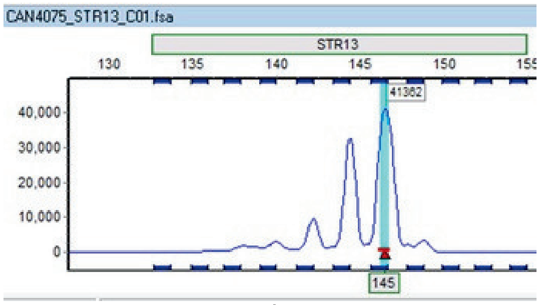

145/145

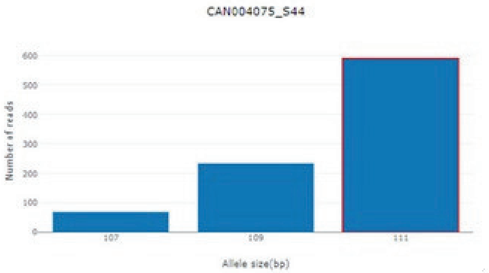

111/111

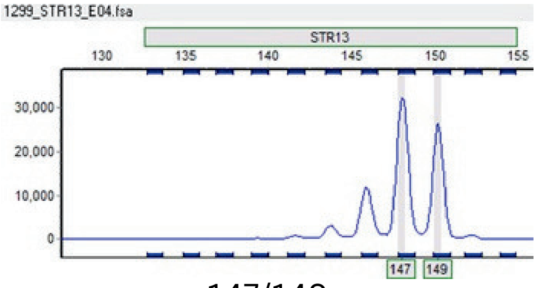

147/149

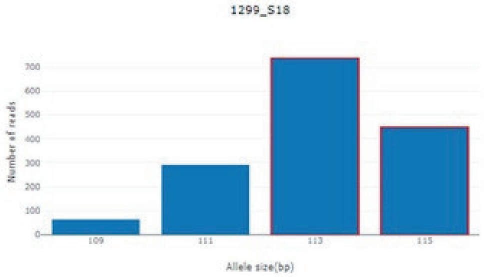

113/115

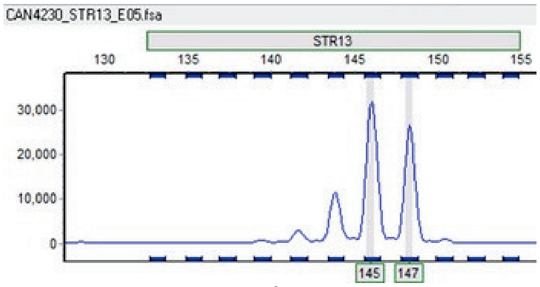

145/147

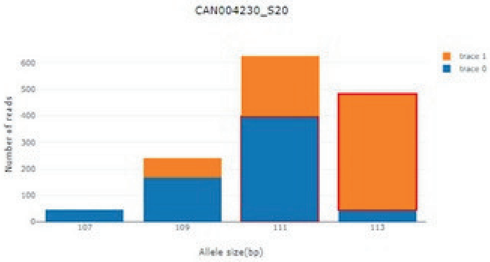

111/113

Capillary Electrophoresis - GeneMarker

Genotyping By Sequencing - SatAnalyzer

CfamSTR013 - Allele size difference - 34 bp

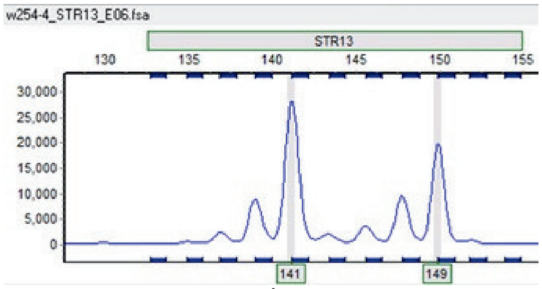

141/149

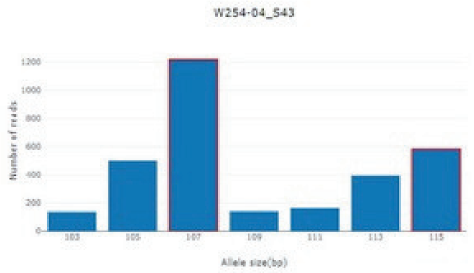

107/115

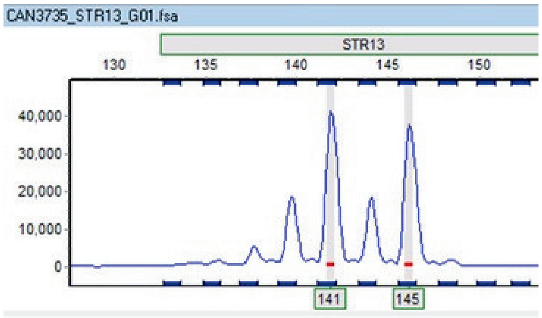

141/145

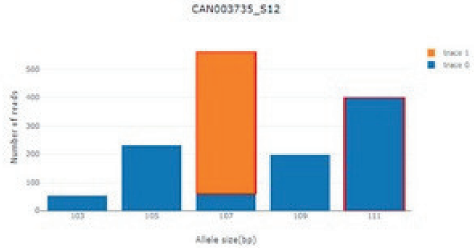

107/111

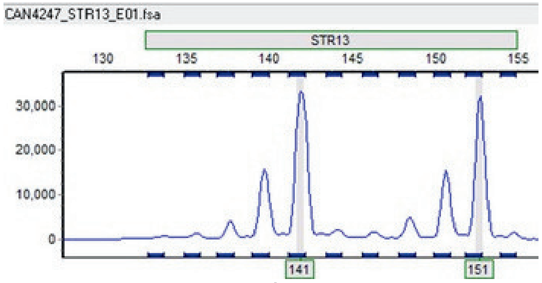

141/151

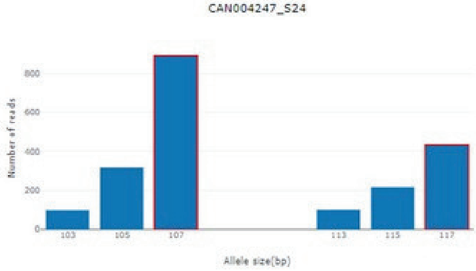

107/117

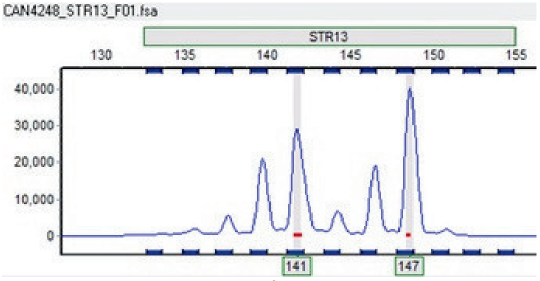

141/147

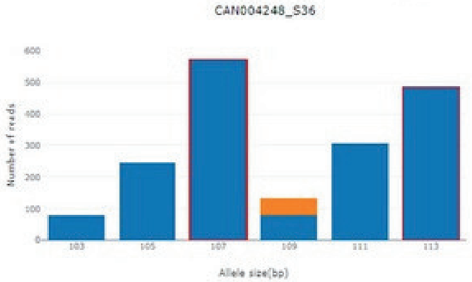

107/113

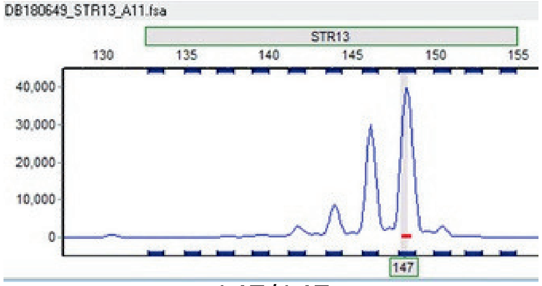

147/147

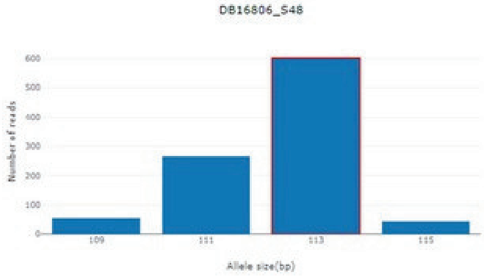

113/113

Capillary Electrophoresis - GeneMarker

Genotyping By Sequencing - SatAnalyzer

CfamSTR014 - Allele size difference - 41 bp

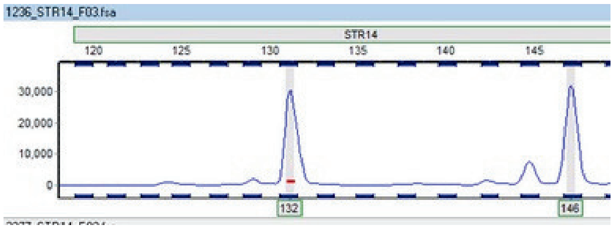

132/146

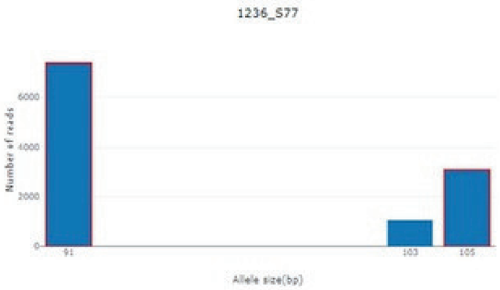

91/105

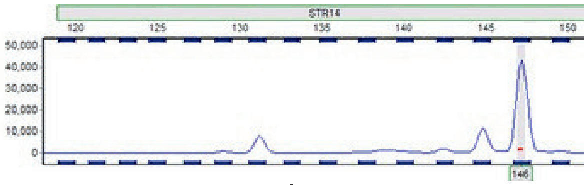

146/146

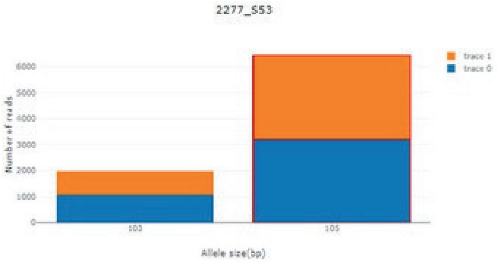

105/105*

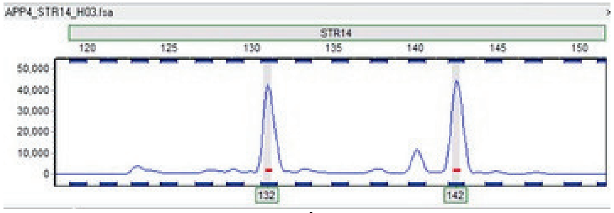

132/142

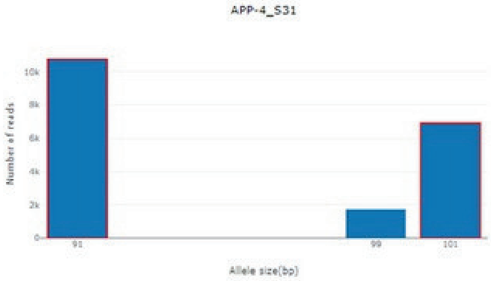

91/101

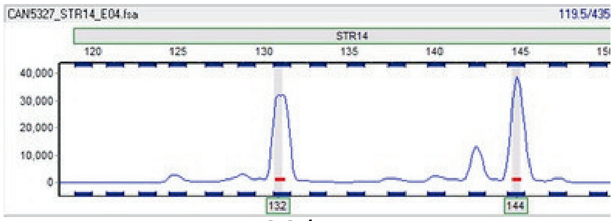

132/144

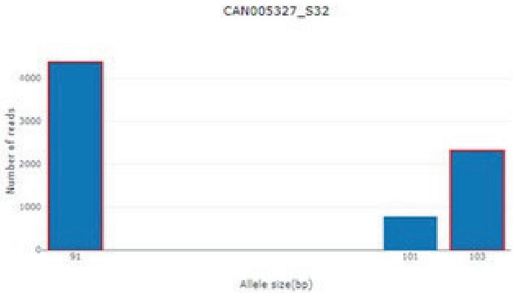

91/103

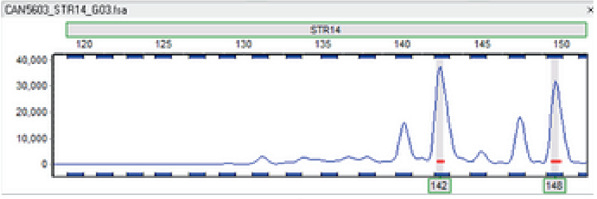

142/148

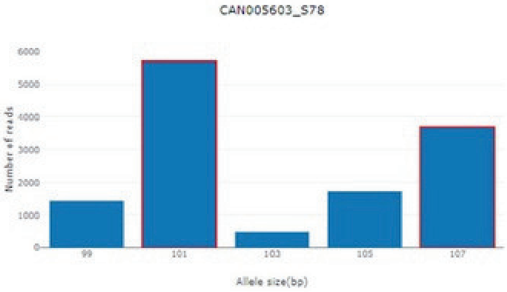

101/107

Capillary Electrophoresis - GeneMarker

Genotyping By Sequencing - SatAnalyzer

CfamSTR014 - Allele size difference - 41 bp

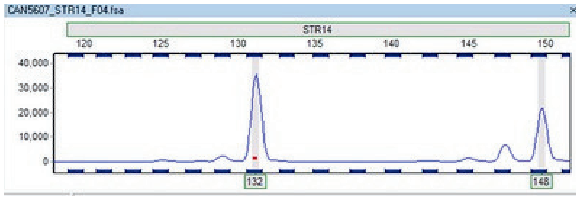

132/148

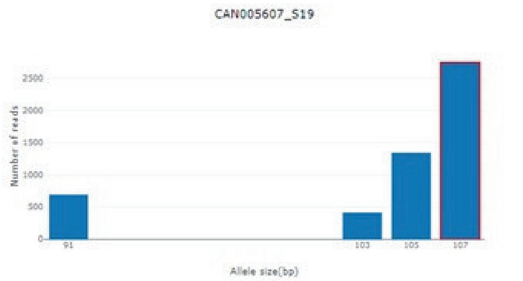

91/107

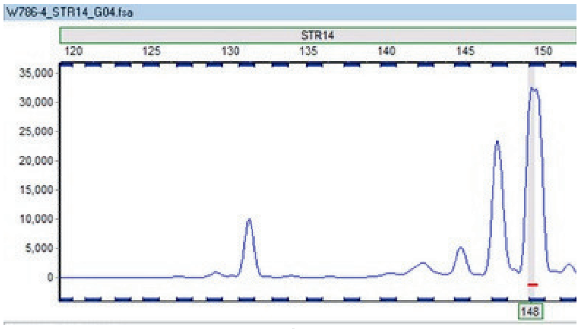

148/148

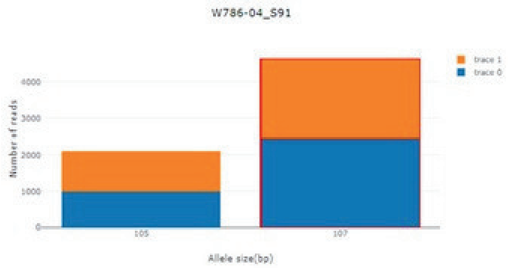

107/107*

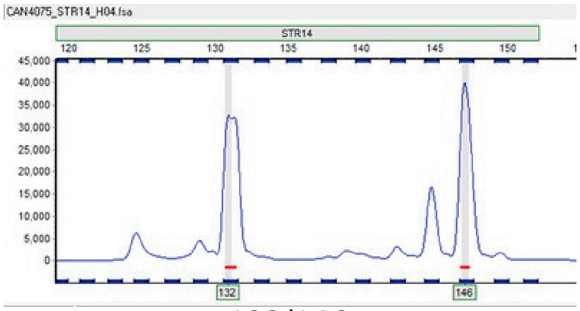

132/146

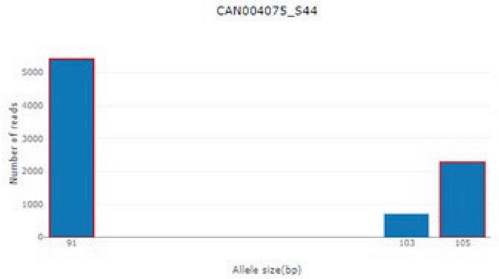

91/105

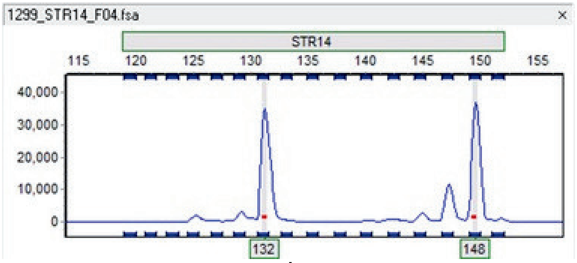

132/148

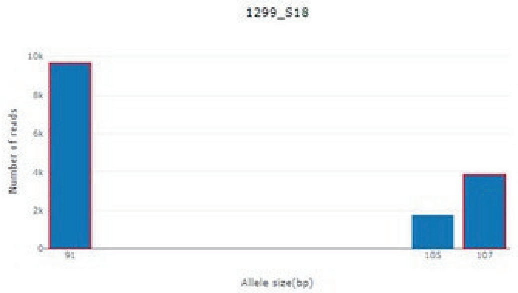

91/107

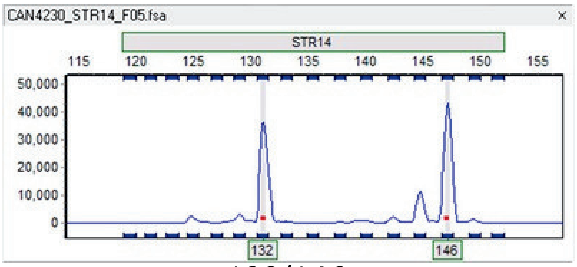

132/146

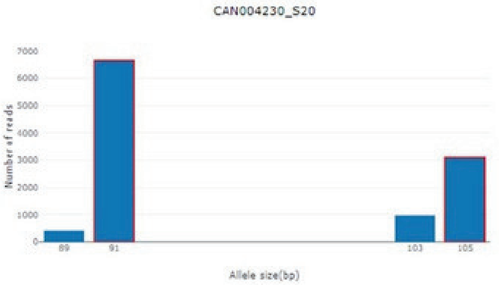

91/105

Capillary Electrophoresis - GeneMarker | Genotyping By Sequencing - SatAnalyzer

CfamSTR014 - Allele size difference - 41 bp

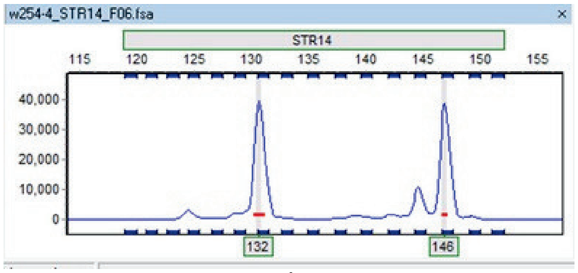

132/146

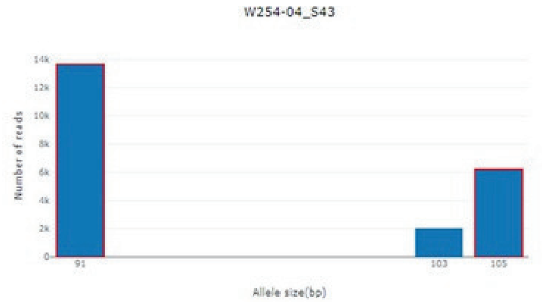

91/105

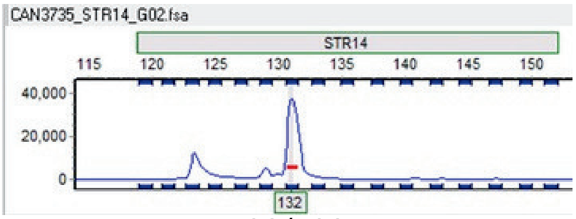

132/132

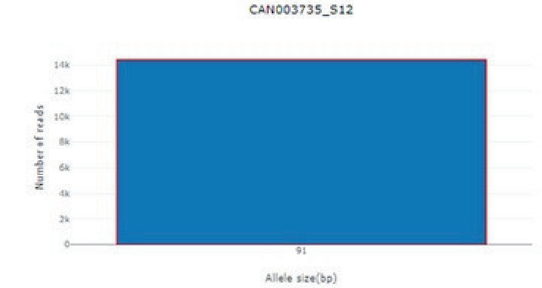

91/91

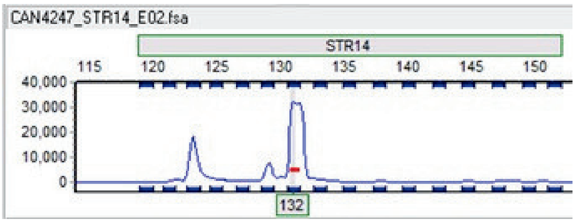

132/132

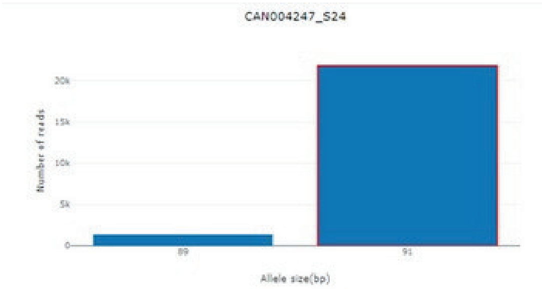

91/91

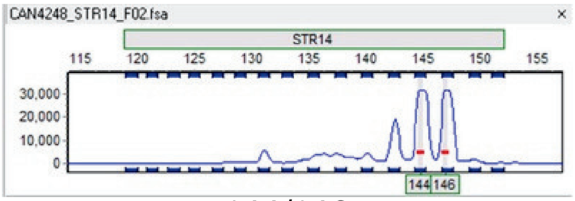

144/146

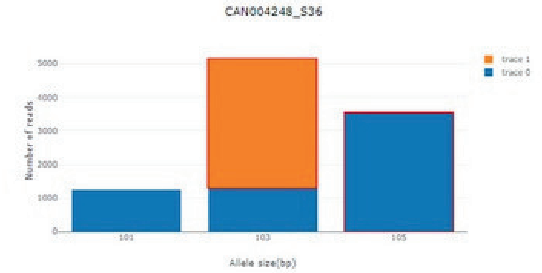

103/105

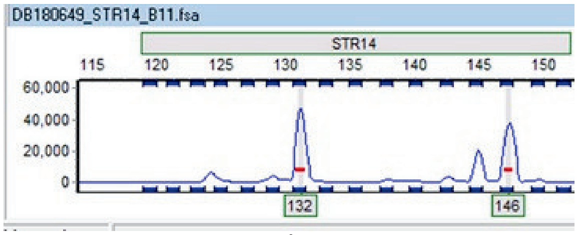

132/146

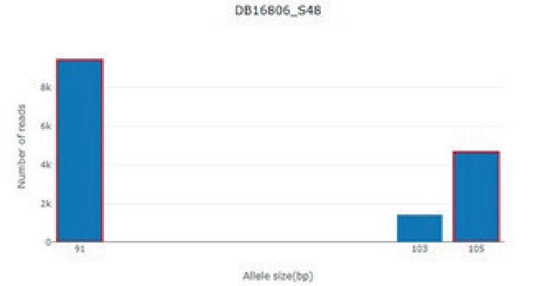

91/105

Capillary Electrophoresis - GeneMarker

Genotyping By Sequencing - SatAnalyzer

CfamSTR015 - Allele size difference - 34 bp

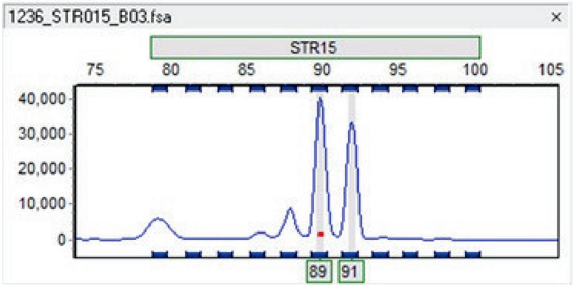

89/91

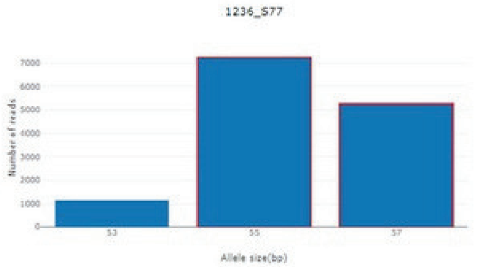

55/57

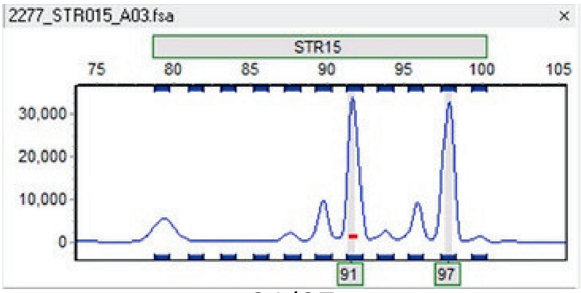

91/97

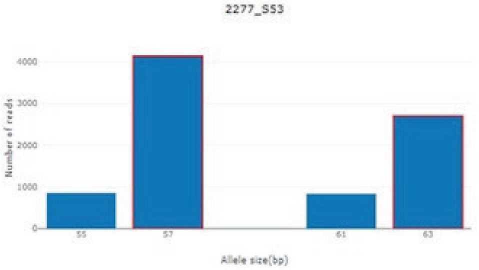

57/63

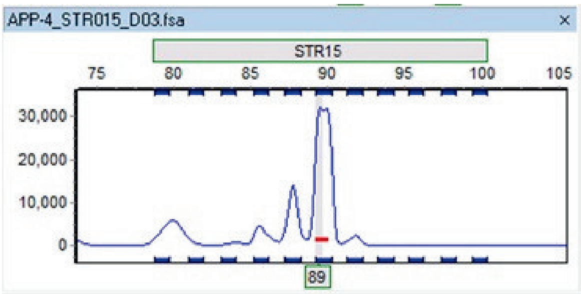

89/89

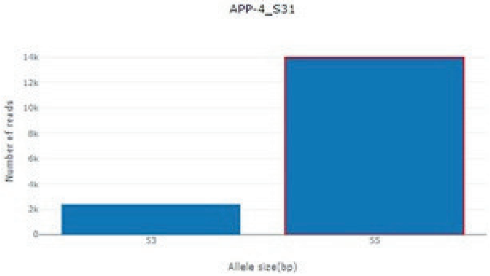

55/55

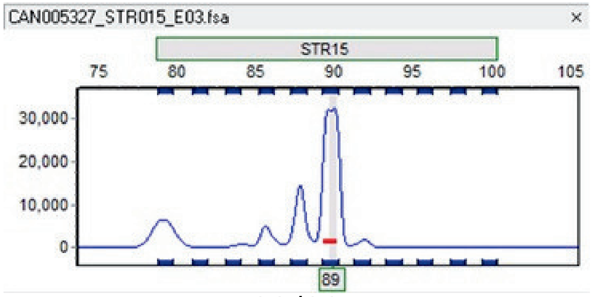

89/89

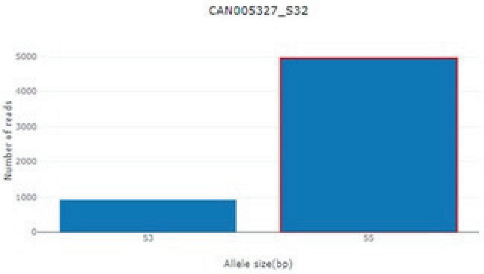

55/55

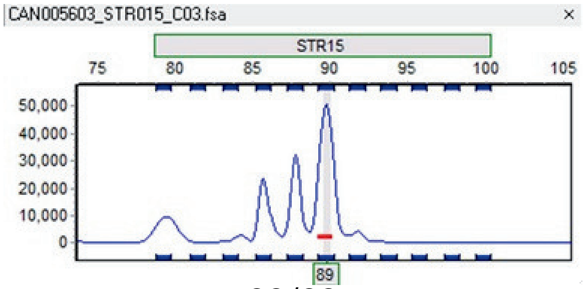

89/89

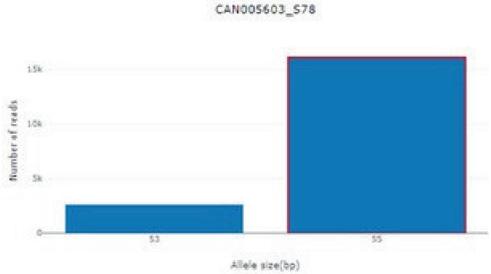

55/55

Capillary Electrophoresis - GeneMarker

Genotyping By Sequencing - SatAnalyzer

CfamSTR015 - Allele size difference - 34 bp

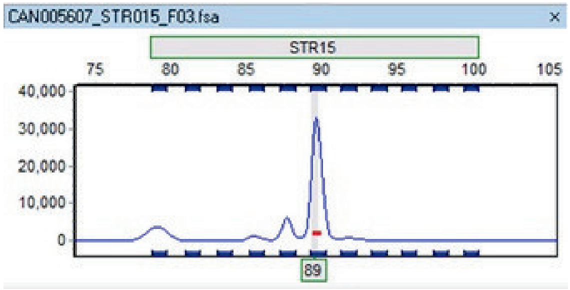

89/89

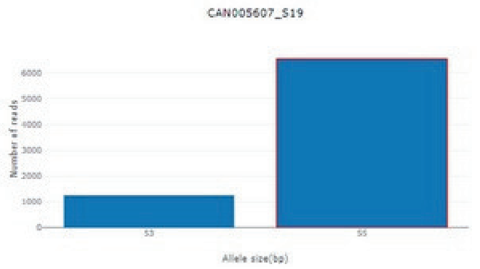

55/55

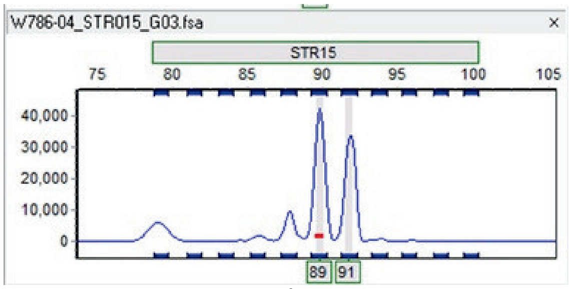

89/91

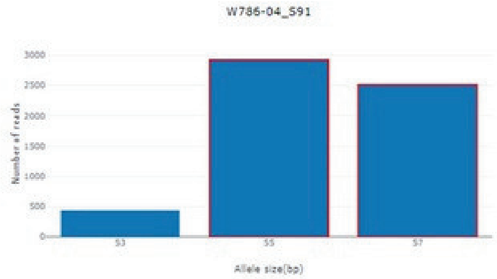

55/57

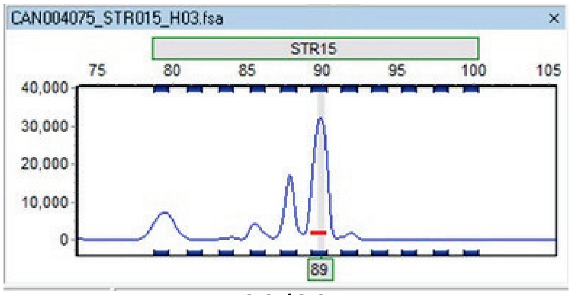

89/89

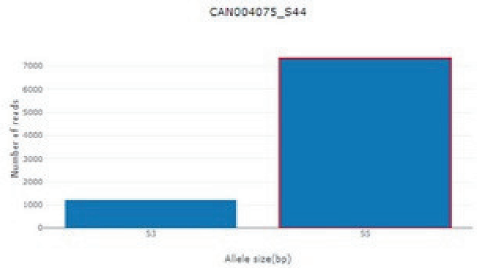

55/55

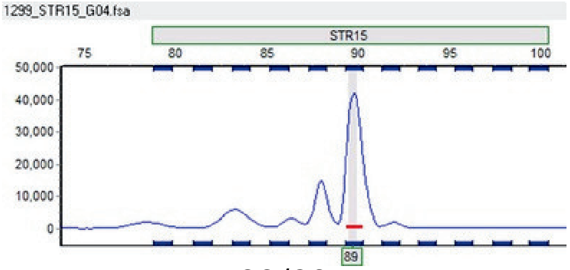

89/89

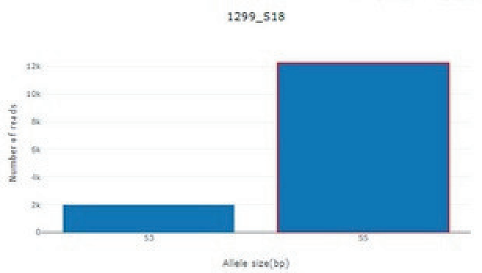

55/55

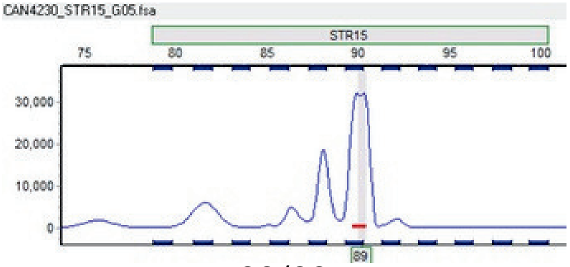

89/89

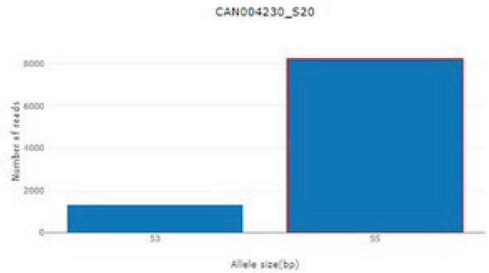

55/55

Capillary Electrophoresis - GeneMarker | Genotyping By Sequencing - SatAnalyzer

CfamSTR015 - Allele size difference - 34 bp

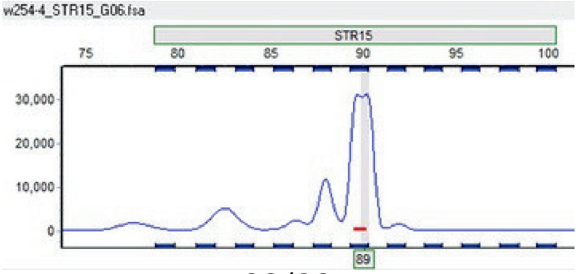

89/89

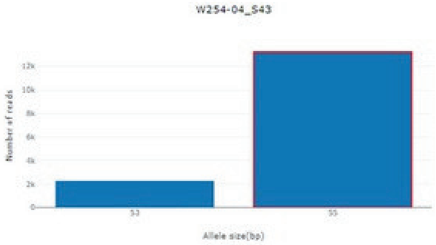

55/55

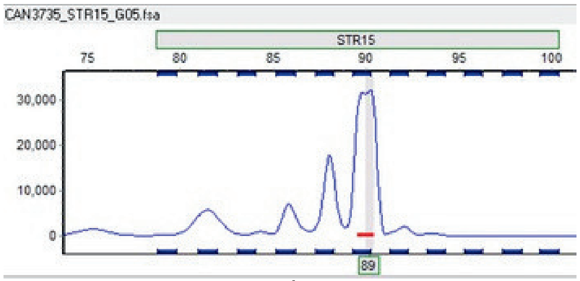

89/89

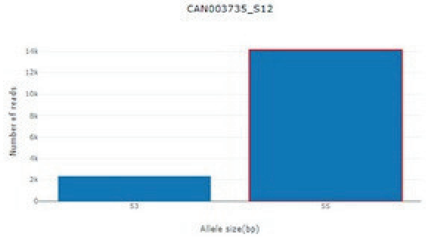

55/55

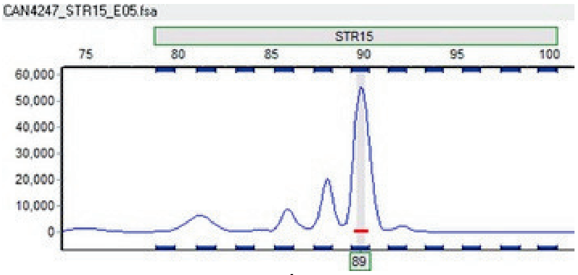

89/89

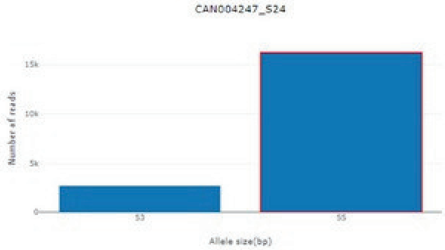

55/55

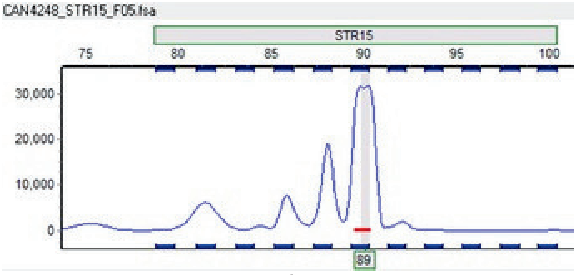

89/89

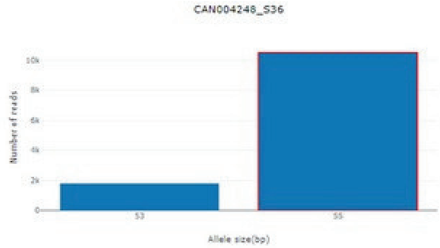

55/55

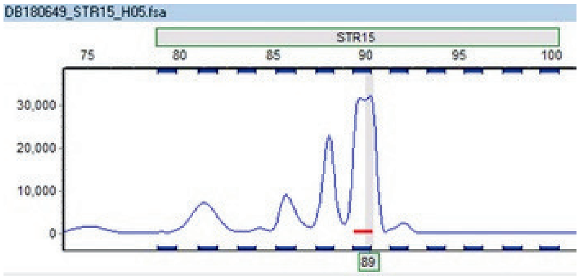

89/89

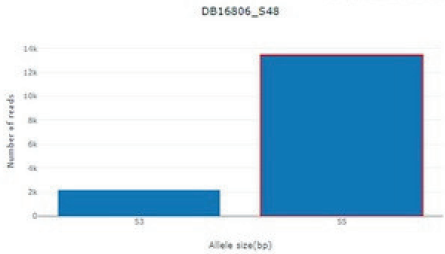

55/55

Capillary Electrophoresis - GeneMarker

Genotyping By Sequencing - SatAnalyzer

CfamSTR016 - Allele size difference - 36 bp

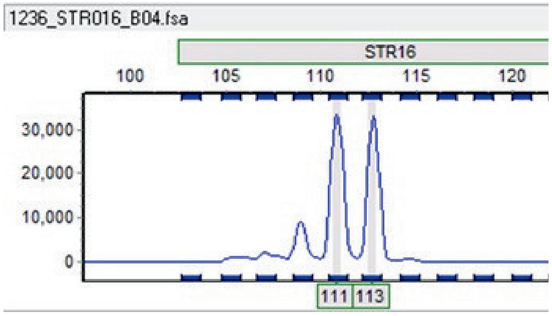

111/113

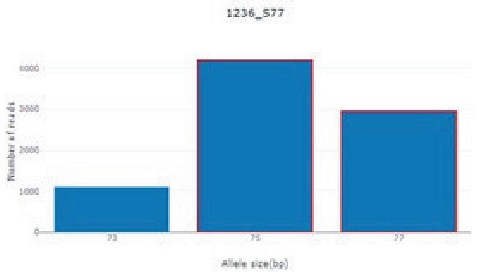

75/77

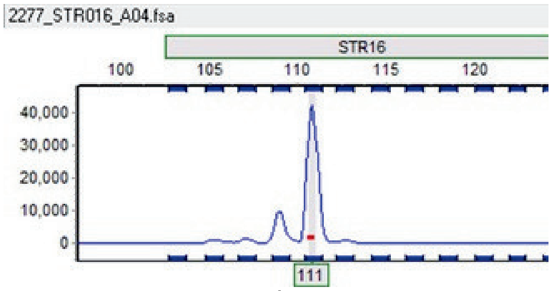

111/111

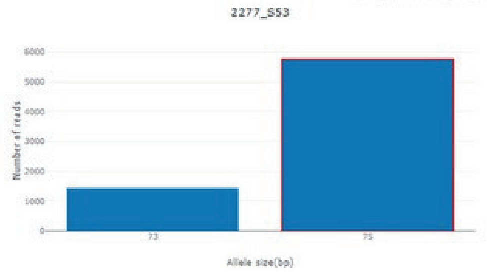

75/75

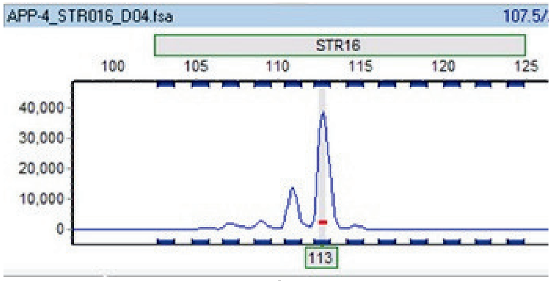

113/113

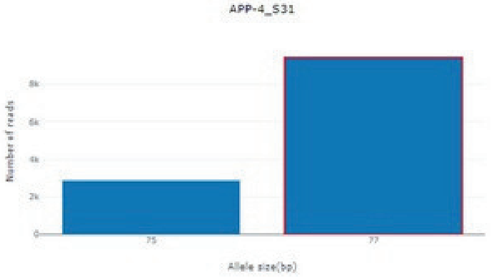

77/77

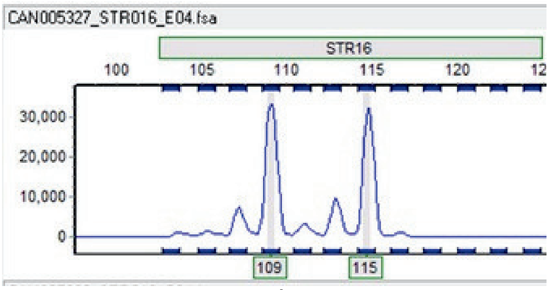

109/115

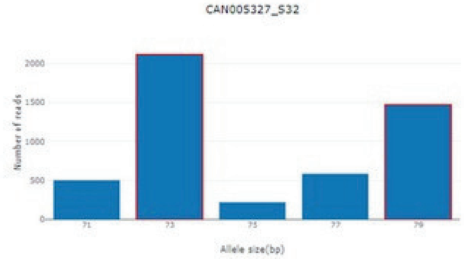

73/79

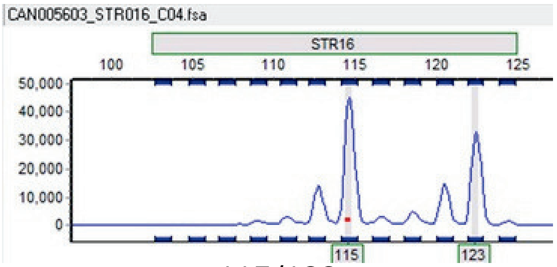

115/123

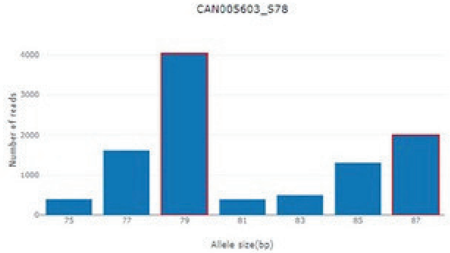

79/87

Capillary Electrophoresis - GeneMarker | Genotyping By Sequencing - SatAnalyzer

CfamSTR016 - Allele size difference - 36 bp

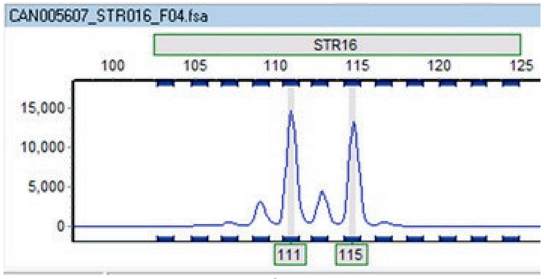

111/115

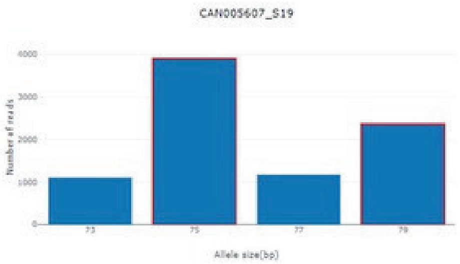

75/79

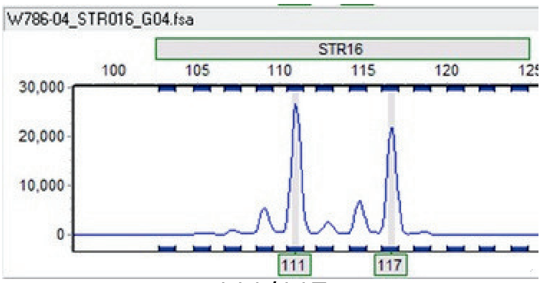

111/117

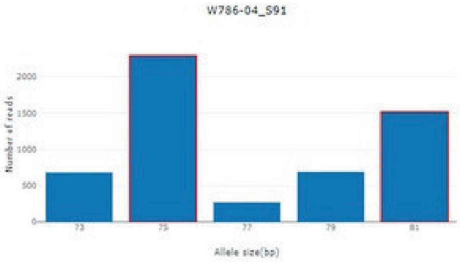

75/81

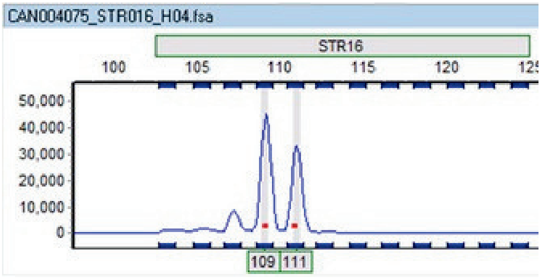

109/111

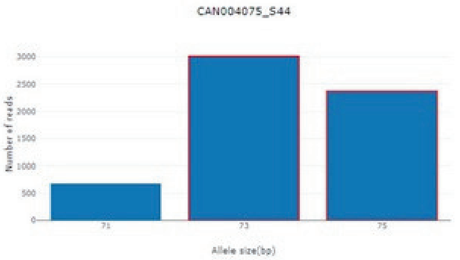

73/75

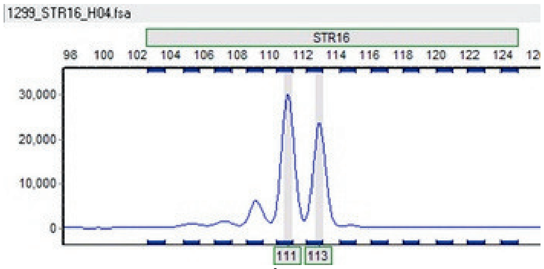

111/113

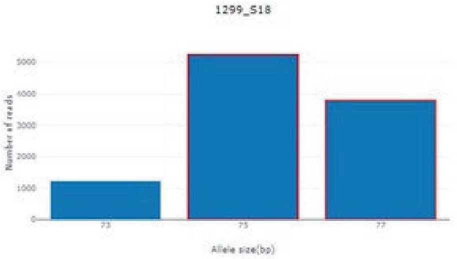

75/77

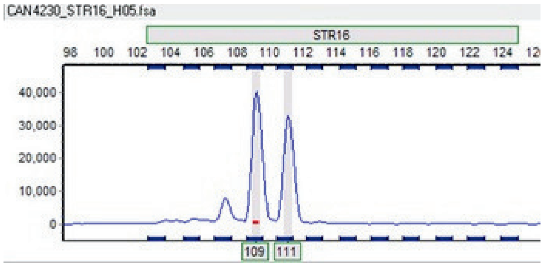

109/111

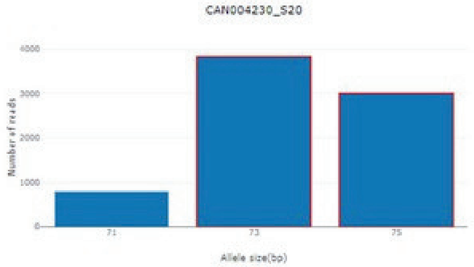

73/75

Capillary Electrophoresis - GeneMarker

Genotyping By Sequencing - SatAnalyzer

CfamSTR016 - Allele size difference - 36 bp

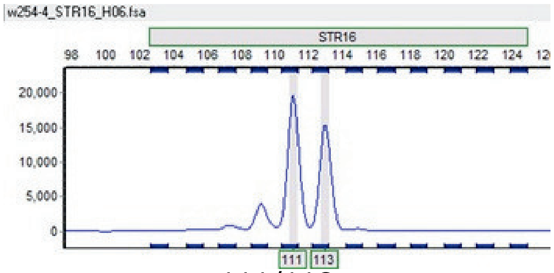

111/113

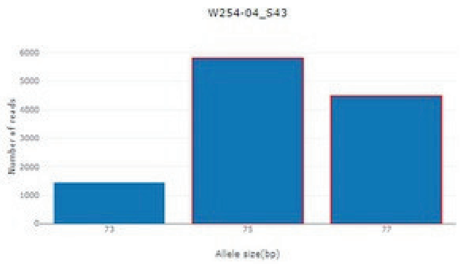

75/77

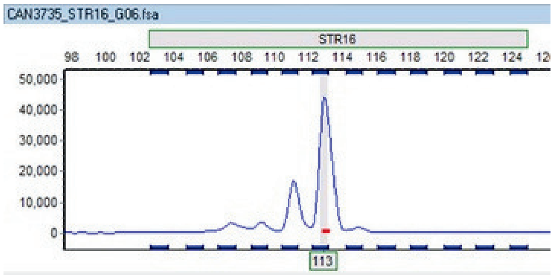

113/113

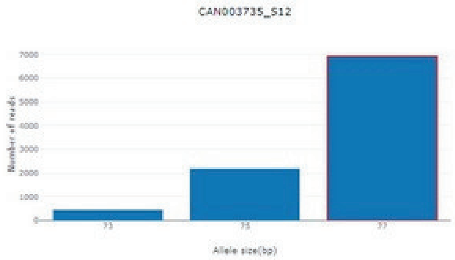

77/77

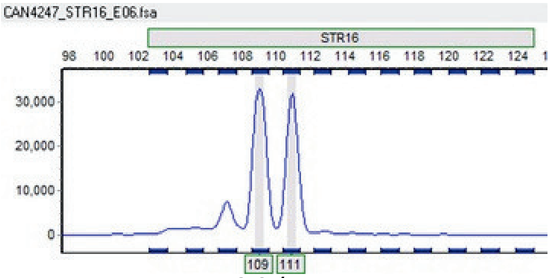

109/111

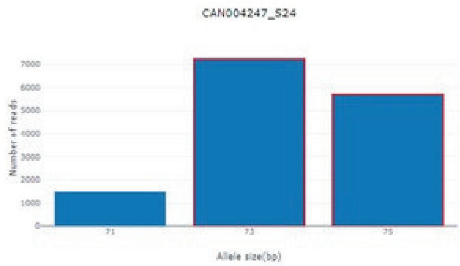

73/75

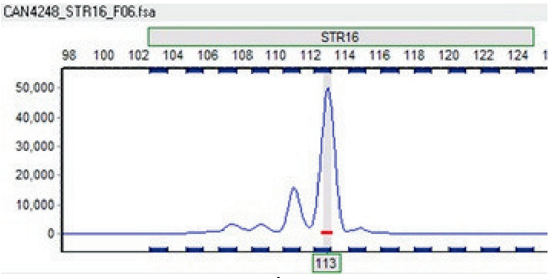

113/113

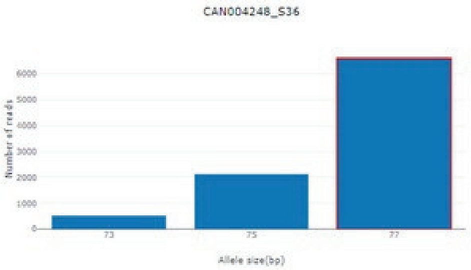

77/77

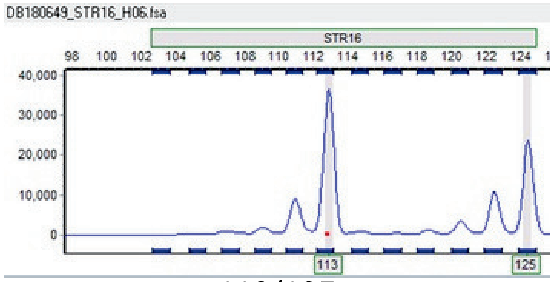

113/125

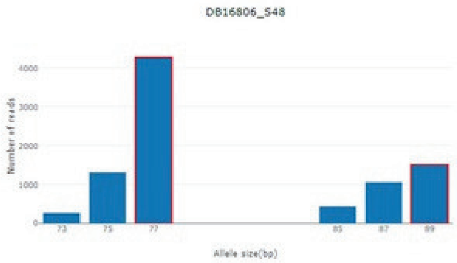

77/89

Capillary Electrophoresis - GeneMarker

Genotyping By Sequencing - SatAnalyzer

CfamSTR017 - Allele size difference - 35 bp

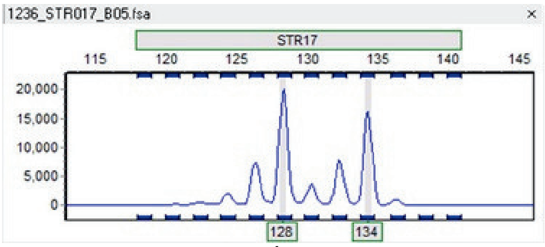

128/134

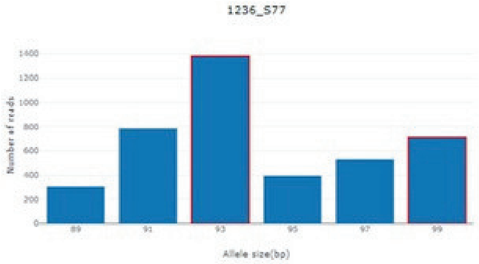

93/99

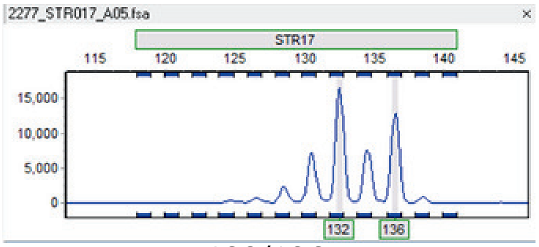

132/136

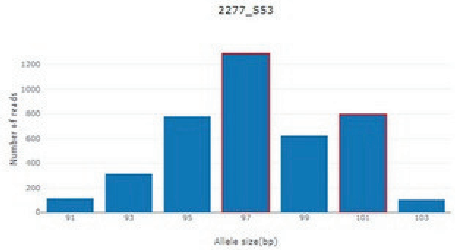

97/101

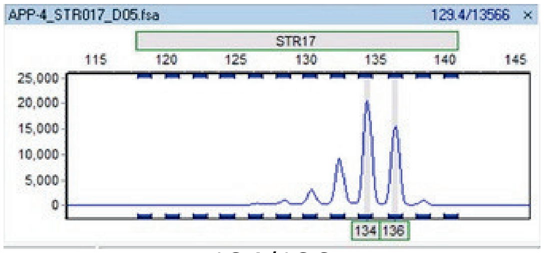

134/136

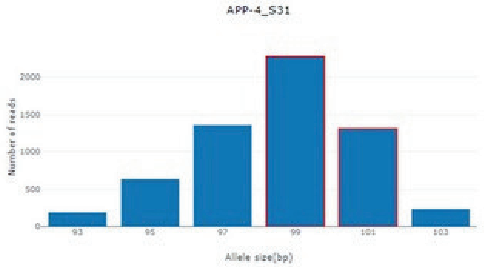

99/101

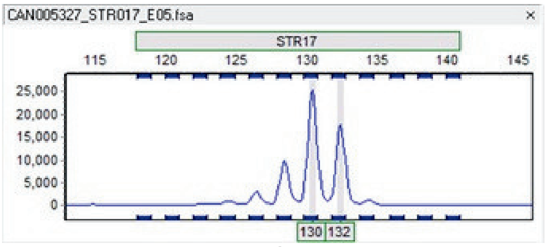

130/132

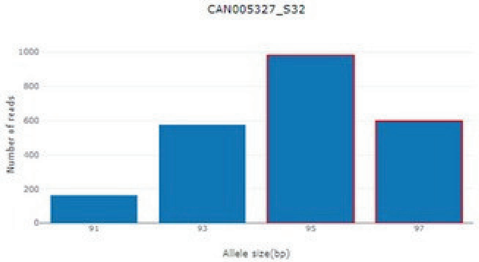

95/97

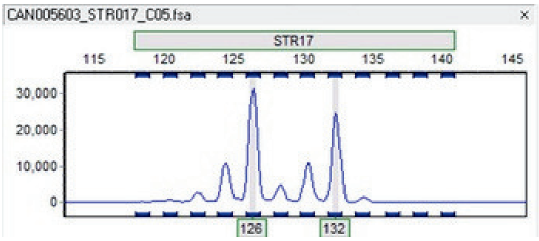

126/132

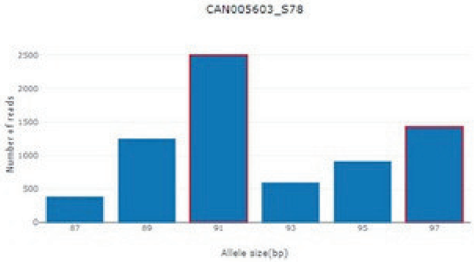

91/97

Capillary Electrophoresis - GeneMarker

Genotyping By Sequencing - SatAnalyzer

CfamSTR017 - Allele size difference - 35 bp

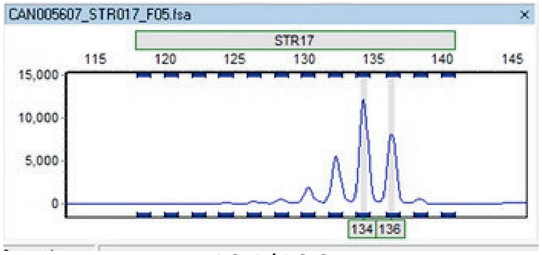

134/136

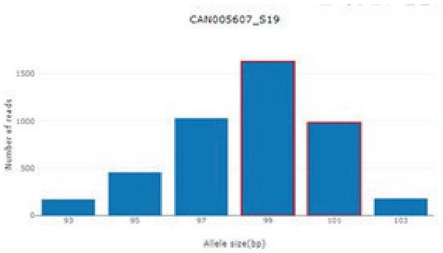

99/101

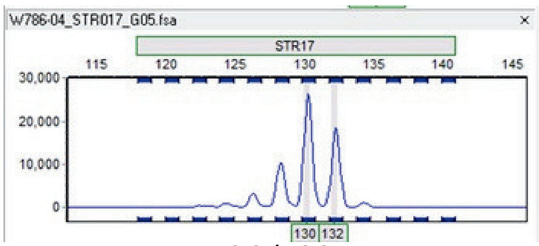

130/132

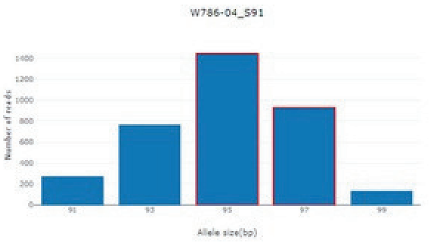

95/97

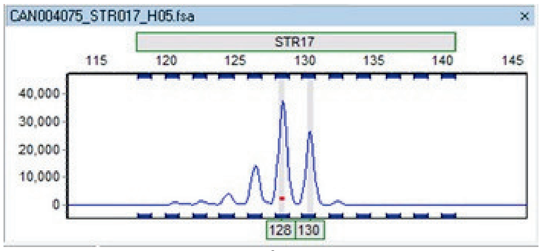

128/130

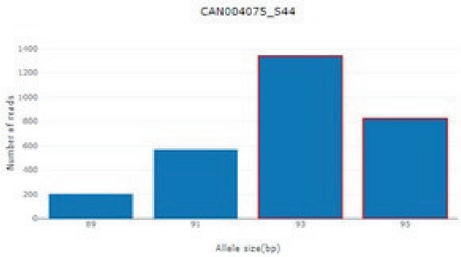

93/95

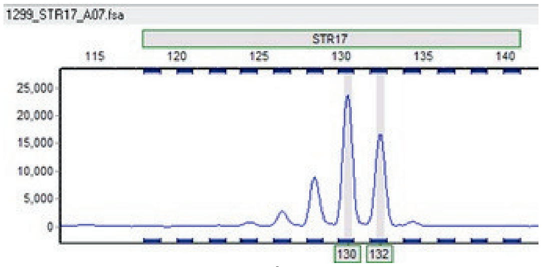

130/132

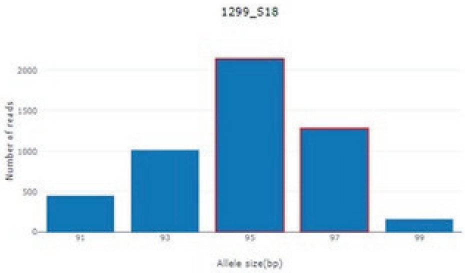

95/97

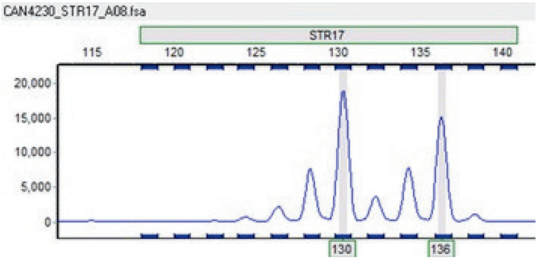

130/136

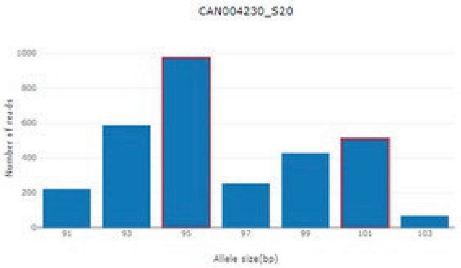

95/101

Capillary Electrophoresis - GeneMarker

Genotyping By Sequencing - SatAnalyzer

CfamSTR017 - Allele size difference - 35 bp

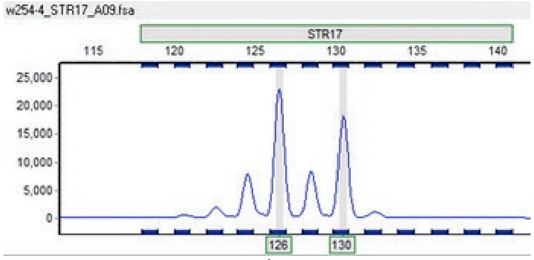

126/130

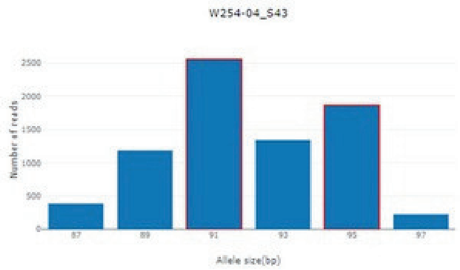

91/95

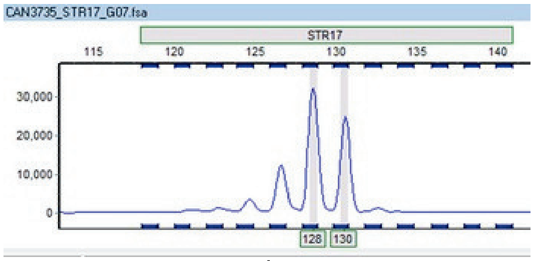

128/130

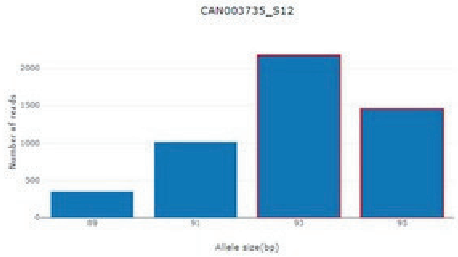

93/95

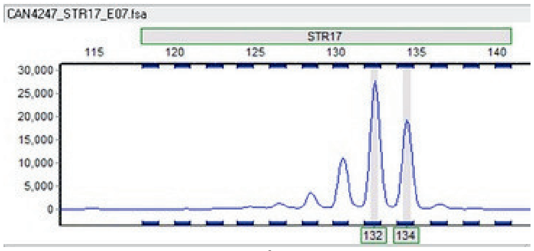

132/134

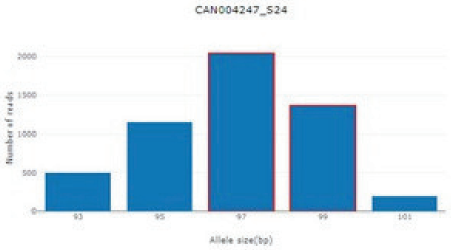

97/99

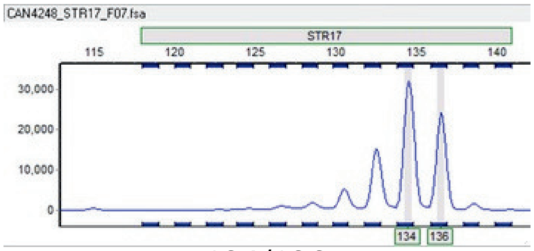

134/136

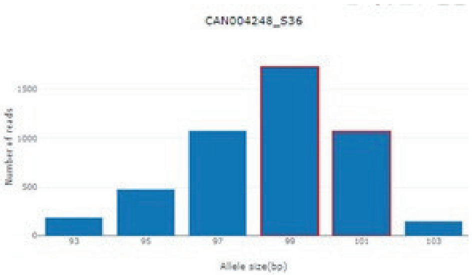

99/101

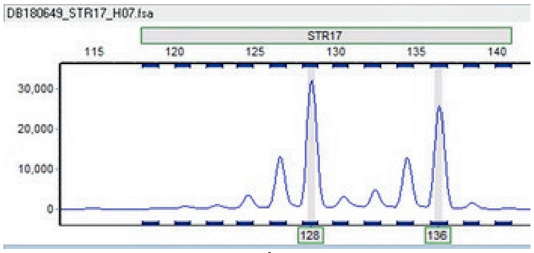

128/136

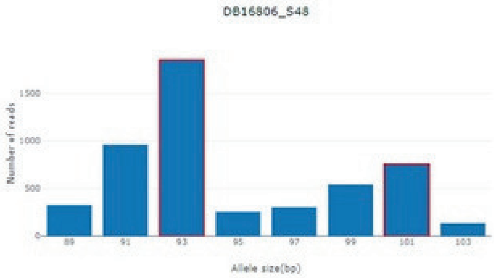

93/101

Capillary Electrophoresis - GeneMarker

Genotyping By Sequencing - SatAnalyzer

CfamSTR018 - Allele size difference - 39 bp

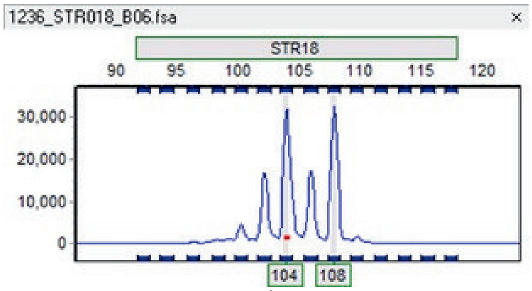

104/108

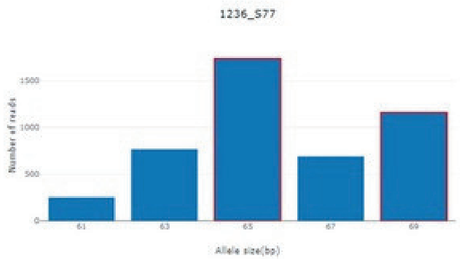

65/69

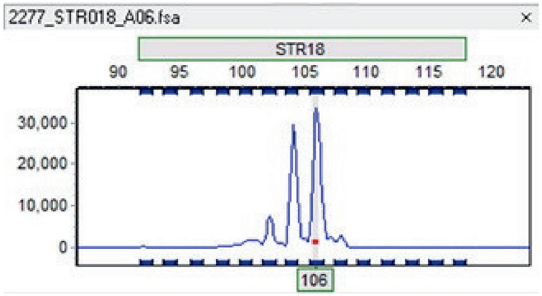

106/106

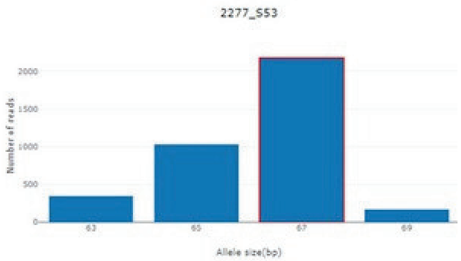

67/67

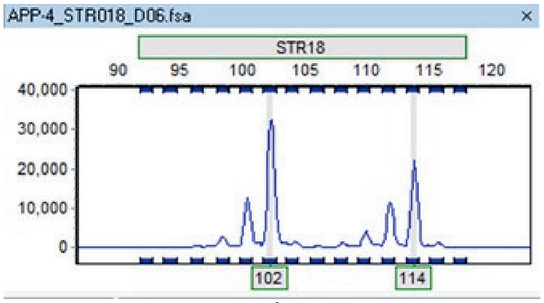

102/114

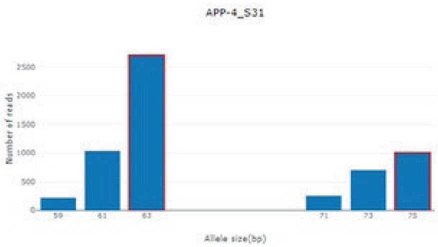

63/75

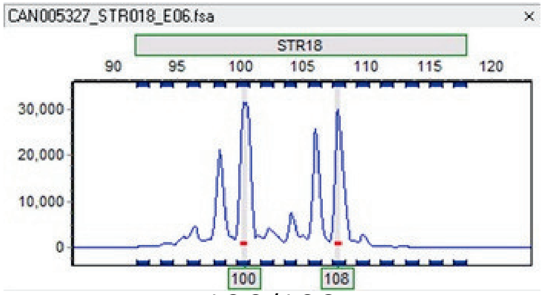

100/108

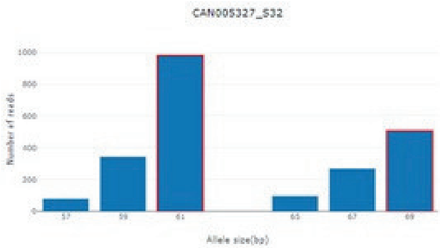

61/69

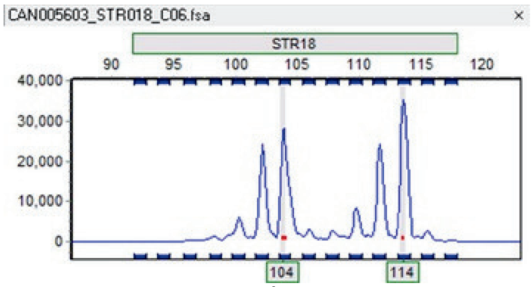

104/114

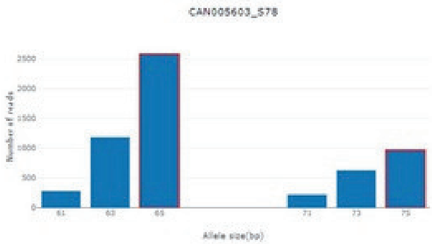

65/75

Capillary Electrophoresis - GeneMarker

Genotyping By Sequencing - SatAnalyzer

CfamSTR018 - Allele size difference - 39 bp

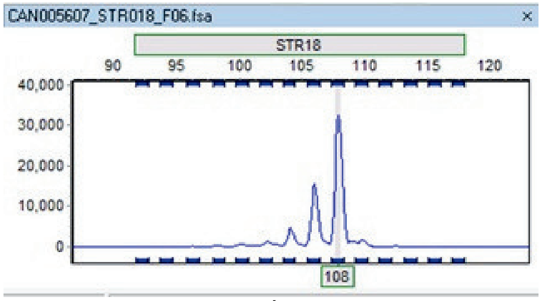

108/108

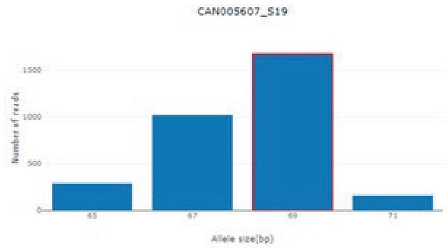

69/69

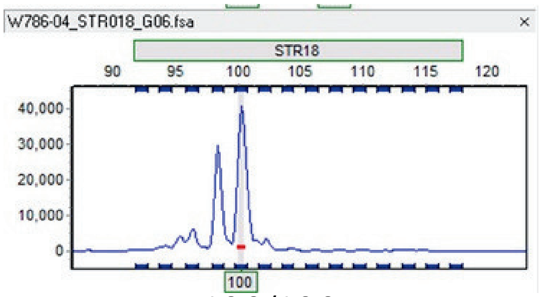

100/100

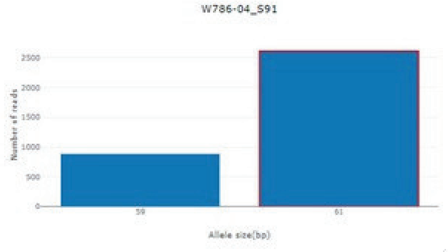

61/61

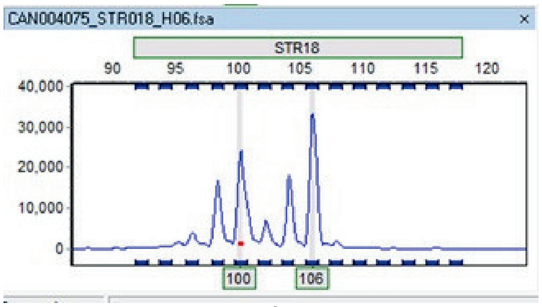

100/106

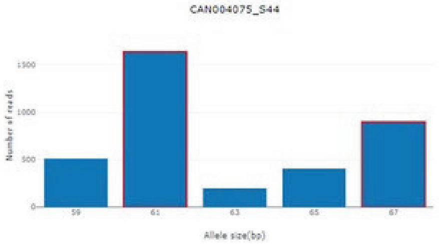

61/67

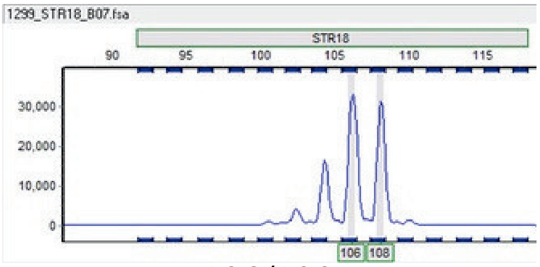

106/108

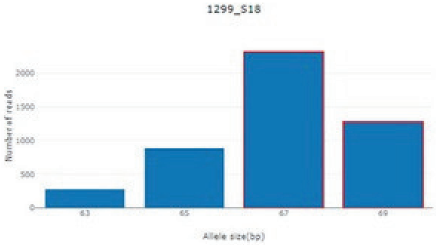

67/69

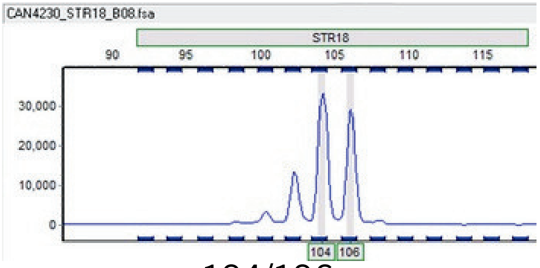

104/106

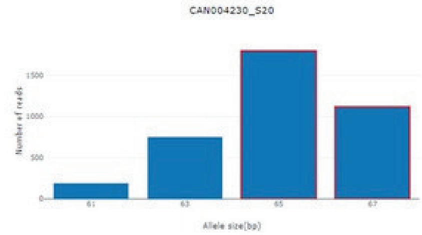

65/67

Capillary Electrophoresis - GeneMarker

Genotyping By Sequencing - SatAnalyzer

CfamSTR018 - Allele size difference - 39 bp

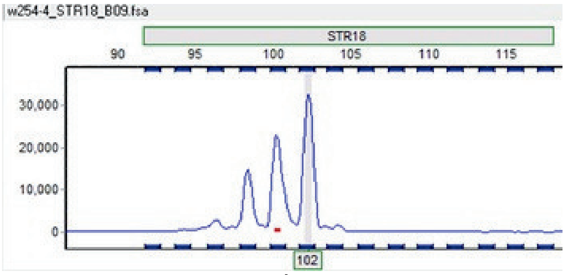

102/102

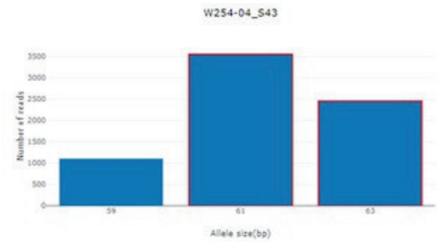

61/63

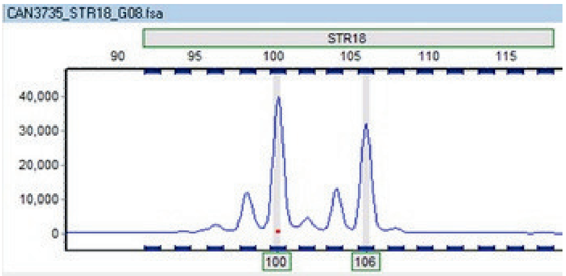

100/106

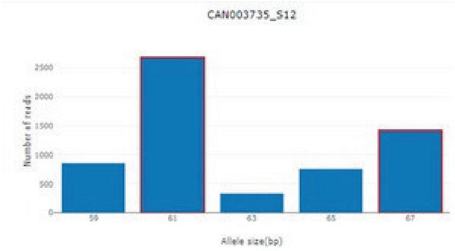

61/67

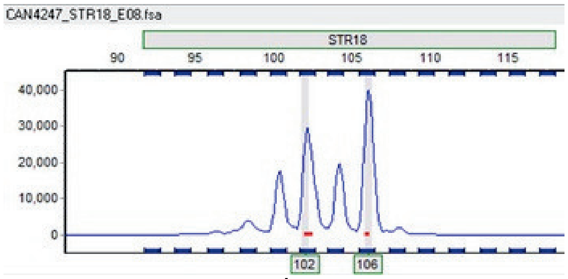

102/106

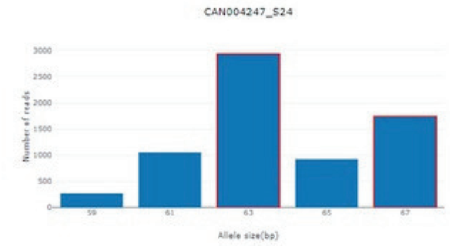

63/67

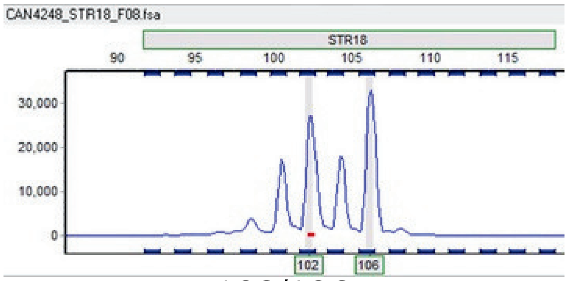

102/106

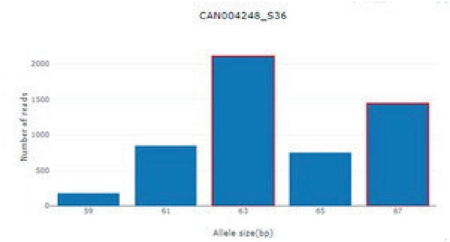

63/67

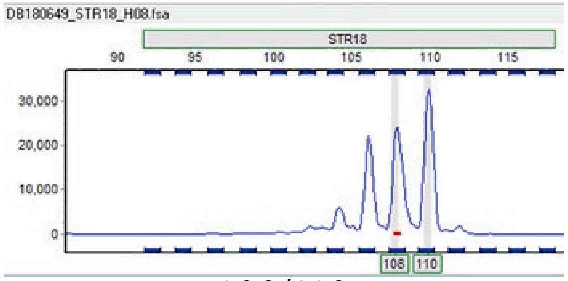

108/110

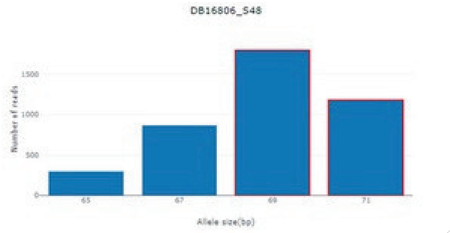

69/71

Capillary Electrophoresis - GeneMarker

Genotyping By Sequencing - SatAnalyzer

CfamSTR019 - Allele size difference - 42 bp

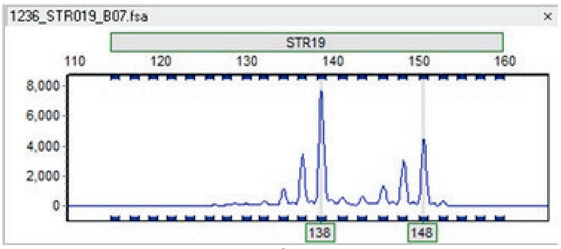

138/148

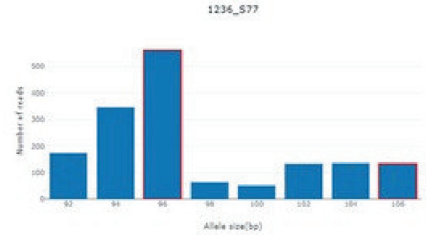

96/106

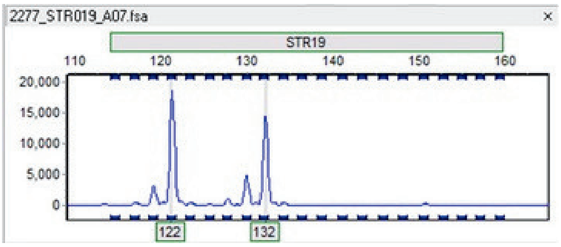

122/132

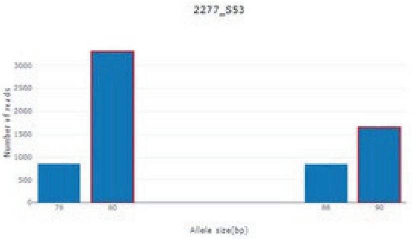

80/90

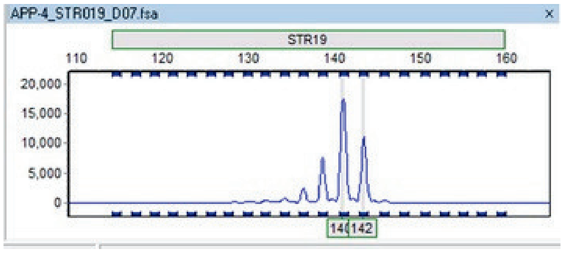

140/142

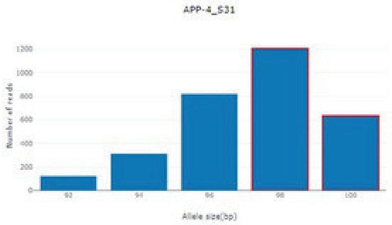

98/100

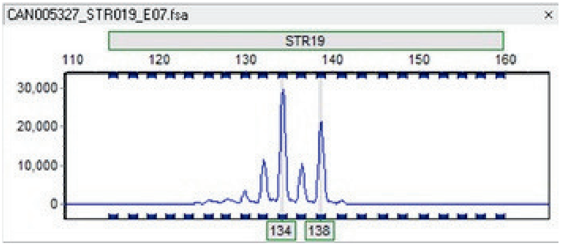

134/138

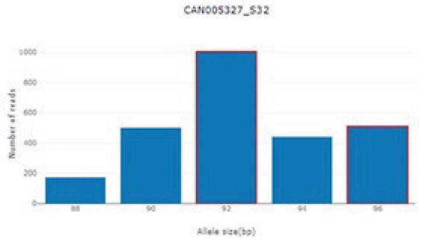

92/96

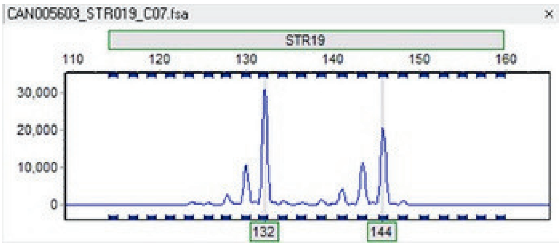

132/144

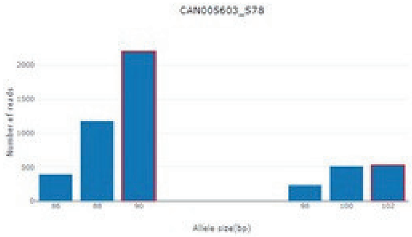

90/102

Capillary Electrophoresis - GeneMarker

Genotyping By Sequencing - SatAnalyzer

CfamSTR019 - Allele size difference - 42 bp

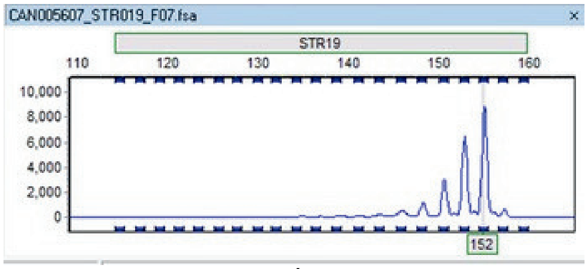

152/152

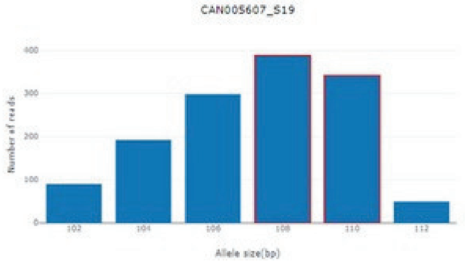

108/110

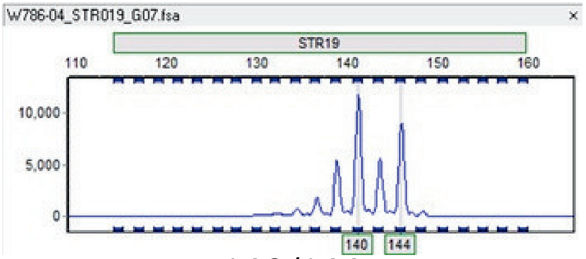

140/144

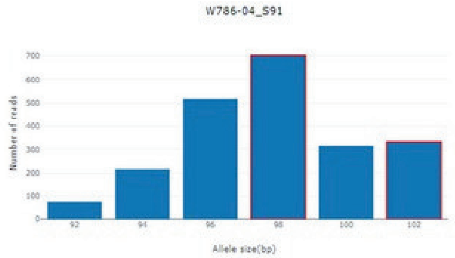

98/102

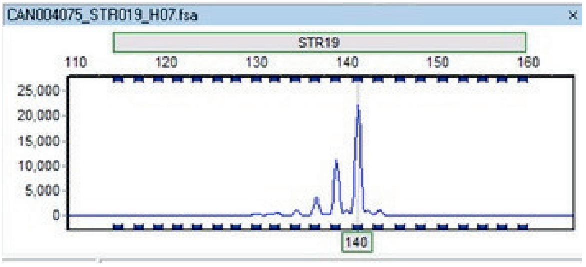

140/140

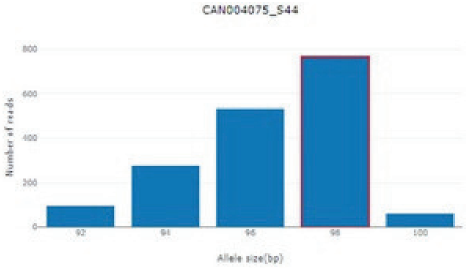

98/98

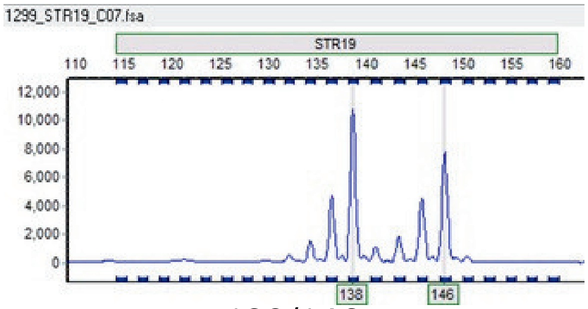

138/146

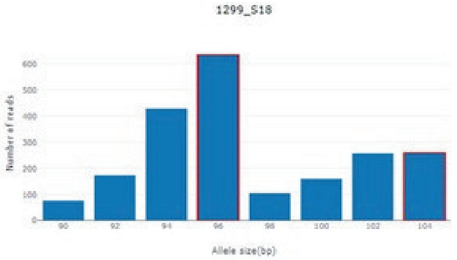

96/104

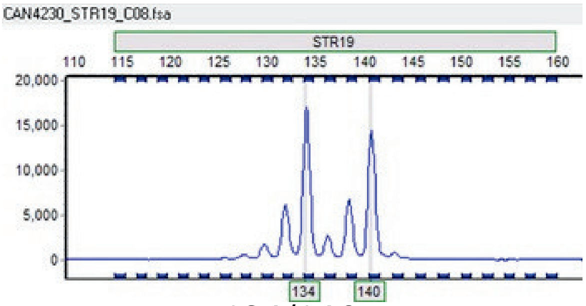

134/140

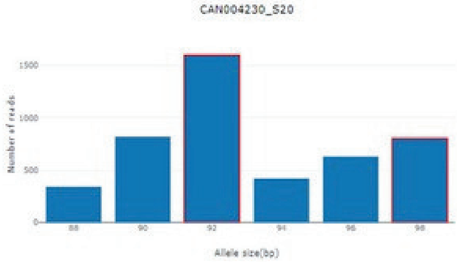

92/98

Capillary Electrophoresis - GeneMarker

Genotyping By Sequencing - SatAnalyzer

CfamSTR019 - Allele size difference - 42 bp

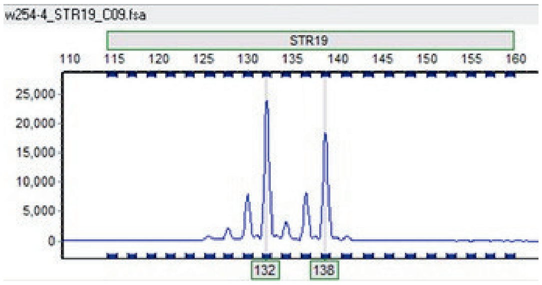

132/138

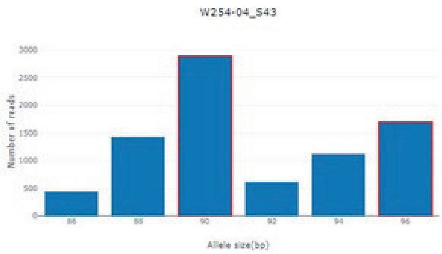

90/96

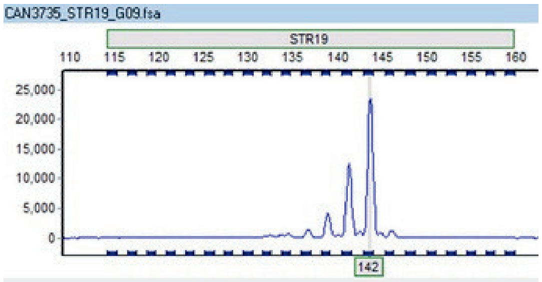

142/142

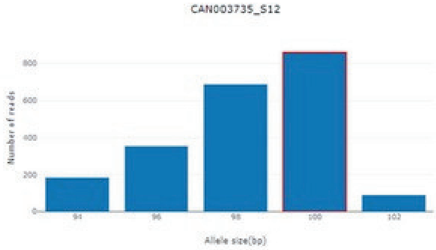

100/100

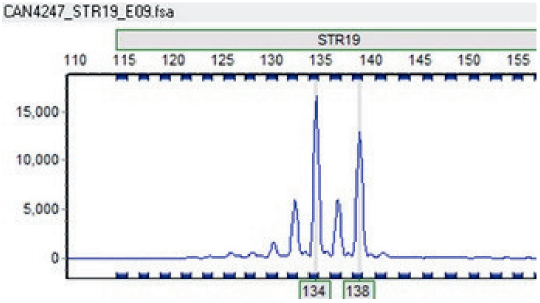

134/138

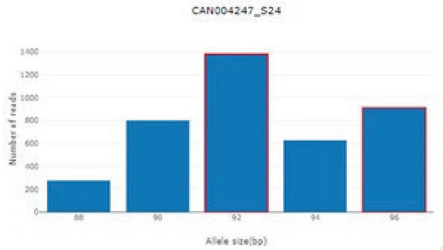

92/96

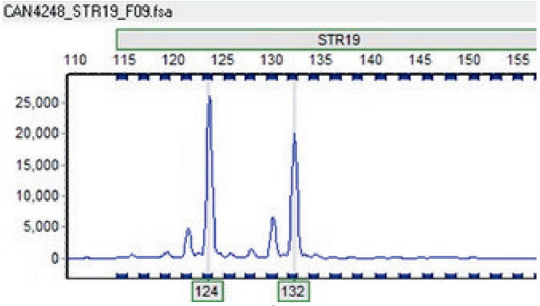

124/132

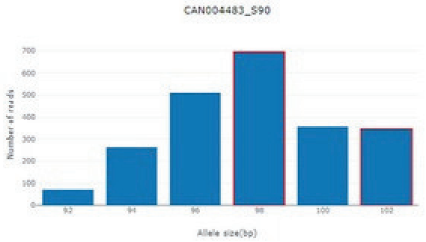

82/90

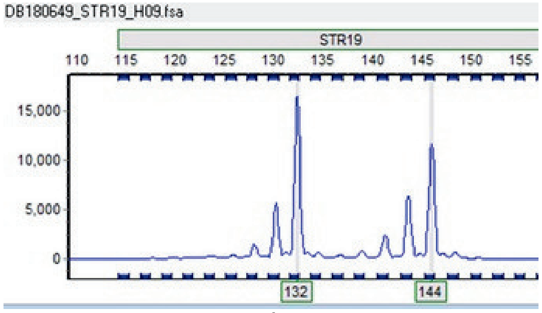

132/144

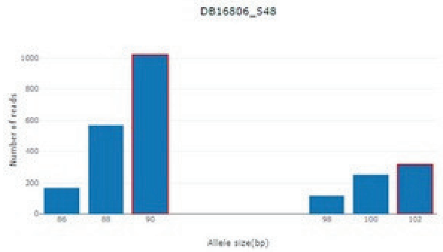

90/102

Capillary Electrophoresis - GeneMarker | Genotyping By Sequencing - SatAnalyzer

CfamSTR020 - Allele size difference - 39 bp

Note non-specific amplification at 95bp in some samples (CE)

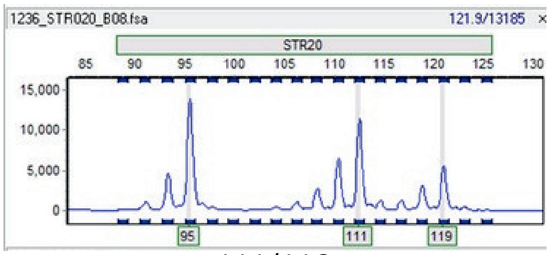

111/119

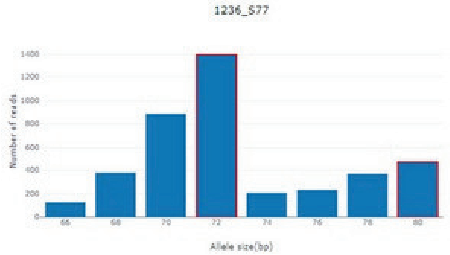

72/80

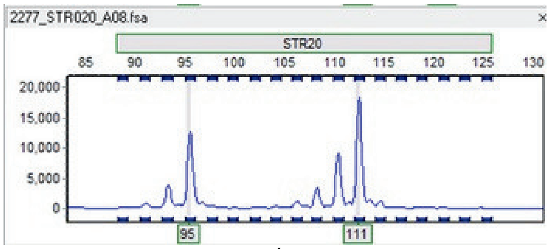

111/111

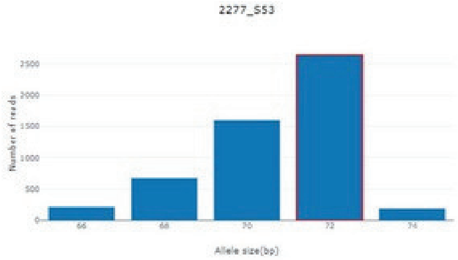

72/72

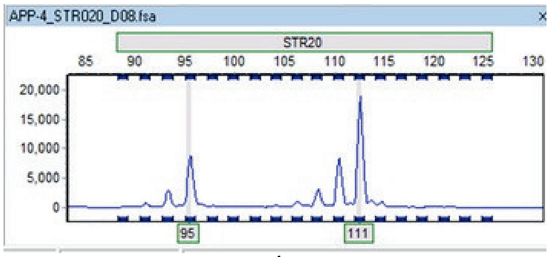

111/111

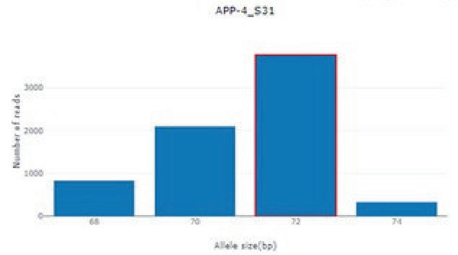

72/72

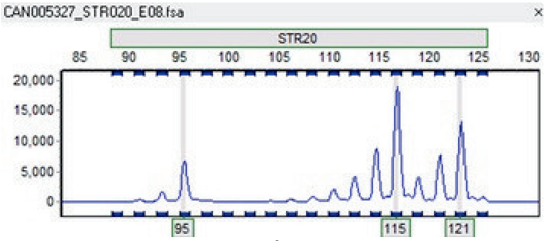

115/121

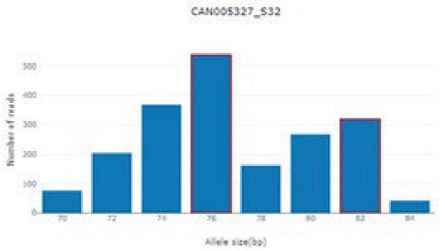

76/82

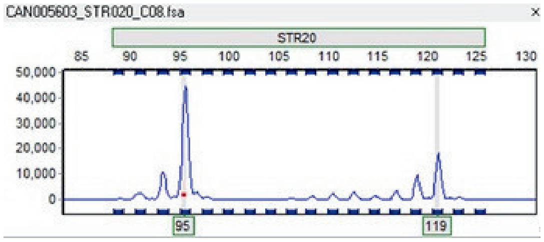

95/119

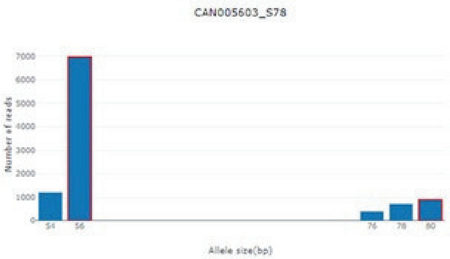

56/80

Capillary Electrophoresis - GeneMarker

Genotyping By Sequencing - SatAnalyzer

CfamSTR020 - Allele size difference - 39 bp

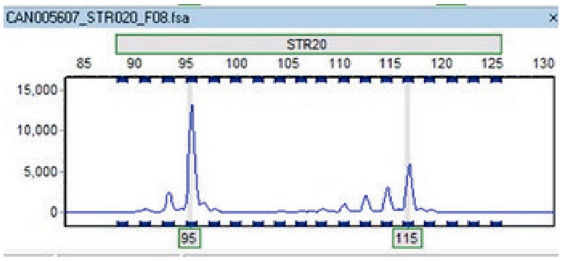

95/115

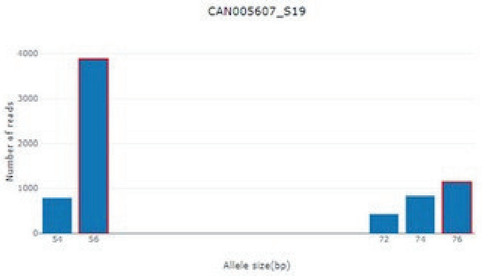

56/76

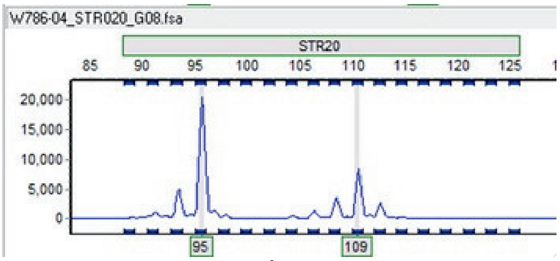

95/109

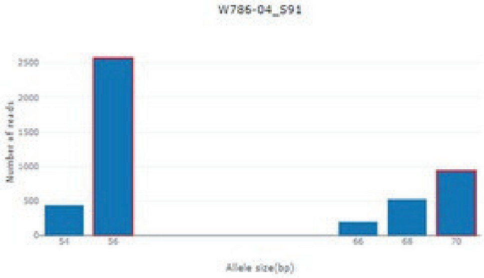

56/70

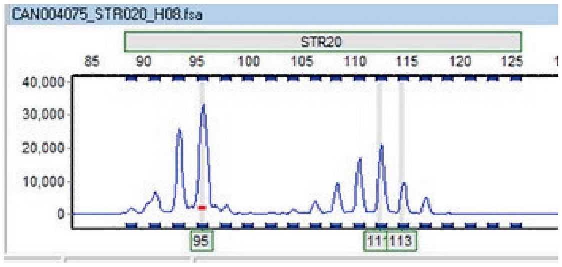

111/113

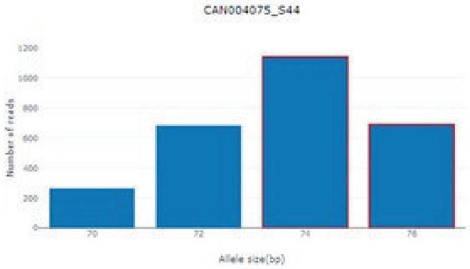

74/76

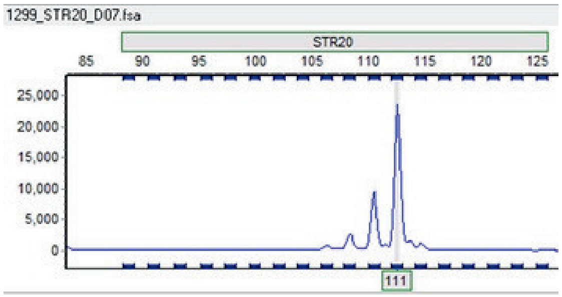

111/111

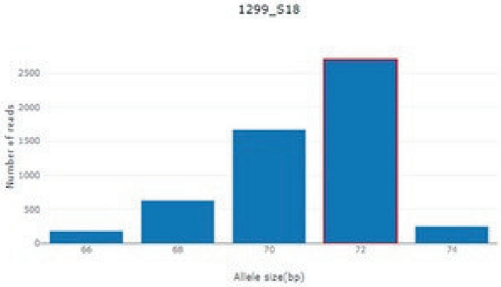

72/72

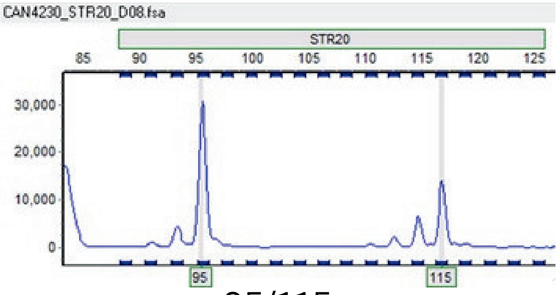

95/115

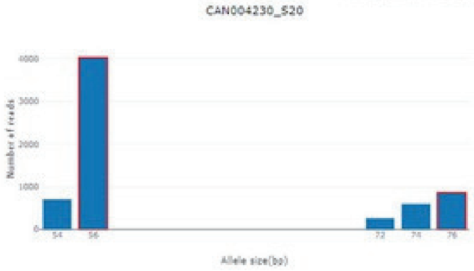

56/76

Capillary Electrophoresis - GeneMarker | Genotyping By Sequencing - SatAnalyzer

CfamSTR020 - Allele size difference - 39 bp

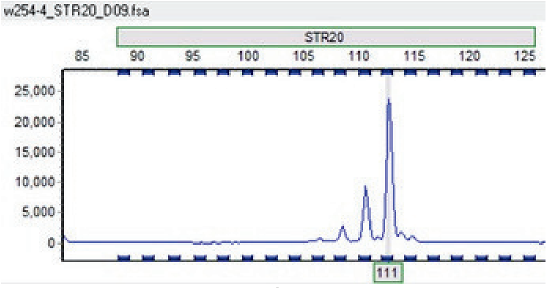

111/111

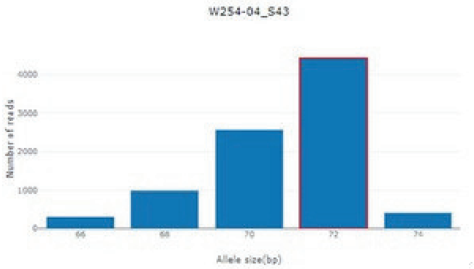

72/72

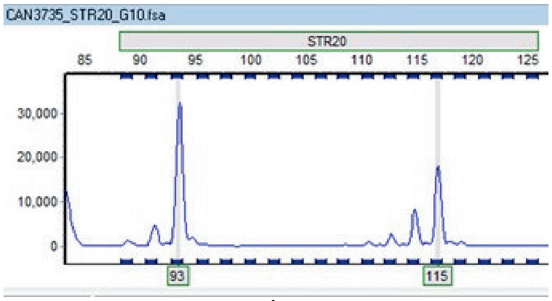

93/115

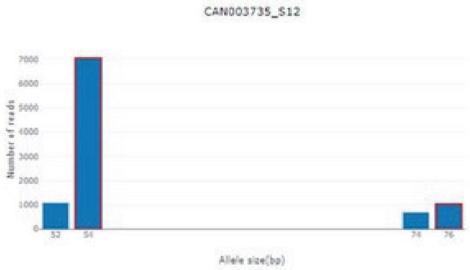

54/76

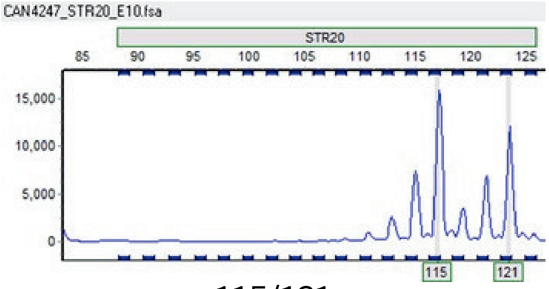

115/121

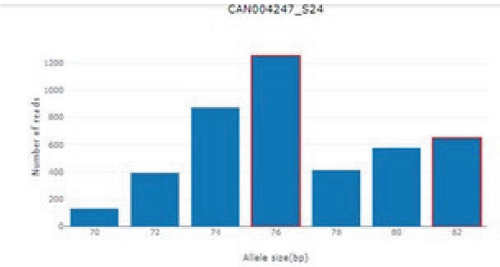

76/82

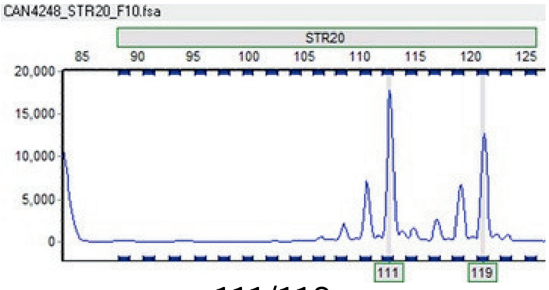

111/119

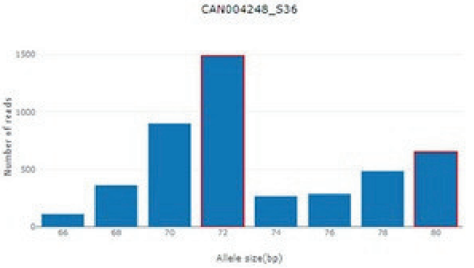

72/80

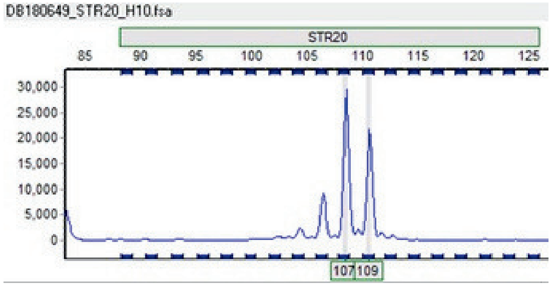

107/109

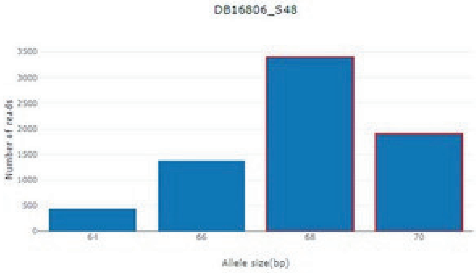

68/70

Capillary Electrophoresis - GeneMarker

Genotyping By Sequencing - SatAnalyzer

CfamSTR021 - Allele size difference - 40 bp

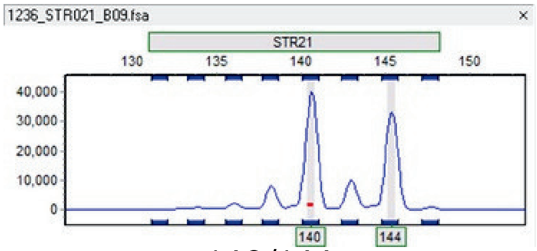

140/144

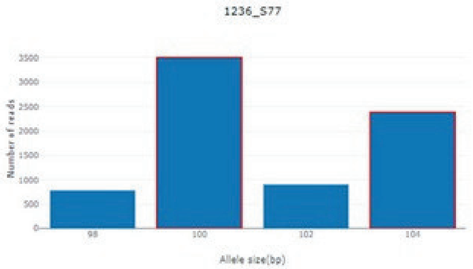

100/104

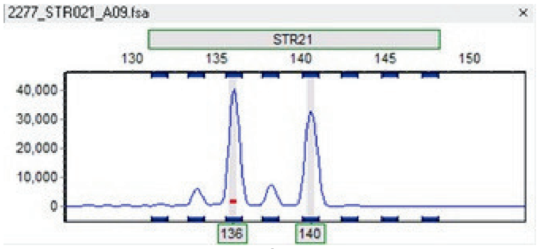

136/140

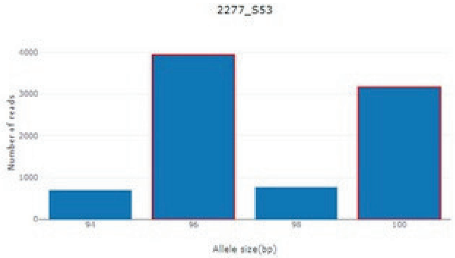

96/100

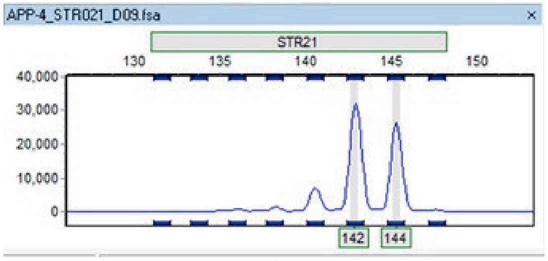

142/144

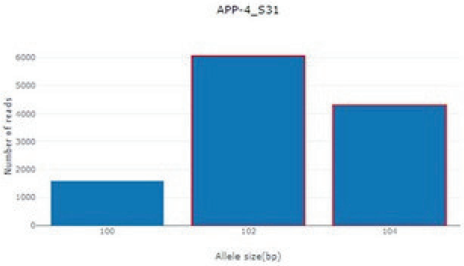

102/104

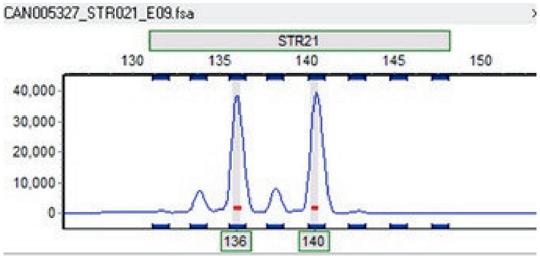

136/140

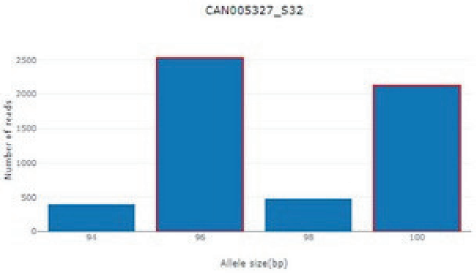

96/100

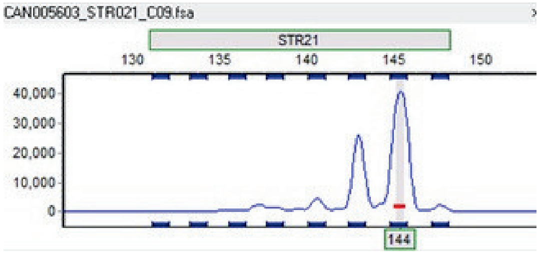

144/144

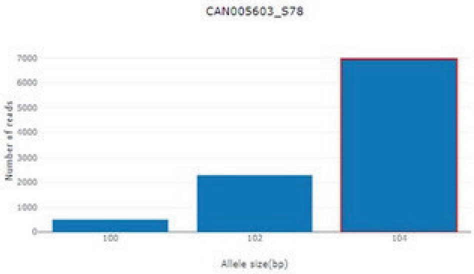

104/104

Capillary Electrophoresis - GeneMarker

Genotyping By Sequencing - SatAnalyzer

CfamSTR021 - Allele size difference - 40 bp

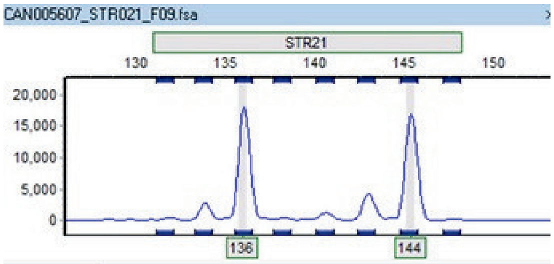

136/144

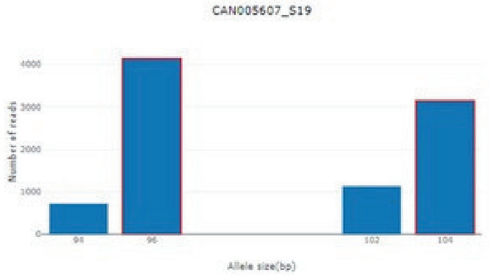

96/104

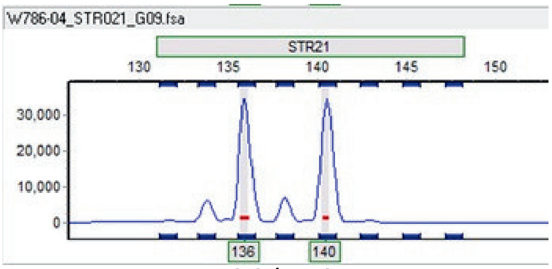

136/140

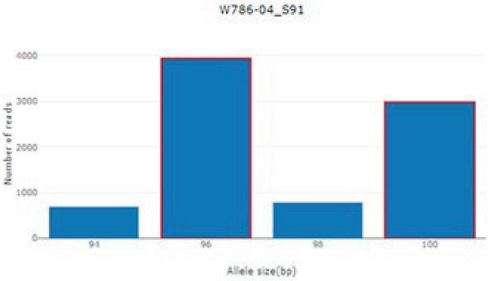

96/100

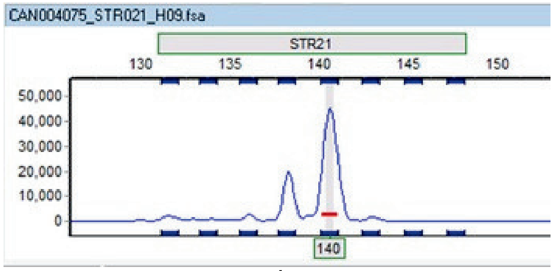

140/140

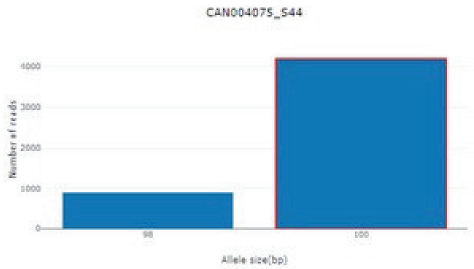

100/100

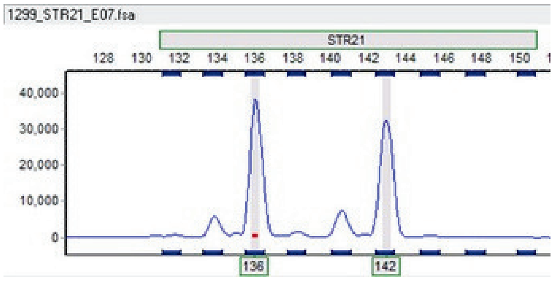

136/142

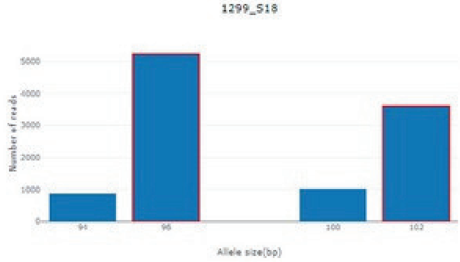

96/102

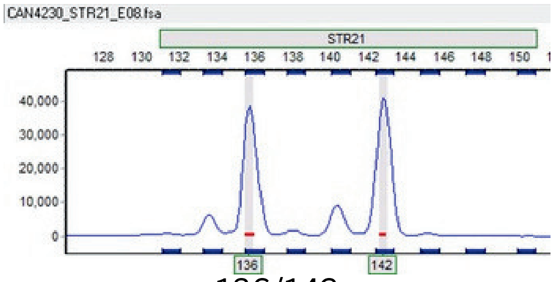

136/142

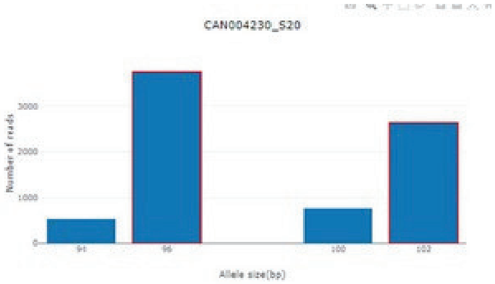

96/102

Capillary Electrophoresis - GeneMarker

Genotyping By Sequencing - SatAnalyzer

CfamSTR021 - Allele size difference - 40 bp

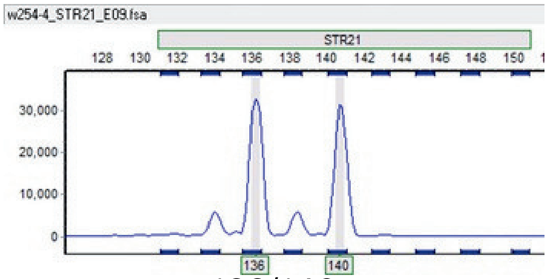

136/140

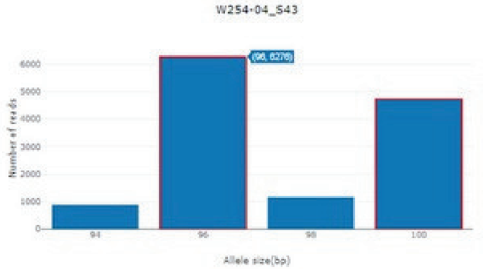

96/100

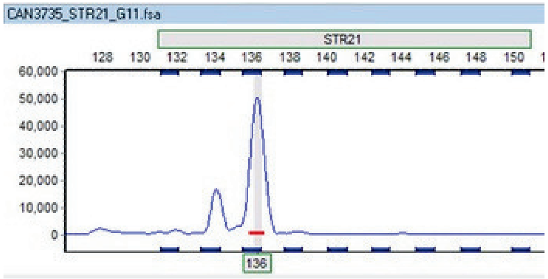

136/136

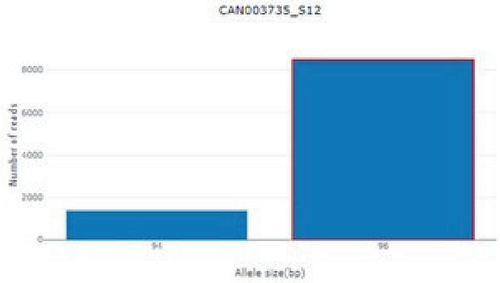

96/96

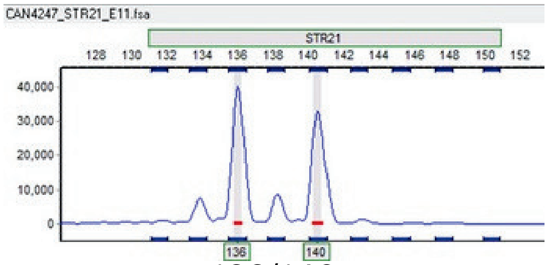

136/140

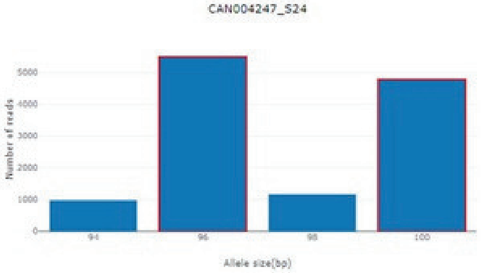

96/100

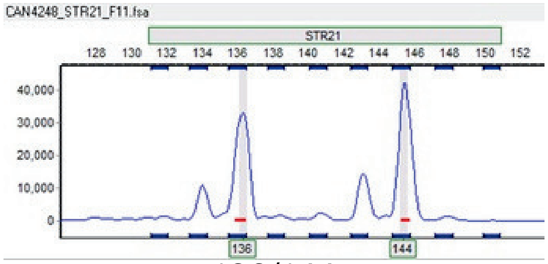

136/144

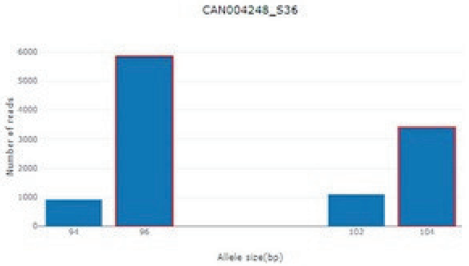

96/104

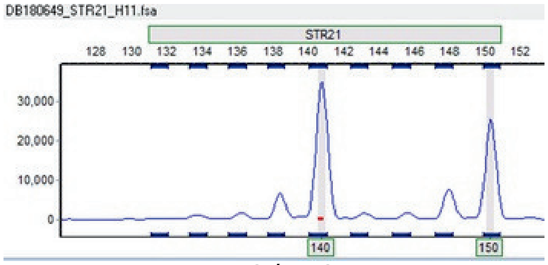

140/150

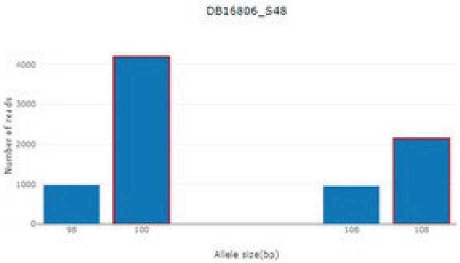

100/108

Capillary Electrophoresis - GeneMarker

Genotyping By Sequencing - SatAnalyzer

CfamSTR022 - Allele size difference - 37 bp

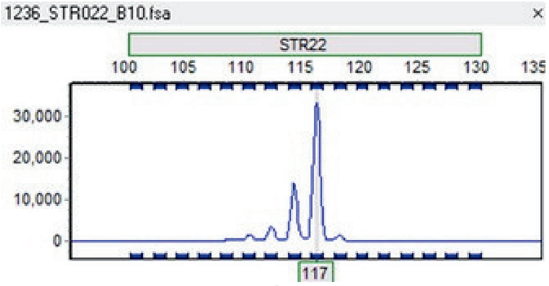

117/117

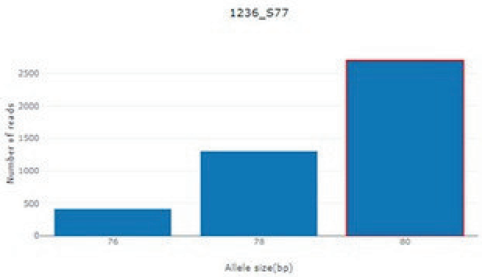

80/80

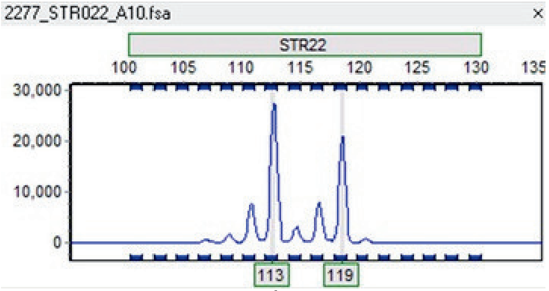

113/119

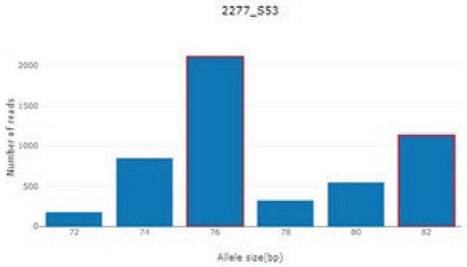

76/82

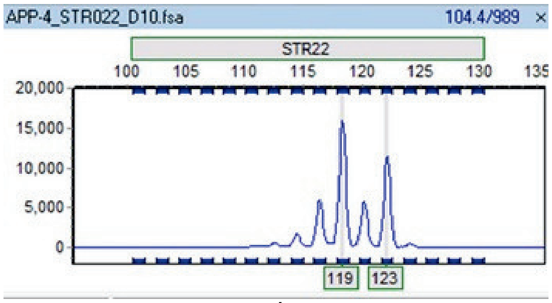

119/123

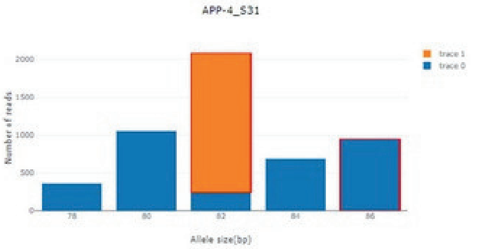

82/86

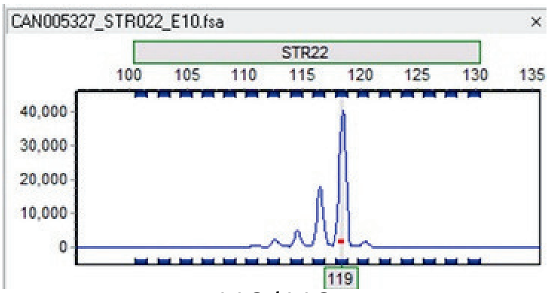

119/119

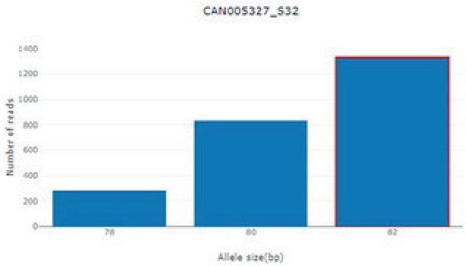

82/82

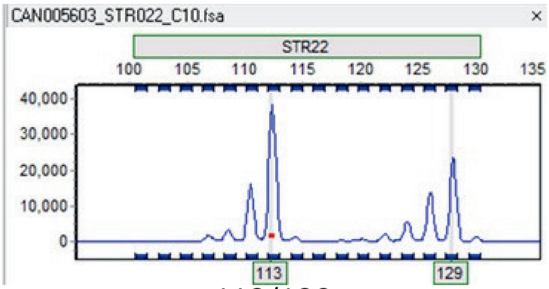

113/129

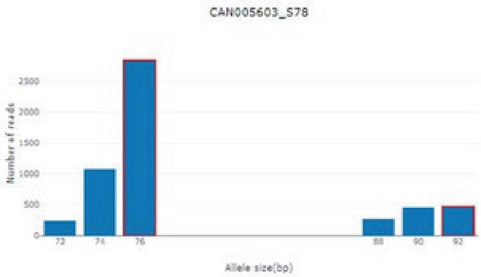

76/92

Capillary Electrophoresis - GeneMarker

Genotyping By Sequencing - SatAnalyzer

CfamSTR022 - Allele size difference - 37 bp

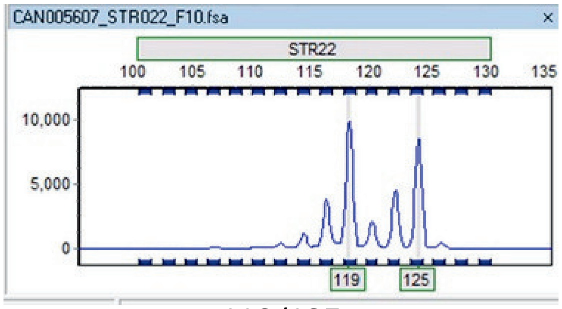

119/125

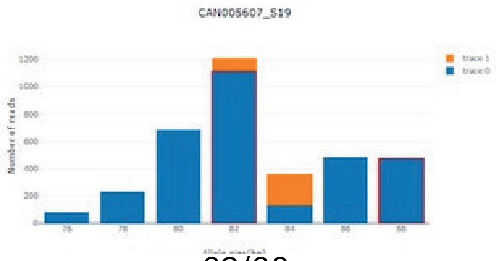

82/88

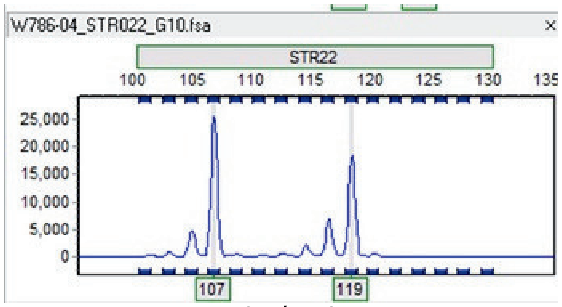

107/119

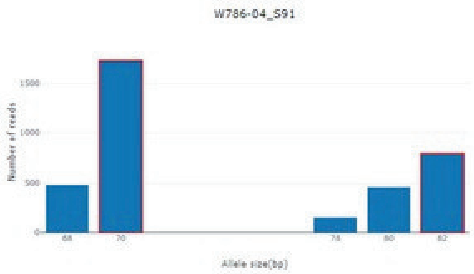

70/82

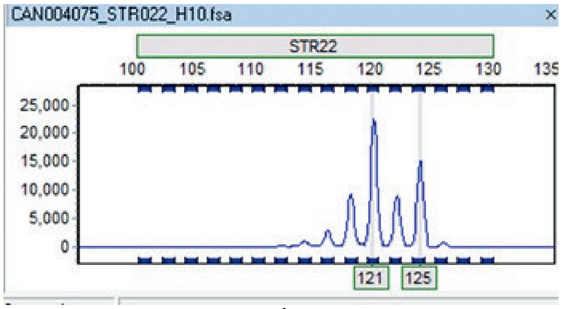

121/125

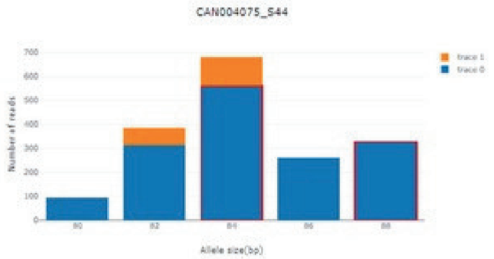

84/88

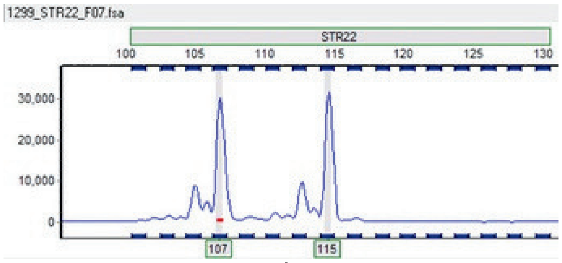

107/115

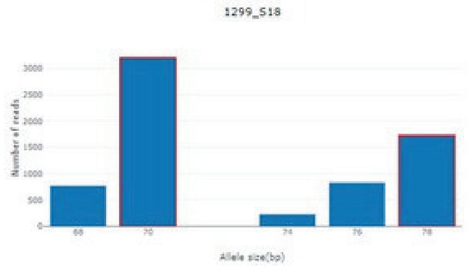

70/78

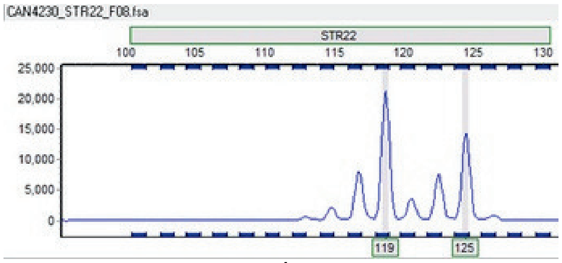

119/125

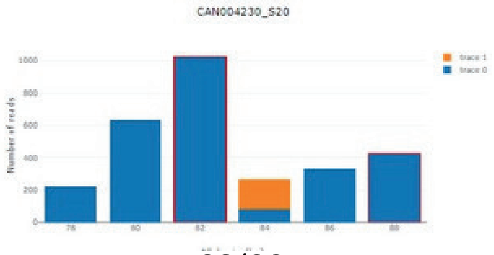

82/88

Capillary Electrophoresis - GeneMarker

Genotyping By Sequencing - SatAnalyzer

CfamSTR022 - Allele size difference - 37 bp

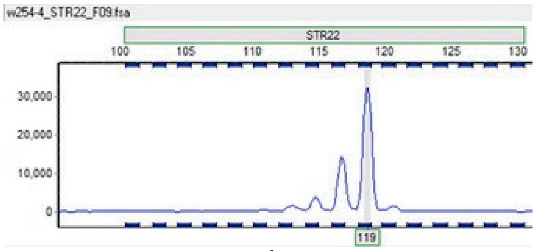

119/119

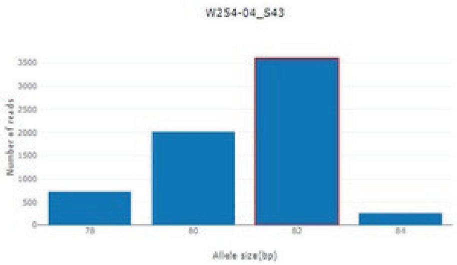

82/82

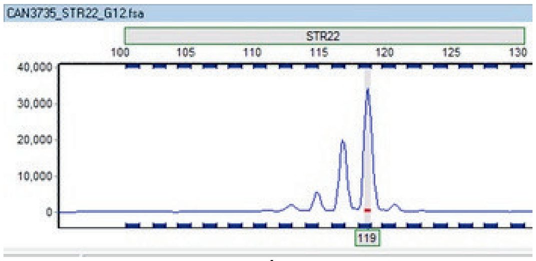

119/119

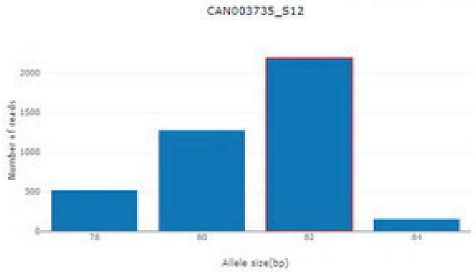

82/82

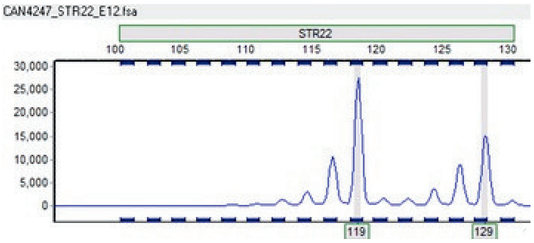

119/129

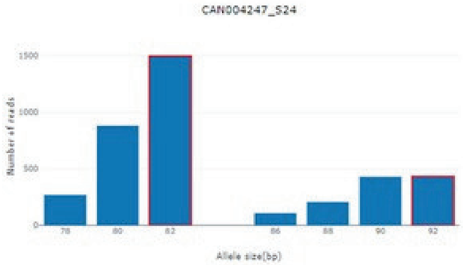

82/92

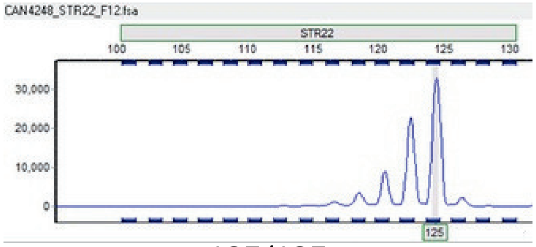

125/125

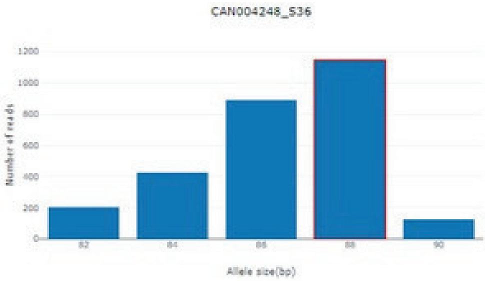

88/88

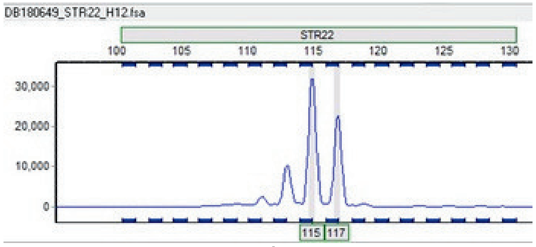

115/117

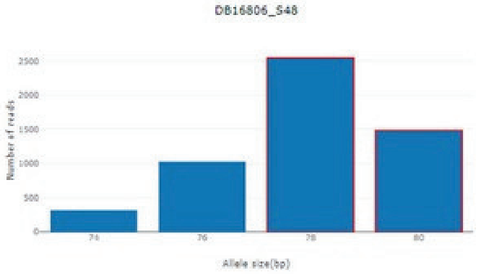

78/80

Capillary Electrophoresis - GeneMarker

Genotyping By Sequencing - SatAnalyzer

CfamSTR023 - Allele size difference - 36 bp

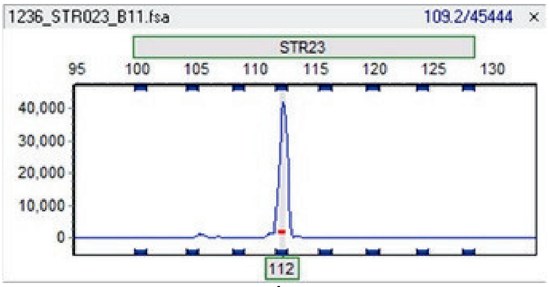

112/112

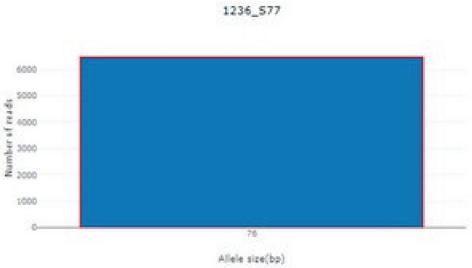

76/76

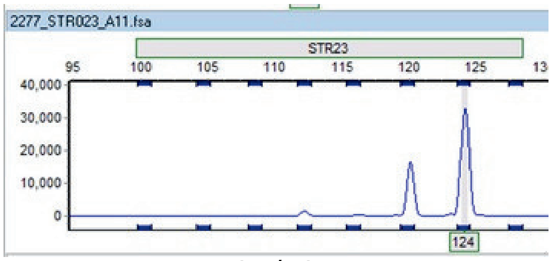

124/124

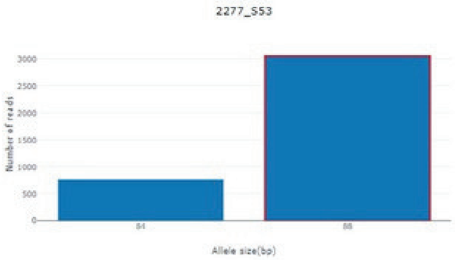

88/88

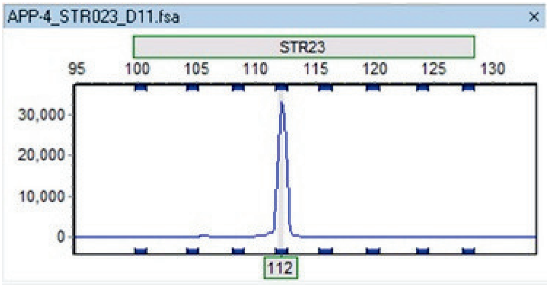

112/112

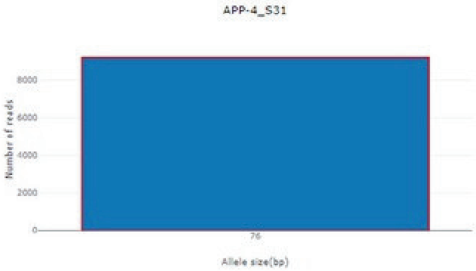

76/76

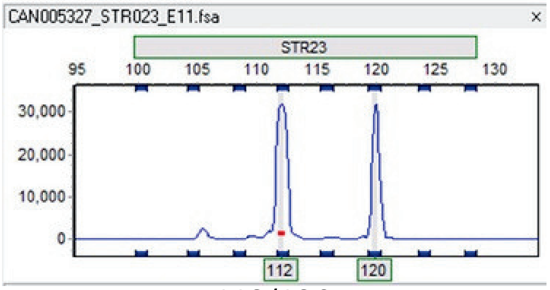

112/120

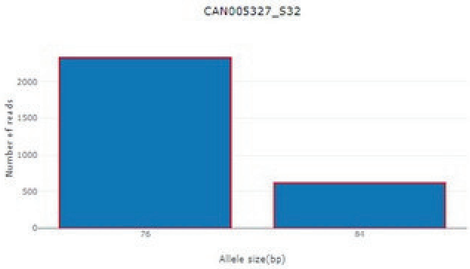

76/84

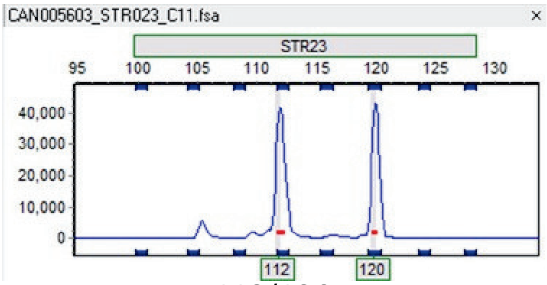

112/120

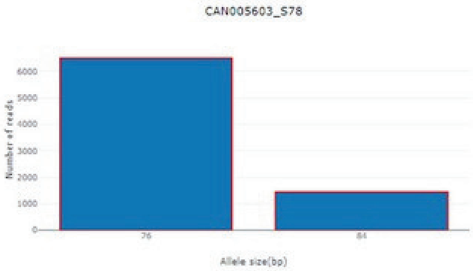

76/84

Capillary Electrophoresis - GeneMarker | Genotyping By Sequencing - SatAnalyzer

CfamSTR023 - Allele size difference - 36 bp

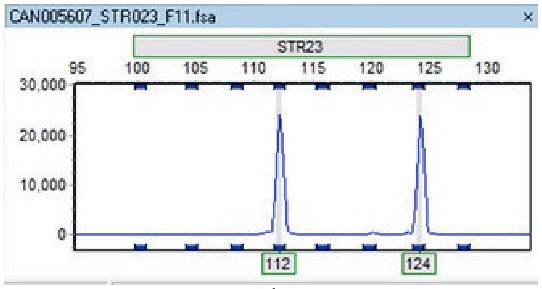

112/124

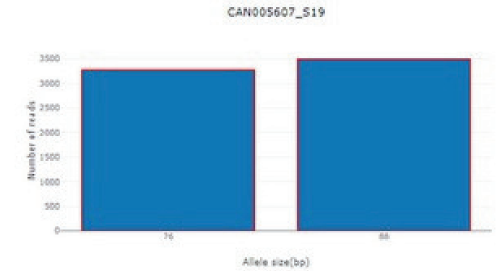

76/88

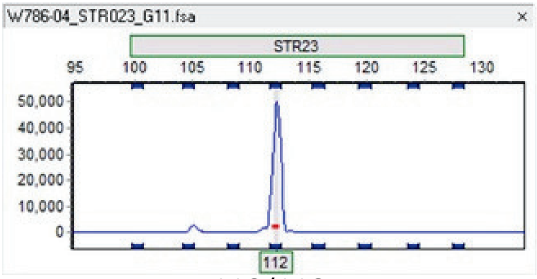

112/112

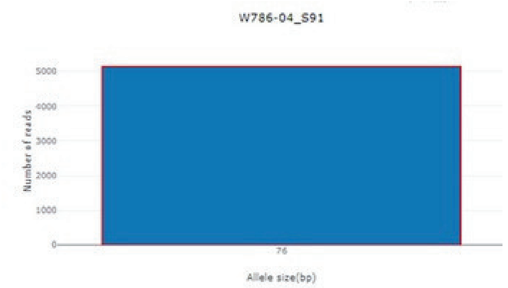

76/76

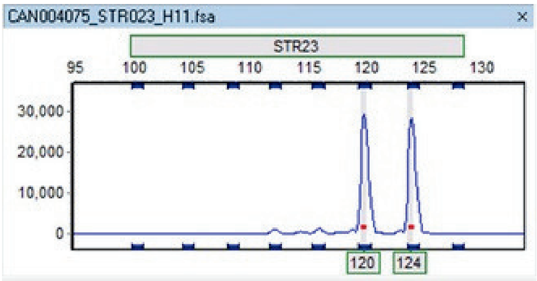

120/124

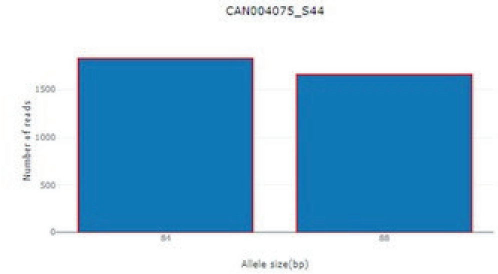

84/88

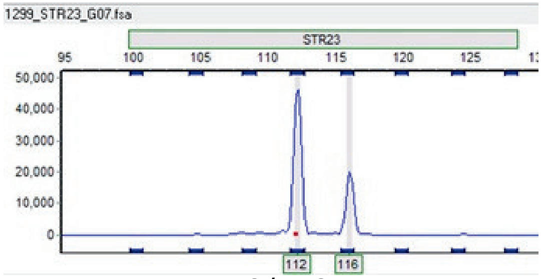

112/116

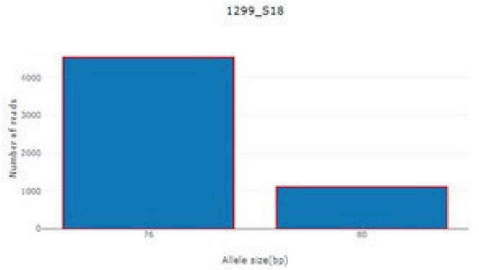

76/80

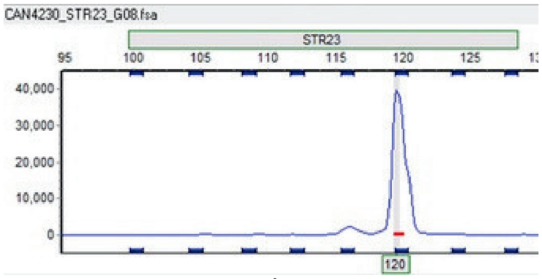

120/120

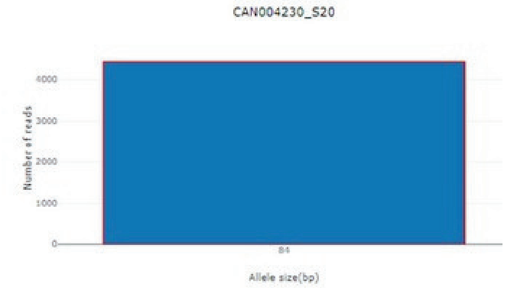

84/84

Capillary Electrophoresis - GeneMarker

Genotyping By Sequencing - SatAnalyzer

CfamSTR023 - Allele size difference - 36 bp

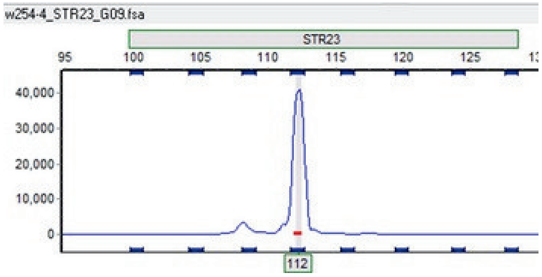

112/112

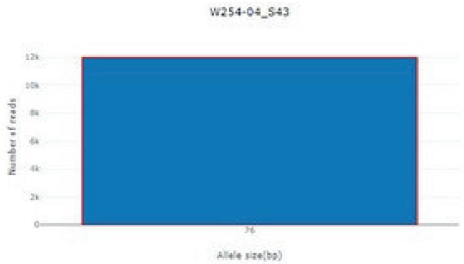

76/76

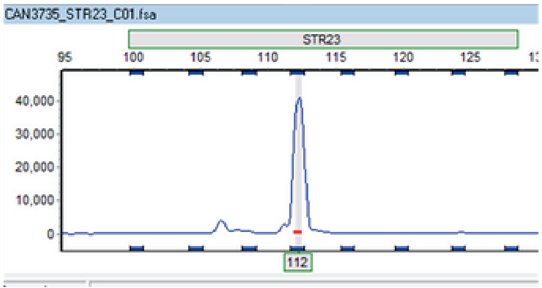

112/112

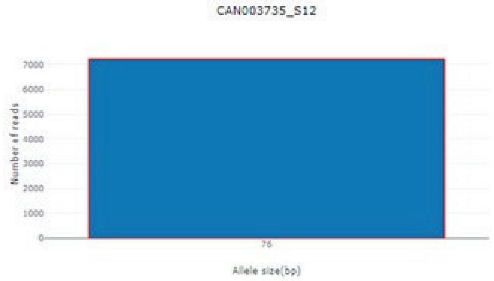

76/76

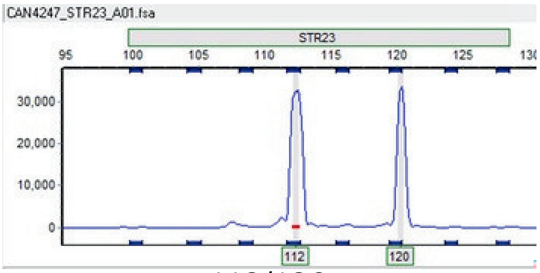

112/120

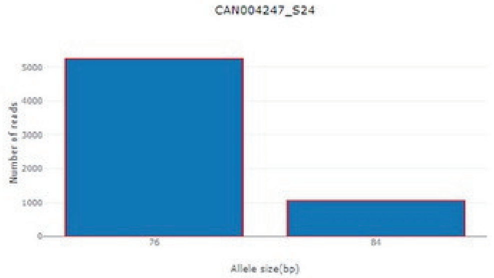

76/84

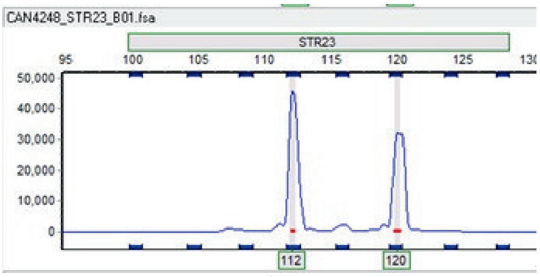

112/120

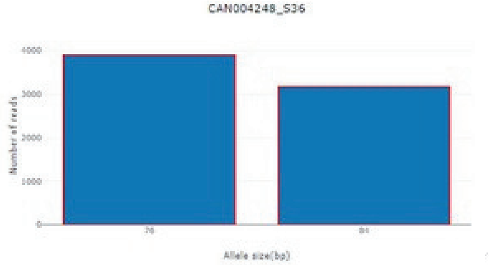

76/84

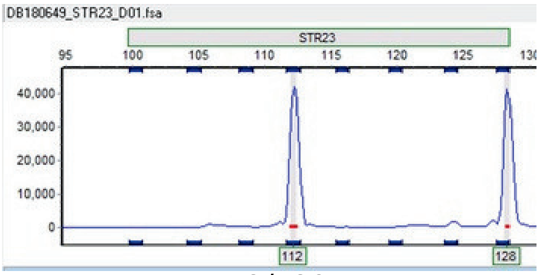

112/128

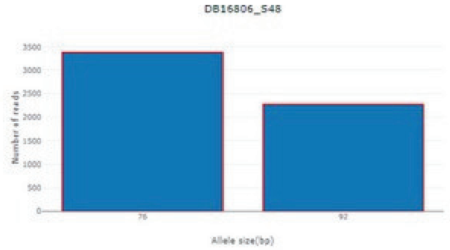

76/92

CfamSTR024 - Allele size difference - 38 bp

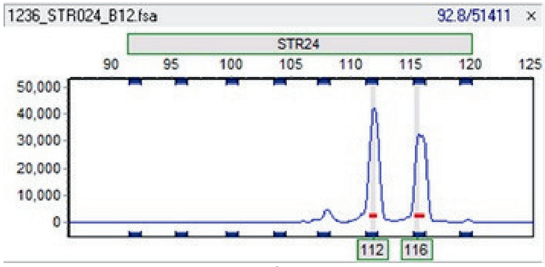

112/118

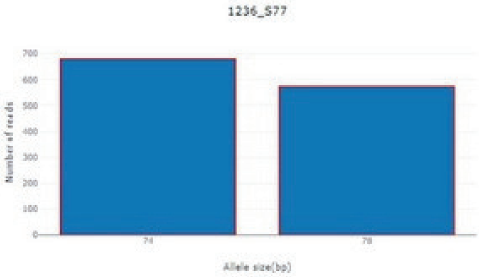

74/78

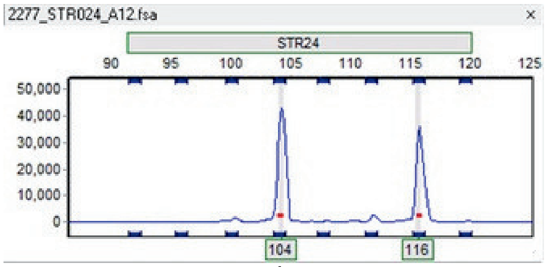

104/116

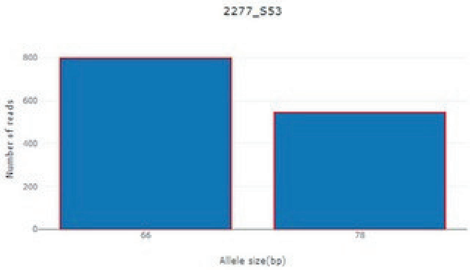

66/78

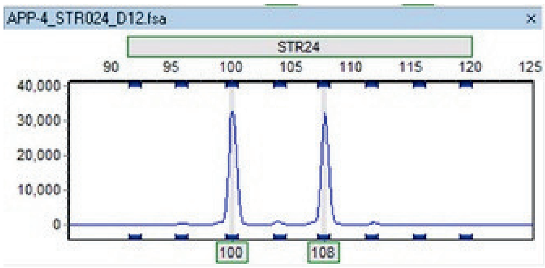

100/108

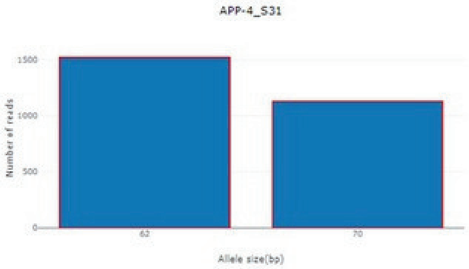

62/70

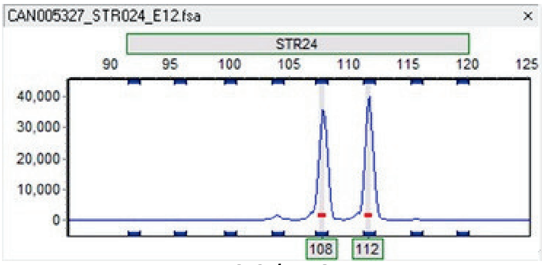

108/112

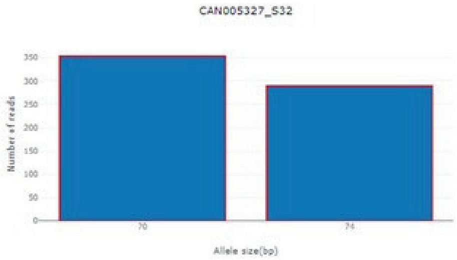

70/74

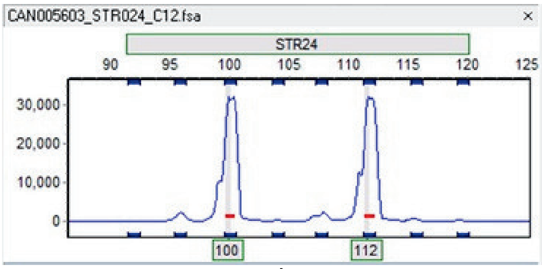

100/112

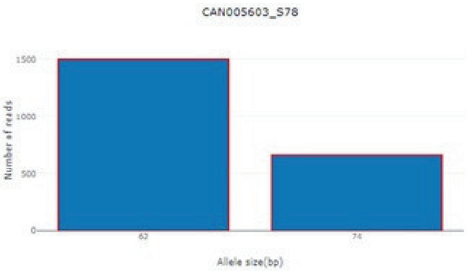

62/74

CfamSTR024 - Allele size difference - 38 bp

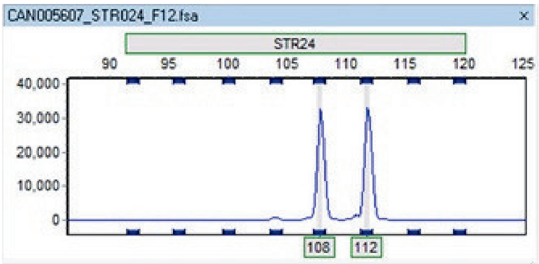

108/112

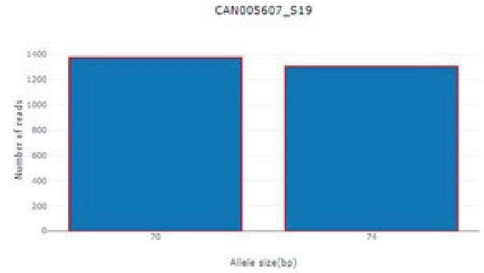

70/74

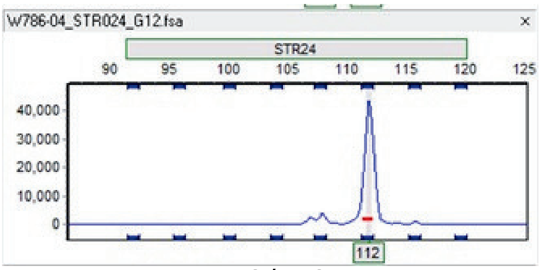

112/112

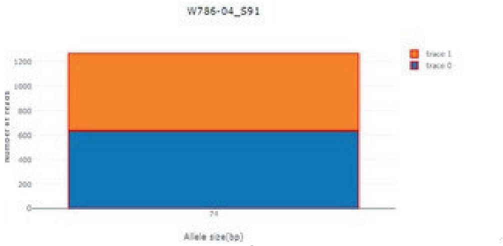

74/74*

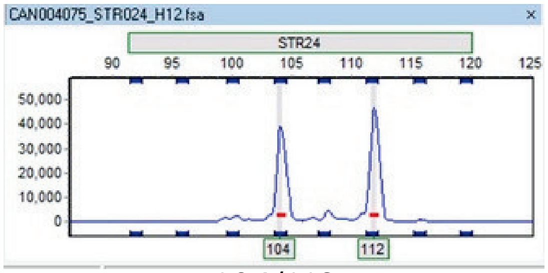

104/112

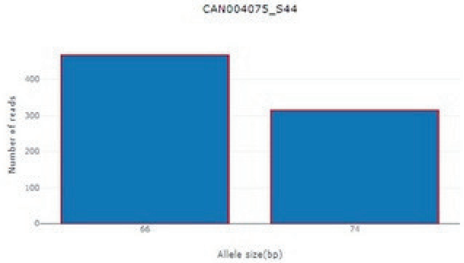

66/74

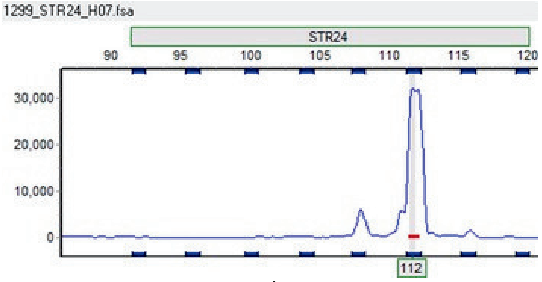

112/112

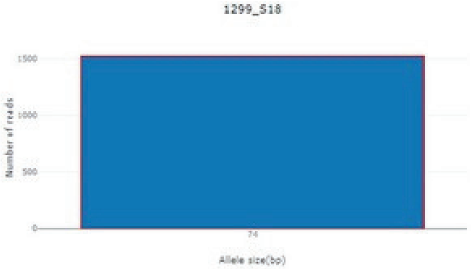

74/74

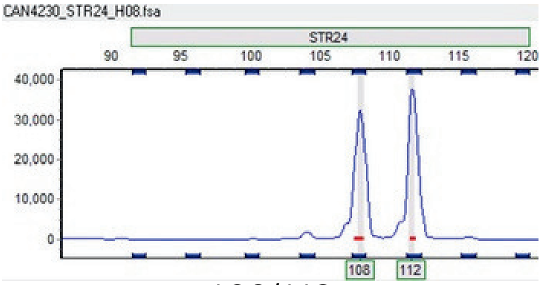

108/112

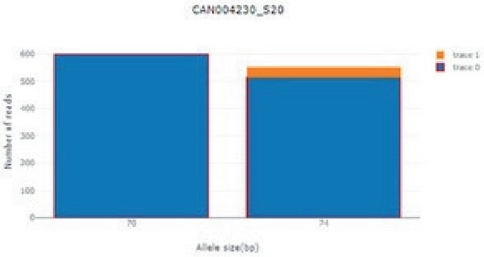

70/74

CfamSTR024 - Allele size difference - 38 bp

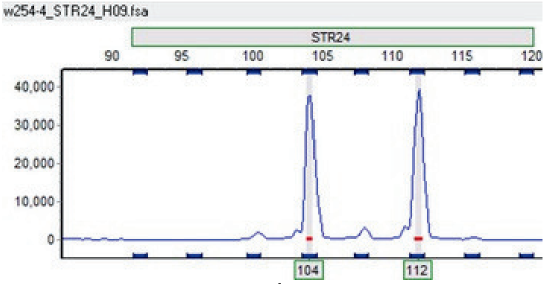

104/112

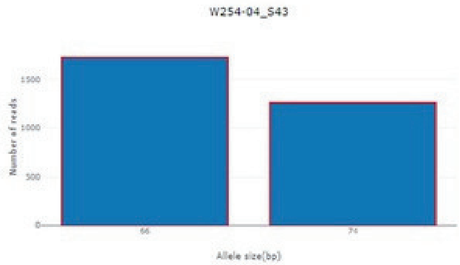

66/74

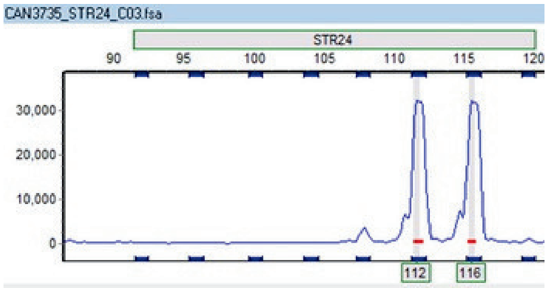

112/116

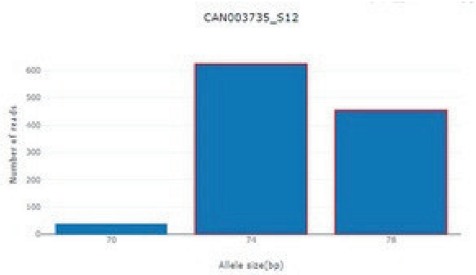

74/78

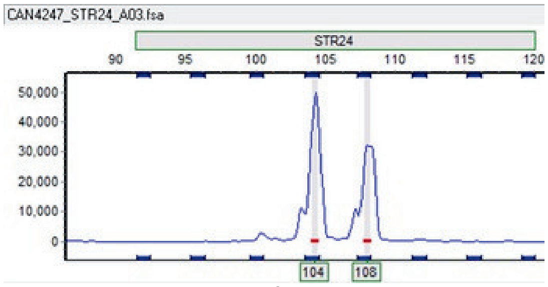

104/108

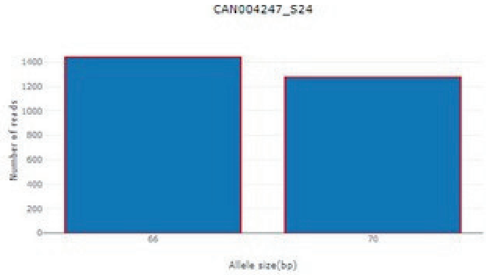

66/70

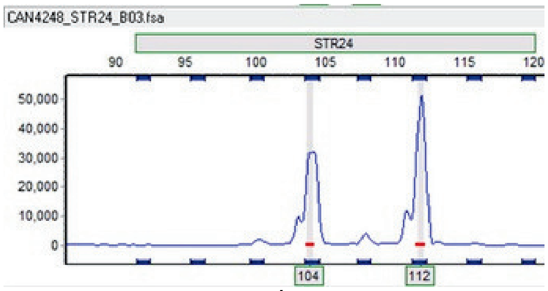

104/112

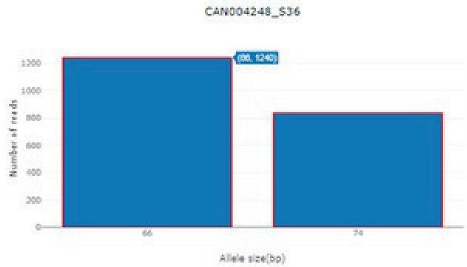

66/74

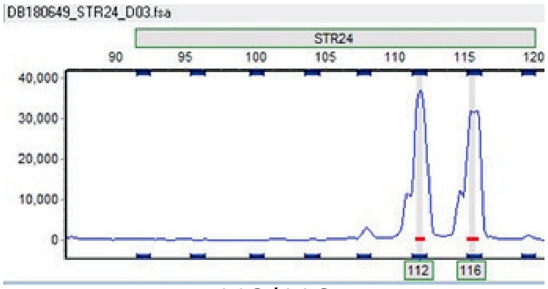

112/116

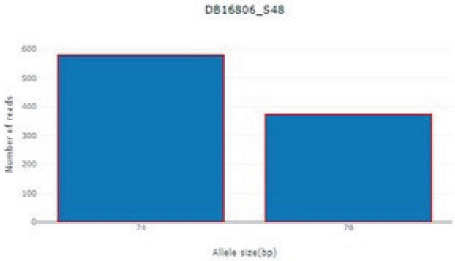

74/78

Capillary Electrophoresis - GeneMarker

Genotyping By Sequencing - SatAnalyzer

CfamSTR025 - Allele size difference - 39 or 40 bp

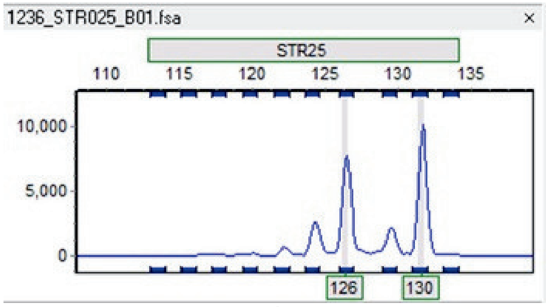

126/130

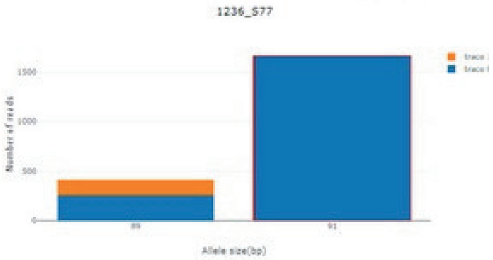

91/91

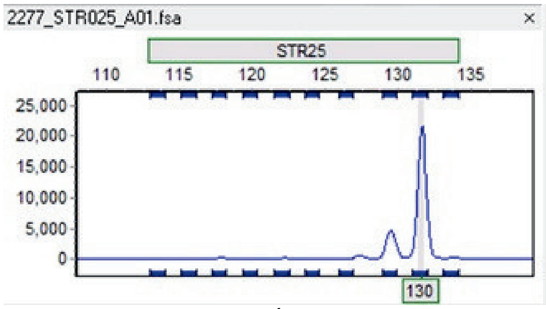

130/130

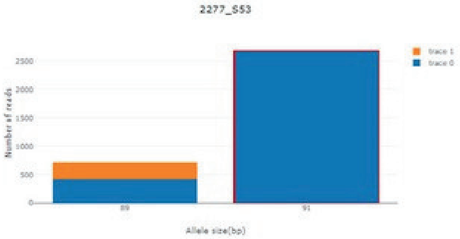

91/91

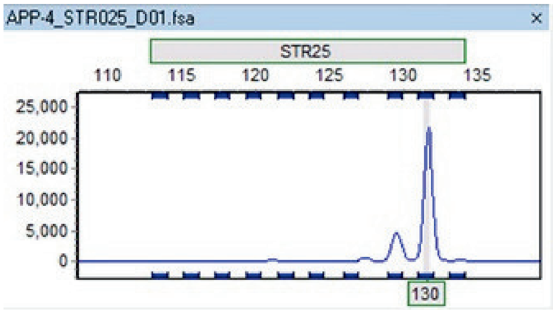

130/130

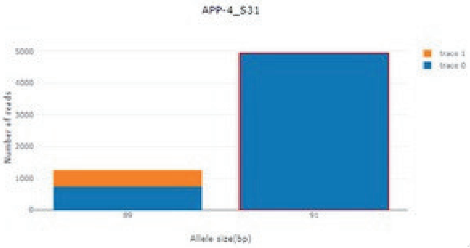

91/91

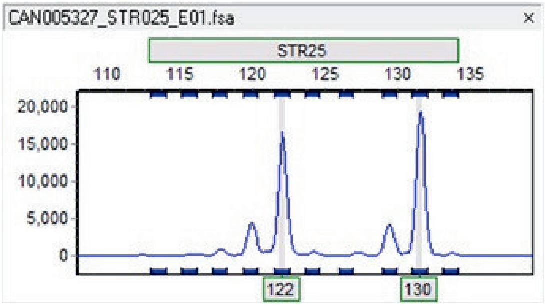

122/130

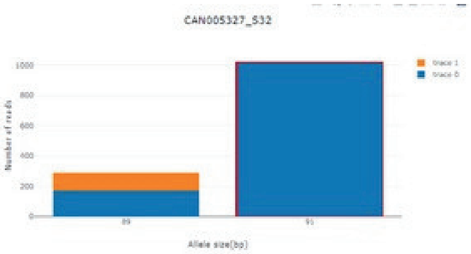

91/91

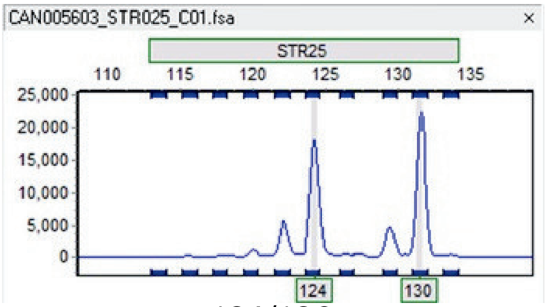

124/130

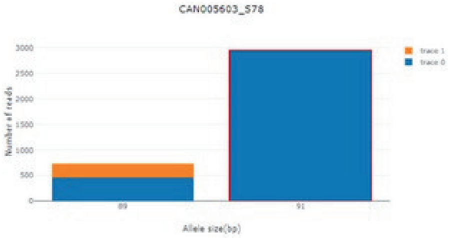

91/91

Capillary Electrophoresis - GeneMarker

Genotyping By Sequencing - SatAnalyzer

CfamSTR025 - Allele size difference - 39 or 40 bp

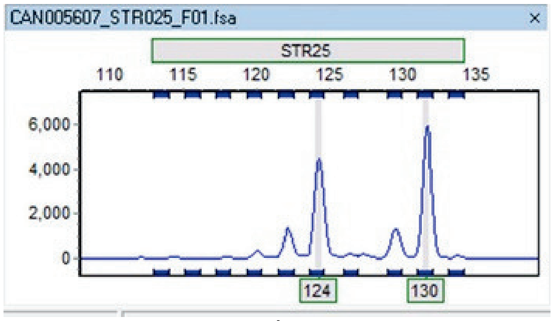

124/130

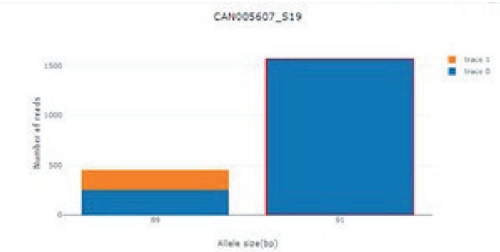

91/91

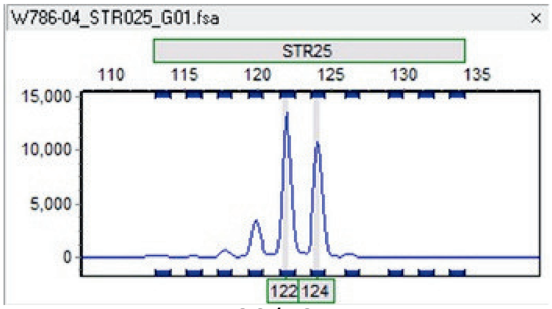

122/124

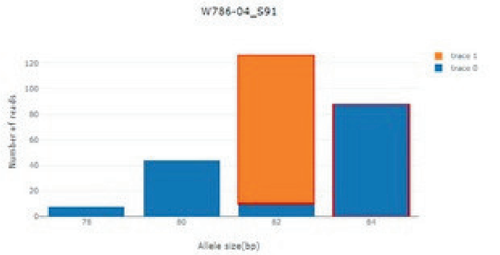

82/84

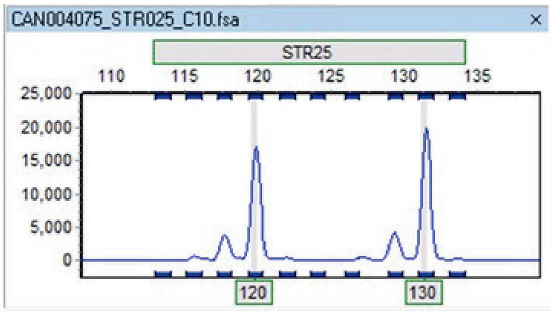

120/130

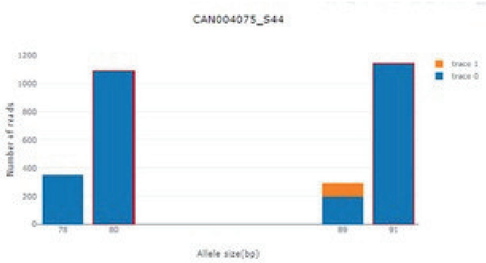

80/91

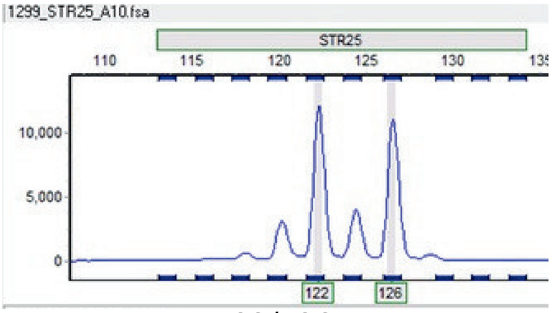

122/126

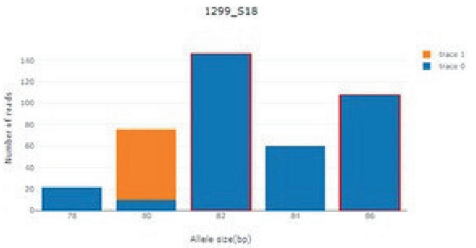

82/86

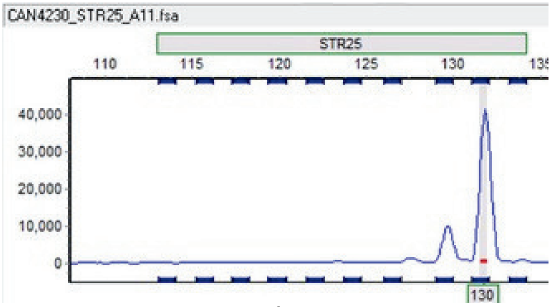

130/130

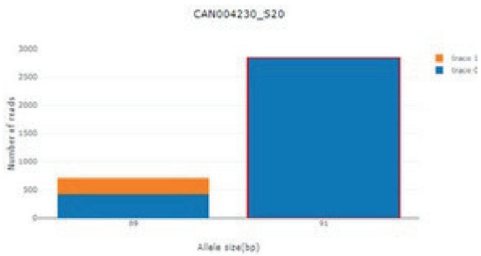

91/91

Capillary Electrophoresis - GeneMarker

Genotyping By Sequencing - SatAnalyzer

CfamSTR025 - Allele size difference - 39 or 40 bp

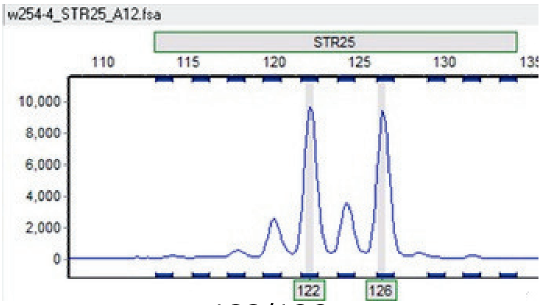

122/126

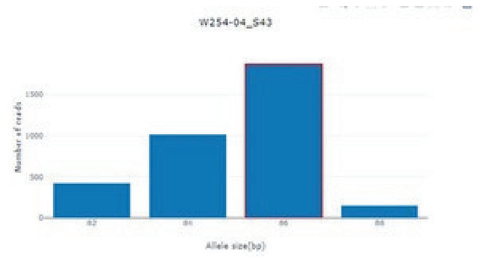

86/86

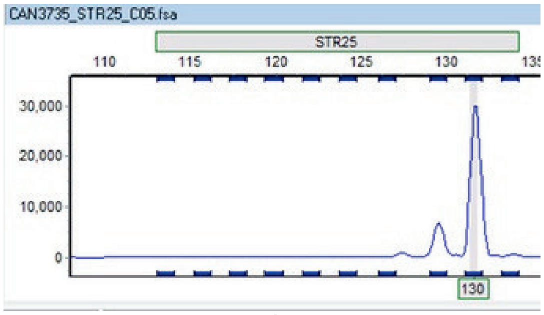

130/130

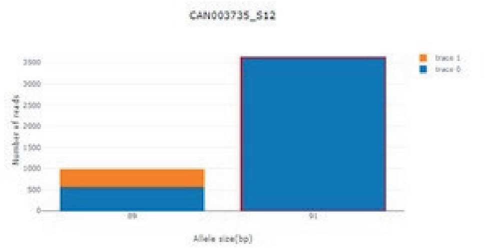

91/91

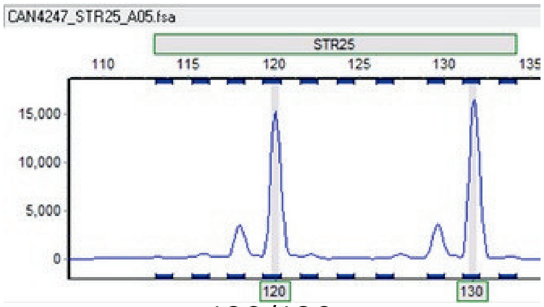

120/130

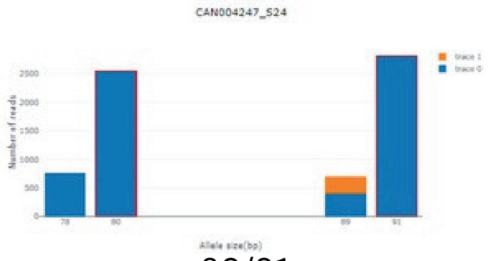

80/91

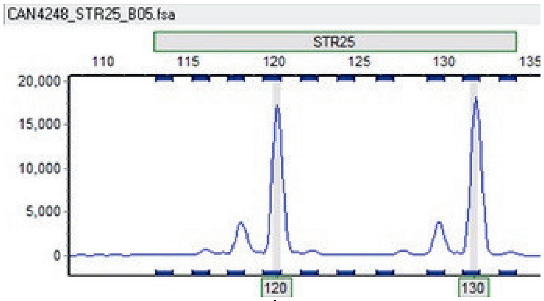

120/130

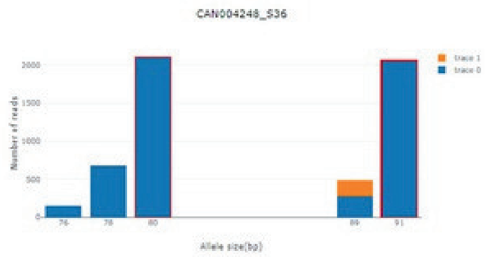

80/91

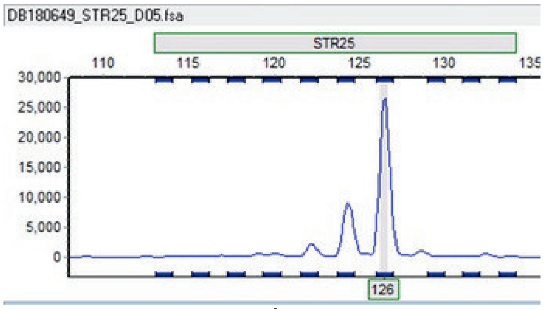

126/126

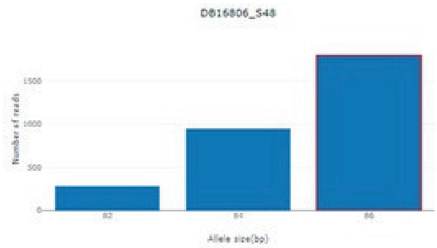

86/86

CfamSTR026 - Allele size difference - 38 bp

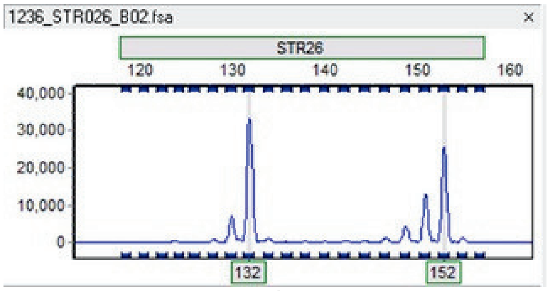

132/152

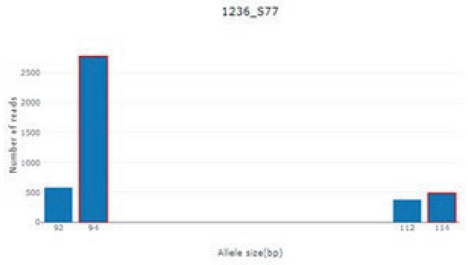

94/114

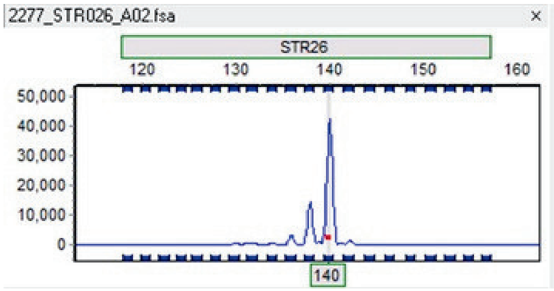

140/140

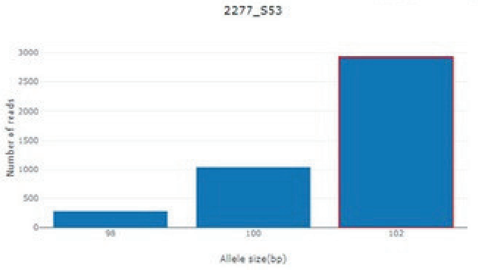

102/102

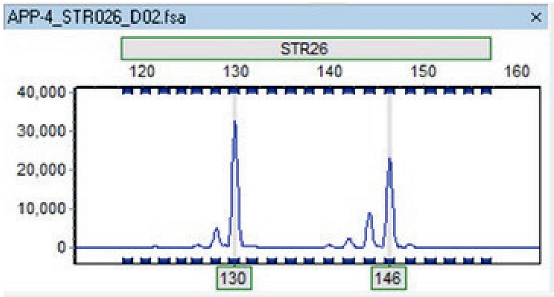

130/146

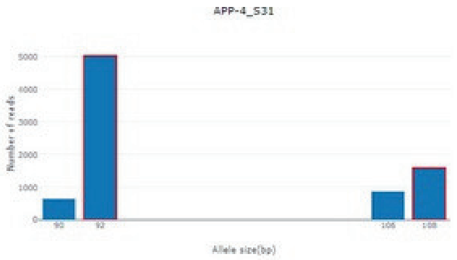

92/108

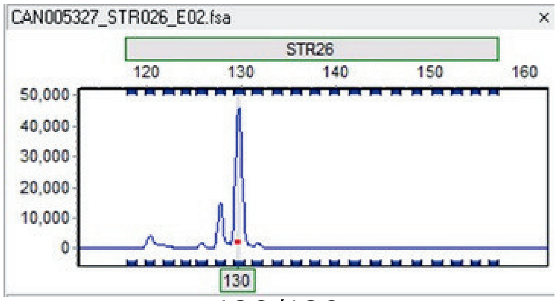

130/130

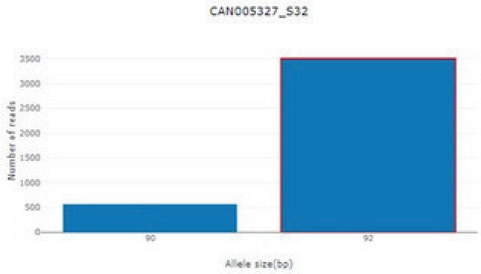

92/92

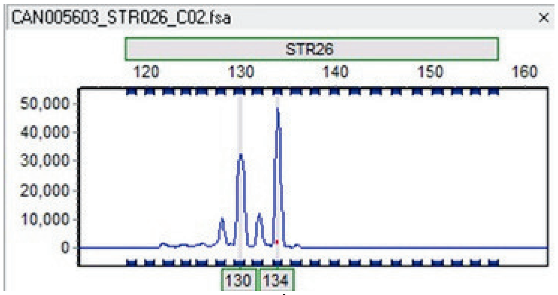

130/134

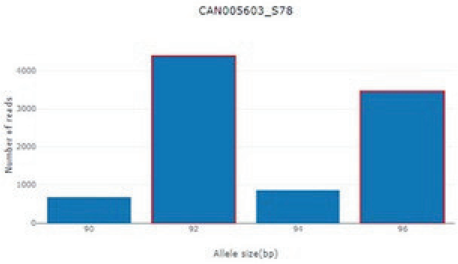

92/96

Capillary Electrophoresis - GeneMarker

Genotyping By Sequencing - SatAnalyzer

CfamSTR026 - Allele size difference - 38 bp

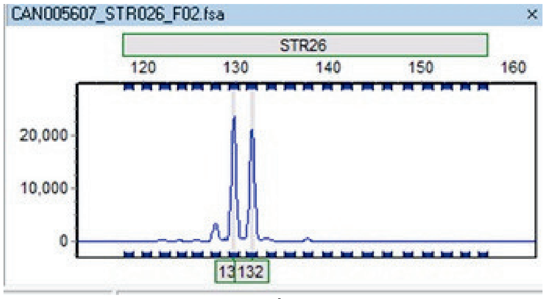

130/132

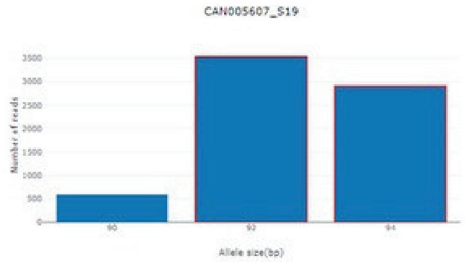

92/94

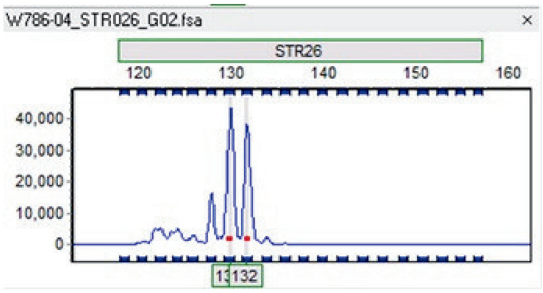

130/132

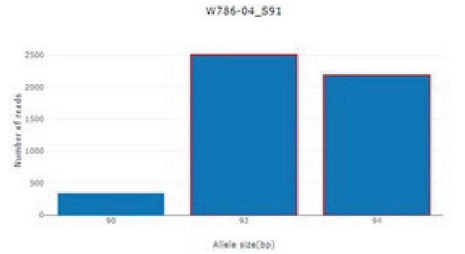

92/94

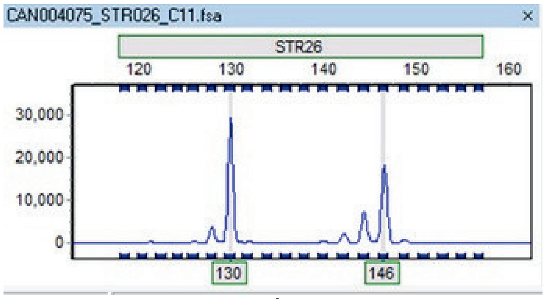

130/146

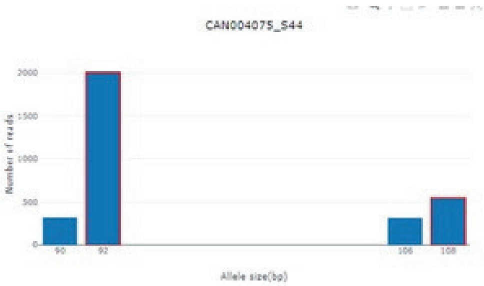

92/108

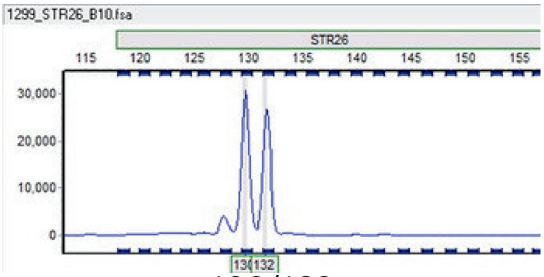

130/132

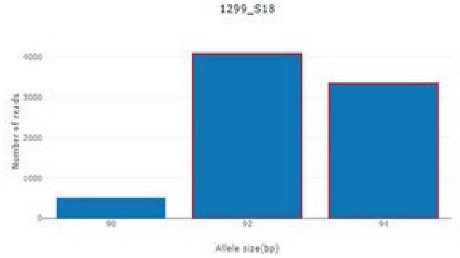

92/94

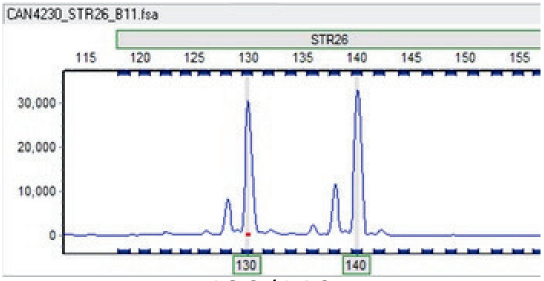

130/140

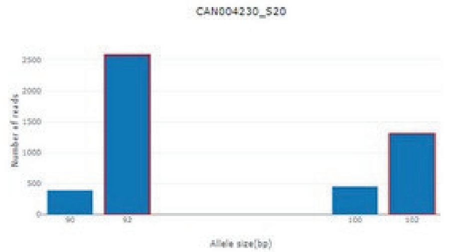

92/102

Capillary Electrophoresis - GeneMarker

Genotyping By Sequencing - SatAnalyzer

CfamSTR026 - Allele size difference - 38 bp

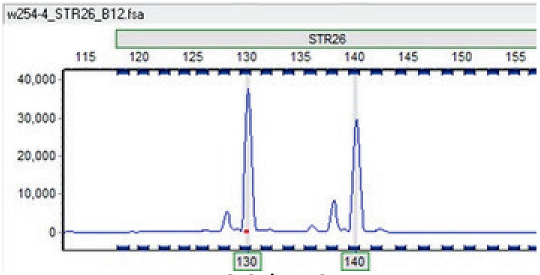

130/140

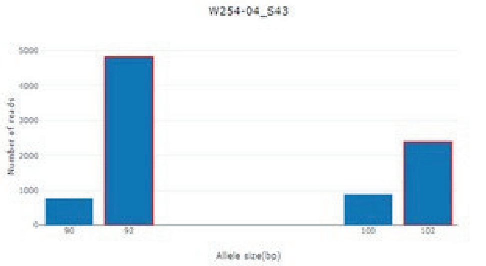

92/102

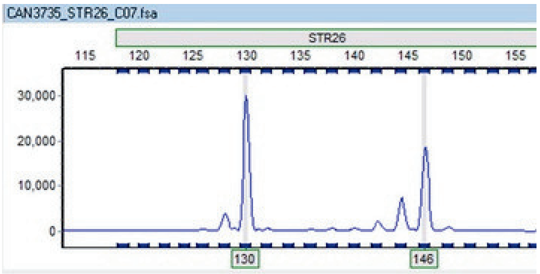

130/146

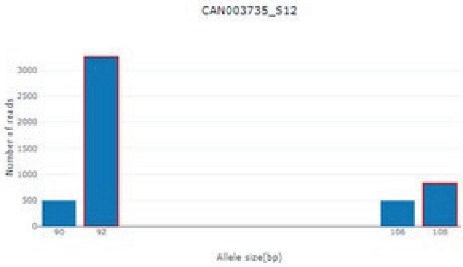

92/108

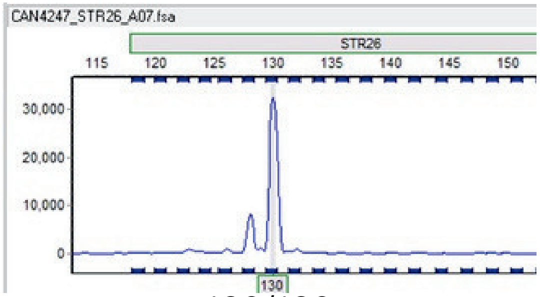

130/130

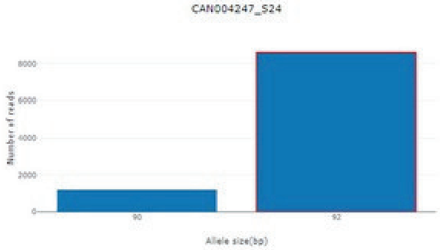

92/92

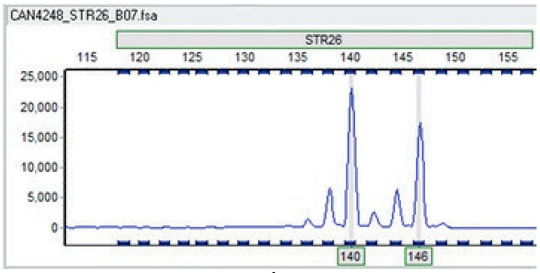

140/146

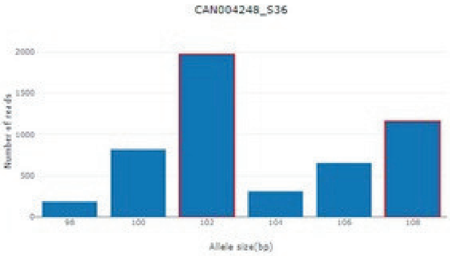

102/108

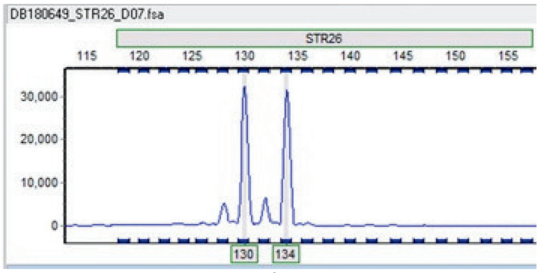

130/134

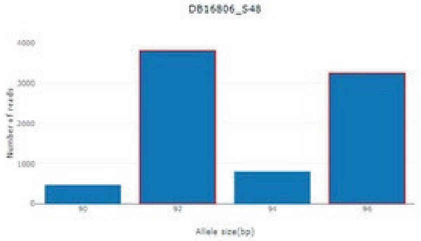

92/96

Capillary Electrophoresis - GeneMarker

Genotyping By Sequencing - SatAnalyzer

CfamSTR027 - Allele size difference - 40 bp

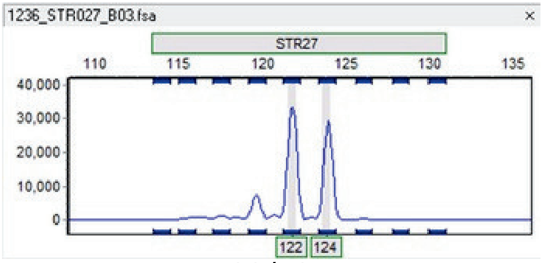

122/124

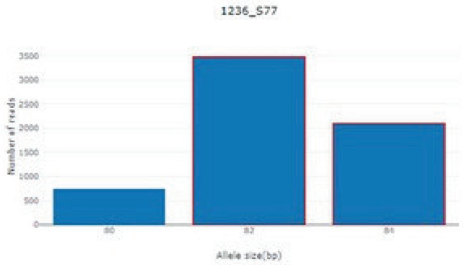

82/84

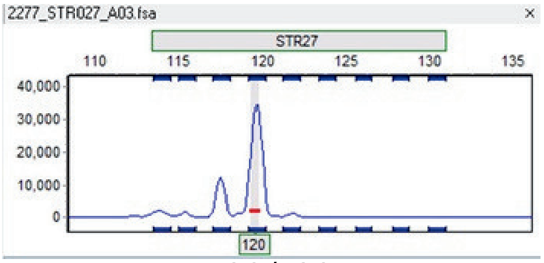

120/120

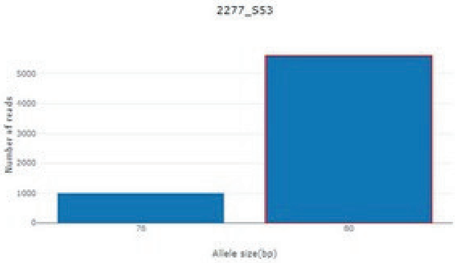

80/80

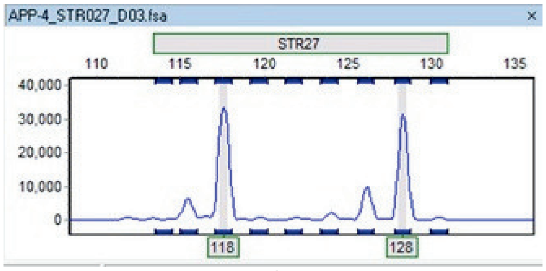

118/128

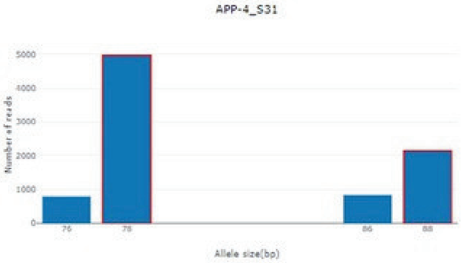

78/88

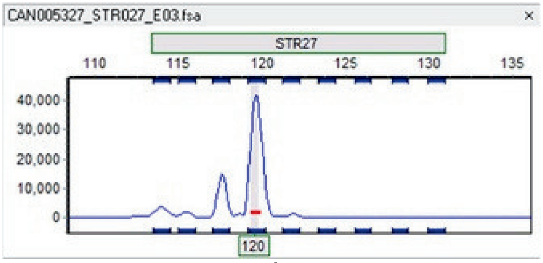

120/120

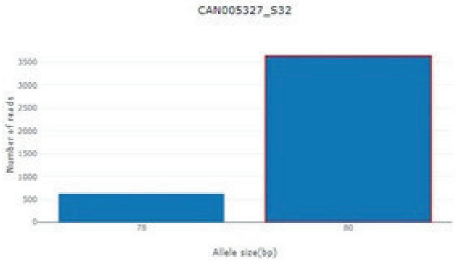

80/80

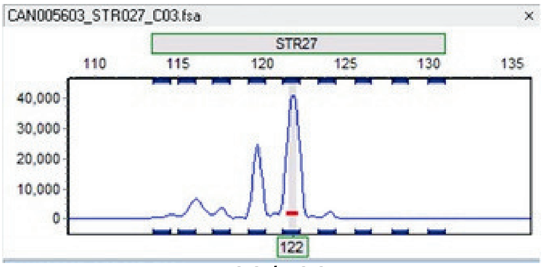

122/122

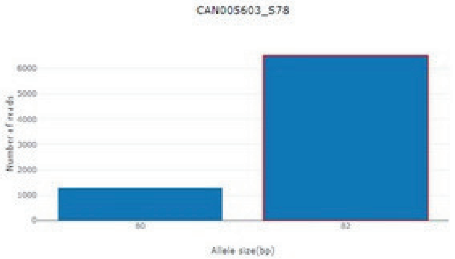

82/82

Capillary Electrophoresis - GeneMarker

Genotyping By Sequencing - SatAnalyzer

CfamSTR027 - Allele size difference - 40 bp

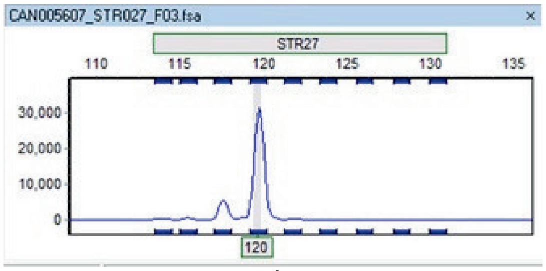

120/120

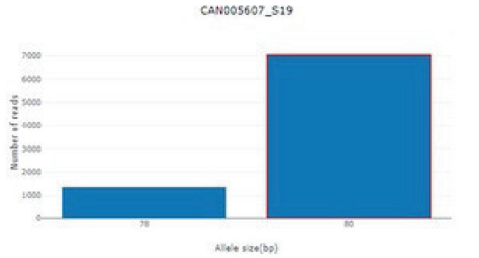

80/80

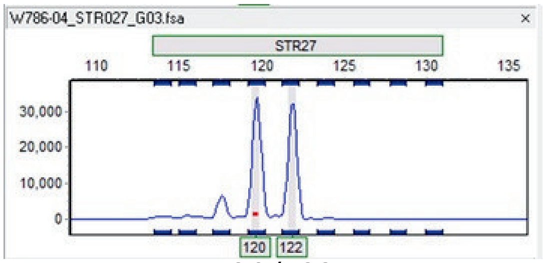

120/122

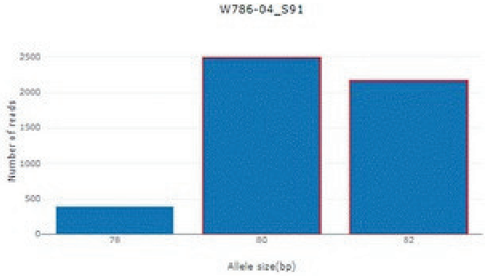

80/82

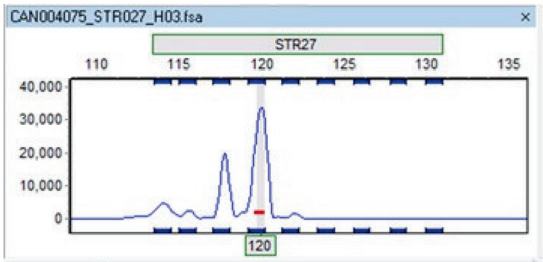

120/120

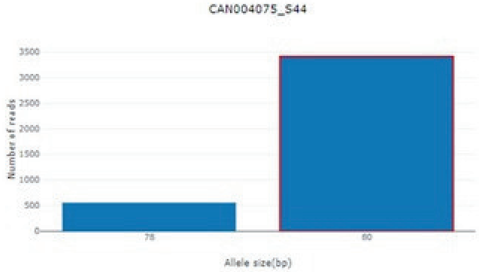

80/80

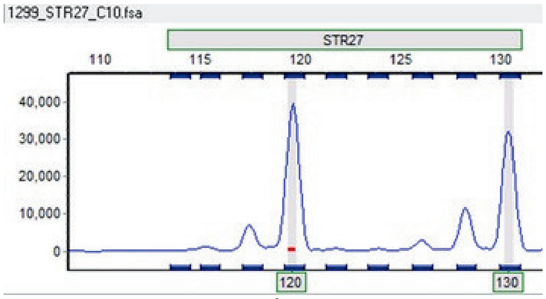

120/130

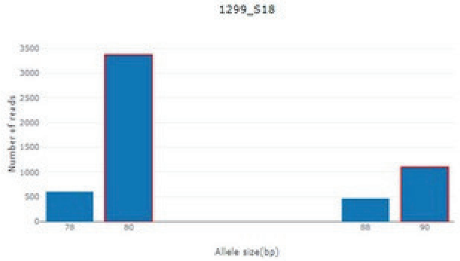

80/90

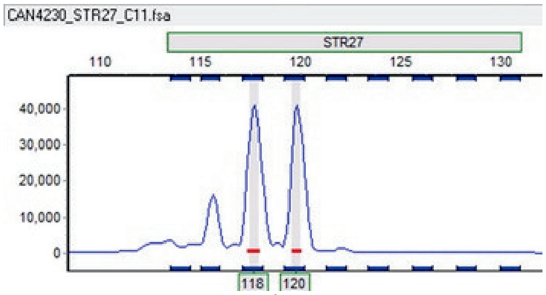

118/120

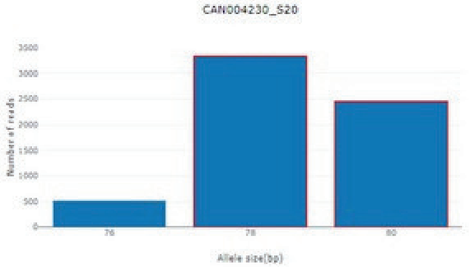

78/80

Capillary Electrophoresis - GeneMarker

Genotyping By Sequencing - SatAnalyzer

CfamSTR027 - Allele size difference - 40 bp

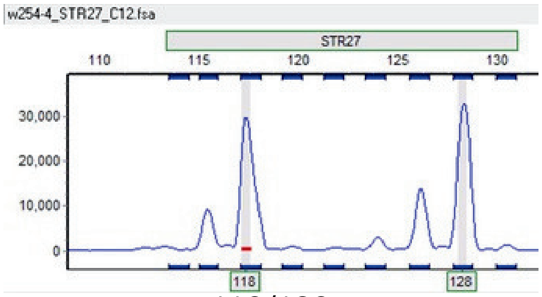

118/128

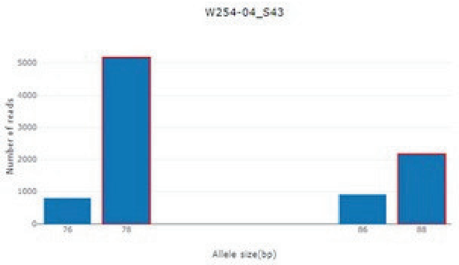

78/88

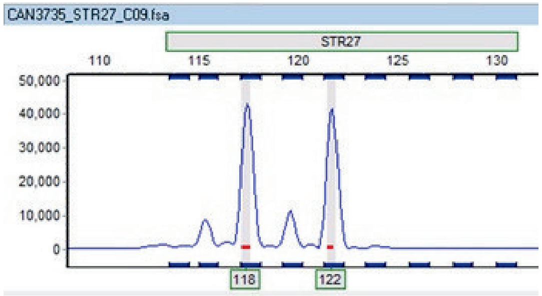

118/122

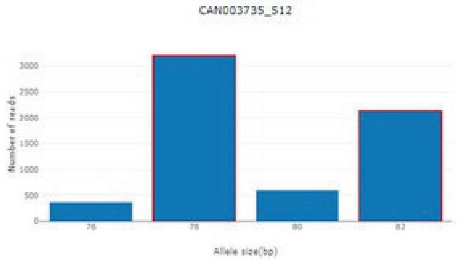

78/82

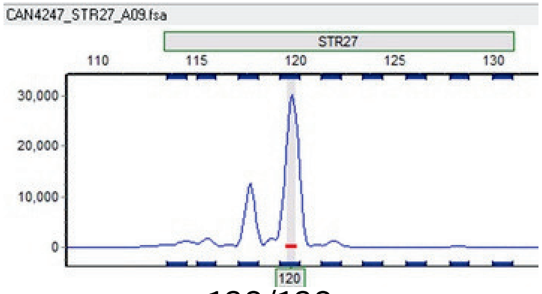

120/120

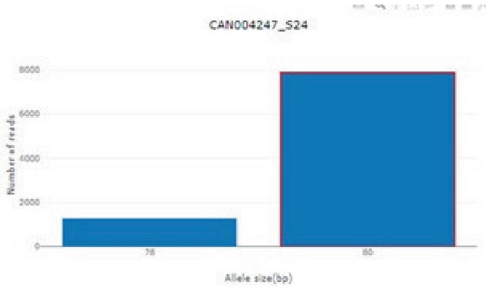

80/80

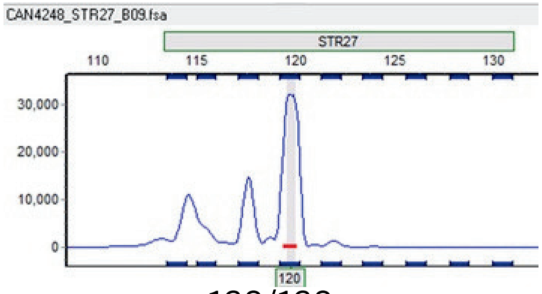

120/120

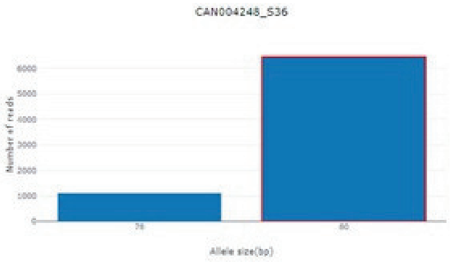

80/80

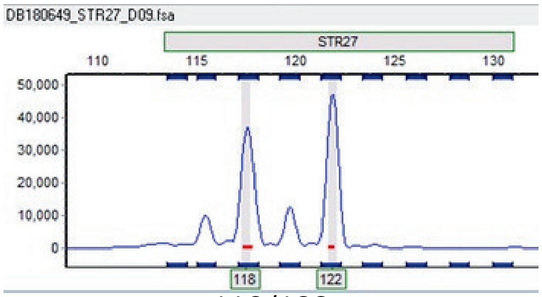

118/122

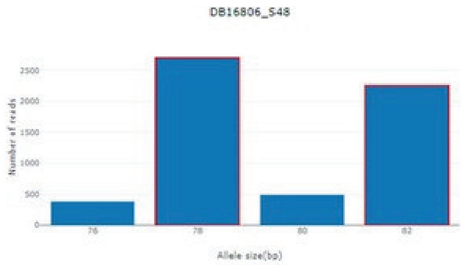

78/82

Capillary Electrophoresis - GeneMarker

Genotyping By Sequencing - SatAnalyzer

CfamSTR029 - Allele size difference - 37 bp

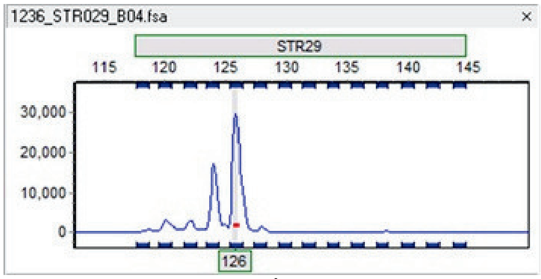

126/126

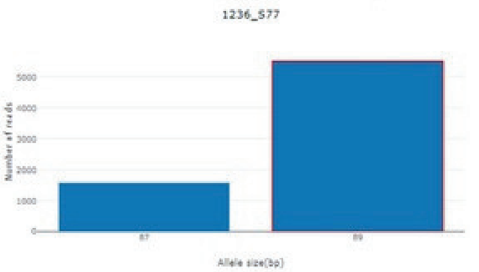

89/89

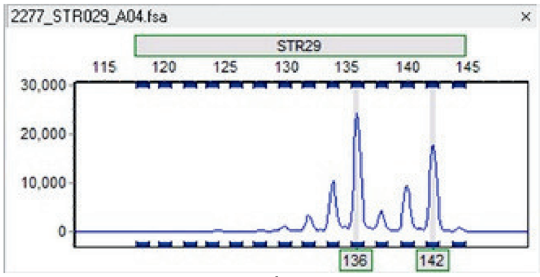

136/142

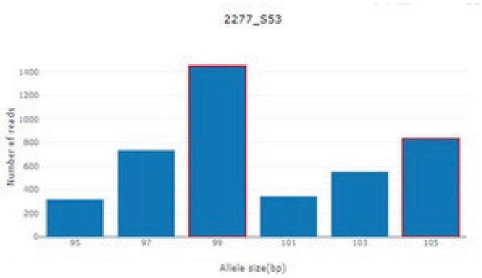

99/105

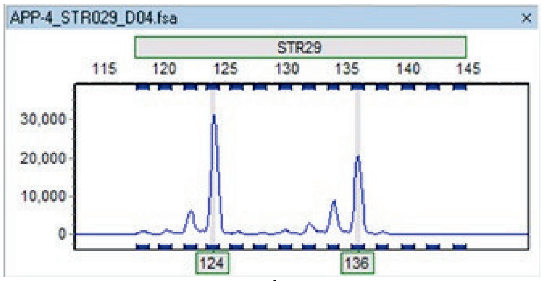

124/136

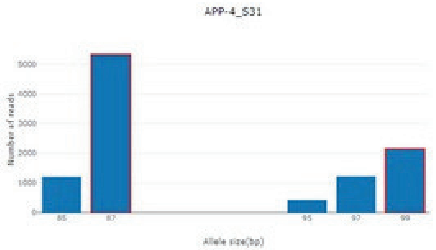

87/99

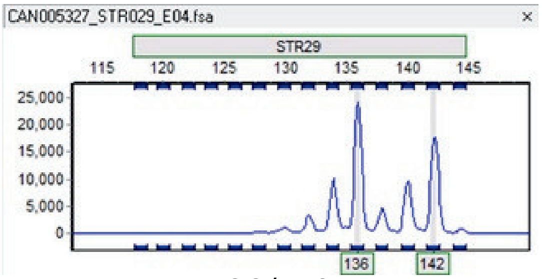

136/142

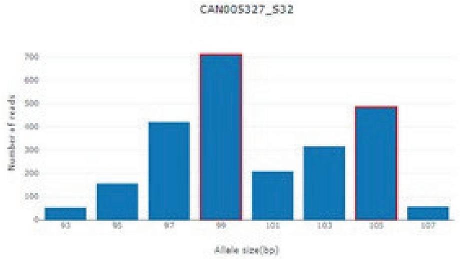

99/105

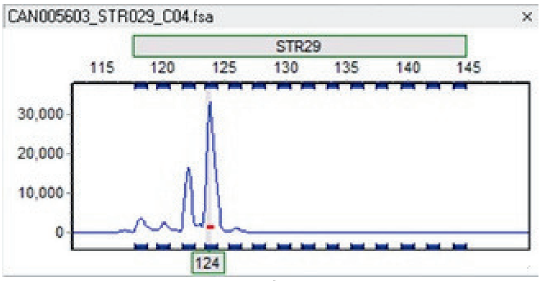

124/124

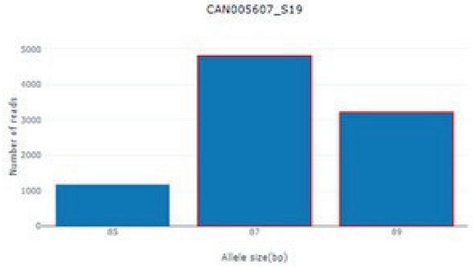

87/89

Capillary Electrophoresis - GeneMarker

Genotyping By Sequencing - SatAnalyzer

CfamSTR029 - Allele size difference - 37 bp

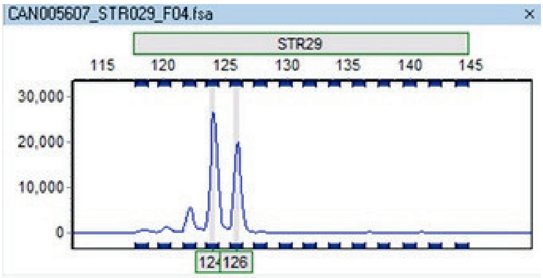

124/126

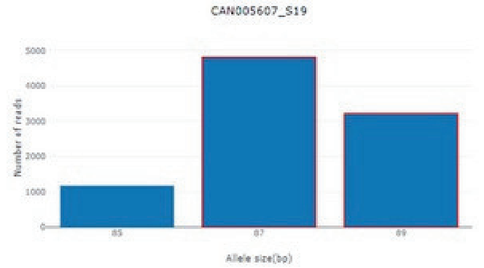

87/89

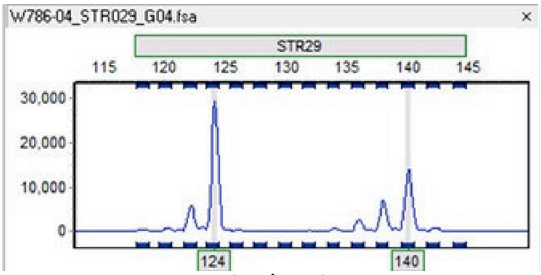

124/140

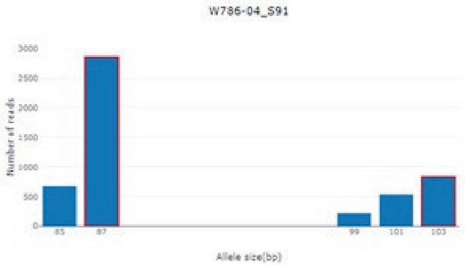

87/103

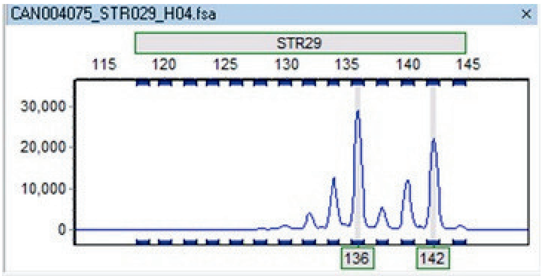

136/142

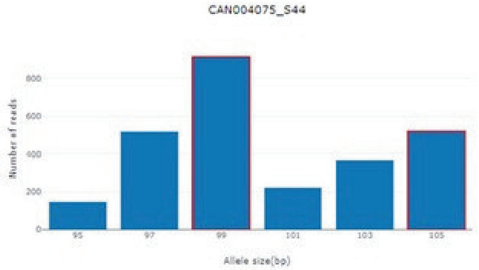

99/105

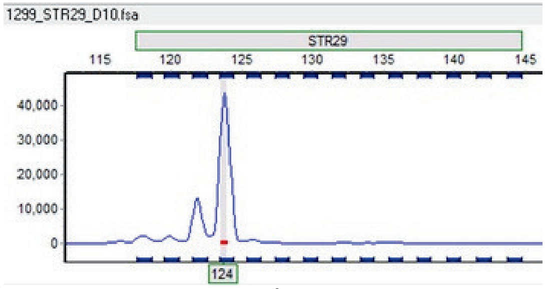

124/124

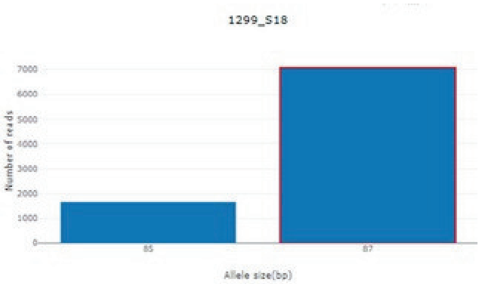

87/87

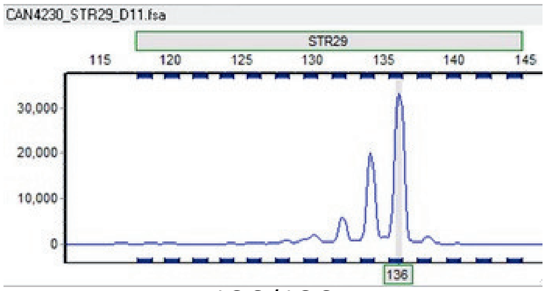

136/136

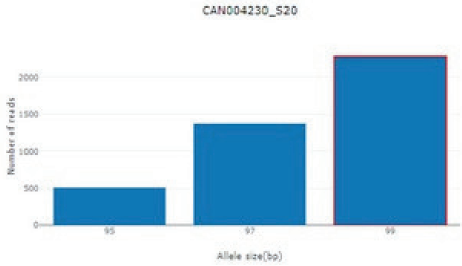

99/99

Capillary Electrophoresis - GeneMarker

Genotyping By Sequencing - SatAnalyzer

CfamSTR029 - Allele size difference - 37 bp

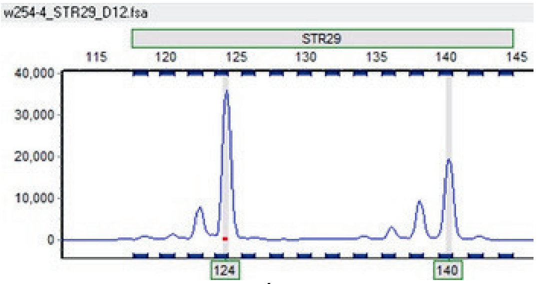

124/140

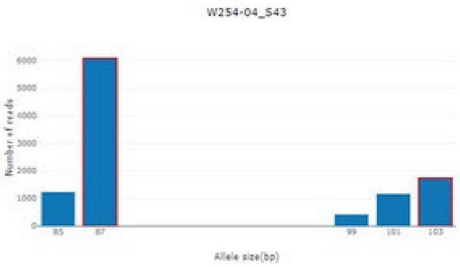

87/103

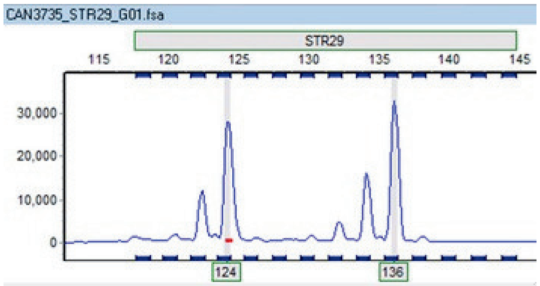

124/136

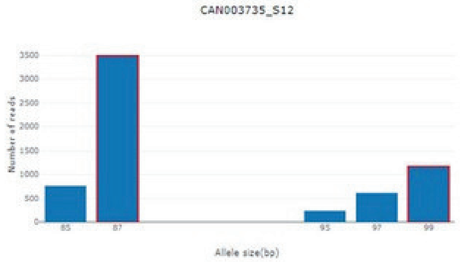

87/99

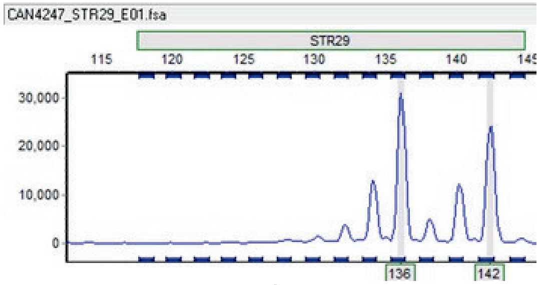

136/142

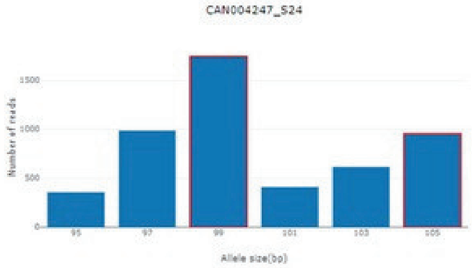

99/105

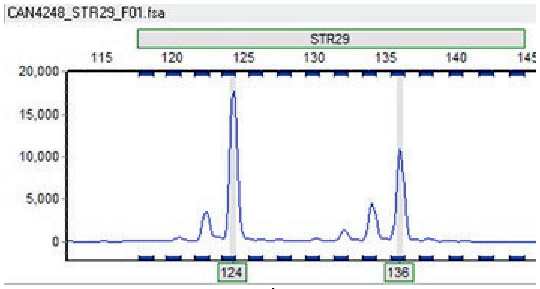

124/136

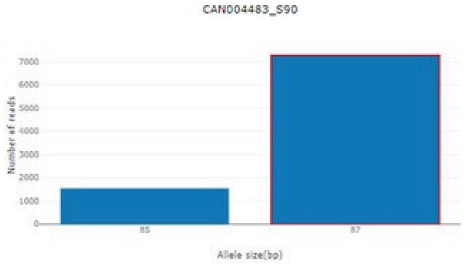

87/99

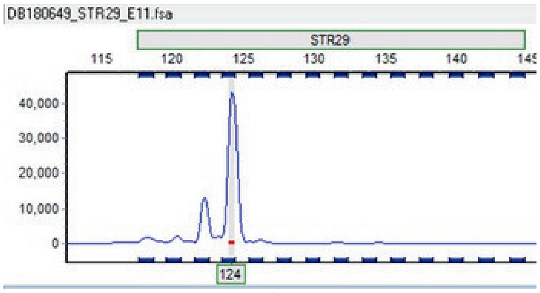

124/124

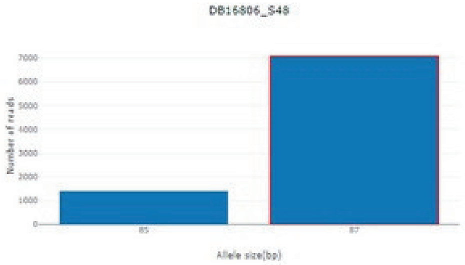

87/87

CfamSTR030 - Allele size difference - 37 bp

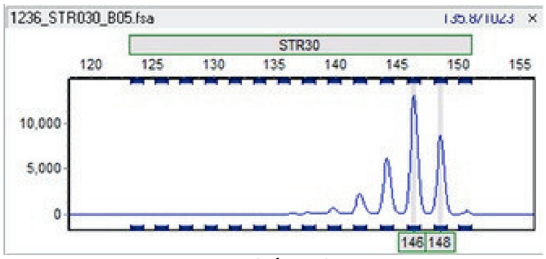

146/148

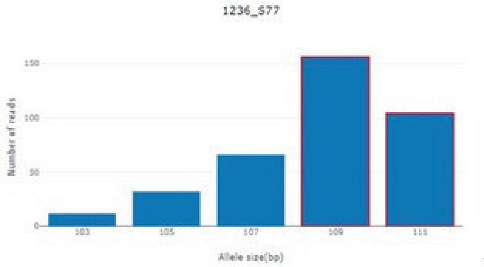

109/101

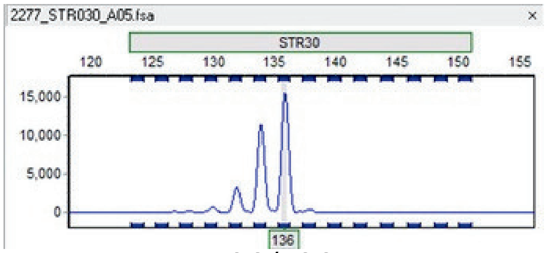

136/136

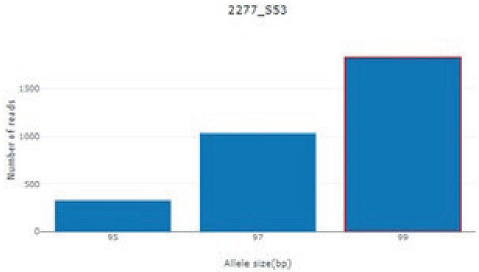

99/99

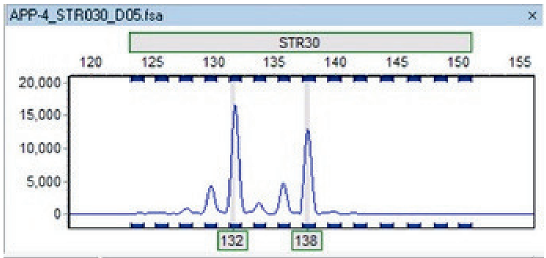

132/138

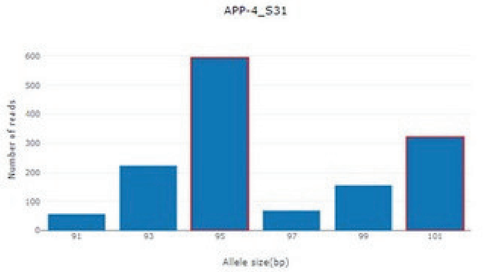

95/101

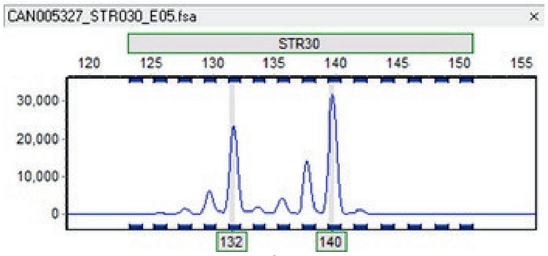

132/140

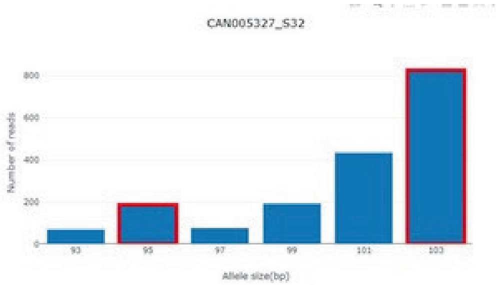

95/103

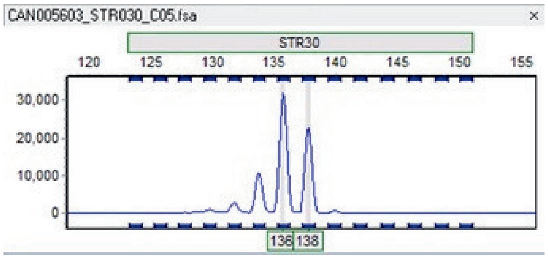

136/138

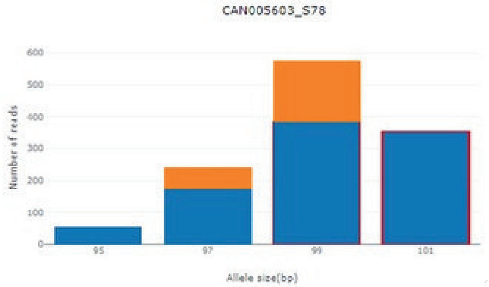

99/101

Capillary Electrophoresis - GeneMarker

Genotyping By Sequencing - SatAnalyzer

CfamSTR030 - Allele size difference - 37 bp

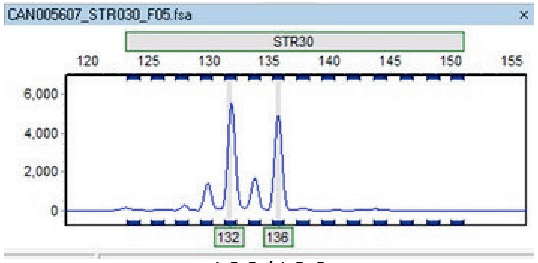

132/136

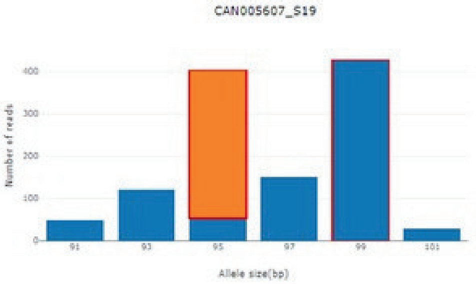

95/99

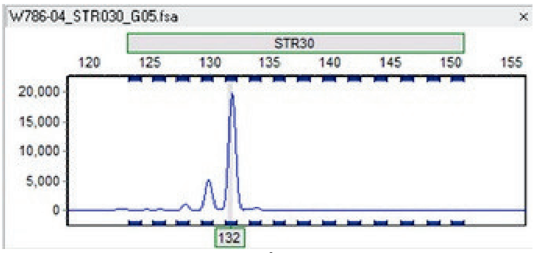

132/132

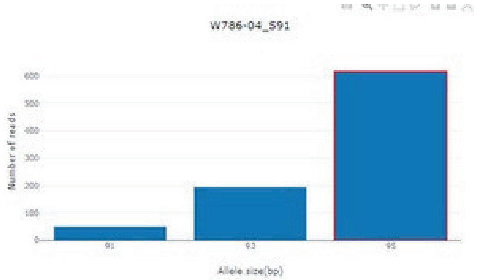

95/95

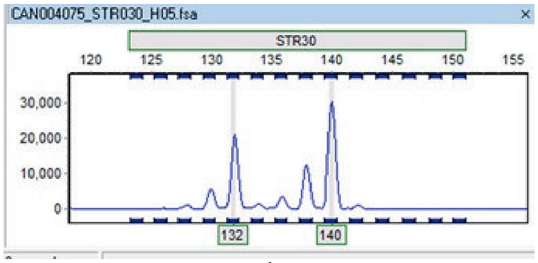

132/140

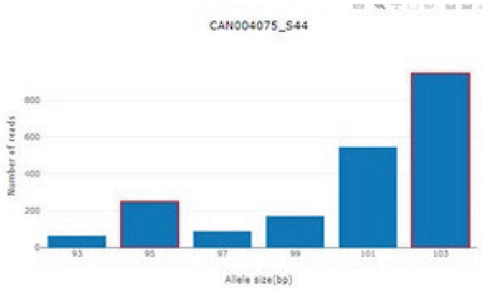

95/103

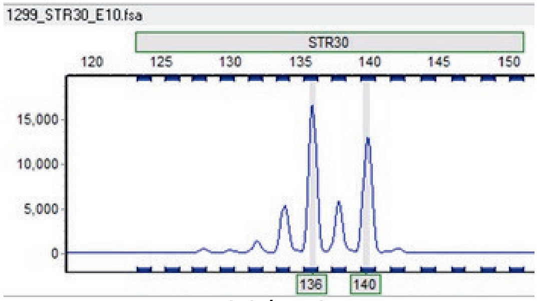

136/140

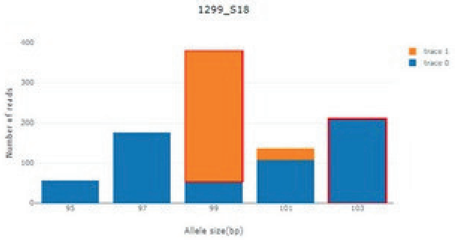

99/103

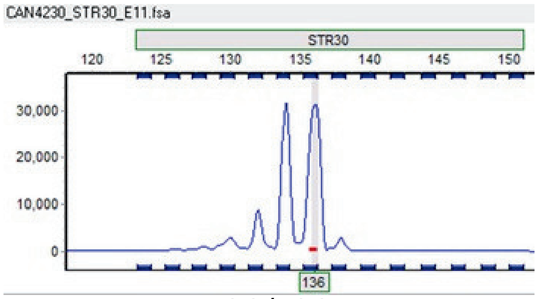

136/136

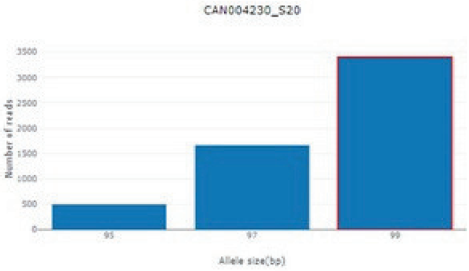

99/99

Capillary Electrophoresis - GeneMarker | Genotyping By Sequencing - SatAnalyzer

CfamSTR030 - Allele size difference - 37 bp

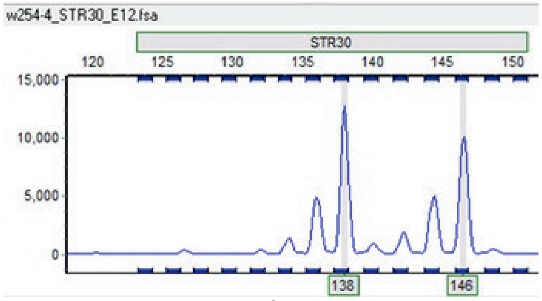

138/146

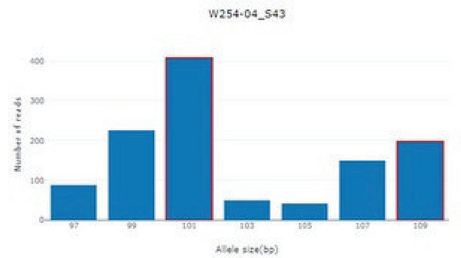

101/109

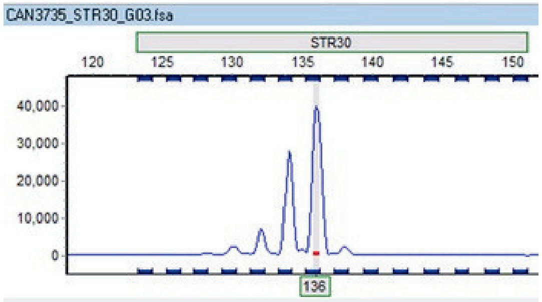

136/136

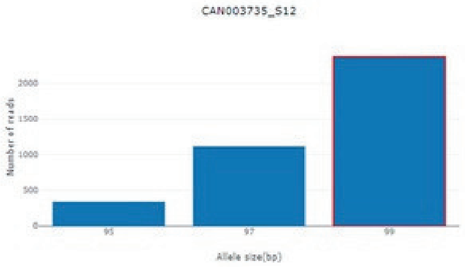

99/99

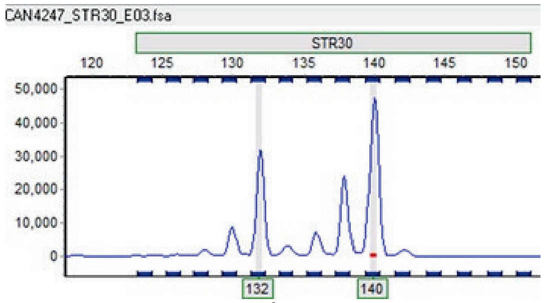

132/140

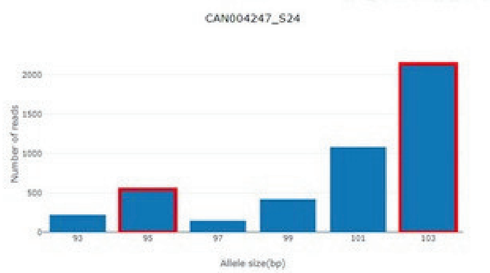

95/103

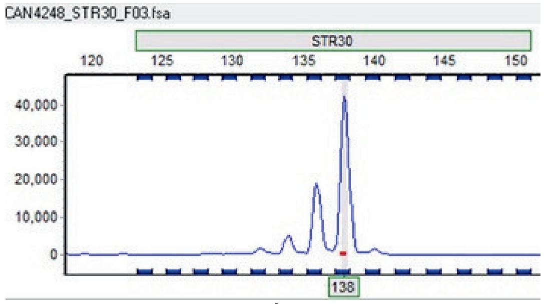

138/138

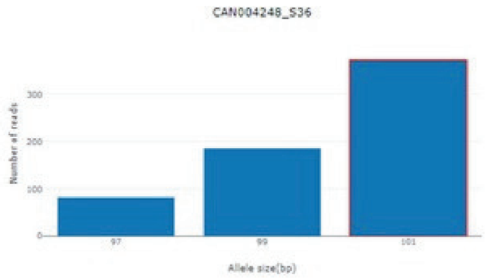

101/101

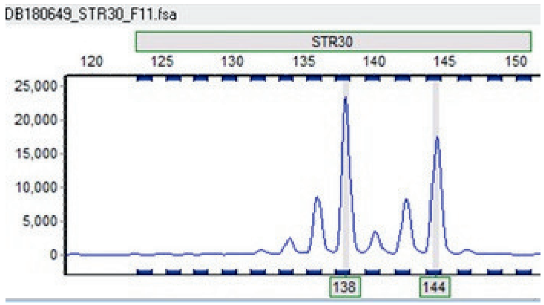

138/144

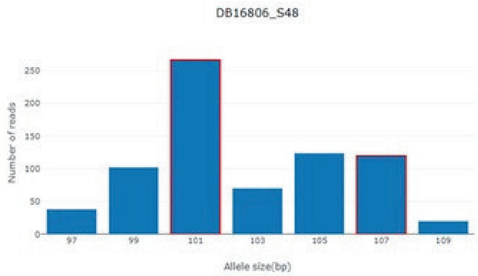

101/107

Capillary Electrophoresis - GeneMarker

Genotyping By Sequencing - SatAnalyzer

CfamSTR031 - Allele size difference - 41 bp

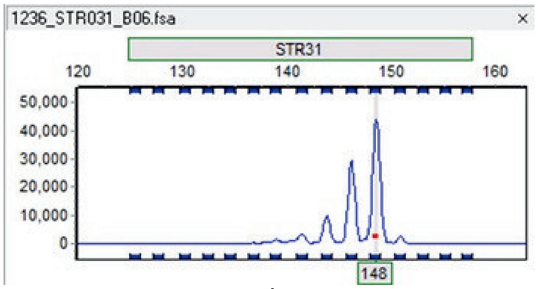

148/148

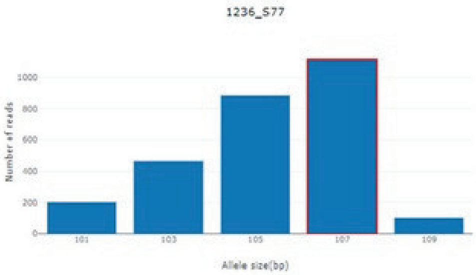

107/107

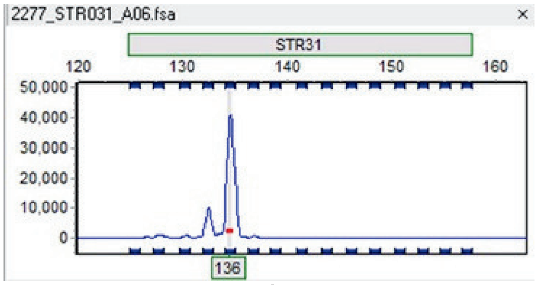

136/136

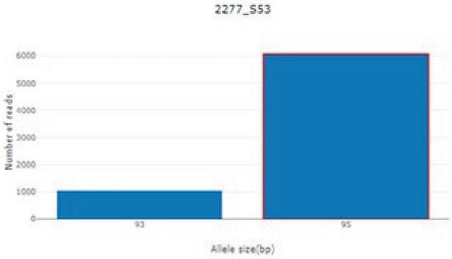

95/95

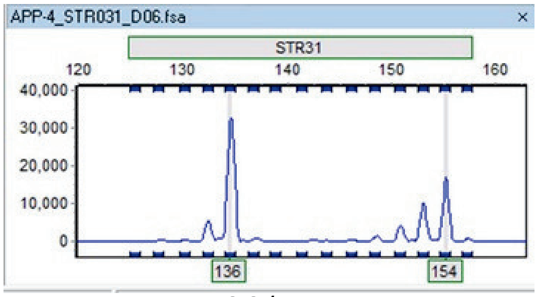

136/154

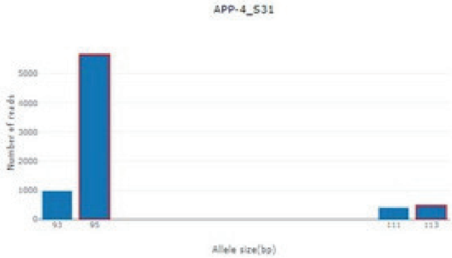

95/113

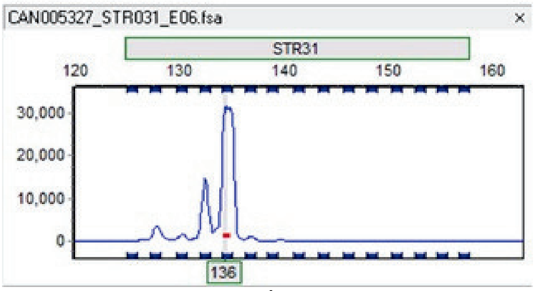

136/136

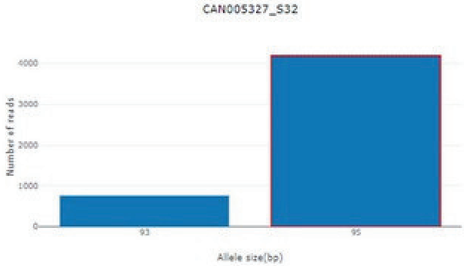

95/95

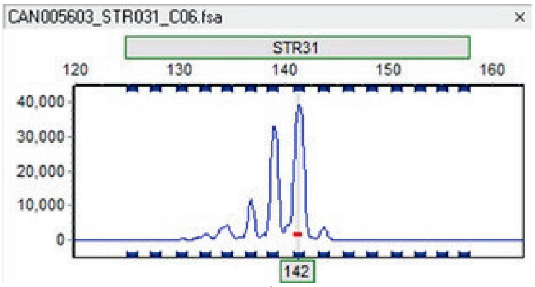

142/142

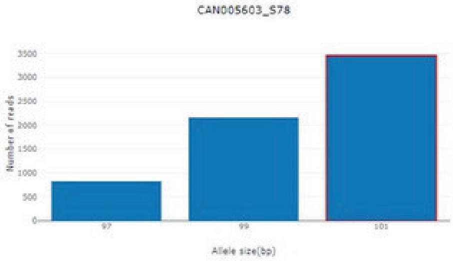

101/101

Capillary Electrophoresis - GeneMarker

Genotyping By Sequencing - SatAnalyzer

CfamSTR031 - Allele size difference - 41 bp

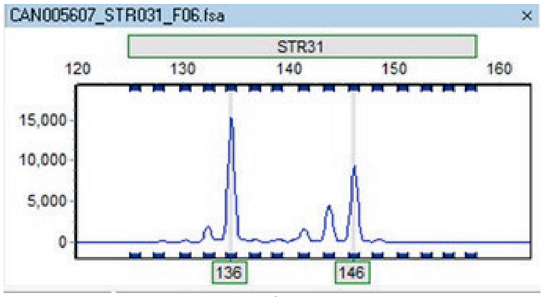

136/146

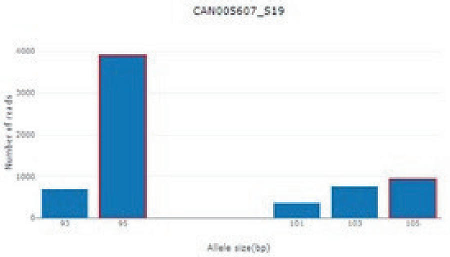

95/105

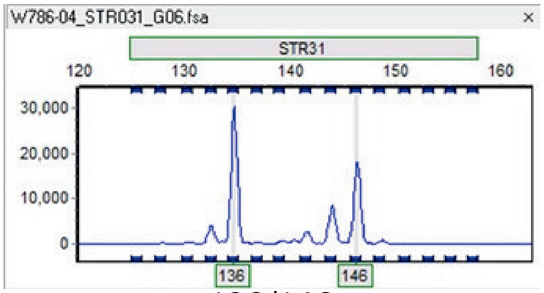

136/146

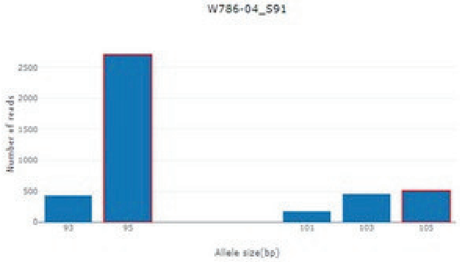

95/105

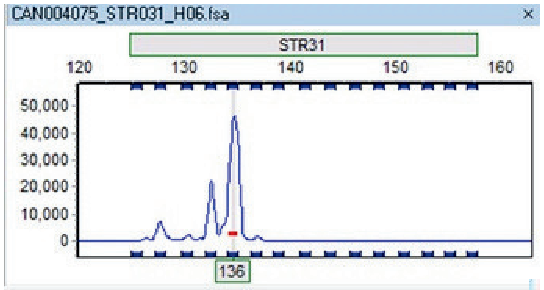

136/136

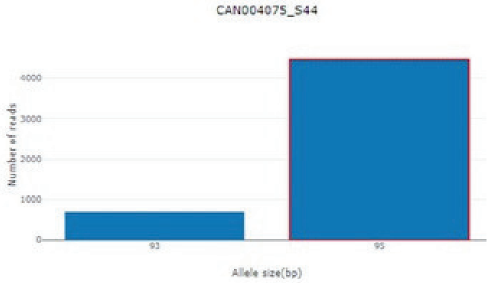

95/95

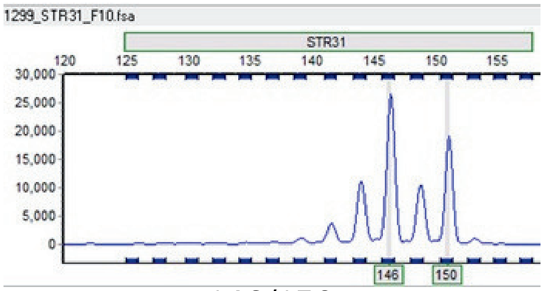

146/150

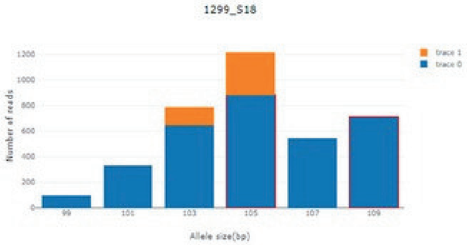

105/109

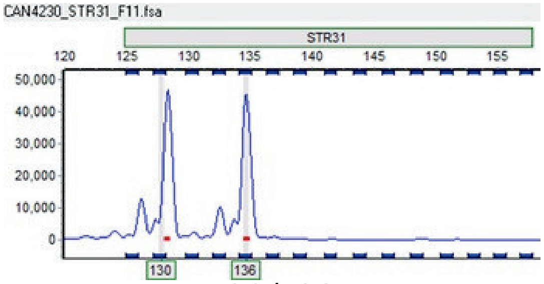

130/136

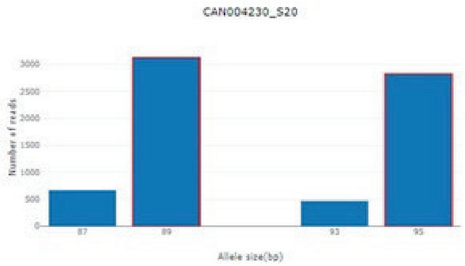

89/95

Capillary Electrophoresis - GeneMarker

Genotyping By Sequencing - SatAnalyzer

CfamSTR031 - Allele size difference - 41 bp

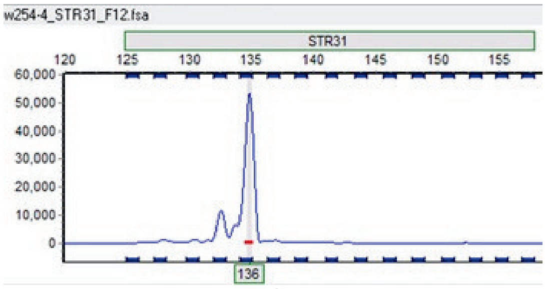

136/136

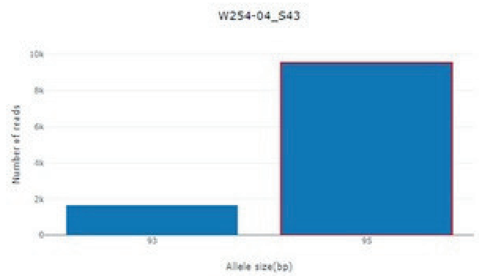

95/95

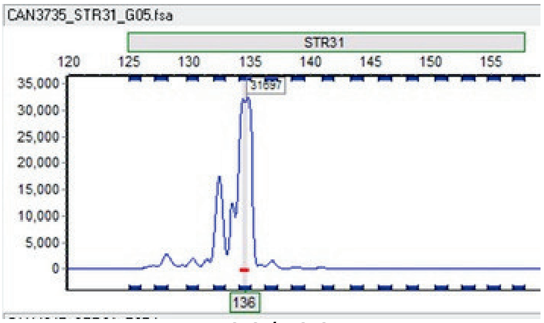

136/136

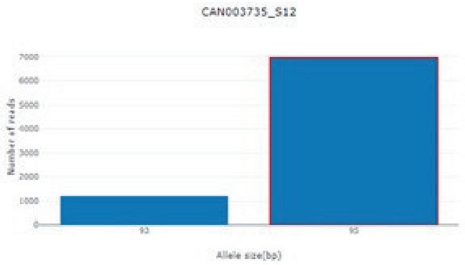

95/95

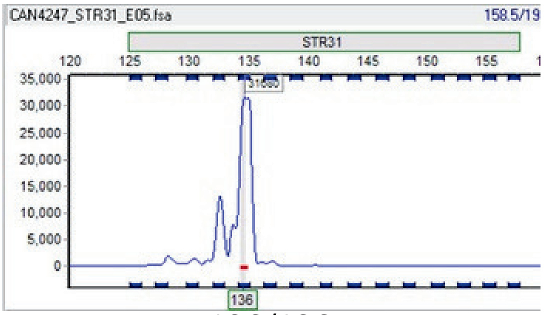

136/136

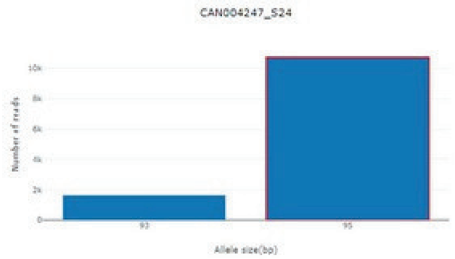

95/95

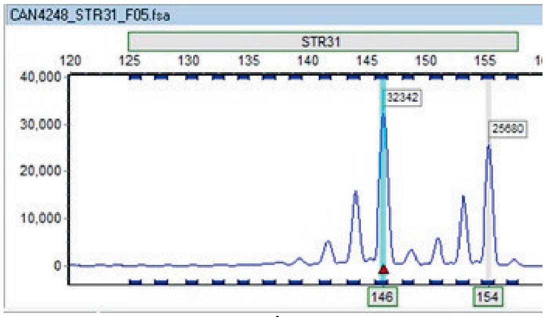

146/154

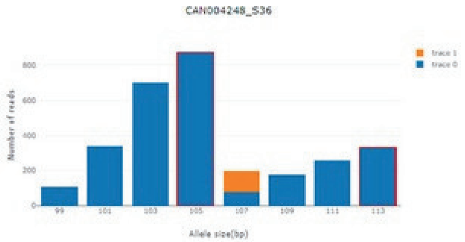

105/113

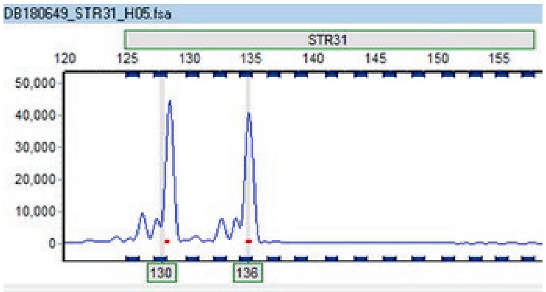

130/136

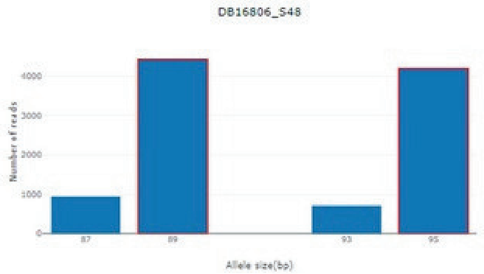

89/95

Capillary Electrophoresis - GeneMarker

Genotyping By Sequencing - SatAnalyzer

CfamSTR032 - Allele size difference - 39 bp

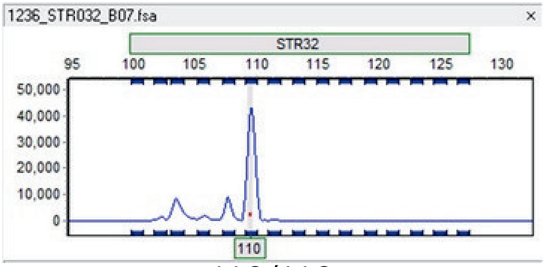

110/110

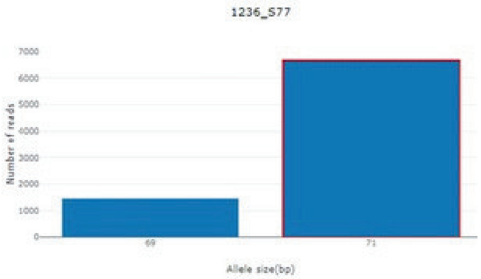

71/71

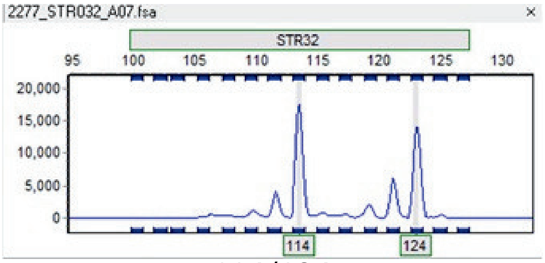

114/124

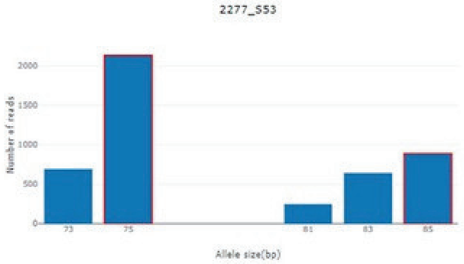

75/85

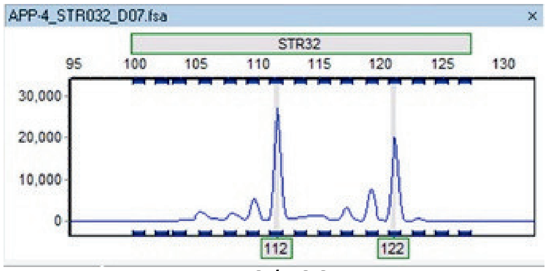

112/122

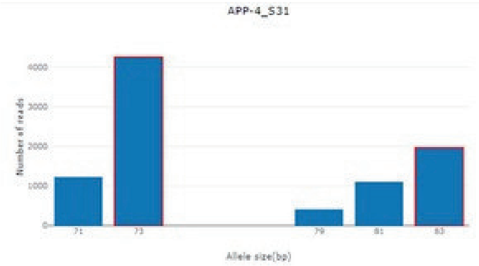

73/83

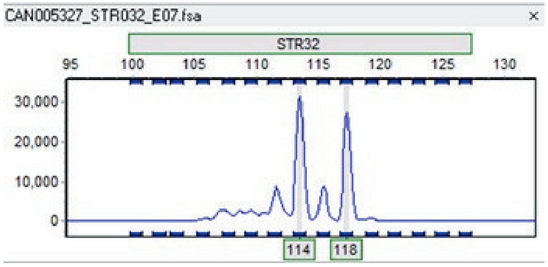

114/118

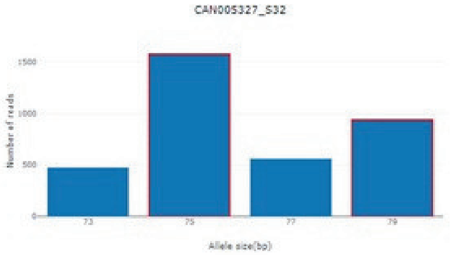

75/79

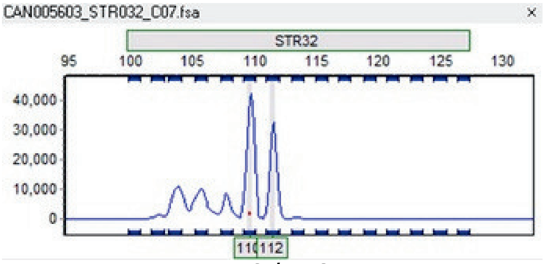

110/112

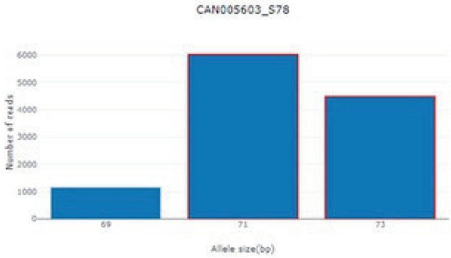

71/73

Capillary Electrophoresis - GeneMarker

Genotyping By Sequencing - SatAnalyzer

CfamSTR032 - Allele size difference - 39 bp

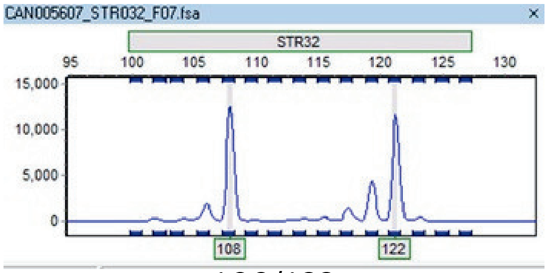

108/122

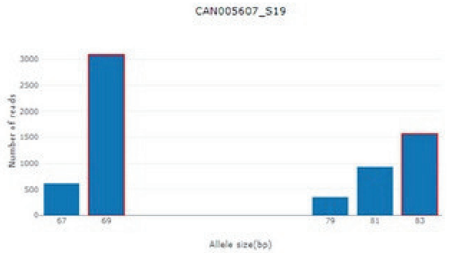

69/83

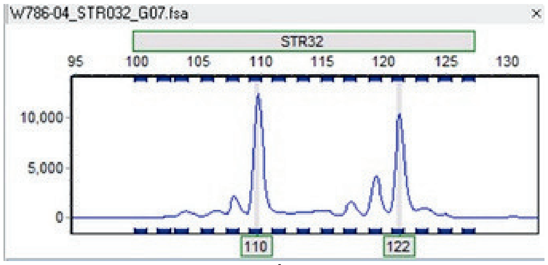

110/122

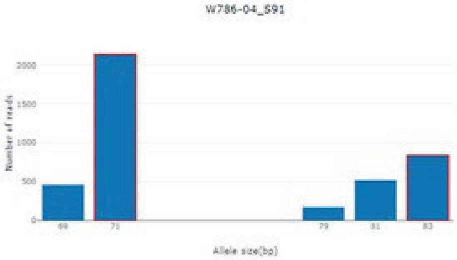

71/83

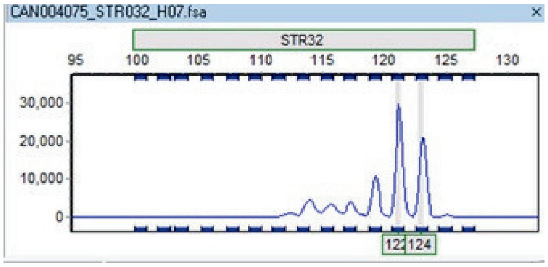

122/124

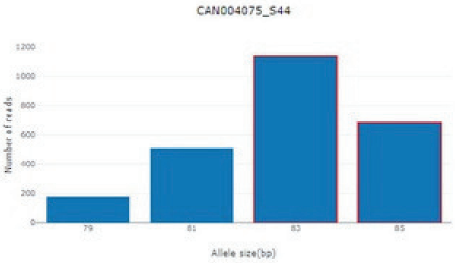

83/85

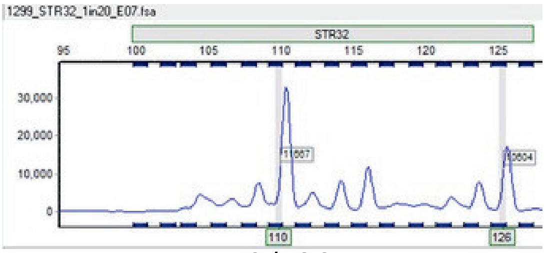

110/126

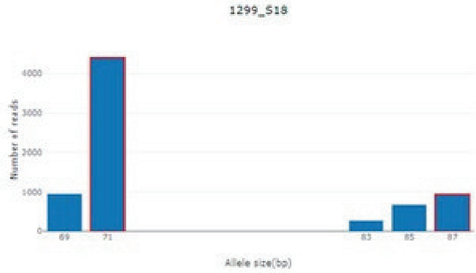

71/87

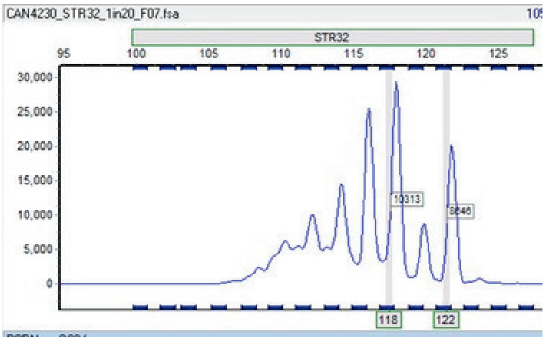

116/122

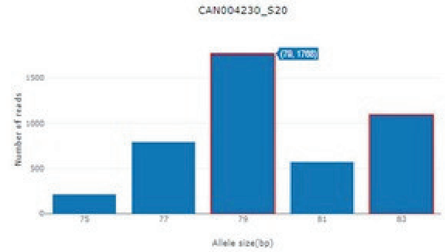

79/83

Capillary Electrophoresis - GeneMarker

Genotyping By Sequencing - SatAnalyzer

CfamSTR032 - Allele size difference - 39 bp

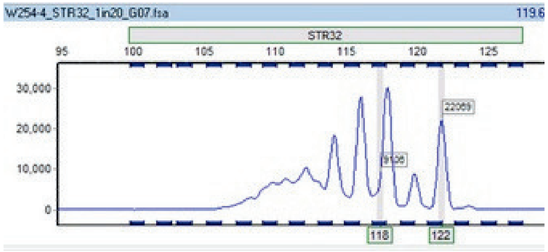

118/122

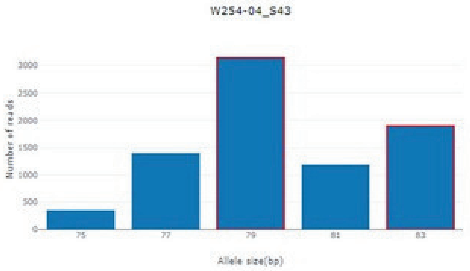

79/83

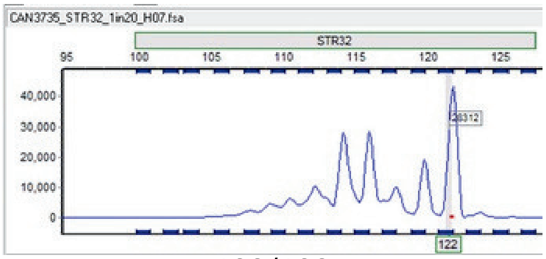

122/122

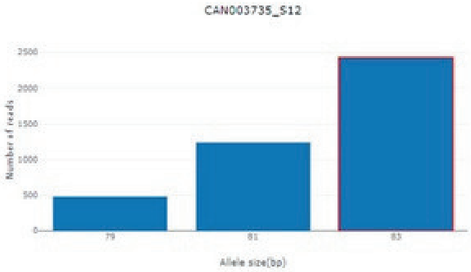

83/83

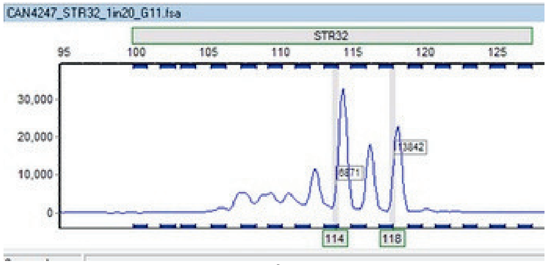

114/118

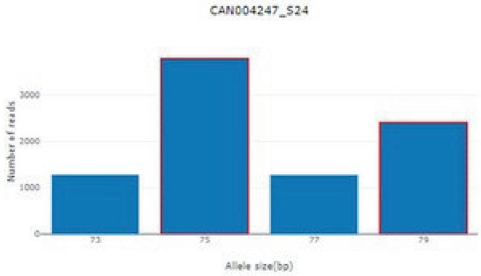

75/79

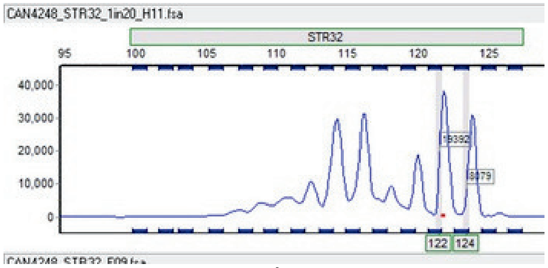

122/124

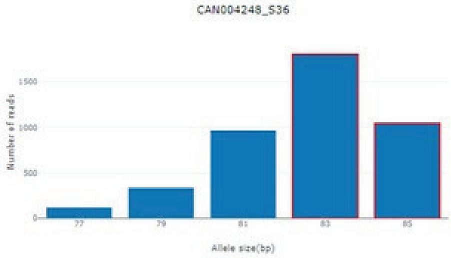

83/85

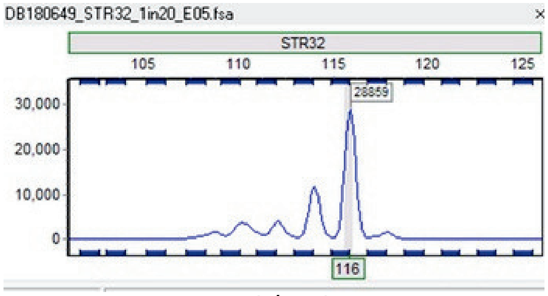

116/116

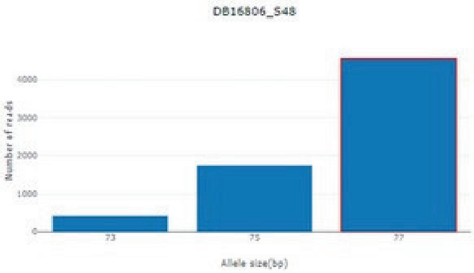

77/77

Capillary Electrophoresis - GeneMarker

Genotyping By Sequencing - SatAnalyzer

CfamSTR033 - Allele size difference - 42 bp

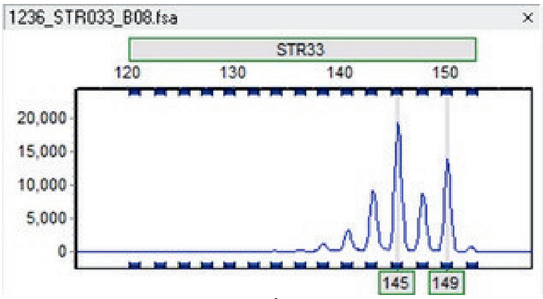

145/149

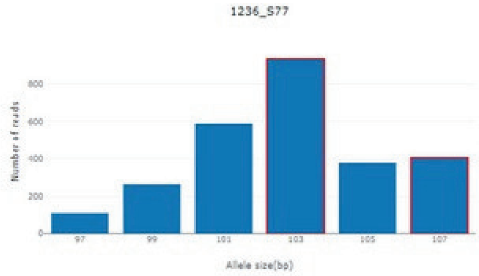

103/107

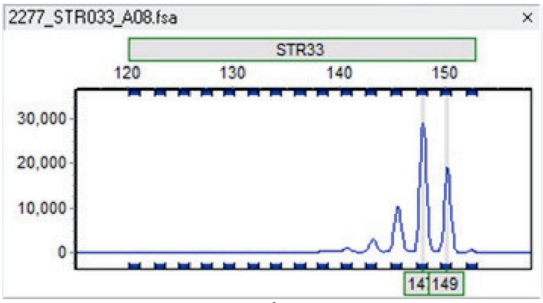

147/149

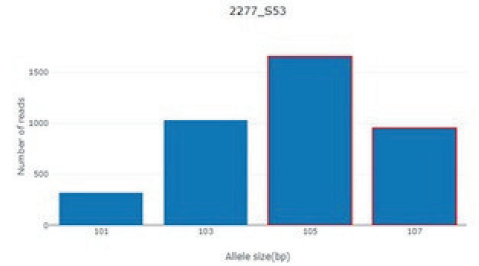

105/107

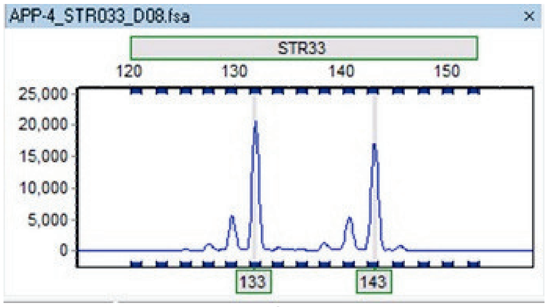

133/143

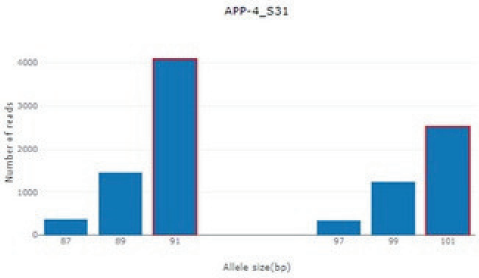

91/101

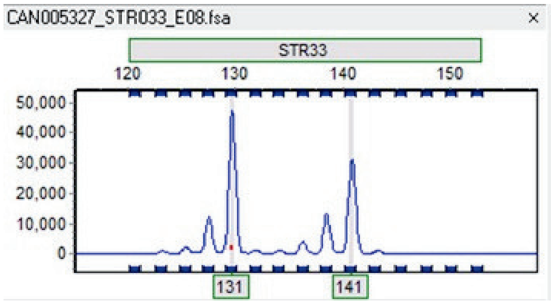

131/141

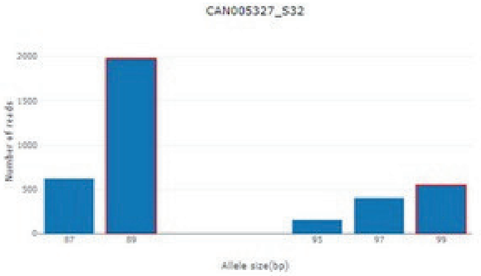

89/99

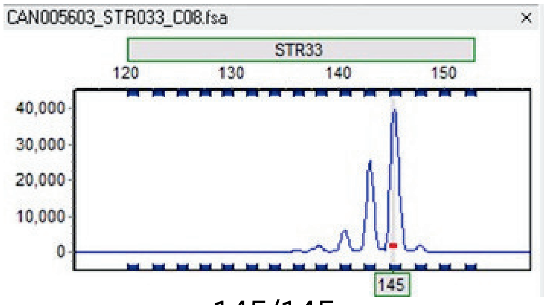

145/145

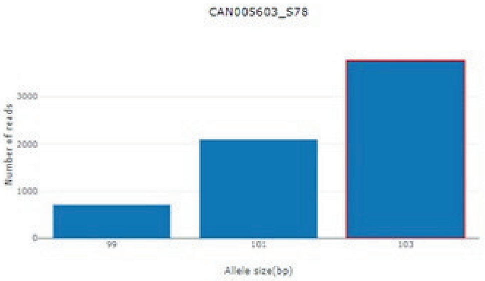

103/103

Capillary Electrophoresis - GeneMarker

Genotyping By Sequencing - SatAnalyzer

CfamSTR033 - Allele size difference - 42 bp

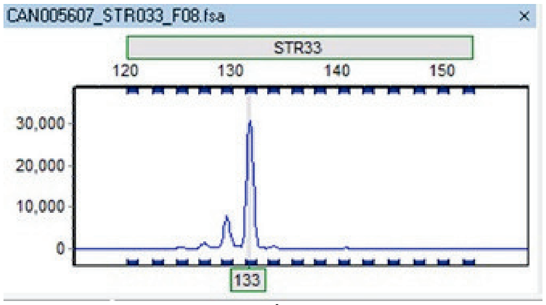

133/133

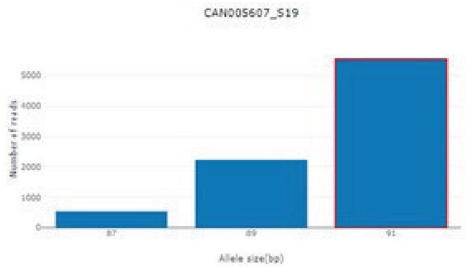

91/91

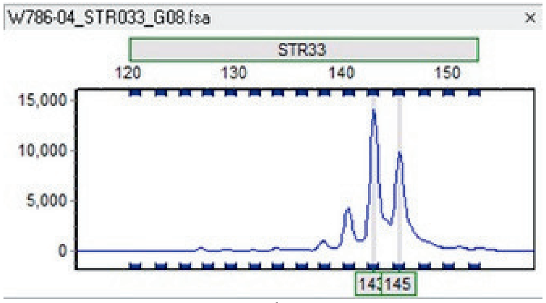

143/145

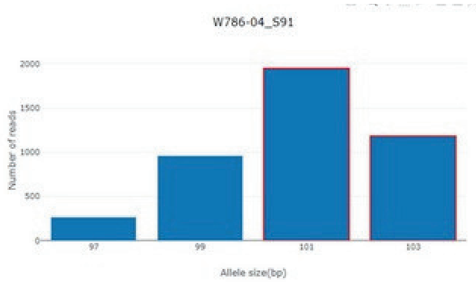

101/103

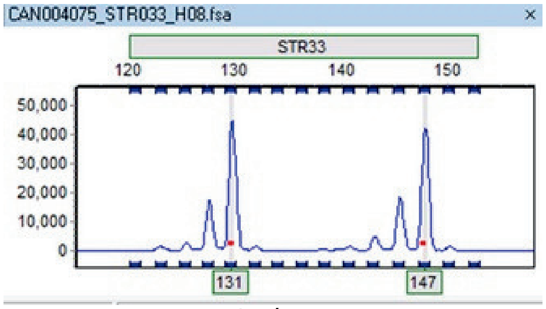

131/147

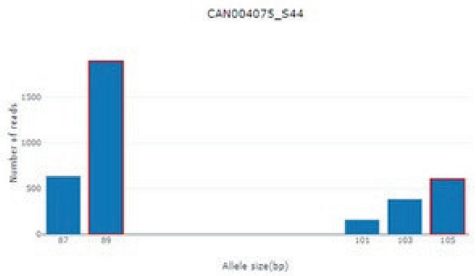

89/105

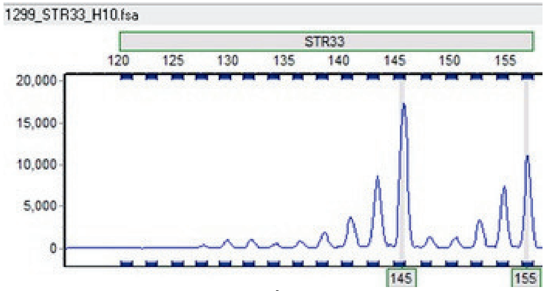

145/155

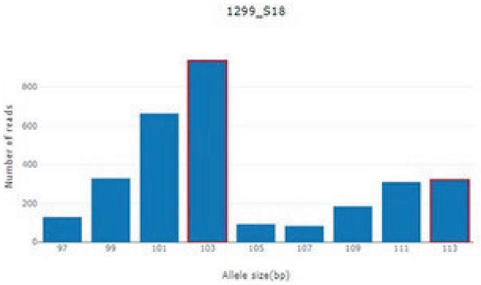

103/113

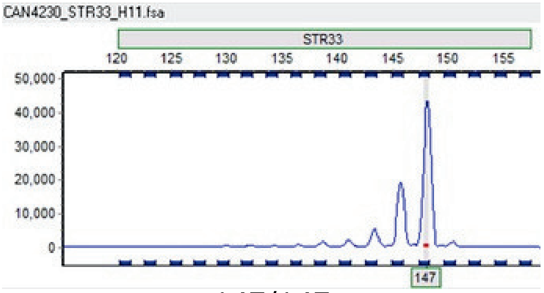

147/147

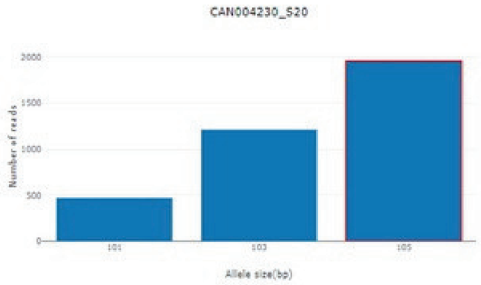

105/105

Capillary Electrophoresis - GeneMarker

Genotyping By Sequencing - SatAnalyzer

CfamSTR033 - Allele size difference - 42 bp

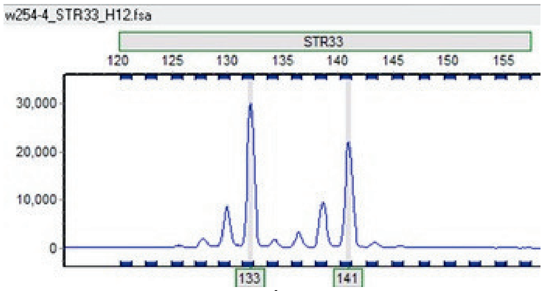

133/141

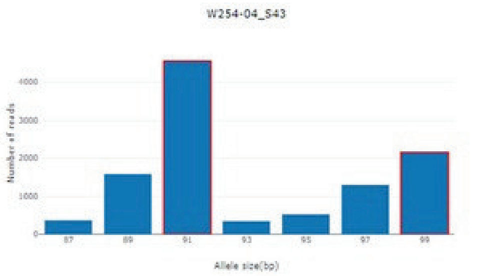

91/99

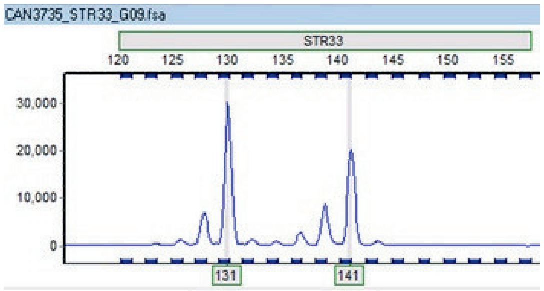

131/141

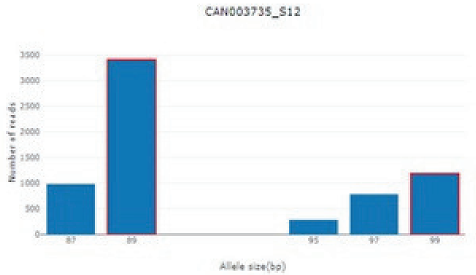

89/99

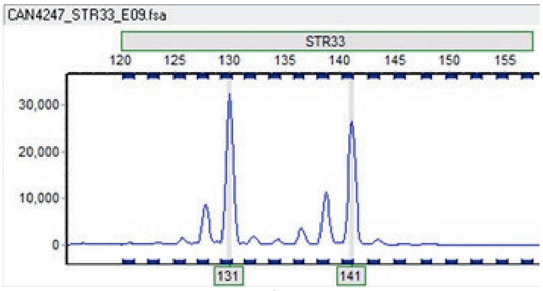

131/141

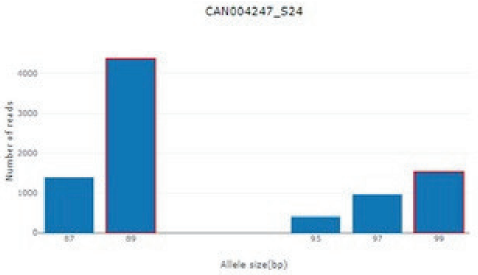

89/99

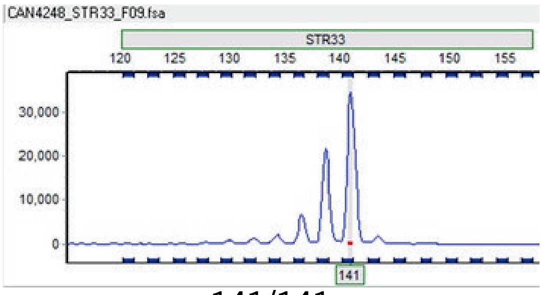

141/141

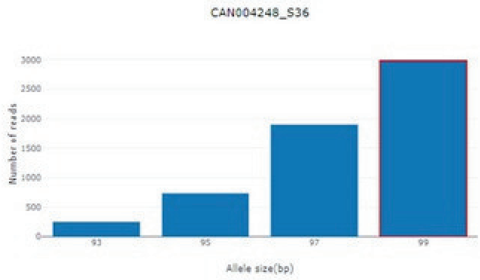

99/99

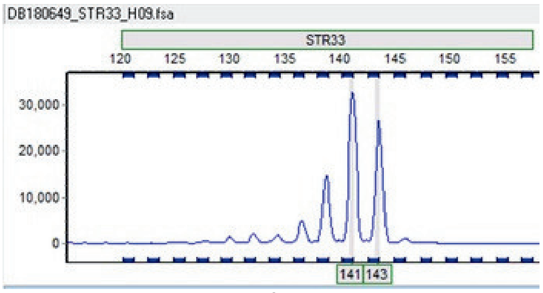

141/143

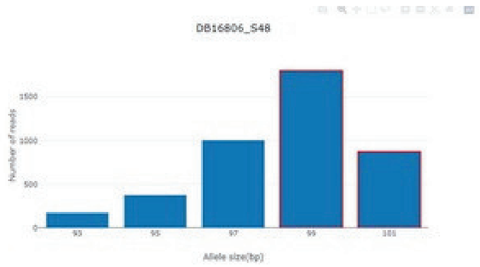

99/101
